# Facile Access to 2-Selenoxo-1,2,3,4-tetrahydro-4-quinazolinone Scaffolds and Corresponding Diselenides via Cyclization between Methyl Anthranilate and Isoselenocyanates: Synthesis and Structural Features

**DOI:** 10.3390/molecules27185799

**Published:** 2022-09-07

**Authors:** Vladimir K. Osmanov, Evgeniy V. Chipinsky, Victor N. Khrustalev, Alexander S. Novikov, Rizvan Kamiloglu Askerov, Alexander O. Chizhov, Galina N. Borisova, Alexander V. Borisov, Maria M. Grishina, Margarita N. Kurasova, Anatoly A. Kirichuk, Alexander S. Peregudov, Andreii S. Kritchenkov, Alexander G. Tskhovrebov

**Affiliations:** 1 Department of Chemistry, R.E. Alekseev Nizhny Novgorod State Technical University, Minin St., 24, 603155 Nizhny Novgorod, Russia; 2 Research Institute of Chemistry, Peoples’ Friendship University of Russia, Miklukho-Maklaya St., 6, 117198 Moscow, Russia; 3 N.D. Zelinsky Institute of Organic Chemistry, Russian Academy of Sciences, Leninsky Prosp., 47, 119334 Moscow, Russia; 4 Institute of Chemistry, Saint Petersburg State University, Universitetskaya Nab., 7/9, 199034 Saint Petersburg, Russia; 5 Department of Organic Chemistry, Baku State University, Z. Xalilov, 23, Baku 1148, Azerbaijan; 6 Institute of Organoelement Compounds of the Russian Academy of Sciences, Vavilov St., 28, 119991 Moscow, Russia; 7 N.N. Semenov Federal Research Center for Chemical Physics, Russian Academy of Sciences, Ul. Kosygina, 4, 119991 Moscow, Russia

**Keywords:** quinazolinones, selenium, heterocycles, chalcogen bonding, cyclization, isoselenocyanates

## Abstract

A practical method for the synthesis of 2-selenoxo-1,2,3,4-tetrahydro-4-quinazolinone was reported. The latter compounds were found to undergo facile oxidation with H_2_O_2_ into corresponding diselenides. Novel organoselenium derivatives were characterized by the ^1^H, ^77^Se, and ^13^C NMR spectroscopies, high-resolution electrospray ionization mass spectrometry, IR, elemental analyses (C, H, N), and X-ray diffraction analysis for several of them. Novel heterocycles exhibited multiple remarkable chalcogen bonding (ChB) interactions in the solid state, which were studied theoretically.

## 1. Introduction

Quinazolinones are an important class of heterocycles, which are widespread in natural alkaloids and synthetic biologically active compounds [1]. Quinazolinone derivatives are known to exhibit hypotensive, anticonvulsant, anti-inflammatory, antibacterial, antimalarial, fungicidal effects, and antiproliferative activity [2,3,4,5,6,7,8,9]. Interestingly, the introduction of the S or Se atoms in 2- or 4-positions of the quinazolinone core results in the enhancement of the anticancer activity [5,6,10,11,12,13,14,15]. 

There are several approaches to the synthesis of heterocyclic thiones and selones described in the literature. The first one includes halogen to sulfur or selenium substitution employing hydrosulphide or hydroselenide or thio- or selenourea [16,17,18,19]. The Se atom can also be conveniently introduced via substitution of the SMe moiety on treatment with NaSeH [20]. Another widely spread approach to the synthesis of sulfur-containing derivatives of quinazolinones involves the reaction between o-aminonitriles or o-aminocarboxylates and isothiocyanates or thiourea. However, this approach has been studied little for the preparation of derivatives of quinazolin-2(1H)-selones, which is probably due to the lower stability and synthetic availability of isoselenocyanates [21,22]. It should be noted that interest in chacogen-containing derivatives of quinazolinones arises due to their potential applications in supramolecular chemistry. Halogen and chalcogen bonding (ChB) is an area of increasing interest, and these weak interactions are often employed for various applications [23,24,25,26,27,28,29,30,31,32,33].

Following our interest in chalcogen heterocycles [34,35,35,36,37] and noncovalent interactions [38,39,40,41,42,43,44,45,46,47], here we reported a convenient synthesis of 2-selenoxo-1,2,3,4-tetrahydro-4-quinazolinones via cyclization reaction between methyl anthranilate and isoselenocyanates. Moreover, we demonstrated that 2-selenoxo-1,2,3,4-tetrahydro-4-quinazolinones undergo facile oxidation under mild conditions to give corresponding diselenides. 

## 2. Results and Discussion

**Scheme 1:**
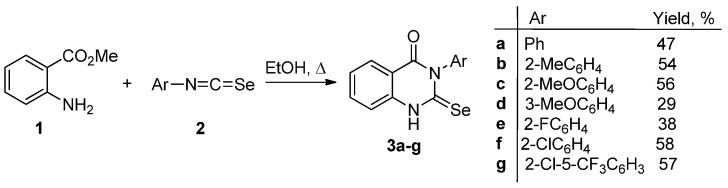
Synthesis of **3a**–**g**.

The addition of isoselenocyanates **2a**–**g** to a solution of methyl anthranilate **1** in ethanol under reflux allowed the preparation of corresponding 2-selenoxo-1,2,3,4-tetrahydro-4-quinazolinones **3a–g** in high yields (Scheme 1).

**Figure 1:**
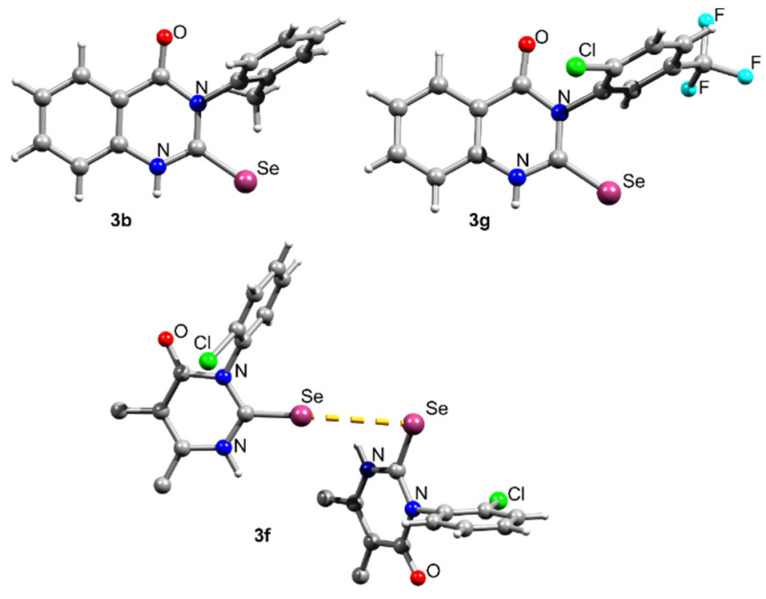
Ball-and-stick representations of **3b**, **3f,** and **3g**. Se···Se ChB for **3f** is depicted as a dashed line. Grey and light grey spheres represent carbon and hydrogen atoms, respectively.

The structures of all new compounds were confirmed by the ^1^H, ^77^Se, and ^13^C NMR spectroscopies; high-resolution electrospray ionization mass spectrometry (HRESI–MS); IR; the elemental analyses (C, H, N); and X-ray diffraction analysis for **3b**, **3f,** and **3g** (Figure 1). Compounds **3b**, **3f**, and **3g** could be recrystallized to furnish monocrystals, suitable for analysis by single crystal X-ray crystallography. The structural investigations confirmed the formation of 2-selenoxo-1,2,3,4-tetrahydro-4-quinazolinones. The plausible mechanism for the formation of **3a**–**g** is depicted in Scheme S1 and is similar to what was observed in the S analogs [15].

Structural investigations revealed that the 2-selenoxo-1,2,3,4-tetrahydro-4-quinazolinone fragment in **3b**, **3f,** and **3g** is virtually planar, and the C=Se distances are within the typical range for the corresponding single bond values. Interestingly, compound **3f** exhibited unsymmetrical supramolecular dimers via type II Se···Se ChB (Figure 1), while **3b** and **3f** were not engaged in ChB, arguably due to the prevalence of other weak interactions in the solid state. Theoretical calculations on the type II Se···Se ChB for compound **3f** are given here further. 

**Scheme 2:**
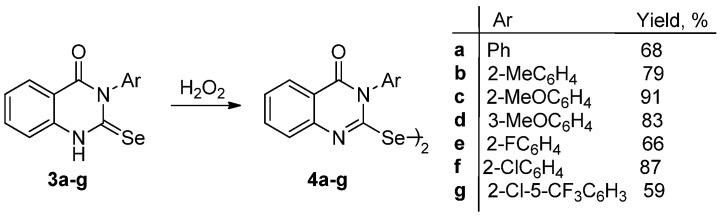
Synthesis of 4**a**–**g**.

When we attempted to recrystallize **3c** from ethanol, its aerobic oxidation coupled with the diselenide formation took place. Similar oxidations were observed earlier in the literature [22,48,49]. We were able to achieve synthetically viable oxidation for **3a**–**g** to furnish **4a**–**g** in good yields employing hydrogen peroxide as an oxidant (Scheme 2).

**Figure 2:**
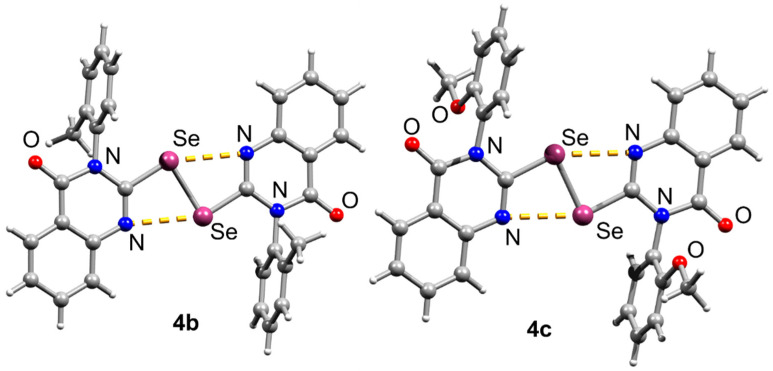
Ball-and-stick representations of **4b** and **4c** demonstrating intramolecular Se···N ChB, depicted as dashed lines. Grey and light grey spheres represent carbon and hydrogen atoms, respectively.

Compounds **4a**–**g** are poorly soluble in common organic solvents; however, we managed to obtain single crystals of **4b** and **4c**, suitable for X-Ray analysis (Figure 2). 

**Figure 3:**
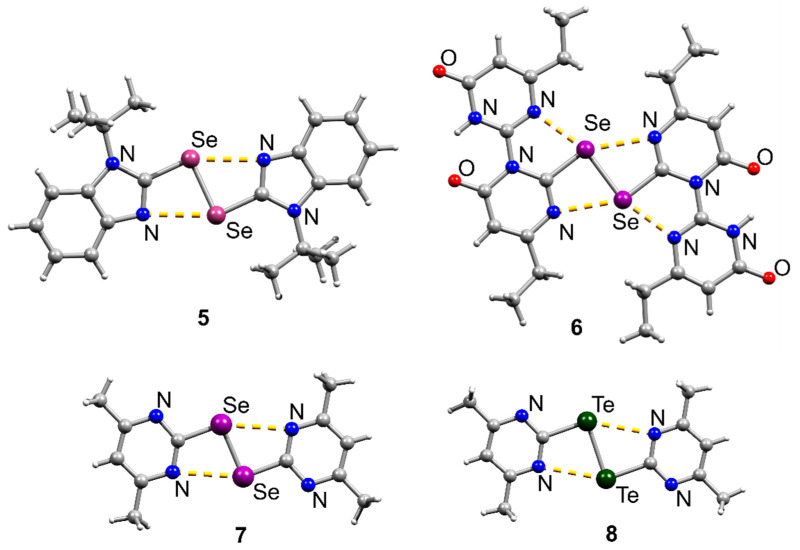
Ball-and-stick representations of **5**–**8** demonstrating intramolecular Se···N ChB, depicted as dashed lines. Grey and light grey spheres represent carbon and hydrogen atoms, respectively.

Both compounds **4b** and **4c** exhibited a pair of intramolecular Se···N ChB (Figure 2). Cambridge Structural Database search revealed that it contained only four other published structures (**5** [49], **6** [50], **7** [51,52], and **8** [52]), which featured such a remarkable pair of intramolecular X···N (X = S, Se, Te) ChB (Figure 3).

Compound **5** is a dibenzimidazole diselenide, as are **4b** and **4c**, which features two intramolecular Se···N ChB. For **6,** the situation is slightly more complicated: each Se atom is involved in two intramolecular Se···N ChB, and overall, the molecule features four Se···N ChB (Figure 3). Compounds **7** [51,52] and **8** [52], which were reported earlier, also featured intramolecular ChB, analogously to **4b** and **4c**.

**Figure 4:**
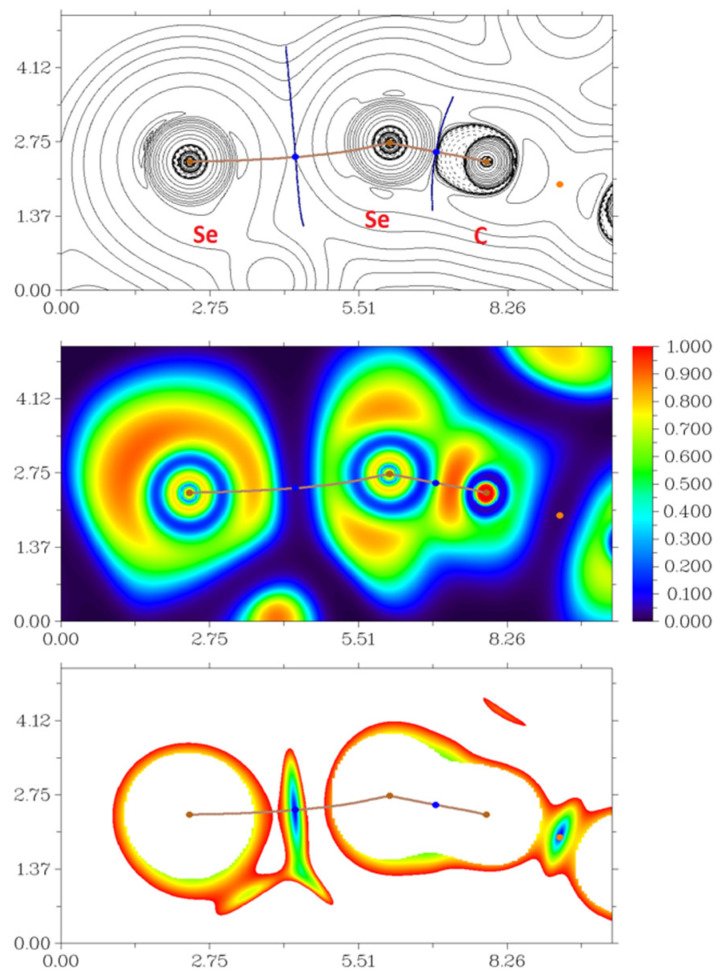
Contour line diagram of the Laplacian of electron density distribution ∇^2^⍴(**r**), bond paths, and selected zero-flux surfaces (**top**), visualization of electron localization function (ELF, **center**), and reduced density gradient (RDG, **bottom**) analyses for contact Se···Se (chalcogen bond) in the X-ray structure **3f**. Bond critical points (3, –1) are shown in blue, nuclear critical points (3, –3)—pale brown, ring critical points (3, +1)—orange, bond paths are shown as pale brown lines, length units—Å, and the color scale for the ELF and RDG maps is presented in a.u.

**Figure 5:**
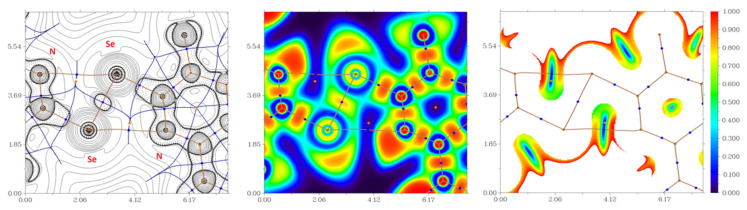
Contour line diagram of the Laplacian of electron density distribution ∇^2^⍴(**r**), bond paths, and selected zero-flux surfaces (**left**), visualization of electron localization function (ELF, **center**), and reduced density gradient (RDG, **right**) analyses for contacts Se–Se and Se···N in the X-ray structure **4b**. Bond critical points (3, –1) are shown in blue, nuclear critical points (3, –3)—pale brown, ring critical points (3, +1)—orange, bond paths are shown as pale brown lines, length units—Å, and the color scale for the ELF and RDG maps is presented in a.u.

**Figure 6:**
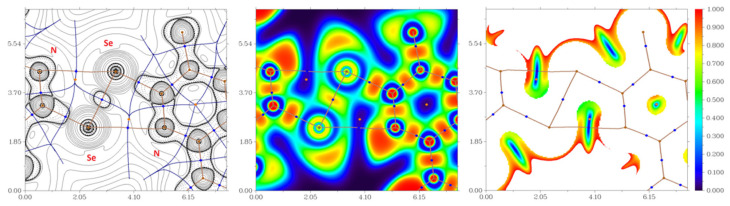
Contour line diagram of the Laplacian of electron density distribution ∇^2^⍴(**r**), bond paths, and selected zero-flux surfaces (**left**), visualization of electron localization function (ELF, **center**), and reduced density gradient (RDG, **right**) analyses for contacts Se–Se and Se···N in the X-ray structure **4c**. Bond critical points (3, –1) are shown in blue, nuclear critical points (3, –3)—pale brown, ring critical points (3, +1)—orange, bond paths are shown as pale brown lines, length units—Å, and the color scale for the ELF and RDG maps is presented in a.u.

**Figure 7:**
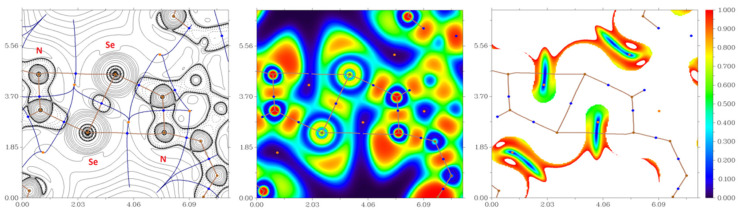
Contour line diagram of the Laplacian of electron density distribution ∇^2^⍴(**r**), bond paths, and selected zero-flux surfaces (**left**), visualization of electron localization function (ELF, **center**), and reduced density gradient (RDG, **right**) analyses for contacts Se–Se and Se···N in the X-ray structure **5**. Bond critical points (3, –1) are shown in blue, nuclear critical points (3, –3)—pale brown, ring critical points (3, +1)—orange, bond paths are shown as pale brown lines, length units—Å, and the color scale for the ELF and RDG maps is presented in a.u.

**Figure 8:**
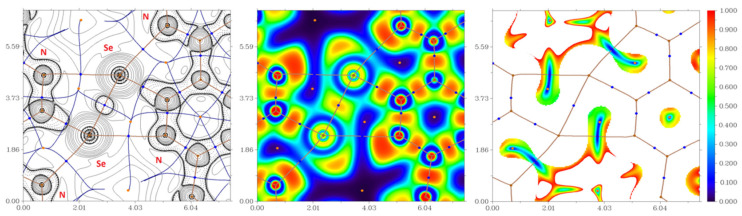
Contour line diagram of the Laplacian of electron density distribution ∇^2^⍴(**r**), bond paths, and selected zero-flux surfaces (**left**), visualization of electron localization function (ELF, **center**), and reduced density gradient (RDG, **right**) analyses for contacts Se–Se and Se···N in the X-ray structure **6**. Bond critical points (3, –1) are shown in blue, nuclear critical points (3, –3)—pale brown, ring critical points (3, +1)—orange, bond paths are shown as pale brown lines, length units—Å, and the color scale for the ELF and RDG maps is presented in a.u.

**Figure 9:**
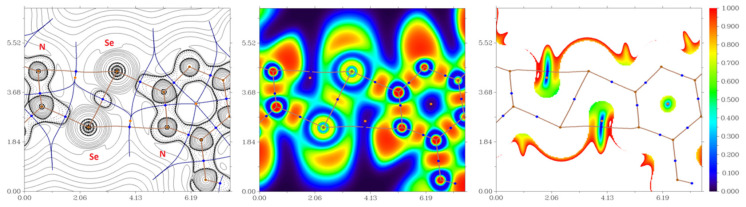
Contour line diagram of the Laplacian of electron density distribution ∇^2^⍴(**r**), bond paths, and selected zero-flux surfaces (**left**), visualization of electron localization function (ELF, **center**), and reduced density gradient (RDG, **right**) analyses for contacts Se–Se and Se···N in the X-ray structure **7**. Bond critical points (3, –1) are shown in blue, nuclear critical points (3, –3)—pale brown, ring critical points (3, +1)—orange, bond paths are shown as pale brown lines, length units—Å, and the color scale for the ELF and RDG maps is presented in a.u.

**Figure 10:**
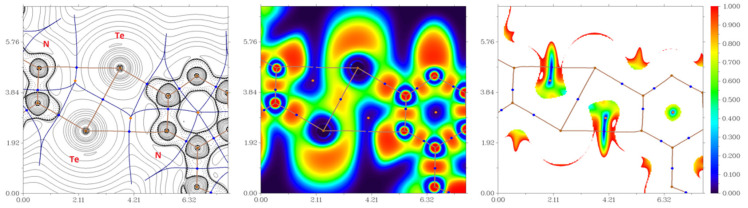
Contour line diagram of the Laplacian of electron density distribution ∇^2^⍴(**r**), bond paths, and selected zero-flux surfaces (**left**), visualization of electron localization function (ELF, **center**) and reduced density gradient (RDG, **right**) analyses for contacts Te–Te and Te···N in the X-ray structure **8**. Bond critical points (3, –1) are shown in blue, nuclear critical points (3, –3)—pale brown, ring critical points (3, +1)—orange, bond paths are shown as pale brown lines, length units—Å, and the color scale for the ELF and RDG maps is presented in a.u.

**Figure 11:**
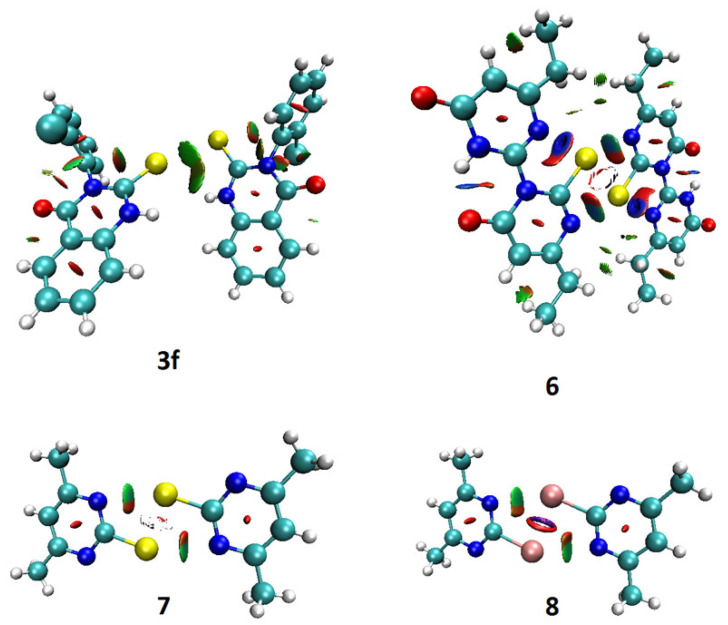
Visualization of noncovalent interactions Se···Se, Se···N, and Te···N in 3D using NCI analysis technique in model supramolecular associates **3f**, **6**, **7**, and **8**.

In order to theoretically study chalcogen bonds Se···Se, Se···N, and Te···N observed in the X-ray structures **3f**, **4b**, **4c**, **5**, **6**, **7**, and **8**, the DFT calculations followed by the topological analysis of the electron density distribution within the QTAIM approach [53] were carried out for model supramolecular associates (see Computational details and Table S1 in Supplementary Materials). The results of the QTAIM analysis are summarized in Table 1. The contour line diagrams of the Laplacian of electron density distribution ∇^2^⍴(**r**), bond paths, and selected zero-flux surfaces; visualization of electron localization function (ELF); and reduced density gradient (RDG) analyses for contacts Se···Se, Se···N, and Te···N in the X-ray structures **3f**, **4b**, **4c**, **5**, **6**, **7**, and **8** are shown in Figure 4, Figure 5, Figure 6, Figure 7, Figure 8, Figure 9 and Figure 10; the visualization of these noncovalent interactions in 3D using NCI analysis technique [54] is shown in Figure 11.

The QTAIM analysis of model supramolecular associates demonstrates the presence of bond critical points (3, –1) for contacts Se···Se, Se···N, and Te···N in the X-ray structures **3f**, **4b**, **4c**, **5**, **6**, **7**, and **8** (Table 1 and Figure 4, Figure 5, Figure 6, Figure 7, Figure 8, Figure 9 and Figure 10). The low magnitude of the electron density, positive values of the Laplacian of electron density, and very close to zero energy density in bond critical points (3, –1) for chalcogen bonds Se···Se (**3f**) and Se···N (**4b**, **4c**, **5**, **6**, and **7**) or Te···N (**8**) in studied model supramolecular associates, as well as their estimated strength, are typical for noncovalent interactions involving chalcogen atoms [34,35,35,36,37,57,58,59,60,61,62], in contrast with these descriptors (viz. relatively large magnitude of the electron density, negative Laplacian of electron density, and large negative energy density) for covalent bonds Se–Se and Te–Te in **4b**, **4c**, **5**, **6**, **7**, and **8**. The sign of λ_2_ can be utilized to distinguish bonding (attractive, λ_2_ < 0) interactions from nonbonding ones (repulsive, λ_2_ > 0) [54,63], which allows us to conclude that chalcogen bonds contact Se···Se, Se···N, and Te···N in the X-ray structures **3f**, **4b**, **4c**, **5**, **6**, **7**, and **8** are attractive in nature and purely noncovalent (in all cases, except Se···N interactions (2.479 Å) in **6**, which has some covalent contribution), because the balance between the Lagrangian kinetic energy G(**r**) and potential energy density V(**r**) at the appropriate bond critical points (3, –1) for these contacts is –G(**r**)/V(**r**) > 1 [64].

## 3. Materials and Methods

Methyl anthranilate (Acros Organics, Belgium) was used in this work without additional purification. The isoselenocyanates **2 a**–**g** used in this work were obtained by the literature method [65]. Isoselenocyanates **2b, c, g** were purified by recrystallization from hexane at −20 °C. Ethanol was dried by distillation over CaO and CaH_2_.

All melting points were determined with a “Stuart SMP3” melting point apparatus. Infrared spectra were recorded on the “Shimadzu IR Prestige-21” (Kyoto, Japan) instrument in KBr disk (4000–400 cm^−1^). High-resolution mass spectra (HR-MS) were measured on a “Bruker micrOTOF II” (Karlsruhe, Germany) instrument using electrospray ionization (ESI). The measurements were performed in a positive ion mode (interface capillary voltage −4500 V); mass range from *m/z* 50 to m/z 30 0 0 Da; internal calibration was performed with Electrospray Calibrant Solution («Agilent Tuning Mix», «Agilent»). The most intensive peak in the isotopic pattern was reported. A syringe injection was used for solutions in acetonitrile (flow rate 5 McL/min). Nitrogen was applied as a dry gas; interface temperature was set at 180 °C.

^1^H, COSY, ^13^C-NMR, DEPT, HSQC, and HMBC spectra compounds **3a**–**f** were measured on an “Agilent DD2 400” spectrometer (400 MHz for ^1^H and 100.60 MHz for ^13^C, Santa Clara, CA, USA) using DMSO-d6 as the NMR solvents. Chemical shifts were indicated in parts per million (ppm) relative to tetramethylsilane as an internal standard. The ^77^Se-NMR spectra compound **3a-f** were measured on an “Agilent DD2 400” spectrometer at 76.30 MHz using diphenylselenide as a standard. The ^19^F-NMR spectra compound **3e** were measured on an “Agilent DD2 400” spectrometer at 376.30 MHz using trichlorofluoro-methane as a standard. The ^1^H, COSY, ^13^C, JMODECHO, HSQC, and HMBC compounds **3g** were measured on a “Bruker Avance^TM^ 600” (Karlsruhe, Germany) spectrometer (600 MHz for ^1^H and 150.925 MHz for ^13^C) using DMSO-d6 as the NMR solvents. The ^1^H, COSY, ^13^C, JMODECHO, HSQC, and HMBC compounds **4a**–**g** were measured on a “Bruker Avance^TM^ 500” spectrometer (500 MHz for ^1^H and 125.72 MHz for ^13^C) using DMSO- *d*6 as the NMR solvents. The ^77^Se-NMR spectra compounds, **3g** and **4a**–**g,** were measured on a “Bruker Avance^TM^ 400” spectrometer at 76.35 MHz and referenced to diphenylselenide, using DMSO- *d*6 as the NMR solvents. The ^19^F-NMR spectra compounds, **3g**, **4e,** and **4g,** were measured on a “Bruker Avance^TM^ 300” spectrometer at 282.38 MHz and referenced to trichlorofluoromethane, using DMSO-d6 as NMR solvent.

### 3.1. Synthetic Part

**Synthesis of compounds 3a**–**g** (general method). To a solution (0.01 mol) of methyl anthranilate, **1** in 100 mL of absolute ethanol (0.01 mol) of the corresponding isoselenocyanate **2 a**–**g** in 20 mL of absolute ethanol was added, boiled for 6 h, then cooled to 0 °C. Precipitates precipitated from the solution were separated by filtration, washed with ethanol (2 × 25 mL), and dried at 40 °C.


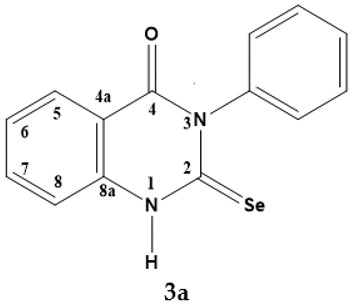


3-phenyl-2-selenoxo-2,3-dihydroquinazolin4(1*H*)-one. Light yellow solid (47%), mp 235 °C. Anal. Calcd. for C_14_H_10_N_2_OSe: C 55.82; H 3.35; N 9.30. Found: C 55.72; H 3.30; N 9.38. ESI^+^-MS, *m*/*z*: calcd for [C_14_H_10_N_2_OSe + H]^+^ 303.0031, found 303.0036 [C_14_H_10_N_2_OSe + H]^+^. IR (KBr, selected bands, sm^−1^): 3244, 1664, 1621, 1528, 1488, 1266, 1189, 759, 691. ^1^H NMR (400 MHz, DMSO-*d*_6_), δ (ppm): 13.46 (bs, 1H, NH), 7.93 (dd, *J* = 8.0, *J* = 1.0, 1H, H-5), 7.77 (t, 1H, H-7), 7.51 (d, *J* = 8.0 Hz, 1H, H-8), 7.46 (m, 2H, Ph), 7,39 (m, 2H, Ph and H-6), 7.24 (m, 2H, Ph). ^13^C NMR (100.60 MHz, DMSO-*d*_6_), δ (ppm): 176.4 (C=Se), 159.3 (C=O), 141.2 (C, Ph), 140.4 (C-8a), 136.2 (C-7), 129.5 (2CH, Ph), 129.3 (2CH, Ph), 128.7 (CH, Ph), 127.9 (C-5), 125.4 (C-6), 117.2 (C-4a), 117.2 (C-4a), 116.5 (C-8). ^77^Se NMR (76.30 MHz, DMSO-*d*6), δ (ppm): 462.0 (s).


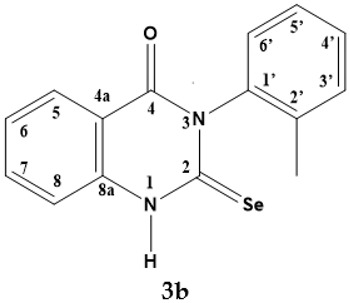


3-(2-methylphenyl)-2-selenoxo-2,3-dihydro-quinazolin- 4(1*H*)-one. Light brown solid (54%), mp 210 °C. Anal. Calcd. for C_15_H_12_N_2_OSe: 57.15; H 3.84; N 8.89. Found: C 57.35; H 3.72; N 8.78. ESI^+^-MS, *m*/*z*: calcd for [C_15_H_12_N_2_OSe + H]^+^ 317.0188, found 317.0184 [C_15_H_12_N_2_OSe + H]^+^. IR (KBr, selected bands, sm^−1^): 3241, 1702, 1619, 1520, 1410, 1262, 1189, 753. ^1^H NMR (400 MHz, DMSO-*d*_6_), δ (ppm): 13.59 (bs, 1H, NH), 7.95 (d, *J* = 7.5, 1H, H-5), 7.80 (m, 1H, H-7), 7.55 (d, *J* = 8.0, 1H, H-8), 7.41 (t, 1H, H-6), 7,26-7,33 (m, 3H, Ar), 7.20 (d, *J* = 10.0, 1H, Ar), 2.03 (s, 3H, CH_3_). ^13^C NMR (100.60 MHz, DMSO-*d*_6_), δ (ppm): 175.7 (C=Se), 158.7 (C=O), 140.5 (C-8a), 140.0 (C-1′), 136.4 (C-7), 135.5 (C-5′), 130.9 (C-3′), 129.4 (C-6′); 129.0 (C-4′), 128.0 (C-5), 127.2 (C-2′), 125.6 (C-6), 116.9 (C-4a), 116.6 (C-8), 17.55 (CH_3_). ^77^Se NMR (76.30 MHz, DMSO-*d*6), δ (ppm): 442.5 (s).


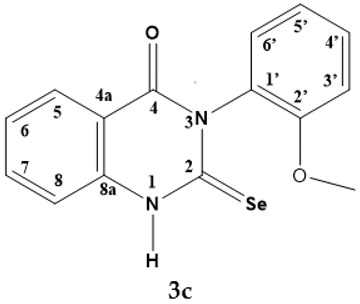


3-(2-methoxyphenyl)-2-selenoxo-2,3-dihydroquinazolin- 4(1*H*)-one. Yellow solid (56%), mp 250 °C. Anal. Calcd. for C_15_H_12_N_2_O_2_Se: C 54.39; H 3.65; N 8.46. Found: C 54.24; H 3.60; N 8.35. ESI^+^-MS, *m*/*z*: calcd for [C_15_H_12_N_2_O_2_Se + H]^+^ 333.0137, found 333.0137 [C_15_H_12_N_2_O_2_Se + H]^+^. IR (KBr, selected bands, sm^−1^): 2946, 1711, 1621, 1533, 1420, 1265, 1190, 1020, 752. ^1^H NMR (400 MHz, DMSO-*d*_6_), δ (ppm): 13.53 (bs, 1H, NH), 7.93 (d, *J* = 8.0, 1H, H-5), 7.80 (m, 1H, H-7), 7.54 (d, *J* = 8.5, 1H, H-8), 7.40 (m, 2H, H-4′ and H-6), 7.23 (dd, *J* = 8.0, *J* = 1.5, 1H, H-6′), 7.14 (d, *J* = 8.0, 1H, H-3′), 7.02 (t, 1H, H-5′), 3.70 (s, 3H, OCH_3_). ^13^C NMR (100.60 MHz, DMSO-*d*_6_), δ (ppm): 176.6 (C=Se), 158.7 (C=O), 154.7 (C-2′-O), 140.4 (C-8a), 136.4 (C-7), 130.6 (C-6′), 130.4 (C-4′), 129.4 (C-1′), 128.0 (C-5), 125.5 (C-6), 121.0 (C-5′), 116.7 (C-4a), 116.5 (C-8), 112.8 (C-3′), 56.18 (OCH_3_). ^77^Se NMR (76.30 MHz, DMSO-*d*6), δ (ppm): 441.6 (s).


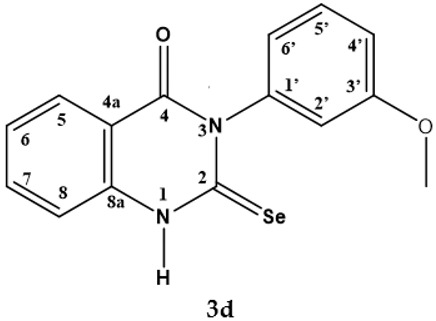


3-(3-methoxyphenyl)-2-selenoxo-2,3-dihydro-quinazolin- 4(1*H*)-one. Light beige solid (29%), mp 222 °C. Anal. Calcd. for C_15_H_12_N_2_O_2_Se: C 54.39; H 3.65; N 8.46. Found: C 54.31; H 3.61; N 8.40. ESI^+^-MS, *m*/*z*: calcd for [C_15_H_12_N_2_O_2_Se + H]^+^ 333.0128, found 333.0137 [C_15_H_12_N_2_O_2_Se + H]^+^. IR (KBr, selected bands, sm^−1^): 2943, 1665, 1525, 1377, 1262, 759. ^1^H NMR (400 MHz, DMSO-*d*_6_), δ (ppm): 13.49 (bs, 1H, NH), 7.93 (dd, *J* = 8.0, *J* = 1.6, 1H, H-5), 7.78 (m, 1H, H-7), 7.53 (d, *J* = 8.4, 1H, H-8), 7.39 (m, 1H, H-6), 7.36 (t, 1H, H-5′), 6.97 (dd, *J* = 8.4, *J* = 2.5, 1H, H-6′), 6.90 (t, 1H, H-2′), 6.85 (d, *J* = 8.0,1H, H-4′), 3.74 (s, 3H, OCH_3_). ^13^C NMR (100.60 MHz, DMSO-*d*_6_), δ (ppm): 176.3 (C=Se), 160.1 (C3′-O), 159.2 (C=O), 142.2 (C-1′), 140.4 (C-8a), 136.1 (C-7), 129.9 (C-5′), 127.9 (C-5), 125.4 (C-6), 121.7 (C-4′), 117.2 (C-4a), 116.5 (C-8), 115.5 (C-2′), 114.2 (C-6′), 55.7 (OCH_3_). ^77^Se NMR (76.30 MHz, DMSO-*d*6), δ (ppm): 457.8 (s).


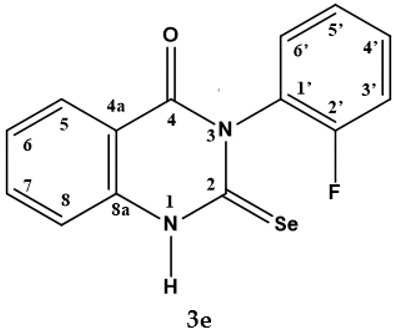


3-(2-fluorophenyl)-2-selenoxo-2,3-dihydroquina-zolin-4(1*H*)-one. Light green solid (38%), mp 212 °C. Anal. Calcd. for C_14_H_9_FN_2_OSe: C 52.68; H 2.84; N 8.78. Found: C 52.62; H 2.87; N 8.67. ESI^+^-MS, *m*/*z*: calcd for [C_14_H_9_FN_2_OSe + H]^+^ 320.9937, found 320.9941 [C_14_H_9_FN_2_OSe + H]^+^. IR (KBr, selected bands, sm^−1^): 3209, 1697, 1670, 1621, 1527, 1263, 1182. ^1^H NMR (400 MHz, DMSO-*d*_6_), δ (ppm): 13.71 (bs, 1H, NH), 7.96 (dd, *J* = 8.0, *J* = 1.2, 1H, H-5), 7.82 (m, 1H, H-7), 7.55 (d, *J* = 8.0, 1H, H-8), 7.28–7.52 (m, 5H, H-6, 4H Ar). ^13^C NMR (100.60 MHz, DMSO-*d*_6_), δ (ppm): 176.1 (C=Se), 158.7 (C=O), 157.6 (d, *^1^J* (^13^C-^19^F) = 310.5, C-2′-F,), 140.4 (C-8a), 136.6 (C-7), 131.8 (C-5′), 131.3 (d, *^3^J*(^13^C-^19^F) = 10.0, C-4′), 128.3 (d., *^2^J*(^13^C-^19^F, C-1′) = 16.5), 128.0 (C-5), 125.80 (C-6), 125.3 (d, *^3^J*(^13^C-^19^F) = 4.5, C-6′), 116.7 (C-8), 116.5 (C-4a), 116.4 (d, *^2^J*(^13^C-^19^F) = 24.3, C-3′). ^19^F NMR (376.30 MHz, DMSO-*d*6), δ (ppm): −122.96 (m, 1F). ^77^Se NMR (76.30 MHz, DMSO-*d*6), δ (ppm): 447.0 (d, *J* = 2.5).


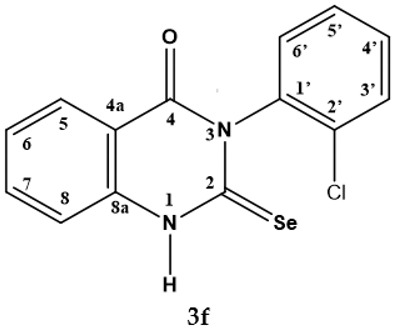


3-(2-chlorophenyl)-2-selenoxo-2,3-dihydroquinazolin-4(1*H*)-one. Beige solid (58%), mp 225 °C. Anal. Calcd. for C_14_H_9_ClN_2_OSe: C 50.10; H 2.70; N 8.35. Found: C 50.16; H 2.67; N 8.27. ESI^+^-MS, *m*/*z*: calcd for [C_14_H_9_ClN_2_OSe + H]^+^ 336.9639, found 336.9640 [C_14_H_9_ClN_2_OSe + H]^+^. IR (KBr, selected bands, sm^−1^): 3210, 1706, 1676, 1619, 1526, 1486, 1410, 1262, 1189, 758. ^1^H NMR (400 Mm, Hz, DMSO-*d*_6_), δ (ppm): 13.69 (bs, 1H, NH), 7.97 (d, *J* = 8.0, 1H, H-5), 7.82 (m, 1H, H-7), 7.60 (m, 1H, Ar), 7.55 (d, *J* = 8.0, 1H, H-8), 7.50 (m, 1H, Ar), 7.46 (m, 2H, Ar), 7.42 (t, 1H, H-6). ^13^C NMR (100.60 MHz, DMSO-*d*_6_), δ (ppm): 175.7 (C=Se), 158.6 (C=O), 140.4 (C-8a), 138.2 (C Ar), 136.6 (C-7), 131.93 (C, Ar), 131.86 (CH, Ar), 130.8 (CH, Ar), 130.1 (CH, Ar), 128.5 (CH, Ar), 128.0 (C-5), 125.7 (C-6), 116.7 (C-8), 116.6 (C-4a). ^77^Se NMR (76.30 MHz, DMSO-*d*6), δ (ppm): 450.0 (s).


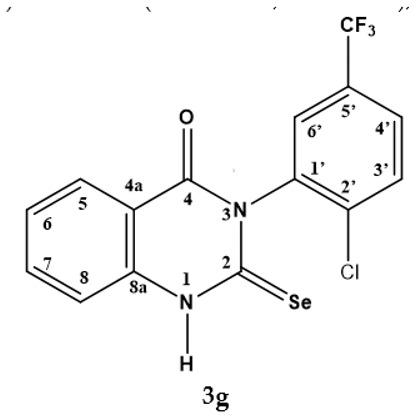


3-[2-chloro-5-(trifluoromethyl)phenyl]-2-selenoxo-2,3-dihydroquinazolin-4(1*H*)-one. Beige solid (57%), mp 201 °C. Anal. Calcd. for C_15_H_8_ClF_3_N_2_OSe: C 44.63%, H 2.00%, N 6.94%. Found: C 44.52; H 2.06; N 6.87. ESI^+^-MS, *m*/*z*: calcd. for [C_15_H_8_ClF_3_N_2_OSe + H]^+^ 404.9513, found 404.9502 [C_15_H_8_ClF_3_N_2_OSe + H]^+^. IR (KBr, selected bands, sm^−1^): 3164, 3113, 3019, 2958, 1718, 1701, 1621, 1534, 1328, 1190, 1175, 1132, 756. ^1^H NMR (600 MHz, DMSO-*d*_6_), δ (ppm): 13.86 (bs, 1H, NH), 8.10 (d, *J* = 1.9, 1H, H-6′), 8.00 (dd, *J* = 7.9, *J* = 0.9, 1H, H-5), 7.84–7.90 (m, 3H, H-7, H-3′, H-4′), 7.58 (d, 1H, H-8), 7.46 (t, 1H, H-6). ^13^C NMR (150.925 MHz, DMSO-*d*_6_), δ (ppm): 175.4 (C=Se), 158.6 (C=O), 140.5 (C-8a), 139.2 (C-2′), 136.81 (C-1′), 136.79(C-7), 131.4 (C-3′), 129.4 (k, *^3^J*(^13^C,^19^F) = 3.2, C-6′), 129.2 (k, *^2^J*(^13^C,^19^F) = 32.8, C-5′), 127.8 (k, *^1^J*(^13^C-^19^F) = 272.6, CF_3_); 128.1 (C-5), 127.6 (d, *^3^J*(^13^C,^19^F) = 2.3, C-4′), 125.9 (C-6), 116.8 (C-8), 116.7 (C-4a). ^19^F NMR (282.38 MHz, DMSO-*d*6), δ (ppm): −61.02 (s, 3F, CF_3_). ^77^Se NMR (76.35 MHz, DMSO-*d*6), δ (ppm): 451.0 (s).

**Synthesis of compounds 4a**–**g**. To a solution (10 mmol) of the corresponding selon **3a**–**g** in 100 mL of absolute ethanol, 1.7 mL of 30% hydrogen peroxide was added (15 mmol) and refluxed for 1 h, then cooled to 20 °C. The solid precipitated from the solution was separated by filtration, washed with ethanol (2 × 50 mL), and dried at 40 °C.


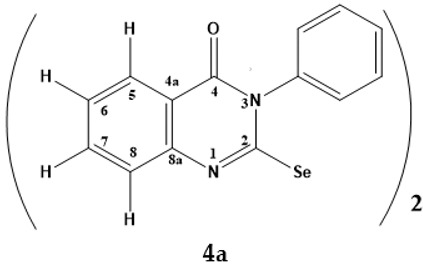


2,2′-diselane-1,2-diylbis(3-phenylquinazolin-4(3*H*)-one). Light brown solid (68%), mp 290 °C. Anal. Calcd. for C_28_H_18_N_4_O_2_Se_2_: C 56.01%, H 3.02%, N 9.33%. Found: C 56.09; H 3.06; N 9.37. ESI^+^-MS, *m*/*z*: calcd for [C_28_H_18_N_4_O_2_Se_2_+ H]^+^ 602.9838, found 602.9827 [C_28_H_18_N_4_O_2_Se_2_+ H]^+^. IR (KBr, selected bands, sm^−1^): 1685, 1544, 1468, 1261, 1201, 952, 770, 696. ^1^H NMR (500 MHz, DMSO-*d*_6_), δ (ppm): 8.05 (d, *J* = 8.0, 1H, H-5), 7.82 (t, 1H, H-7), 7.66 (m, 5H, Ph), 7.47 (t, 1H, H-6), 7.40 (d, J = 8.0, 1H, H-8).


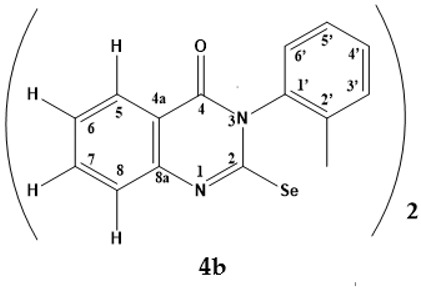


2,2′-diselane-1,2-diylbis [3-(2-methylphenyl)-quinazolin-4(3*H*)-one]. Orange solid (79%), mp 230 °C. Anal. Calcd. for C_30_H_22_N_4_O_2_Se_2_: C 57.33%, H 3.53%, N 8.91%. Found: C 57.22; H 3.56; N 8.79. ESI^+^-MS, *m*/*z*: calcd for [C_30_H_22_N_4_O_2_Se_2_+ H]^+^ 631.0152, found 631.0132 [C_30_H_22_N_4_O_2_Se_2_+ H]^+^. IR (KBr, selected bands, sm^−1^): 1684, 1610, 1575, 1538, 1467, 1254, 1199, 763, 695. ^1^H NMR (500 MHz, DMSO-*d*_6_), δ (ppm): 8.10 (d, 1H, H-5), 7.83 (m, 1H, H-7), 7.42–7.65 (m, 6H, H-6, H-8, 4H Ar), 2.24, 2.34 (s, 3H, CH_3_). ^13^C NMR (125.72 MHz, DMSO-*d*_6_), δ (ppm): 160.30, 160.25 (C=O), 151.00, 151.62 (C-Se), 148.4 (C-8a), 137.4 137.5 (C-2′), 135.8, 135.9 (C-1′), 135.76, 135.73, (C-7), 131.9 (C-3′), 132.0 (CH Ar), 130.2 (CH Ar), 128.2 (CH Ar), 127.5 (C-6), 127.3 (C-5), 126.4, 126.6 (C-8), 120.1 (C-4a), 17.6, 17.7 (CH_3_). ^77^Se NMR (76.35 MHz, DMSO-*d*6), δ (ppm): 522.3, 513.9 (s, 2Se).


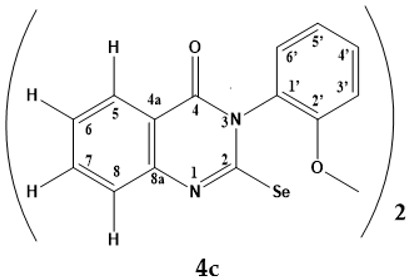


2,2′-diselane-1,2-diylbis [3-(2-methoxyphenyl)-quinazolin-4(3*H*)-one]. Orange solid (91%), mp 277 °C. Anal. Calcd. for C_30_H_22_N_4_O_4_Se_2_: C 54.56%, H 3.36%, N 8.48%. Found: 54.48; H 3.43; N 8.43. ESI^+^-MS, *m*/*z*: calcd for [C_30_H_22_N_4_O_4_Se_2_+H]^+^ 663.0050, found 663.0043 [C_30_H_22_N_4_O_4_Se_2_+H]^+^. IR (KBr, selected bands, sm^−1^): 1680, 1541, 1498, 1465, 1263, 1021, 764, 695, 640. ^1^H NMR (500 MHz, DMSO-*d*_6_), δ (ppm): 8.07 (dd, *J* = 12.5, *J* = 2.5, 1H, H-5), 7.81 (m, 1H, H-7), 7.70 (m, 1H, H-4′), 7.61 (m, 1H, H-6′), 7.51 (t, 1H, H-6), 7.41 (d, 1H, H-8), 7.38 (d, 1H, H-3′), 7.23 (t, 1H, H-5′), 3.84, 3.85 (3H, c, OCH_3_).


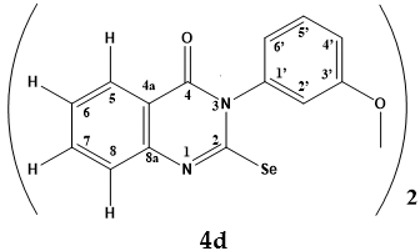


2,2′-diselane-1,2-diylbis [3-(3-methoxyphenyl)-quinazolin-4(3*H*)-one]. Orange solid (83%), mp 271 °C. Anal. Calcd. for C_30_H_22_N_4_O_4_Se_2_: C 54.56%, H 3.36%, N 8.48%. Found: C 54.44; H 3.32; N 8.39. ESI^+^-MS, *m*/*z*: calcd for [C_30_H_22_N_4_O_4_Se_2_+H]^+^ 663.0050, found 663.0043 [C_30_H_22_N_4_O_4_Se_2_+H]^+^. IR (KBr, selected bands, sm^−1^): 3067, 1695, 1603, 1539, 1464, 1268, 1236, 1199, 1032, 905, 839, 768, 690. ^1^H NMR (500 MHz, DMSO-*d*_6_), δ (ppm): 8.08 (d, 1H, H-5), 7.80 (t, 1H, H-7), 7.60 (t, 1H, H-5′), 7.50 (t, 1H, H-6); 7.44 (d, 1H, H-8); 7.20–7.33 (m, 3H, 3H Ar), 3.85 (s, 3H, OCH_3_). ^13^C NMR (125.72 MHz, DMSO-*d*_6_), δ (ppm): 160.61 (C=O), 160.55 (C-O), 153.3 (C-Se), 147.9 (C-8a), 138.4 (C-1′), 135.56 (C-7), 131.2 (C-5′), 127.2 (C-5, C-6), 126.5 (C-8), 121.7 (C-6′), 120.5 (C-4a), 117.1 (C-4′), 115.4 (C-2′), 56.1 (OCH_3_). ^77^Se NMR (76.35 MHz, DMSO-*d*6), δ (ppm): 534.7 (s, 2Se).


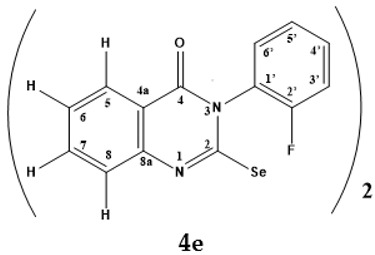


2,2′-diselane-1,2-diylbis [3-(2-fluorophenyl)-quinazolin-4(3*H*)-one]. Orange—red solid (66%), mp 250 °C. Anal. Calcd. for C_28_H_16_F_2_N_4_O_2_Se_2_: C 52.85%, H 2.53%, N 8.80%. Found: C 52.79; H 2.46; N 8.69. ESI^+^-MS, *m*/*z*: calcd for [C_28_H_16_F_2_N_4_O_2_Se_2_+H]^+^ 638.9650, found 638.9636 [C_28_H_16_F_2_N_4_O_2_Se_2_+H]^+^. IR (KBr, selected bands, sm^−1^): 1700, 1680, 1544, 1498, 1464, 1258, 1199, 1114, 951, 879, 771, 691, 638. ^1^H NMR (500 MHz, DMSO-*d*_6_), δ (ppm): 8.10 (d, *J* = 5.0, 1H, H-5), 7.70-7.82 (m, 2H, H-7, 1H Ar), 7.89 (m, 1H, 1H Ar), 7.65 (t, 1H, 1H Ar), 7.50–7.57 (m, 2H, H-6, 1H Ar), 7.48 (d, *J* = 8.0, 1H, H-8). ^13^C NMR (125.72 MHz, DMSO-*d*_6_), δ (ppm): 160.2 (C=O), 158.1 (d, ^1^*J(^13^C-^19^F)* = 252.5, C-2′-F,); 151.2 (C-Se), 147.8 (C-8a), 136.0 (C-7), 132.0 (C-5′), 134.36 (d, ^3^*J(^13^C-^19^F)* = 7.6, C-6′), 127.7 (C-6), 127.3 (C-5), 126.6 (C-8), 126.4 (d, ^2^*J(^13^C-^19^F)* = 3.8, C-3′), 124.3 (d, ^2^*J(^13^C-^19^F)* = 12.5, C-1′), 119.8 (C-4a), 117.6 (d, ^3^*J(^13^C-^19^F)* = 18.8, C-4′). ^19^F NMR (282.38 MHz, DMSO-*d*6), δ (ppm): -120.27 (s, 1F). ^77^Se NMR (76.35 MHz, DMSO-*d*6), δ (ppm): 535.6–533.8 (m, 2Se).


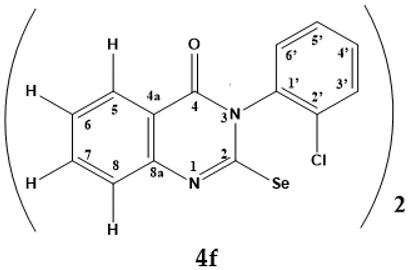


2,2′-diselane-1,2-diylbis [3-(2-chlorophenyl)-quinazolin-4(3*H*)-one]. Cherry-red solid (87%), mp 255 °C. ESI^+^-MS, *m*/*z*: calcd for [C_28_H_16_Cl_2_N_4_O_2_Se_2_+H]^+^ 670.9051, found 670.9042 [C_28_H_16_Cl_2_N_4_O_2_Se_2_+H]^+^. IR (KBr, selected bands, sm^−1^): 3076, 1683, 1542, 1466, 1335, 1262, 1246, 1198, 947, 979, 765, 695, 637. ^1^H NMR (500 MHz, DMSO-*d*_6_), δ (ppm): 8.10 (dd, *J* = 10.0, *J* = 1.5, 1H, H-5), 7.80–7.95 (m, 2H, H-7, H Ar), 7.77 (m, 1H, H Ar), 7.70 (m, 1H, H Ar), 7.53 (m, 1H, H-6), 7.49 (m,1H, H-6). ^13^C NMR (125.72 MHz, DMSO-*d*_6_), δ (ppm): 160.0 (C=O), 150.9 (C-Se), 147.9 (C-8a), 136.0 (C-7), 134.2 (C, Ar), 133.6 (CH, Ar), 132.3 (CH, Ar), 131.2 (CH, Ar), 129.5 (CH, Ar), 127.6 (C-6), 127.3 (C-5), 126.7 (C-8), 120.0 (C-4a). ^77^Se NMR (76.35 MHz, DMSO-*d*6), δ (ppm): 532.5, 529.6, 528.5 (s, 2Se).


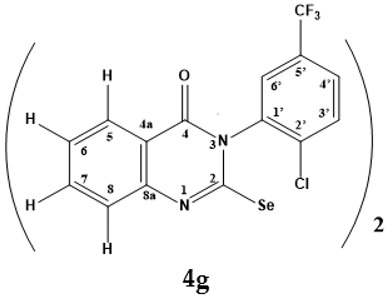


2,2′-diselane-1,2-diylbis [3-[2-chloro-5-(trifluoromethyl)phenyl]quinazolin-4(3*H*)-one]. Orange solid (59%), mp 210 °C. Anal. Calcd. for C_30_H_14_Cl_2_F_6_N_4_O_2_Se_2_: C 44.74%, H 1.75%, N 6.96%. Found: C 44.66; H 1.66; N 6.87. ESI^+^-MS, *m*/*z*: calcd for [C_30_H_14_Cl_2_F_6_N_4_O_2_Se_2_+H]^+^ 806.8799, found 806.8784 [C_30_H_14_Cl_2_F_6_N_4_O_2_Se_2_+ H]^+^. IR (KBr, selected bands, sm^−1^): 1715, 1704, 1609, 1544, 1467, 1338, 1130, 1074, 959, 886, 847, 770, 694, 614. ^1^H NMR (500 MHz, DMSO-*d*_6_), δ (ppm): 8.53 (2 bs, 1H, H-6′), 8.16 (bs, 2H, H-3′, H-4′), 8.12 (dd, *J* = 8.0, *J* = 1.2, 1H, H-5); 7.87 (m, 1H, H-7); 7.48–7.58 (m, 2H, H-6, H-8). ^13^C NMR (125.72 MHz, DMSO-*d*_6_), δ (ppm): 160.1 (C=O), 149.8, 149.5 (C-Se), 147.8 (C-8a), 138.2 (C-1′), 136.1 (C-7), 135.2 (C-2′), 132.5 (C-3′), 130.37 (C-6′), 130.02 (k, ^2^*J*(^13^C,^19^F) = 33.2, C-5′), 129.8 (C-4′), 127.8 (C-5), 127.3 (C-6), 126.8 (C-8), 125.6 (к, ^1^*J(^13^C-^19^F)* = 271.3, CF_3_), 120.0 (C-4a). ^19^F NMR (282.38 MHz, DMSO-*d*6), δ (ppm): −61.09 (s, 3F, CF_3_). ^77^Se NMR (76.35 MHz, DMSO-*d*6), δ (ppm): 536.8, 533.5 (s, 2Se).

### 3.2. Computational Details

The DFT calculations based on the experimental X-ray geometries of **3f**, **4b**, **4c**, **5**, **6**, **7**, and **8** were carried out using the dispersion-corrected hybrid functional ωB97XD [66] with the help of Gaussian-09 [67] program package. The 6-311++G** basis sets were used for all atoms, except Te (for which quasi-relativistic MWB46 pseudopotentials [68], which described 46 core electrons, and the appropriate contracted basis sets were utilized). The topological analysis of the electron density distribution with the help of the quantum theory of atoms-in-molecules (QTAIM) method, electron localization function (ELF), reduced density gradient (RDG), and noncovalent interactions (NCI) analyses was performed by using the Multiwfn program (version 3.7) [69]. The VMD program [70] was used for the visualization of noncovalent interactions (NCI analysis). The Cartesian atomic coordinates for model supramolecular associates are presented in Table S1, Supplementary Materials.

## 4. Conclusions

In summary, we reported a convenient synthesis of series novel 2-selenoxo-1,2,3,4-tetrahydro-4-quinazolinone via a reaction between methyl anthranilate and isoselenocyanates. These compounds were found to undergo facile oxidation to furnish corresponding diselenides in high yields. The structures and purity of all compounds were unambiguously established using the ^1^H, ^77^Se, and ^13^C NMR spectroscopies; high-resolution electrospray ionization mass spectrometry; IR; elemental analyses; and X-ray diffraction analysis for several of them. X-Ray single crystal analysis was performed for **3f**, **4b**, **4c**, **5**, **6**, **7**, and **8**, which revealed that selone **3f** featured the formation of unsymmetrical supramolecular dimers via type II Se···Se ChB, while **3b** and **3f** did not exhibit ChB interactions, arguably due to dominance of other weak interactions in the crystal. For compounds **4b** and **4c,** a pair of intramolecular Se···N ChB were found in the solid state. Such intramolecular ChB interactions are scarce—CCDC contained only four structures featuring such contacts. The existence of all the above-mentioned ChB was additionally confirmed by DFT calculations followed by the topological analysis of the electron density distribution.

## Figures and Tables

**Table 1:** Values of the density of all electrons—⍴(**r**), Laplacian of electron density—∇^2^⍴(**r**) and appropriate λ_2_ eigenvalues, energy density—H_b_, potential energy density—V(**r**), and Lagrangian kinetic energy—G(**r**) (a.u.) at the bond critical points (3, −1), corresponding to contacts Se···Se, Se···N, and Te···N in the X-ray structures **3f**, **4b**, **4c**, **5**, **6**, **7**, and **8**, and approximately estimated strength for these interactions E_int_ (kcal/mol) [55].

Contact *	⍴(r)	∇^2^⍴(r)	λ_2_	H_b_	V(r)	G(r)	E_int_ ≈ −V(r)/2
**3f**							
Se···Se 3.717 Å	0.007	0.020	−0.007	0.001	−0.003	0.004	0.9
4b							
Se–Se 2.360 Å	0.102	−0.052	−0.102	−0.043	−0.074	0.031	23.2
Se···N 2.899 Å	0.017	0.061	−0.017	0.002	−0.012	0.014	3.8
**4c**							
Se–Se 2.357 Å	0.102	−0.052	−0.102	−0.044	−0.075	0.031	23.5
Se···N 2.870 Å	0.018	0.063	−0.018	0.001	−0.013	0.014	4.1
**5**							
Se–Se 2.359 Å	0.102	−0.054	−0.102	−0.044	−0.074	0.030	23.2
Se···N 2.792 Å	0.021	0.070	−0.021	0.001	−0.015	0.016	4.7
**6**							
Se–Se 2.433 Å	0.095	−0.048	−0.095	−0.039	−0.066	0.027	20.7
Se···N 2.733 Å	0.024	0.080	−0.024	0.001	−0.018	0.019	5.6
Se···N 2.479 Å	0.042	0.115	−0.042	−0.003	−0.035	0.032	11.0
**7**							
Se–Se 2.343 Å	0.104	−0.052	−0.104	−0.045	−0.077	0.032	24.2
Se···N 2.925 Å	0.017	0.059	−0.017	0.002	−0.011	0.013	3.5
**8**							
Te–Te 2.723 Å	0.072	0.138	−0.072	−0.018	−0.031	0.013	9.7
Te···N 3.082 Å	0.016	0.056	−0.016	0.001	−0.010	0.011	3.1

* The Bondi’s (shortest) Van der Waals radii for Te, Se, and N atoms are 2.00, 1.90, and 1.55 Å, respectively [56].

## Data Availability

Not applicable.

## Supplementary Materials

The following supporting information can be downloaded at: https://www.mdpi.com/article/10.3390/molecules27185799/s1, Figure S1: Crystal packing of **3b** demonstrating the H-bonded chains of the crystallographically independent molecules **A**. Figure S2: Crystal packing of 3f demonstrating the ribbons towards the crystallographic c axis. Within the ribbons, the molecules are bound to each other by the strong N─H···O hydrogen bonds and weak nonvalent Se···Se interactions (Se1···Se2 [1−*x*, 2−*y*, 1−*z*] 3.7173(4) Å). Figure S3: The two projections of crystal packing of **3g** demonstrating the two-tier layer parallel to (010). Within the layer, the molecules are bound to each other by the N─H···Se hydrogen bonds as well as the nonvalent Se···O (Se2···O2 [1−*x*, −0.5+*y*, 1.5−*z*] 3.3702(16) Å) and Cl···F (Cl2···F1 [1−x, −0.5+y, 1.5−z] 3.0607(17) Å) interactions. Scheme S1. Plausible mechanism for the formation of **3a–g**. Table S1: Cartesian atomic coordinates for model supramolecular associates. Crystal structure determinations, Table S2: Crystal data and structure refinement for all compounds studied. References [71,72,73,74] are cited in Supplementary Materials.

## References

[1] Khan I., Ibrar A., Ahmed W., Saeed A. (2015). Synthetic approaches, functionalization and therapeutic potential of quinazoline and quinazolinone skeletons: The advances continue. Eur. J. Med. Chem..

[2] Asif M. (2014). Chemical Characteristics, Synthetic Methods, and Biological Potential of Quinazoline and Quinazolinone Derivatives. Int. J. Med. Chem..

[3] Jafari E., Khajouei M., Hassanzadeh F., Hakimelahi G., Khodarahmi G. (2016). Quinazolinone and quinazoline derivatives: Recent structures with potent antimicrobial and cytotoxic activities. Res. Pharm. Sci..

[4] Hameed A., Al-Rashida M., Uroos M., Ali S.A., Arshia, Ishtiaq M., Khan K.M. (2018). Quinazoline and quinazolinone as important medicinal scaffolds: A comparative patent review (2011–2016). Expert Opin. Ther. Pat..

[5] Moreno E., Plano D., Lamberto I., Font M., Encío I., Palop J.A., Sanmartín C. (2012). Sulfur and selenium derivatives of quinazoline and pyrido [2,3-d]pyrimidine: Synthesis and study of their potential cytotoxic activity in vitro. Eur. J. Med. Chem..

[6] Moreno E., Doughty-Shenton D., Plano D., Font M., Encío I., Palop J.A., Sanmartín C. (2014). A dihydroselenoquinazoline inhibits S6 ribosomal protein signalling, induces apoptosis and inhibits autophagy in MCF-7 cells. Eur. J. Pharm. Sci..

[7] Kasibhatla S., Baichwal V., Cai S.X., Roth B., Skvortsova I., Skvortsov S., Lukas P., English N.M., Sirisoma N., Drewe J. (2007). MPC-6827: A Small-Molecule Inhibitor of Microtubule Formation That Is Not a Substrate for Multidrug Resistance Pumps. Cancer Res..

[8] Sirisoma N., Pervin A., Zhang H., Jiang S., Willardsen J.A., Anderson M.B., Mather G., Pleiman C.M., Kasibhatla S., Tseng B. (2009). Discovery of N-(4-Methoxyphenyl)-N,2-dimethylquinazolin-4-amine, a Potent Apoptosis Inducer and Efficacious Anticancer Agent with High Blood Brain Barrier Penetration. J. Med. Chem..

[9] Wu X., Li M., Qu Y., Tang W., Zheng Y., Lian J., Ji M., Xu L. (2010). Design and synthesis of novel Gefitinib analogues with improved anti-tumor activity. Bioorg. Med. Chem..

[10] Hanusek J., Hejtmánková L., Kubicová L., Sedlák M. (2001). Synthesis of Substituted 2-Benzoylaminothiobenzamides and Their Ring Closure to Substituted 2-Phenylquinazoline-4-thiones. Molecules.

[11] Kubicová L., Šustr M., Kráľová K., Chobot V., Vytlačilová J., Jahodář L., Vuorela P., Macháček M., Kaustová J. (2003). Synthesis and Biological Evaluation of Quinazoline-4-thiones. Molecules.

[12] Niewiadomy A., Matysiak J., Karpińska M.M. (2011). Synthesis and Anticancer Activity of New 2-Aryl-4H-3,1-benzothiazines. Arch. Pharm. Weinh..

[13] Sancineto L., Iraci N., Massari S., Attanasio V., Corazza G., Barreca M.L., Sabatini S., Manfroni G., Avanzi N.R., Cecchetti V. (2013). Computer-Aided Design, Synthesis and Validation of 2-Phenylquinazolinone Fragments as CDK9 Inhibitors with Anti-HIV-1 Tat-Mediated Transcription Activity. ChemMedChem.

[14] Häcker H.-G., Grundmann F., Lohr F., Ottersbach P.A., Zhou J., Schnakenburg G., Gütschow M. (2009). 2-Amino- and 2-Alkylthio-4H-3,1-benzothiazin-4-ones: Synthesis, Interconversion and Enzyme Inhibitory Activities. Molecules.

[15] Komar M., Kraljević T.G., Jerković I., Molnar M. (2022). Application of Deep Eutectic Solvents in the Synthesis of Substituted 2-Mercaptoquinazolin-4(3H)-Ones: A Comparison of Selected Green Chemistry Methods. Molecules.

[16] Yadav S., Deka R., Singh H.B. (2018). Recent Developments in the Chemistry of NHC-based Selones: Syntheses, Applications and Reactivity. Chem. Lett..

[17] Mammadova G.Z., Matsulevich Z.V., Osmanov V.K., Borisov A.V., Khrustalev V.N. (2011). 1,3-Benzothia{\-}zole-2(3{\it H})-selone. Acta Crystallogr. Sect. E.

[18] Mammadova G.Z., Matsulevich Z.V., Osmanov V.K., Borisov A.V., Khrustalev V.N. (2012). 1-Methyl-2,3-dihydro-1{\it H}-benzimidazole-2-selone. Acta Crystallogr. Sect. E.

[19] Alcolea V., Plano D., Encío I., Palop J.A., Sharma A.K., Sanmartín C. (2016). Chalcogen containing heterocyclic scaffolds: New hybrids with antitumoral activity. Eur. J. Med. Chem..

[20] Yun L.M., Shakhidoyatov K.M. (1986). 2-Selenoxoquinazolones-4, a new kind of quinazolone. Chem. Heterocycl. Compd..

[21] Šibor J., Žůrek D., Humpa O., Pazdera P. (2000). Acid-Base Initiated Cyclization and Retrocyclization Reactions of Ethyl 2-(3-Acylselenoureido)benzoates, -thiophene-3-carboxylates and the Corresponding 2-(3-Acylisoselenoureido) Derivatives. Molecules.

[22] Atanassov P.K., Linden A., Heimgartner H. (2004). Synthesis of 4-(Phenylamino)quinazoline-2(1H)-selones and Diselenides from Isoselenocyanates: Dimroth Rearrangement of an Intermediate. Helv. Chim. Acta.

[23] Desiraju G.R. (1995). Supramolecular Synthons in Crystal Engineering—A New Organic Synthesis. Angew. Chem. Int. Ed. Engl..

[24] Hobza P., Havlas Z. (2000). Blue-Shifting Hydrogen Bonds. Chem. Rev..

[25] Eliseeva A.A., Ivanov D.M., Novikov A.S., Kukushkin V.Y. (2019). Recognition of the π-hole donor ability of iodopentafluorobenzene – a conventional σ-hole donor for crystal engineering involving halogen bonding. CrystEngComm.

[26] Scheiner S. (2013). The Pnicogen Bond: Its Relation to Hydrogen, Halogen, and Other Noncovalent Bonds. Acc. Chem. Res..

[27] Murray J.S., Lane P., Clark T., Riley K.E., Politzer P. (2012). σ-Holes, π-holes and electrostatically-driven interactions. J. Mol. Model..

[28] Li H., Lu Y., Liu Y., Zhu X., Liu H., Zhu W. (2012). Interplay between halogen bonds and π–π stacking interactions: CSD search and theoretical study. Phys. Chem. Chem. Phys..

[29] Nelyubina Y.V., Antipin M.Y., Lyssenko K.A. (2011). Extremely short halogen bond: The nature and energy of iodine–oxygen interactions in crystalline iodic acid. Mendeleev Commun..

[30] Metrangolo P., Neukirch H., Pilati T., Resnati G. (2005). Halogen Bonding Based Recognition Processes:  A World Parallel to Hydrogen Bonding. Acc. Chem. Res..

[31] Li Q., Li R., Zhou Z., Li W., Cheng J. (2012). S···X halogen bonds and H···X hydrogen bonds in H2CS–XY (XY = FF, ClF, ClCl, BrF, BrCl, and BrBr) complexes: Cooperativity and solvent effect. J. Chem. Phys..

[32] Tsirelson V.G., Zhou P.F., Tang T.-H., Bader R.F.W. (1995). Topological definition of crystal structure: Determination of the bonded interactions in solid molecular chlorine. Acta Crystallogr. Sect. A.

[33] Grabowski S.J. (2017). Lewis Acid Properties of Tetrel Tetrafluorides—The Coincidence of the σ-Hole Concept with the QTAIM Approach. Crystals.

[34] Khrustalev V.N., Grishina M.M., Matsulevich Z.V., Lukiyanova J.M., Borisova G.N., Osmanov V.K., Novikov A.S., Kirichuk A.A., Borisov A.V., Solari E. (2021). Novel cationic 1,2,4-selenadiazoles: Synthesis via addition of 2-pyridylselenyl halides to unactivated nitriles, structures and four-center Se⋯N contacts. Dalt. Trans..

[35] Grudova M.V., Khrustalev V.N., Kubasov A.S., Strashnov P.V., Matsulevich Z.V., Lukiyanova J.M., Borisova G.N., Kritchenkov A.S., Grishina M.M., Artemjev A.A. (2022). Adducts of 2-Pyridylselenenyl Halides and Nitriles as Novel Supramolecular Building Blocks: Four-Center Se···N Chalcogen Bonding versus Other Weak Interactions. Cryst. Growth Des..

[36] Buslov I.V., Novikov A.S., Khrustalev V.N., Grudova M.V., Kubasov A.S., Matsulevich Z.V., Borisov A.V., Lukiyanova J.M., Grishina M.M., Kirichuk A.A. (2021). 2-Pyridylselenenyl versus 2-Pyridyltellurenyl Halides: Symmetrical Chalcogen Bonding in the Solid State and Reactivity towards Nitriles. Symmetry.

[37] Artemjev A.A., Novikov A.P., Burkin G.M., Sapronov A.A., Kubasov A.S., Nenajdenko V.G., Khrustalev V.N., Borisov A.V., Kirichuk A.A., Kritchenkov A.S. (2022). Towards Anion Recognition and Precipitation with Water-Soluble 1,2,4-Selenodiazolium Salts: Combined Structural and Theoretical Study. Int. J. Mol. Sci..

[38] Grudova M.V., Novikov A.S., Kubasov A.S., Khrustalev V.N., Kirichuk A.A., Nenajdenko V.G., Tskhovrebov A.G. (2022). Aurophilic Interactions in Cationic Three-Coordinate Gold(I) Bipyridyl/Isocyanide Complex. Crystals.

[39] Khrustalev V.N., Savchenko A.O., Zhukova A.I., Chernikova N.Y., Kurykin M.A., Novikov A.S., Tskhovrebov A.G. (2021). Attractive fluorine···fluorine interactions between perfluorinated alkyl chains: A case of perfluorinated Cu(II) diiminate Cu[C2F5-C(NH)-CF=C(NH)-CF3]2. Z. Fur Krist.-Cryst. Mater..

[40] Tskhovrebov A.G., Novikov A.S., Kritchenkov A.S., Khrustalev V.N., Haukka M. (2020). Attractive halogen···halogen interactions in crystal structure of trans-dibromogold(III) complex. Z. Fur Krist.-Cryst. Mater..

[41] Shikhaliyev N.G., Maharramov A.M., Suleymanova G.T., Babazade A.A., Nenajdenko V.G., Khrustalev V.N., Novikov A.S., Tskhovrebov A.G. (2021). Arylhydrazones of α-keto esters via methanolysis of dichlorodiazabutadienes: Synthesis and structural study. Mendeleev Commun..

[42] Tskhovrebov A.G., Novikov A.S., Tupertsev B.S., Nazarov A.A., Antonets A.A., Astafiev A.A., Kritchenkov A.S., Kubasov A.S., Nenajdenko V.G., Khrustalev V.N. (2021). Azoimidazole Gold(III) Complexes: Synthesis, Structural Characterization and Self-Assembly in the Solid State. Inorg. Chim. Acta.

[43] Shikhaliyev N.G., Maharramov A.M., Bagirova K.N., Suleymanova G.T., Tsyrenova B.D., Nenajdenko V.G., Novikov A.S., Khrustalev V.N., Tskhovrebov A.G. (2021). Supramolecular organic frameworks derived from bromoaryl-substituted dichlorodiazabutadienes via Cl···Br halogen bonding. Mendeleev Commun..

[44] Tsyrenova B., Nenajdenko V. (2020). Synthesis and spectral study of a new family of 2,5-diaryltriazoles having restricted rotation of the 5-aryl substituent. Molecules.

[45] Repina O.V., Novikov A.S., Khoroshilova O.V., Kritchenkov A.S., Vasin A.A., Tskhovrebov A.G. (2020). Lasagna-like supramolecular polymers derived from the PdII osazone complexes via C(sp2)–H⋯Hal hydrogen bonding. Inorg. Chim. Acta.

[46] Mikhaylov V.N., Sorokoumov V.N., Novikov A.S., Melnik M.V., Tskhovrebov A.G., Balova I.A. (2020). Intramolecular hydrogen bonding stabilizes trans-configuration in a mixed carbene/isocyanide PdII complexes. J. Organomet. Chem..

[47] Nenajdenko V.G., Shikhaliyev N.G., Maharramov A.M., Atakishiyeva G.T., Niyazova A.A., Mammadova N.A., Novikov A.S., Buslov I.V., Khrustalev V.N., Tskhovrebov A.G. (2022). Structural Organization of Dibromodiazadienes in the Crystal and Identification of Br···O Halogen Bonding Involving the Nitro Group. Molecules.

[48] Liu Y., Varava P., Fabrizio A., Eymann L.Y.M., Tskhovrebov A.G., Planes O.M., Solari E., Fadaei-Tirani F., Scopelliti R., Sienkiewicz A. (2019). Synthesis of aminyl biradicals by base-induced Csp3–Csp3 coupling of cationic azo dyes. Chem. Sci..

[49] Palmer J.H., Parkin G. (2013). 2-Seleno-1-alkylbenzimidazoles and their diselenides: Synthesis and structural characterization of a 2-seleno-1-methylbenzimidazole complex of mercury. Polyhedron.

[50] Antoniadis C.D., Blake A.J., Hadjikakou S.K., Hadjiliadis N., Hubberstey P., Schröder M., Wilson C. (2006). Structural characterization of selenium and selenium-diiodine analogues of the antithyroid drug 6-{\it n}-propyl-2-thiouracil and its alkyl derivatives. Acta Crystallogr. Sect. B.

[51] Bhasin K.K., Arora E., Grover A.S., Jyoti, Singh H., Mehta S.K., Bhasin A.K.K., Jacob C. (2013). Synthesis and characterization of new 2-pyrimidyl chalcogen (S, Se, Te) compounds: X-ray crystal structure of bis(4,6-dimethyl-2-pyrimidyl)diselenide and 4,6-dimethyl-2-(phenylselanyl)pyrimidine. J. Organomet. Chem..

[52] Borisov A.V., Matsulevich Z.V., Osmanov V.K., Borisova G.N., Chizhov A.O., Mammadova G.Z., Maharramov A.M., Aisin R.R., Khrustalev V.N. (2013). Diorganyl dichalcogenides with intramolecular coordination interactions: The synthesis and structure of bis(4,6-dimethylpyrimidin-2-yl) diselenide. Russ. Chem. Bull..

[53] Bader R.F.W. (1991). A Quantum Theory of Molecular Structure and Its Applications. Chem. Rev..

[54] Johnson E.R., Keinan S., Mori-Sánchez P., Contreras-García J., Cohen A.J., Yang W. (2010). Revealing noncovalent interactions. J. Am. Chem. Soc..

[55] Espinosa E., Molins E., Lecomte C. (1998). Hydrogen bond strengths revealed by topological analyses of experimentally observed electron densities. Chem. Phys. Lett..

[56] Bondi A. (1966). Van der Waals volumes and radii of metals in covalent compounds. J. Phys. Chem..

[57] Grudova M.V., Kubasov A.S., Khrustalev V.N., Novikov A.S., Kritchenkov A.S., Nenajdenko V.G., Borisov A.V., Tskhovrebov A.G. (2022). Exploring Supramolecular Assembly Space of Cationic 1,2,4-Selenodiazoles: Effect of the Substituent at the Carbon Atom and Anions. Molecules.

[58] Novikov A.S., Gushchin A.L. (2021). Trinuclear molybdenum clusters with sulfide bridges as potential anionic receptors via chalcogen bonding. CrystEngComm.

[59] Mikherdov A.S., Novikov A.S., Kinzhalov M.A., Zolotarev A.A., Boyarskiy V.P. (2018). Intra-/Intermolecular Bifurcated Chalcogen Bonding in Crystal Structure of Thiazole/Thiadiazole Derived Binuclear (Diaminocarbene)PdII Complexes. Crystals.

[60] Mikherdov A.S., Novikov A.S., Kinzhalov M.A., Boyarskiy V.P., Starova G.L., Ivanov A.Y., Kukushkin V.Y. (2018). Halides Held by Bifurcated Chalcogen–Hydrogen Bonds. Effect of μ(S,N–H)Cl Contacts on Dimerization of Cl(carbene)PdII Species. Inorg. Chem..

[61] Pairan N.F., Kasim N.A.M., Yamin B.M., Shah N.A.A. (2017). Crystal structure of (E)-N,N-diethyl-2-(5-nitrothiazol-2-yl)-1-phenylethen-1-amine, C15H17N3O2S. Z. Für Krist.-New Cryst. Struct..

[62] Mikherdov A.S., Kinzhalov M.A., Novikov A.S., Boyarskiy V.P., Boyarskaya I.A., Dar’In D.V., Starova G.L., Kukushkin V.Y. (2016). Difference in Energy between Two Distinct Types of Chalcogen Bonds Drives Regioisomerization of Binuclear (Diaminocarbene)PdII Complexes. J. Am. Chem. Soc..

[63] Contreras-García J., Johnson E.R., Keinan S., Chaudret R., Piquemal J.-P., Beratan D.N., Yang W. (2011). NCIPLOT: A Program for Plotting Noncovalent Interaction Regions. J. Chem. Theory Comput..

[64] Espinosa E., Alkorta I., Elguero J., Molins E. (2002). From weak to strong interactions: A comprehensive analysis of the topological and energetic properties of the electron density distribution involving X-H⋯F-Y systems. J. Chem. Phys..

[65] Zakrzewski J., Huras B., Kiełczewska A. (2016). Synthesis of Isoselenocyanates. Syntheses.

[66] Chai J.-D., Head-Gordon M. (2008). Long-range corrected hybrid density functionals with damped atom–atom dispersion corrections. Phys. Chem. Chem. Phys..

[67] Frisch M.J., Trucks G.W., Schlegel H.B., Scuseria G.E., Robb M.A., Cheeseman J.R., Scalmani G., Barone V., Mennucci B., Petersson G.A. (2010). Gaussian 09 C.01.

[68] Bergner A., Dolg M., Küchle W., Stoll H., Preuß H. (1993). Ab initio energy-adjusted pseudopotentials for elements of groups 13–17. Mol. Phys..

[69] Lu T., Chen F. (2012). Multiwfn: A multifunctional wavefunction analyzer. J. Comput. Chem..

[70] Humphrey W., Dalke A., Schulten K. (1996). VMD: Visual molecular dynamics. J. Mol. Graph..

[71] Rigaku (2021). CrysAlisPro Software System, v. 1.171.41.106a.

[72] Bruker (2014). SAINT, v. 8.34A.

[73] Krause L., Herbst-Irmer R., Sheldrick G.M., Stalke D. (2015). Comparison of silver and molybdenum microfocus X-ray sources for single-crystal structure determination. J. Appl. Cryst..

[74] Sheldrick G.M. (2015). Crystal structure refinement with SHELXL. Acta Cryst..

