# Supplementary material for: Facile Access to 2-Selenoxo-1,2,3,4-tetrahydro-4-quinazolinone Scaffolds and Corresponding Diselenides via Cyclization between Methyl Anthranilate and Isoselenocyanates: Synthesis and Structural Features
Source: Molecules. 2022 Sep 7;27(18):5799. doi: 10.3390/molecules27185799 (PMC9504104; doi:10.3390/molecules27185799)
Supplement: Supplementary file 1 [file molecules-27-05799-s001.zip › molecules-1894104-supplementary.pdf]

Table S1. Cartesian atomic coordinates for model supramolecular associates.

Atom	X	Y	Z
**3f**			
Se	6.548107	6.625851	3.304034
Cl	5.485219	3.310934	5.354962
O	3.532790	5.765028	7.306067
N	3.804115	6.886913	3.481704
H	3.860900	7.099757	2.636799
N	4.775701	6.193090	5.456164
C	4.931060	6.560985	4.131738
C	3.542089	6.095181	6.125032
C	2.367163	6.395536	5.335499
C	1.074559	6.251188	5.873783
H	0.963540	5.989761	6.779900
C	-0.029586	6.488572	5.083999
H	-0.903101	6.344624	5.427398
C	0.147679	6.946714	3.763088
H	-0.615420	7.137433	3.230882
C	1.413119	7.125869	3.224428
H	1.522498	7.454323	2.339179
C	2.522737	6.811520	4.011826
C	5.936220	5.809407	6.227867
C	6.331611	4.485362	6.280603
C	7.390856	4.090925	7.096755
H	7.667324	3.182332	7.129879
C	8.033859	5.057522	7.862683
H	8.748326	4.800894	8.433663

C	7.654870	6.383885	7.806180
H	8.118885	7.032511	8.323043
C	6.588814	6.774746	6.991283
H	6.313723	7.682849	6.958901
Se	5.442197	10.103849	2.598000
Cl	6.881341	14.188021	1.632430
O	4.208161	13.767593	-0.713819
N	3.390505	11.955580	2.756961
H	3.173111	11.484733	3.565956
N	4.750628	12.268210	0.906557
C	4.480225	11.556983	2.067627
C	3.906589	13.245218	0.348560
C	2.719280	13.580361	1.123402
C	1.810816	14.544223	0.657567
H	1.949305	14.968347	-0.181412
C	0.715157	14.870246	1.431405
H	0.097868	15.525305	1.128625
C	0.513241	14.233380	2.668189
H	-0.243224	14.465581	3.193691
C	1.392982	13.276246	3.132768
H	1.246831	12.848727	3.968470
C	2.507918	12.946585	2.347881
C	6.001773	12.054518	0.226765
C	7.069715	12.907742	0.473870
C	8.272699	12.744494	-0.205921
H	9.004875	13.322116	-0.026192
C	8.395513	11.735943	-1.143868

H	9.214368	11.622016	-1.611009
C	7.330250	10.894385	-1.403781
H	7.420883	10.203018	-2.049069
C	6.131388	11.052342	-0.727003
H	5.399875	10.476047	-0.913613
**4b**			
Se	3.832819	5.162968	4.835410
O	0.067786	1.799428	4.948483
N	4.002582	2.522508	5.742049
C	3.207926	3.410058	5.272283
N	1.855781	3.198876	5.011187
C	1.258428	1.940541	5.171545
C	2.160562	0.922584	5.647479
C	1.703198	-0.403874	5.809893
H	0.813481	-0.634520	5.569356
C	2.544422	-1.351761	6.314609
H	2.240404	-2.246123	6.414319
C	3.848502	-1.018930	6.685694
H	4.418506	-1.684592	7.051639
C	4.315967	0.272493	6.524308
H	5.202968	0.492434	6.786432
C	3.484082	1.252496	5.976418
C	1.030090	4.311232	4.595901
C	0.423144	5.060588	5.564216
C	-0.365042	6.162239	5.090338
H	-0.803217	6.732528	5.712239
C	-0.487539	6.391912	3.762245

H	-1.003703	7.136402	3.475451
C	0.097516	5.599735	2.818600
H	-0.030744	5.785615	1.895514
C	0.879640	4.525334	3.206132
H	1.296820	3.958937	2.567784
C	0.542975	4.791988	6.951930
H	1.490572	4.705374	7.186299
H	0.076669	3.956991	7.164712
H	0.145683	5.528692	7.461786
Se	6.034073	4.568932	5.443948
O	9.799106	7.932472	5.330875
N	5.864311	7.209392	4.537308
C	6.658966	6.321842	5.007075
N	8.011111	6.533024	5.268171
C	8.608464	7.791359	5.107813
C	7.706330	8.809316	4.631879
C	8.163694	10.135774	4.469465
H	9.053411	10.366420	4.710002
C	7.322470	11.083661	3.964748
H	7.626488	11.978023	3.865038
C	6.018390	10.750830	3.593663
H	5.448386	11.416492	3.227718
C	5.550925	9.459407	3.755049
H	4.663924	9.239466	3.492926
C	6.382810	8.479404	4.302939
C	8.836802	5.420668	5.683457
C	9.443748	4.671312	4.715141

C	10.231934	3.569661	5.189020
H	10.670109	2.999372	4.567119
C	10.354431	3.339988	6.517113
H	10.870595	2.595498	6.803907
C	9.769376	4.132165	7.460758
H	9.897636	3.946285	8.383844
C	8.987252	5.206567	7.073226
H	8.570072	5.772963	7.711574
C	9.323917	4.939912	3.327428
H	8.376320	5.026526	3.093059
H	9.790223	5.774909	3.114645
H	9.721209	4.203208	2.817572
**4c**			
Se	3.311409	-0.271315	4.822077
O	-0.513051	3.011327	5.252943
O	-0.138919	0.007621	6.672488
N	3.483852	2.329229	5.854322
N	1.322706	1.652848	5.123704
C	2.674020	1.458507	5.350914
C	0.703659	2.867477	5.423872
C	1.609946	3.903960	5.921026
C	1.142616	5.205280	6.131560
H	0.235498	5.421531	5.953335
C	2.012864	6.179841	6.601615
H	1.708641	7.068663	6.745446
C	3.342073	5.834029	6.862178
H	3.938370	6.501836	7.181107

C	3.809354	4.568909	6.670404
H	4.710220	4.354563	6.881981
C	2.950223	3.581964	6.158659
C	0.543939	0.587785	4.528579
C	-0.205040	-0.240068	5.345703
C	-0.963984	-1.254640	4.747451
H	-1.491601	-1.837662	5.280041
C	-0.936653	-1.396585	3.384188
H	-1.454812	-2.089161	2.990217
C	-0.182784	-0.570637	2.549345
H	-0.178569	-0.695434	1.607150
C	0.555916	0.437266	3.142386
H	1.072538	1.026004	2.604584
C	-0.876968	-0.880249	7.519838
H	-0.770632	-0.603980	8.453694
H	-0.540686	-1.793840	7.409359
H	-1.826189	-0.850716	7.275951
Se	5.468643	0.271315	5.600429
O	9.293103	-3.011327	5.169563
O	8.918972	-0.007621	3.750018
N	5.296200	-2.329229	4.568184
N	7.457346	-1.652848	5.298802
C	6.106032	-1.458507	5.071591
C	8.076393	-2.867476	4.998634
C	7.170106	-3.903960	4.501480
C	7.637436	-5.205280	4.290946
H	8.544554	-5.421531	4.469170

C	6.767188	-6.179841	3.820891
H	7.071411	-7.068663	3.677060
C	5.437979	-5.834029	3.560328
H	4.841682	-6.501836	3.241399
C	4.970698	-4.568909	3.752102
H	4.069833	-4.354563	3.540525
C	5.829829	-3.581964	4.263847
C	8.236114	-0.587785	5.893927
C	8.985092	0.240068	5.076803
C	9.744036	1.254640	5.675054
H	10.271653	1.837662	5.142464
C	9.716706	1.396585	7.038318
H	10.234864	2.089161	7.432289
C	8.962836	0.570637	7.873161
H	8.958621	0.695435	8.815355
C	8.224136	-0.437266	7.280120
H	7.707514	-1.026004	7.817922
C	9.657020	0.880249	2.902668
H	9.550684	0.603980	1.968811
H	9.320738	1.793840	3.013146
H	10.606241	0.850716	3.146555
**5**			
Se	-0.075110	0.755202	0.903016
N	1.417486	-1.528920	1.419988
N	1.159671	-0.377134	3.350994
C	0.940868	-0.446784	1.979985
C	1.978659	-2.252609	2.454139

C	1.825330	-1.577336	3.658933
C	2.295485	-2.123500	4.846924
H	2.172286	-1.684360	5.680732
C	2.949635	-3.333046	4.757173
H	3.301124	-3.720372	5.550470
C	3.116527	-4.006620	3.563972
H	3.577700	-4.836484	3.552249
C	2.622231	-3.487636	2.392916
H	2.714818	-3.953957	1.570961
C	0.778936	0.667628	4.345285
C	0.238057	1.906054	3.655416
H	-0.608547	1.689457	3.212264
H	0.885777	2.216934	2.988214
H	0.089756	2.611056	4.319493
C	2.013910	1.096575	5.137539
H	2.699739	1.426143	4.520096
H	2.364962	0.329567	5.636443
H	1.768984	1.809222	5.764100
C	-0.298308	0.096832	5.263633
H	-1.103893	-0.099380	4.740239
H	-0.512927	0.750020	5.960796
H	0.029228	-0.728785	5.679429
Se	0.075110	-0.755202	-0.903016
N	-1.417486	1.528920	-1.419988
N	-1.159671	0.377134	-3.350994
C	-0.940868	0.446784	-1.979985
C	-1.978659	2.252609	-2.454139

C	-1.825330	1.577336	-3.658933
C	-2.295485	2.123500	-4.846924
H	-2.172286	1.684360	-5.680732
C	-2.949635	3.333046	-4.757173
H	-3.301124	3.720372	-5.550470
C	-3.116527	4.006620	-3.563972
H	-3.577700	4.836484	-3.552249
C	-2.622231	3.487636	-2.392916
H	-2.714818	3.953957	-1.570961
C	-0.778936	-0.667628	-4.345285
C	-0.238057	-1.906054	-3.655416
H	0.608547	-1.689457	-3.212264
H	-0.885777	-2.216934	-2.988214
H	-0.089756	-2.611056	-4.319493
C	-2.013910	-1.096575	-5.137539
H	-2.699739	-1.426143	-4.520096
H	-2.364962	-0.329567	-5.636443
H	-1.768984	-1.809222	-5.764100
C	0.298308	-0.096832	-5.263633
H	1.103893	0.099380	-4.740239
H	0.512927	-0.750020	-5.960796
H	-0.029228	0.728785	-5.679429
**6**			
N	4.094969	2.429651	8.534136
C	4.537605	2.513459	7.330999
Se	3.736311	1.277511	6.090002
N	5.534954	3.429130	6.922942

C	6.129857	4.277088	7.911533
O	6.985110	5.105830	7.583405
C	5.641727	4.106891	9.233862
H	6.011216	4.633056	9.933588
C	4.670460	3.222407	9.528336
C	4.053928	3.020788	10.875905
H	3.980869	2.047999	11.048382
H	3.136207	3.391617	10.863284
C	4.819656	3.660760	12.025755
H	4.352646	3.482153	12.868512
H	4.876519	4.627916	11.881323
H	5.723103	3.283319	12.066421
N	5.265719	2.697737	4.752249
C	5.904070	3.495791	5.551535
N	6.867934	4.332430	5.146283
H	7.255580	4.855311	5.739438
C	7.278857	4.400793	3.807128
O	8.177328	5.171308	3.457966
C	6.541492	3.492602	2.926510
H	6.731130	3.479126	1.996814
C	5.606746	2.686065	3.424311
C	4.828287	1.673128	2.630634
H	3.869875	1.919581	2.661484
H	4.923529	0.791169	3.068138
C	5.242476	1.537724	1.179298
H	4.683065	0.861080	0.741794
H	6.182035	1.263890	1.131621

H	5.128931	2.398180	0.724967
N	2.114082	-0.837453	5.488434
C	1.671446	-0.921261	6.691570
Se	2.472740	0.314687	7.932567
N	0.674097	-1.836932	7.099627
C	0.079194	-2.684890	6.111036
O	-0.776059	-3.513632	6.439164
C	0.567324	-2.514693	4.788707
H	0.197835	-3.040858	4.088981
C	1.538591	-1.630208	4.494233
C	2.155123	-1.428590	3.146665
H	2.228182	-0.455801	2.974187
H	3.072844	-1.799419	3.159285
C	1.389395	-2.068562	1.996814
H	1.856405	-1.889954	1.154057
H	1.332532	-3.035718	2.141246
H	0.485948	-1.691120	1.956148
N	0.943332	-1.105539	9.270320
C	0.304981	-1.903593	8.471034
N	-0.658883	-2.740232	8.876286
H	-1.046529	-3.263113	8.283132
C	-1.069806	-2.808595	10.215442
O	-1.968277	-3.579110	10.564604
C	-0.332441	-1.900404	11.096059
H	-0.522079	-1.886927	12.025755
C	0.602305	-1.093867	10.598258
C	1.380764	-0.080930	11.391935

H	2.339176	-0.327383	11.361085
H	1.285522	0.801029	10.954431
C	0.966575	0.054474	12.843271
H	1.525986	0.731118	13.280775
H	0.027016	0.328308	12.890948
H	1.080120	-0.805982	13.297602
**7**			
Se	3.136558	3.567720	5.726201
C	1.501623	3.266757	4.799437
N	0.703043	2.409974	5.458940
C	-0.270491	4.473923	1.871721
H	0.242600	4.117438	1.142017
H	-0.046182	5.398690	1.999271
H	-1.205944	4.395479	1.672981
N	1.258405	3.927430	3.679173
C	0.041083	3.706915	3.123490
C	-0.844443	2.850132	3.715757
H	-1.673716	2.693244	3.325197
C	-0.505448	2.223452	4.887437
C	-1.455312	1.331805	5.607253
H	-1.036058	0.991881	6.401227
H	-1.702646	0.599661	5.037728
H	-2.240854	1.828617	5.848511
Se	3.871866	5.148280	4.161391
C	5.506801	5.449243	5.088155
N	6.305381	6.306026	4.428653
C	7.278915	4.242077	8.015871

H	6.765824	4.598562	8.745575
H	7.054606	3.317310	7.888321
H	8.214368	4.320521	8.214612
N	5.750019	4.788570	6.208419
C	6.967341	5.009085	6.764102
C	7.852867	5.865868	6.171835
H	8.682140	6.022756	6.562395
C	7.513872	6.492548	5.000155
C	8.463736	7.384195	4.280339
H	8.044482	7.724119	3.486365
H	8.711071	8.116339	4.849864
H	9.249278	6.887383	4.039081
**8**			
Te	0.394557	0.000000	1.303316
N	3.311283	0.000000	1.623475
C	2.403216	0.000000	0.645523
N	2.621167	-0.000000	-0.667834
C	3.907284	-0.000000	-1.056040
C	4.933892	-0.000000	-0.117503
H	5.845051	-0.000000	-0.389693
C	4.591122	0.000000	1.224857
C	4.188226	-0.000000	-2.526314
H	3.792067	-0.777064	-2.927907
H	5.133774	-0.000000	-2.692901
C	5.624000	0.000000	2.314363
H	5.509045	-0.777064	2.866181
H	6.507276	0.000000	1.938799

Te	-0.394557	-0.000000	-1.303316
N	-3.311283	-0.000000	-1.623475
C	-2.403216	-0.000000	-0.645523
N	-2.621167	0.000000	0.667834
C	-3.907284	0.000000	1.056040
C	-4.933892	0.000000	0.117503
H	-5.845051	0.000000	0.389693
C	-4.591122	-0.000000	-1.224857
C	-4.188226	0.000000	2.526314
H	-3.792067	-0.777064	2.927907
H	-5.133774	0.000000	2.692901
C	-5.624000	-0.000000	-2.314363
H	-5.509045	-0.777064	-2.866181
H	-6.507276	-0.000000	-1.938799
H	-3.792067	0.777064	2.927907
H	-5.509045	0.777064	-2.866181
H	3.792067	0.777064	-2.927907
H	5.509045	0.777064	2.866181

X-ray crystal structure determination

The single-crystal X-ray diffraction data for **3b** and **3g** were collected on a four-circle Rigaku Synergy S diffractometer equipped with a HyPix6000HE area-detector (T = 100 K, λ(Cu$K\alpha$)-radiation, graphite monochromator, shutterless ω-scan mode). The data were integrated and corrected for absorption by the *CrysAlisPro* program [1]. The single-crystal X-ray diffraction data for **3f**, **4b** and **4c** were collected on a three-circle Bruker D8 QUEST PHOTON-III CCD diffractometer (T = 100 K, λ(Mo$K\alpha$)-radiation, graphite monochromator, ω and φ scanning mode). The data were indexed and integrated using the *SAINT* program [2], and then scaled and corrected for absorption using the *SADABS* program [3]. For details, see Table 1.

The structures were determined by direct methods and refined by full-matrix least squares technique on F^2 with anisotropic displacement parameters for non-hydrogen atoms. One of the two trifluoromethyl-substituents in **3g** is disordered over three positions with the occupancies of 0.55:0.35:0.15. The hydrogen atoms were placed in calculated positions and refined within riding model with fixed isotropic displacement parameters [U_{iso}(H) = 1.5U_{eq}(C) for the CH_3-groups and 1.2U_{eq}(C) for the other groups]. All calculations were carried out using the SHELXTL program [4].

Crystallographic data for all investigated compounds have been deposited with the Cambridge Crystallographic Data Center, CCDC 2142342 (**3b**), CCDC 1971645 (**3f**), CCDC 2142343 (**3g**), CCDC 2201113 (**4b**) and CCDC 2201114 (**4c**). Copies of this information may be obtained free of charge from the Director, CCDC, 12 Union Road, Cambridge CB2 1EZ, UK (Fax: +44 1223 336033; e-mail: deposit@ccdc.cam.ac.uk or www.ccdc.cam.ac.uk).

References

[1] Rigaku, *CrysAlisPro Software System, v. 1.171.41.106a*, Rigaku Oxford Diffraction, **2021**.

[2] Bruker, *SAINT*, *v. 8.34A*, Bruker AXS Inc., Madison, WI, **2014**.

[3] L. Krause, R. Herbst-Irmer, G. M. Sheldrick, D. Stalke, *J. Appl. Cryst.* **2015**, *48*, 3-10.

[4] G. M. Sheldrick, *Acta Cryst.* **2015**, *C71*, 3-8.

Table S2. Crystal data and structure refinement for all compounds studied.

Identification code	**3b**	**3f**	**3g**	**4b**	**4c**
Empirical formula	$C_{15}H_{12}N_2OSe$	$C_{14}H_9ClN_2OSe$	$C_{15}H_8ClF_3N_2OSe$	$C_{30}H_{22}N_4O_2Se_2$	$C_{30}H_{22}N_4O_4Se_2$
Formula weight	315.23	335.64	403.64	628.44	660.44
Crystal size, mm	0.03 × 0.04 × 0.17	0.20 × 0.30 × 0.30	0.06 × 0.20 × 0.20	0.15 × 0.15 × 0.20	0.06 × 0.10 × 0.12
Crystal system	Monoclinic	Triclinic	Monoclinic	Monoclinic	Monoclinic
Space group	$P2_1$/c	P-1	$P2_1$/c	$P2_1$/c	$P2_1$/c
a, Å	18.8029(2)	10.0455(6)	13.20125(7)	13.0204(4)	13.2108(10)
b, Å	13.36160(10)	10.5826(6)	9.42592(4)	9.7319(3)	9.5265(8)
c, Å	10.51280(10)	13.3337(8)	24.72066(11)	10.7522(5)	11.3252(9)
α, deg.	90	107.540(2)	90	90	90
β, deg.	96.882(1)	96.5000(10)	95.7824(5)	107.055(4)	113.031(2)
γ, deg.	90	96.750(2)	90	90	90
V, Å^3	2622.17(4)	1325.56(14)	3060.44(2)	1302.53(9)	1311.70(18)
Z	8	4	8	2	2
Density (calc.), Mg/m^3	1.597	1.682	1.752	1.602	1.672
μ, mm^{-1}	3.816	3.026	5.290	2.875	2.865
F(000)	1264	664	1584	628	660
Theta range, deg.	2.37 – 79.64	2.67 – 32.75	3.37 – 79.59	2.66 – 35.40	2.72 – 27.50
Index ranges	$-23 \le h \le 23$, $-16 \le k \le 17$, $-13 \le l \le 13$	$-15 \le h \le 15$, $-15 \le k \le 16$, $-20 \le l \le 20$	$-16 \le h \le 16$, $-12 \le k \le 10$, $-30 \le l \le 31$	$-20 \le h \le 20$, $-15 \le k \le 15$, $-17 \le l \le 16$	$-17 \le h \le 17$, $-12 \le k \le 12$, $-14 \le l \le 14$
Reflections collected	30554	23560	43467	48448	21428
Independent reflections, R_{int}	5654, 0.0339	9640, 0.0595	6635, 0.0426	5509, 0.0659	3018, 0.0910
Reflections observed	5215	7507	6260	4442	1892
R_1 / wR_2 ($I > 2\sigma(I)$)	0.0336 / 0.0868	0.0451 / 0.1077	0.0326 / 0.0846	0.1065 / 0.1846	0.0542 / 0.0934
R_1 / wR_2 (all data)	0.0362 / 0.0887	0.0630 / 0.1169	0.0340 / 0.0858	0.1369 / 0.1970	0.1103 / 0.1169
Goodness-of-fit on F^2	1.039	1.017	1.050	1.064	1.024
Extinction coefficient	–	0.0032(3)	–	–	0.0021(2)
T_{min} / T_{max}	0.555 / 0.808	0.404 / 0.525	0.366 / 0.693	0.532 / 0.633	0.734 / 0.847
$\Delta\rho_{max}$ / $\Delta\rho_{min}$, e·Å^{-3}	1.089 / -0.859	2.254 / -1.719	0.844 / -1.000	1.677 / -0.931	0.772 / -1.055

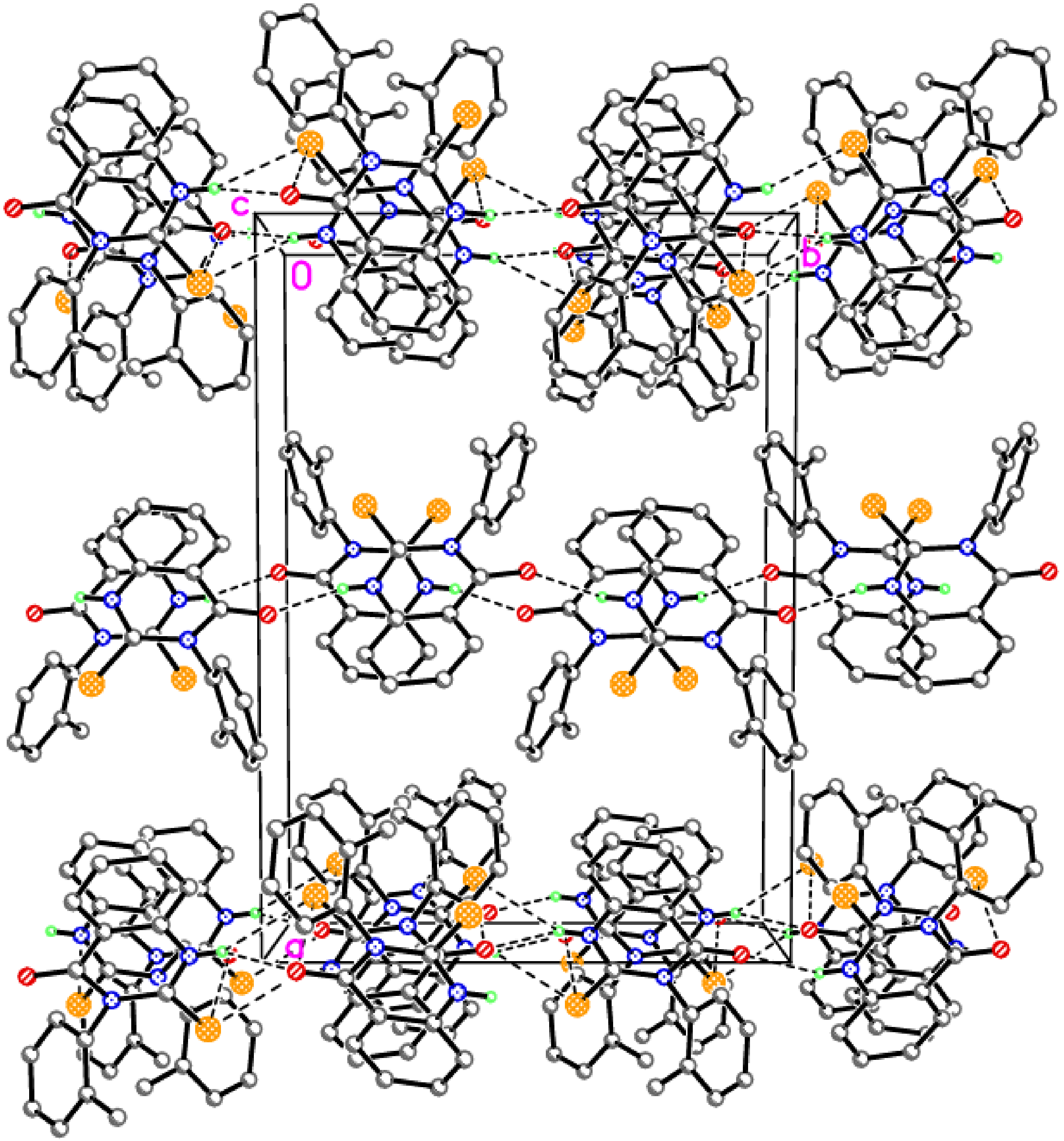

Figure S1. Crystal packing of **3b** demonstrating the H-bonded chains of the crystallographically independent molecules ***A*** (Fig. S1a, see below) and the layers of the crystallographically independent molecules ***B*** parallel to (100) (Fig. S1b, see below). Within the layers, molecules ***B*** are additionally bound to each other by the nonvalent Se⋯O interactions (Se2⋯O2 [*x*, 1.5-*y*, -0.5+*z*] 3.3232(18) Å).

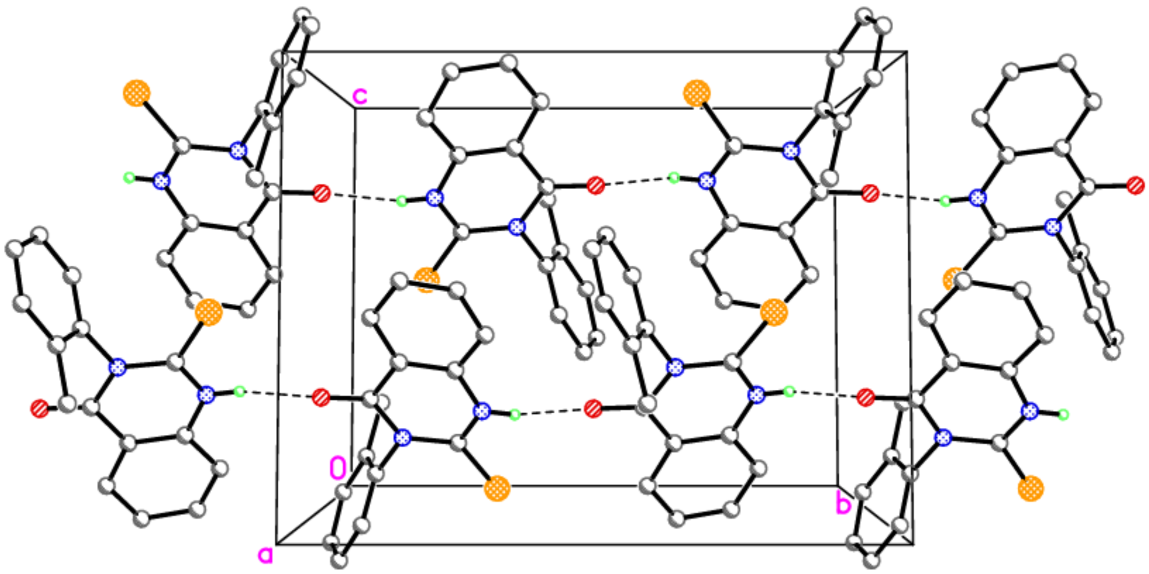

Figure S1a.

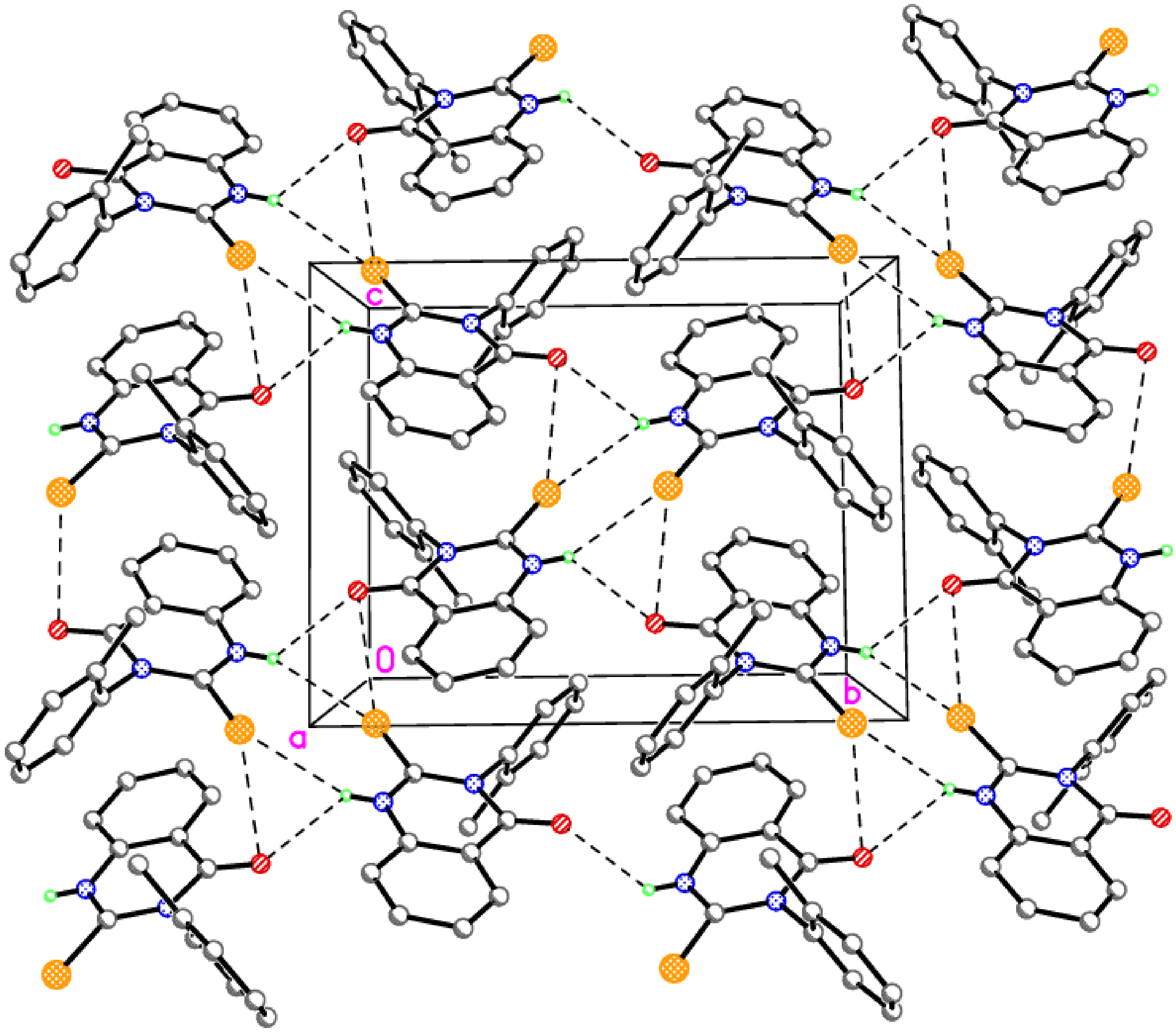

Figure S1b.

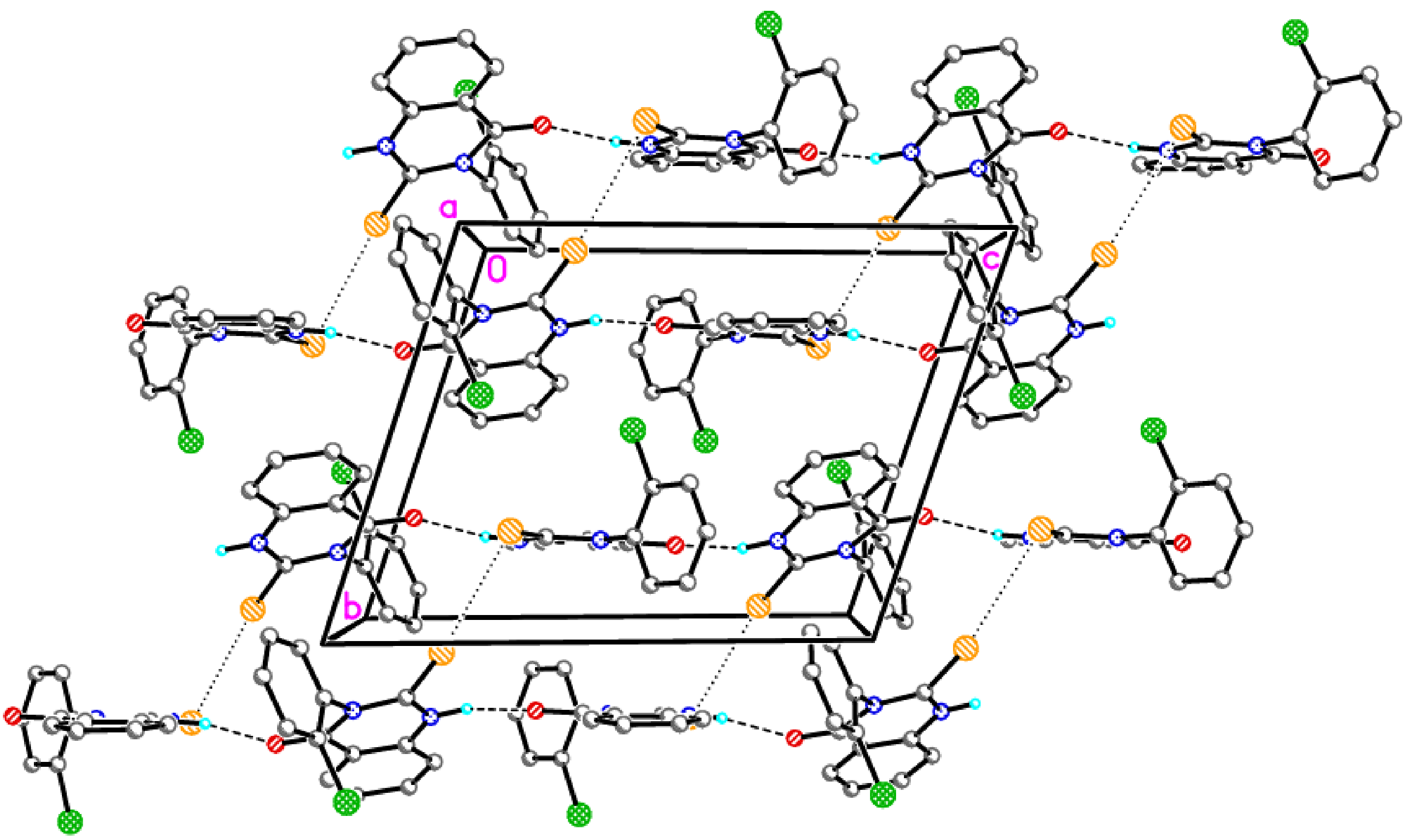

Figure S2. Crystal packing of **3f** demonstrating the ribbons towards the crystallographic ***c*** axis. Within the ribbons, the molecules are bound to each other by the strong N─H···O hydrogen bonds and weak nonvalent Se···Se interactions (Se1···Se2 [1-*x*, 2-*y*, 1-*z*] 3.7173(4) Å).

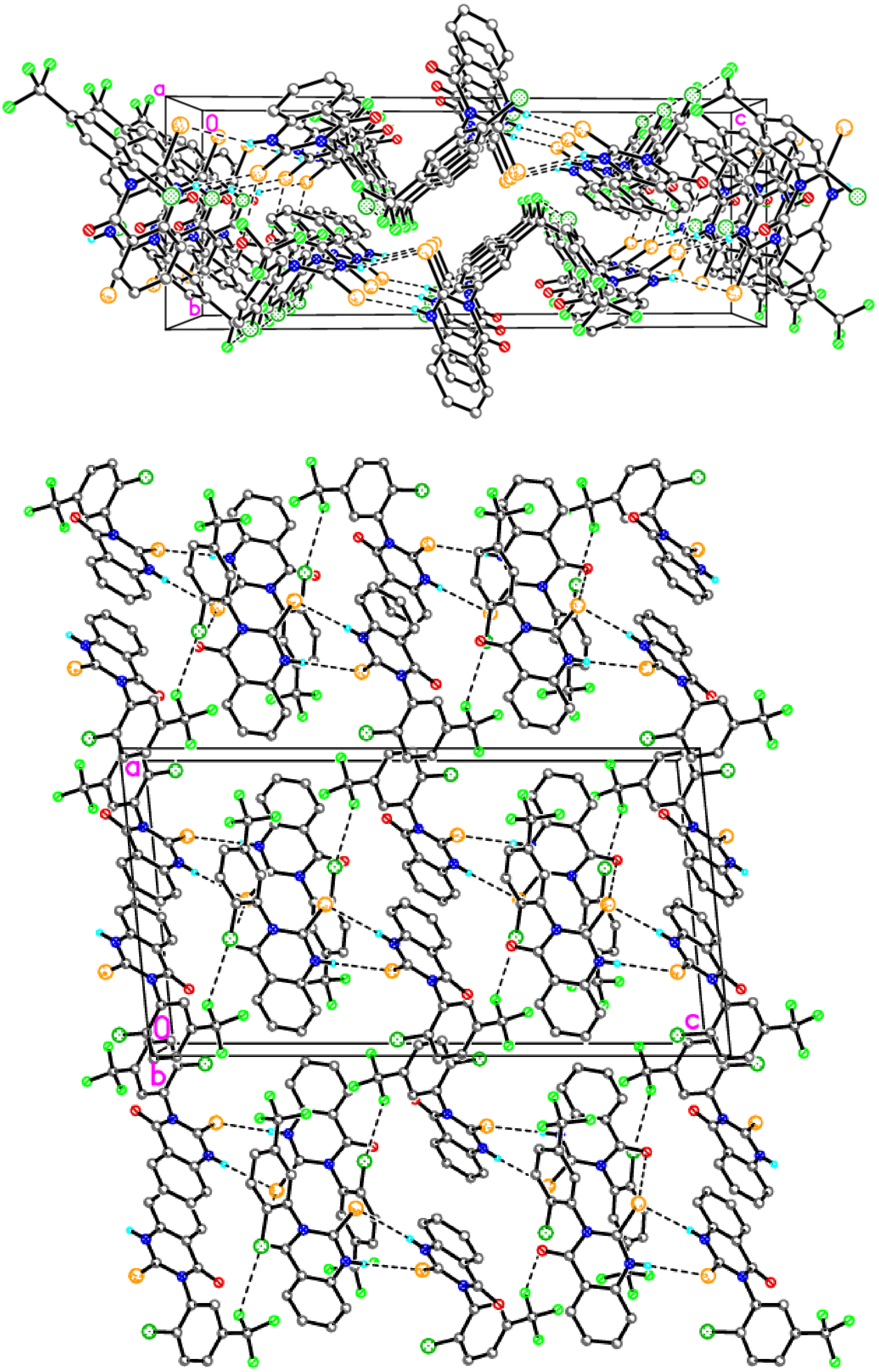

Figure S3. The two projections of crystal packing of **3g** demonstrating the two-tier layer parallel to (010). Within the layer, the molecules are bound to each other by the N─H···Se hydrogen bonds as well as the nonvalent Se···O (Se2···O2 [1-*x*, -0.5+*y*, 1.5-*z*] 3.3702(16) Å) and Cl···F (Cl2···F1 [1-*x*, -0.5+*y*, 1.5-*z*] 3.0607(17) Å) interactions.

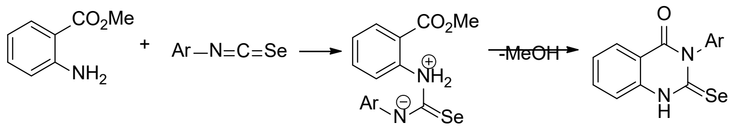

Scheme S1. Plausible mechanism for the formation of **3a–g**.

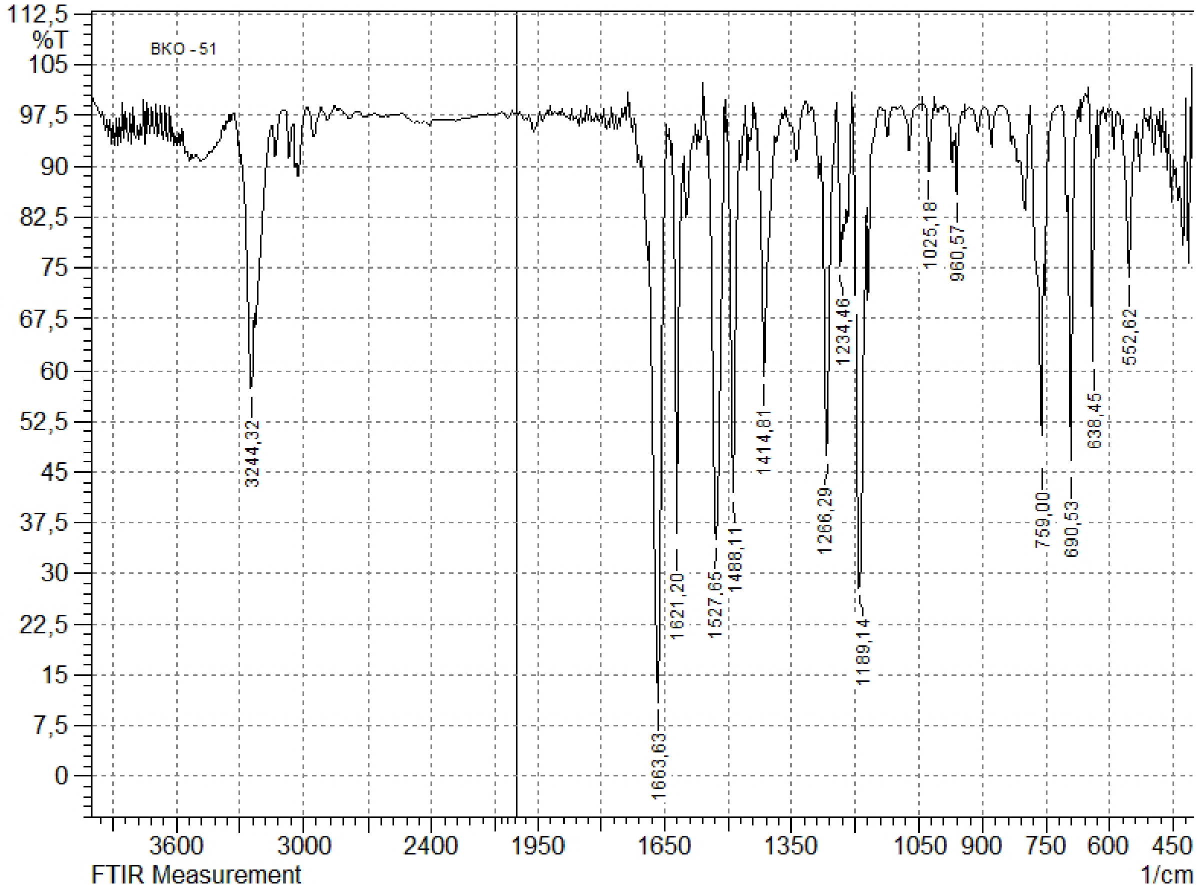

FTIR spectra of compounds **3a**

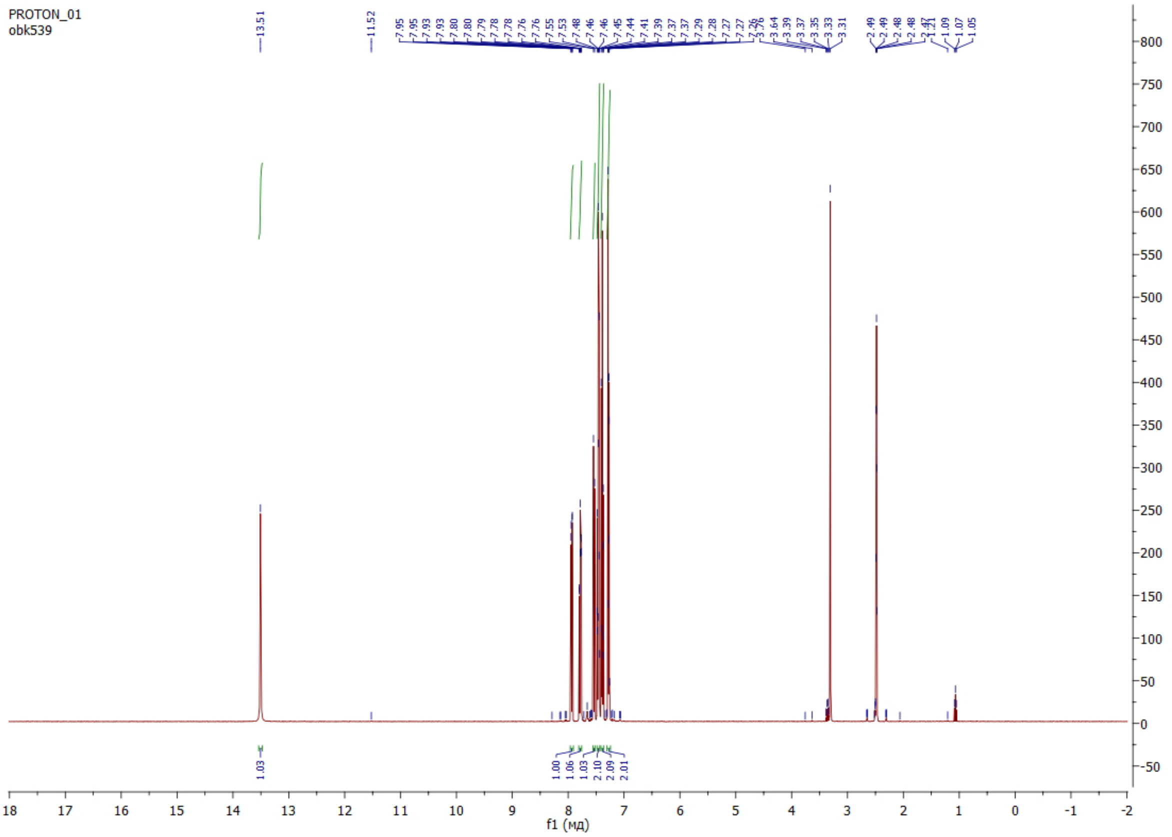

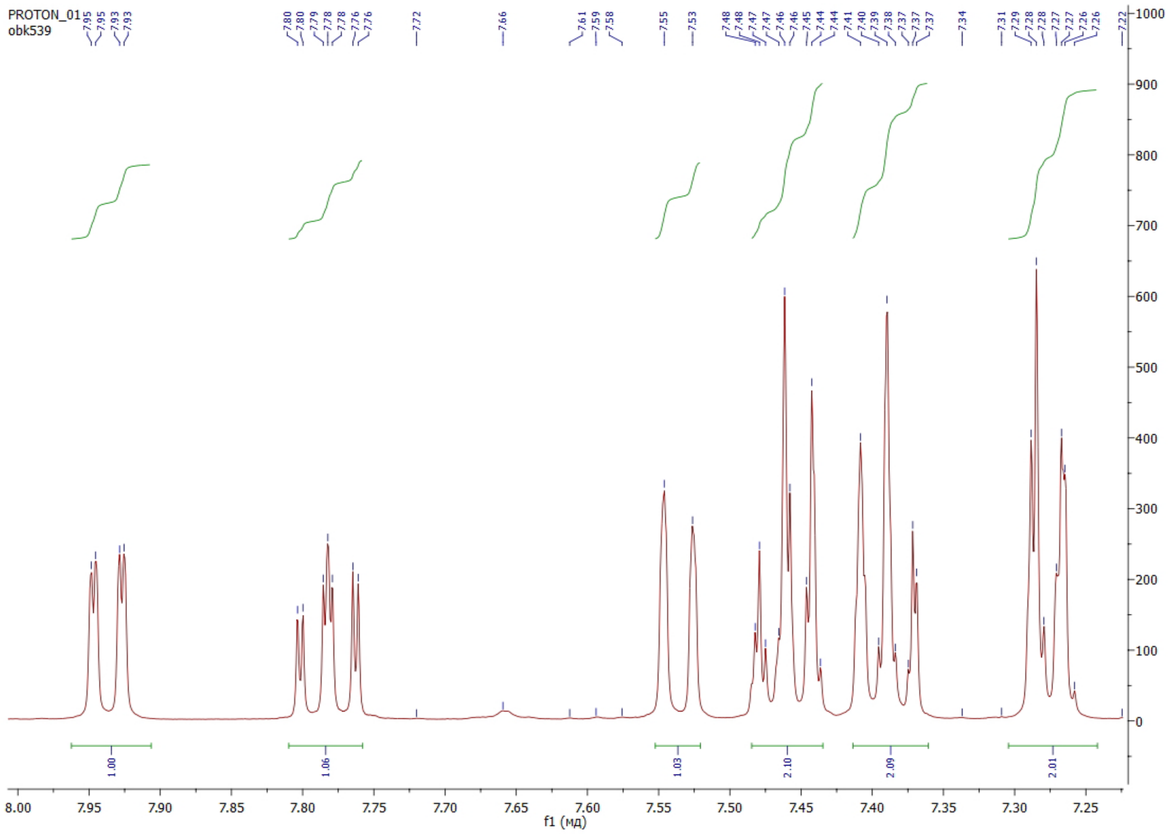

^{1}H NMR spectra of compounds **3a** (DMSO-*d*6)

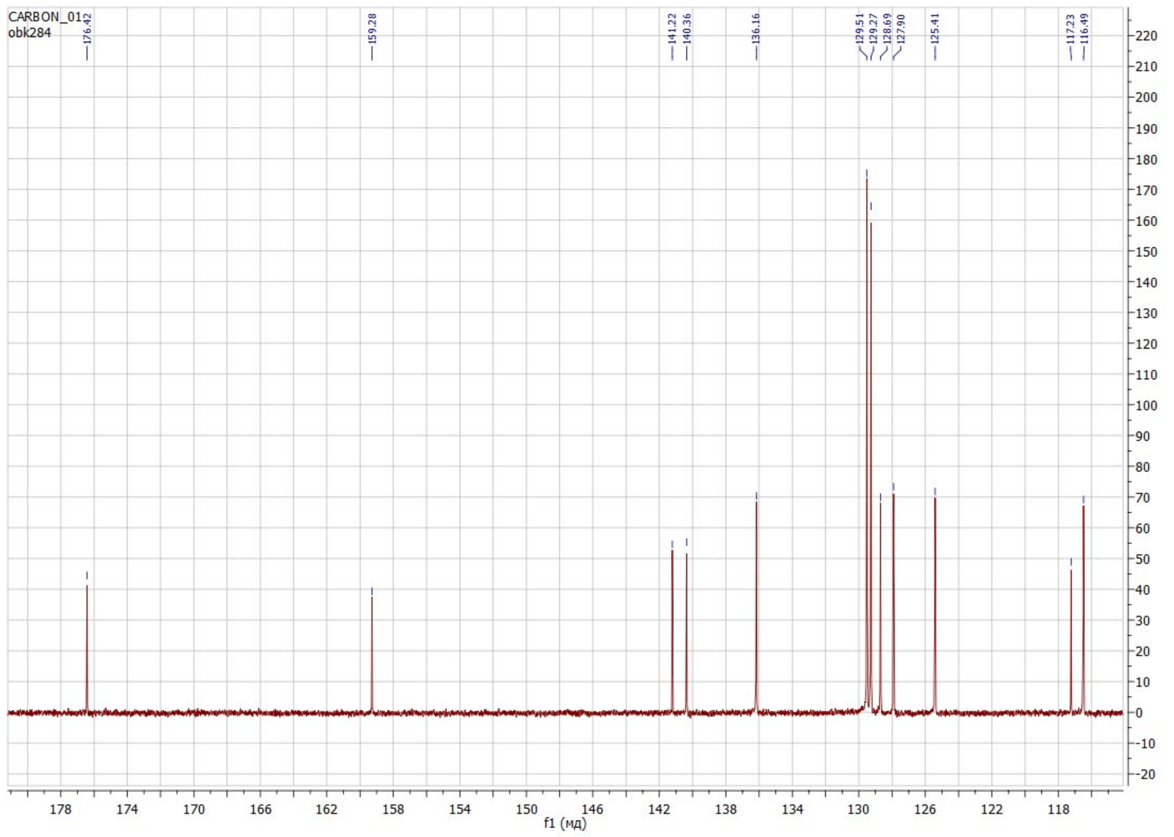

^{13}C NMR spectra of compounds **3a** (DMSO-*d*6)

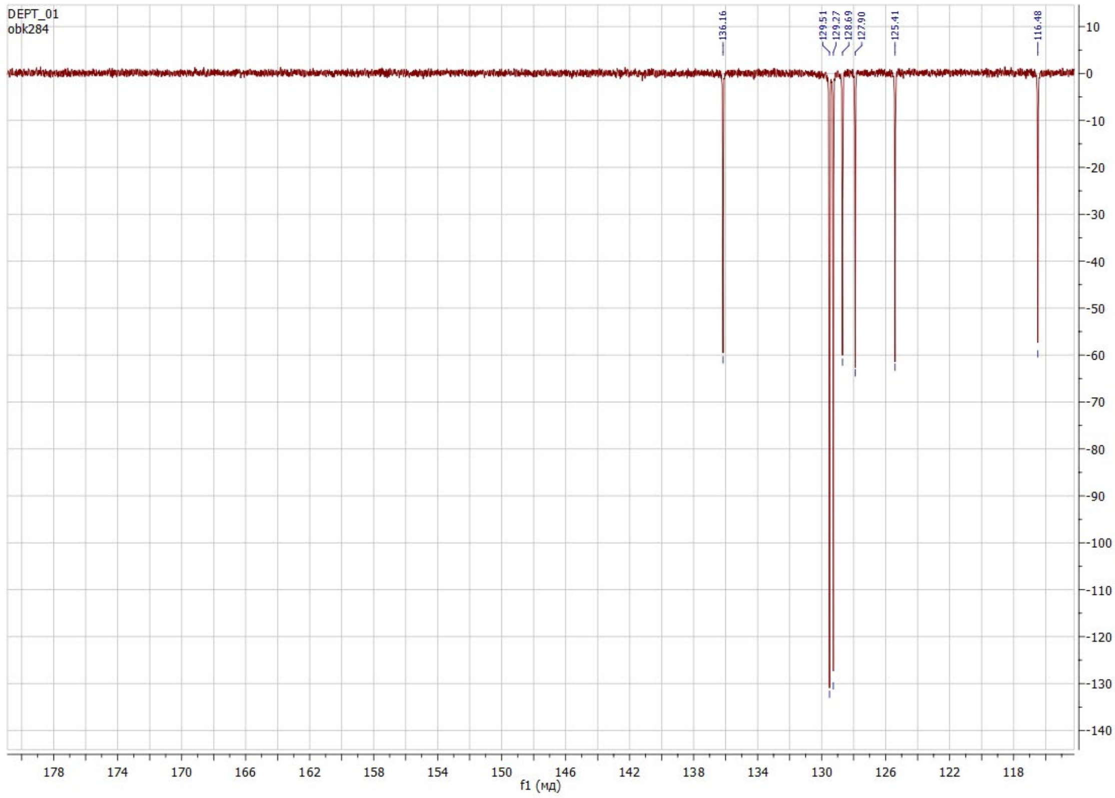

^{13}C DEPT NMR spectra of compounds **3a** (DMSO-*d*6)

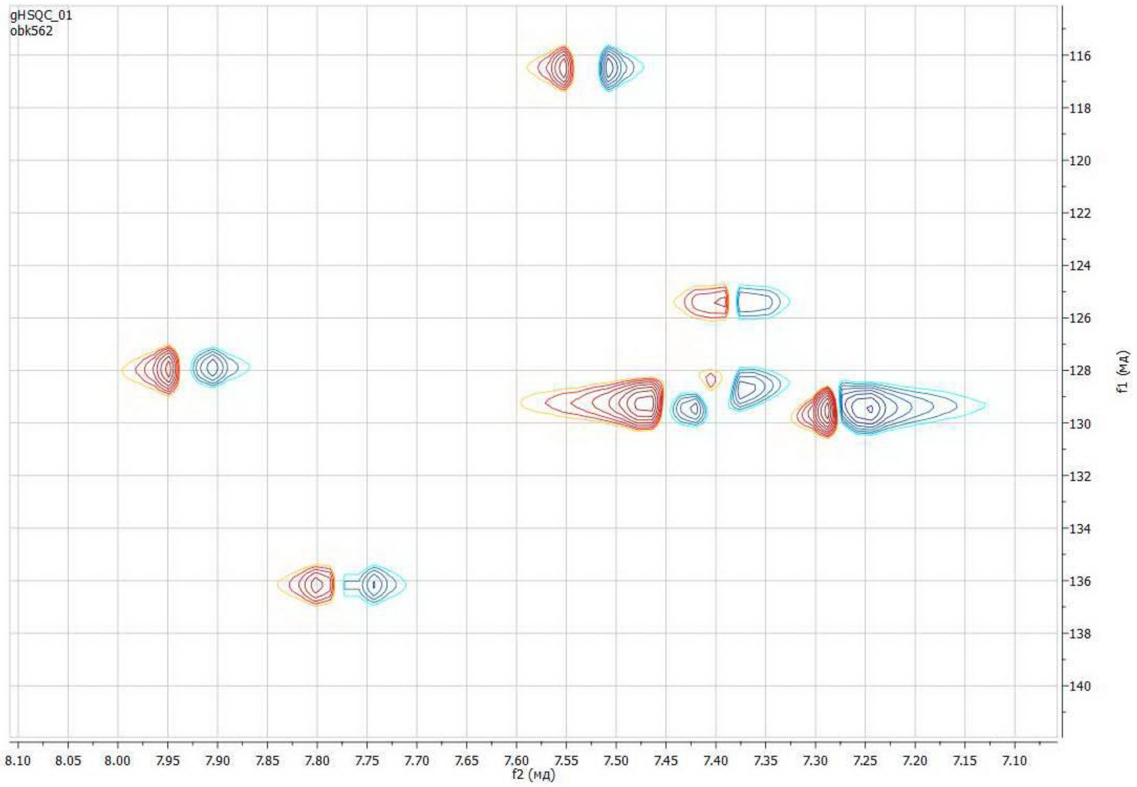

HSQC ^{1}H-^{13}C NMR spectra of compounds **3a** (DMSO-*d*6)

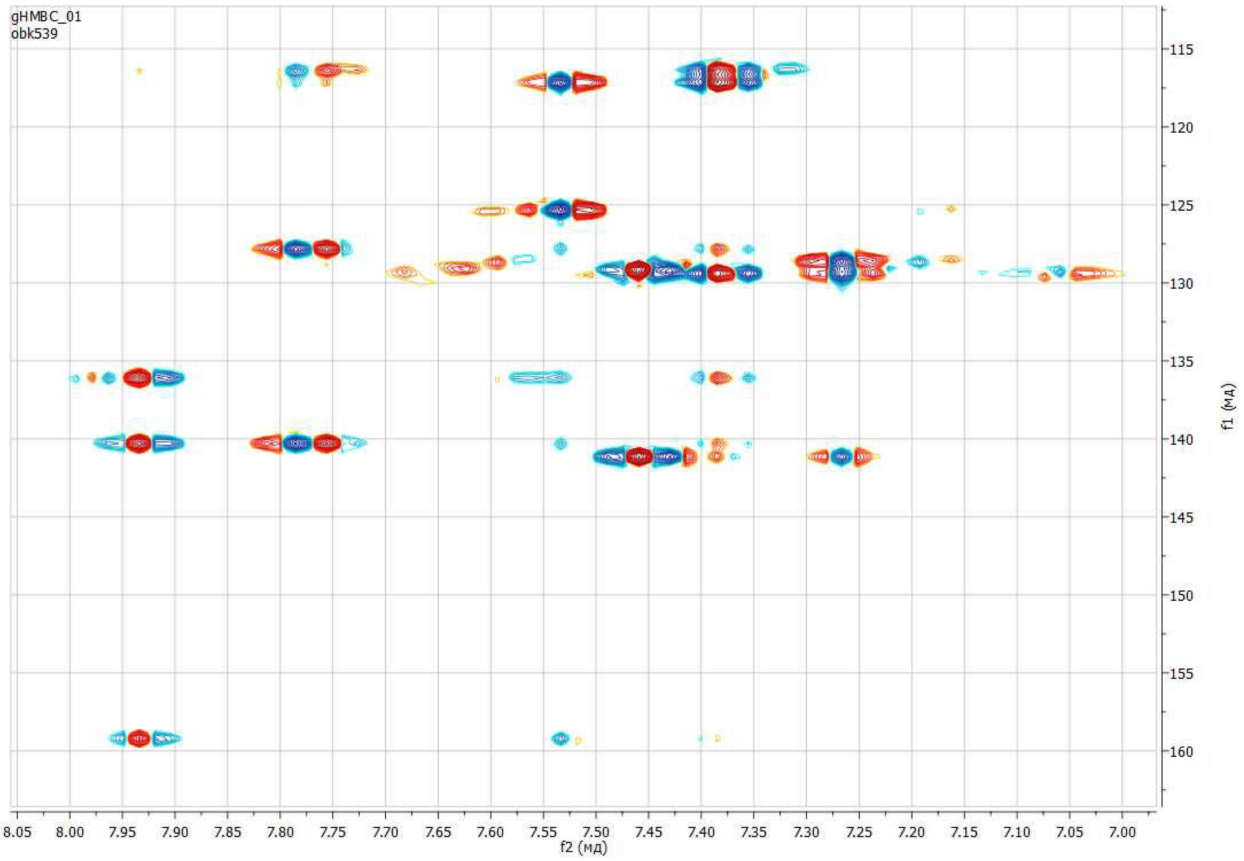

HMBC ^{1}H-^{13}C NMR spectra of compounds **3a** (DMSO-*d*6)

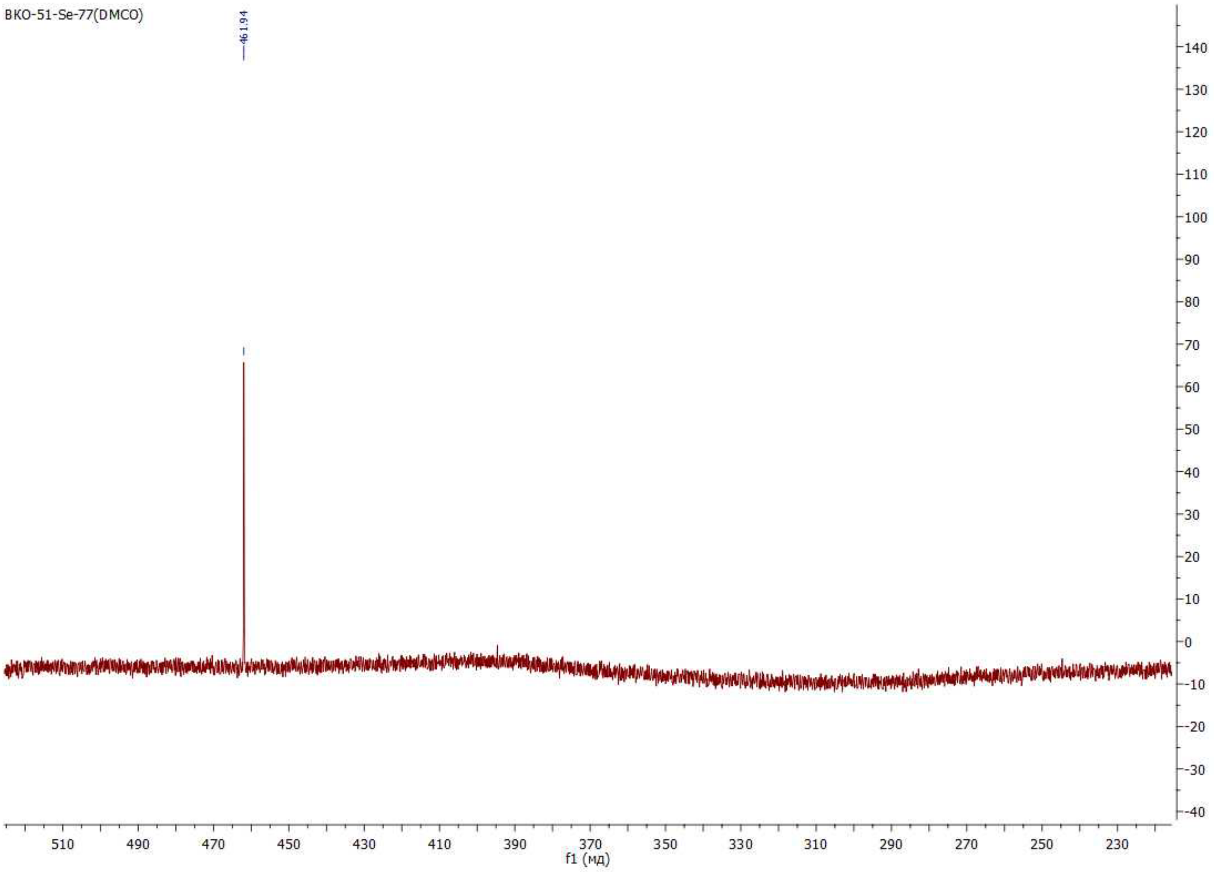

^{77}Se NMR spectra of compounds **3a** (DMSO-*d*6)

Display Report

Analysis Info

Analysis Name	C:\AOC2019\Osmanov\Nov_18\Nov_18_2019\bko-51_&clblow.d	Acquisition Date	18.11.2019 14:52:28
Method	tune_low.m	Operator	BDAL@DE
Sample Name	/CHIZ BKO-51	Instrument / Ser#	micrOTOF 10248
Comment	CH3CN 100 %, dil. 200, calibrant added		

Acquisition Parameter

Source Type	ESI	Ion Polarity	Positive	Set Nebulizer	0.4 Bar
Focus	Not active			Set Dry Heater	180 °C
Scan Begin	50 m/z	Set Capillary	4500 V	Set Dry Gas	4.0 l/min
Scan End	3000 m/z	Set End Plate Offset	-500 V	Set Divert Valve	Waste

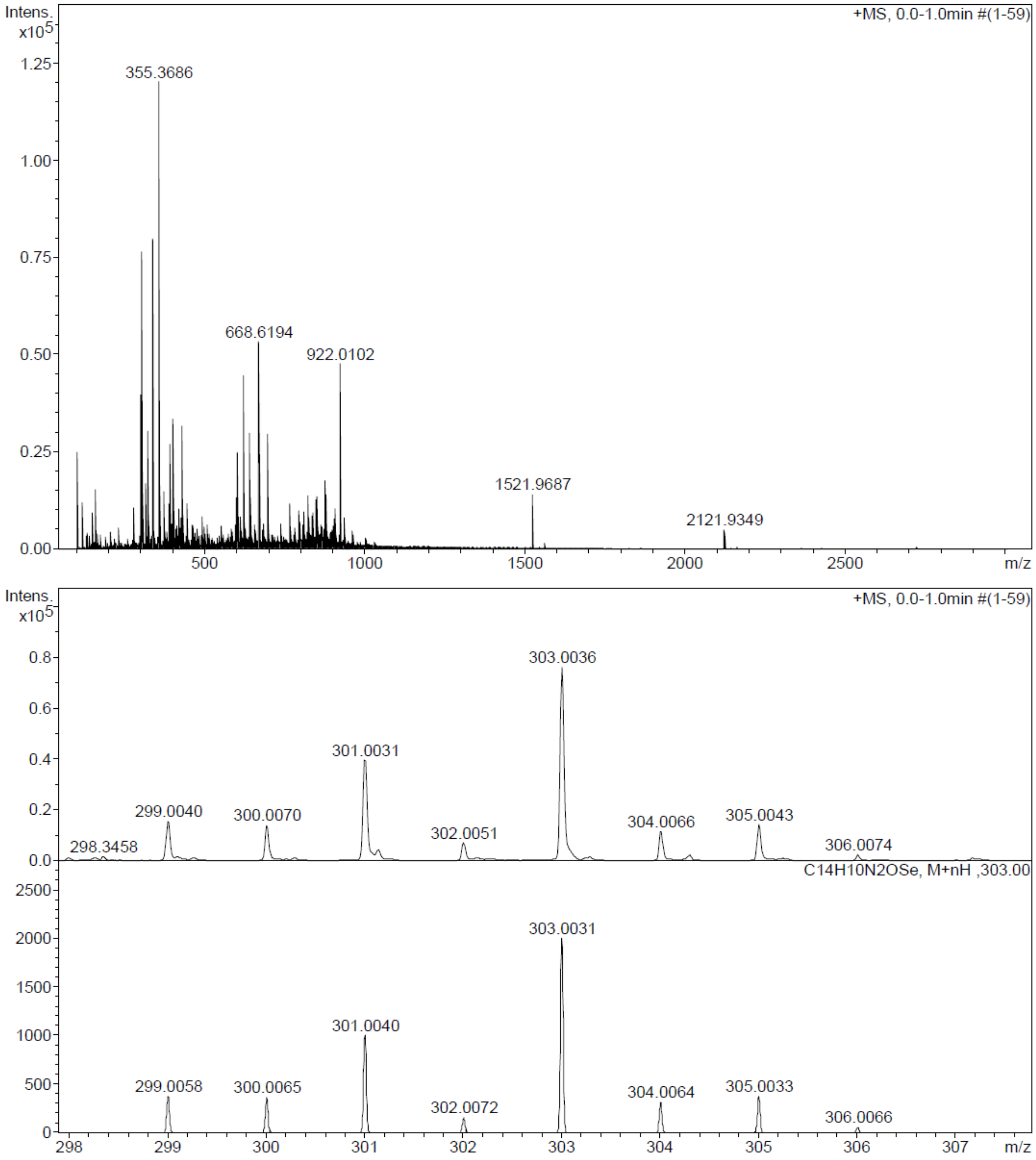

Bruker Compass DataAnalysis 4.0 printed: 20.11.2019 15:14:22

High resolution mass spectra (HR MS) of compounds **3a**

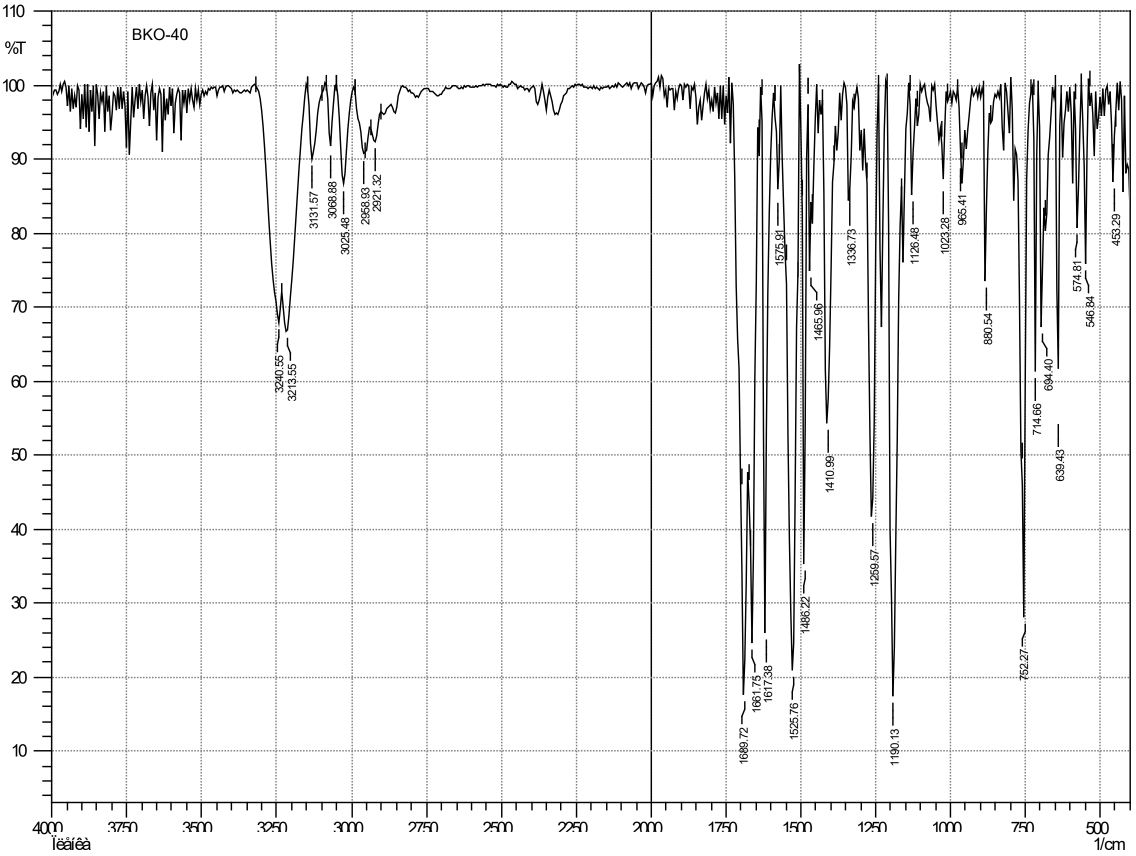

FTIR spectra of compounds **3b**

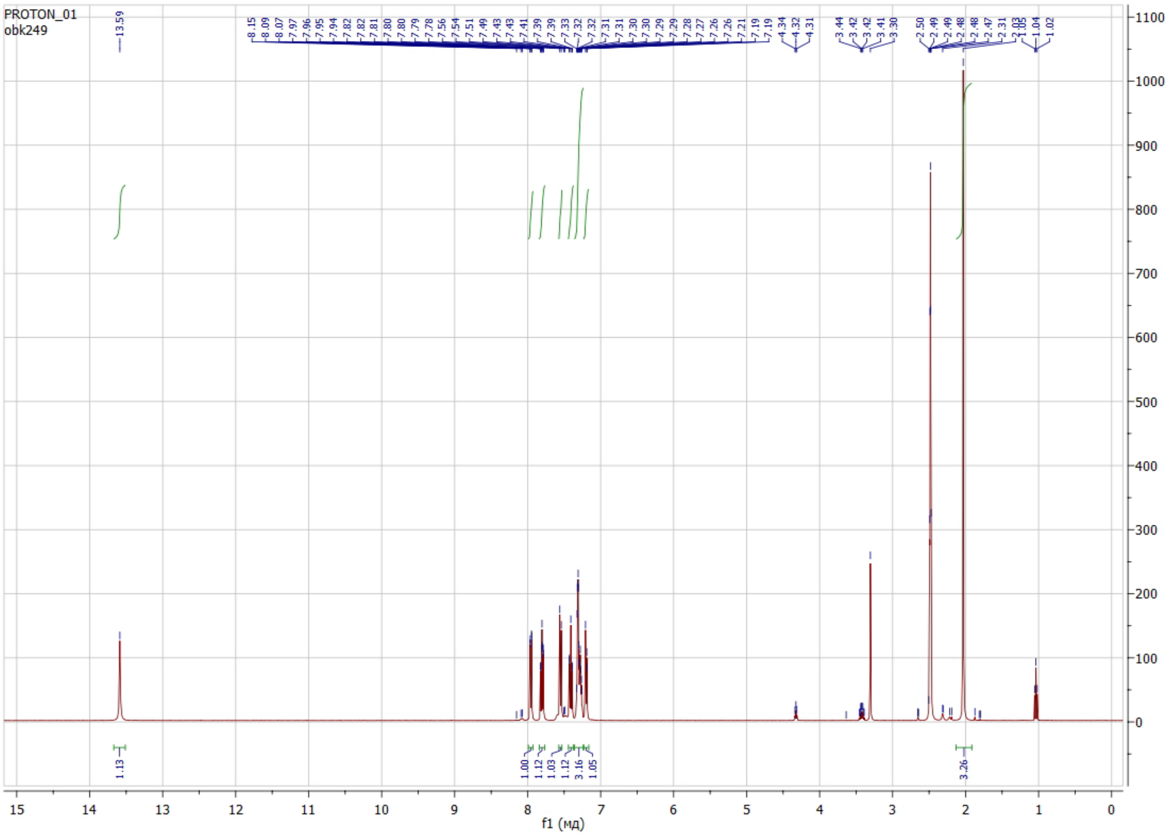

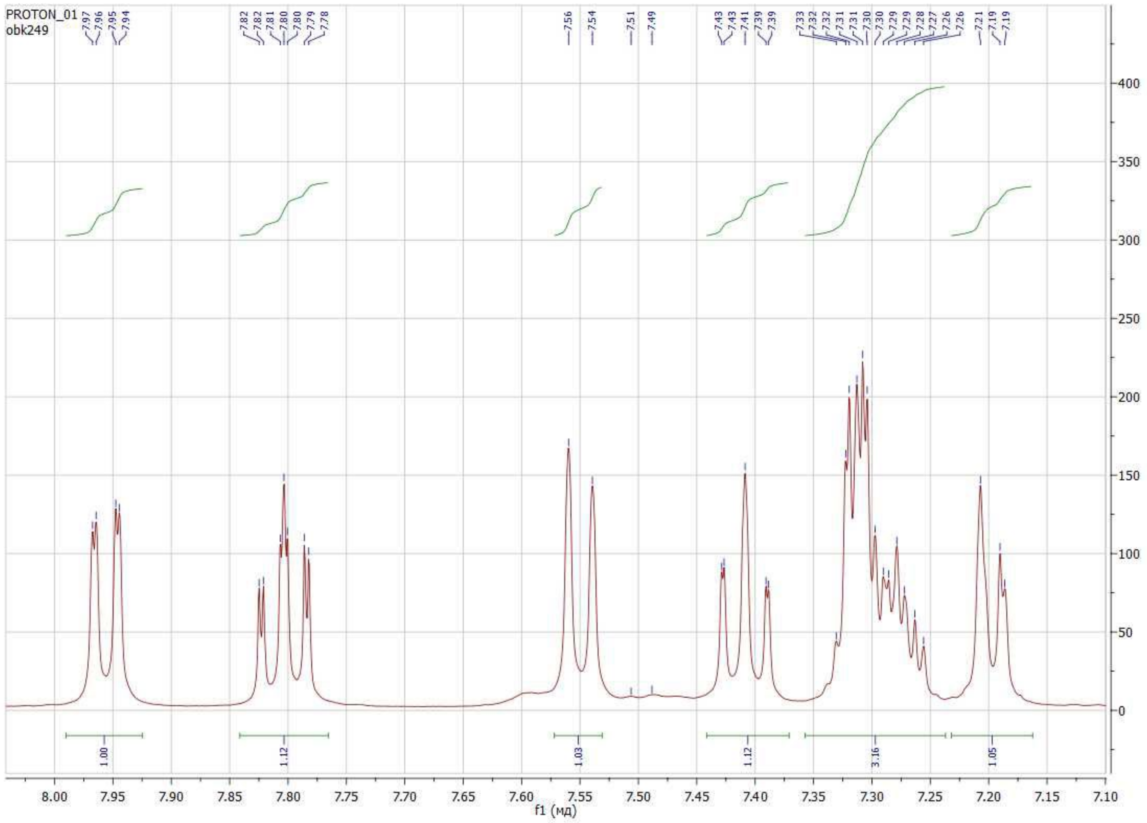

^{1}H NMR spectra of compounds **3b** (DMSO-*d*6)

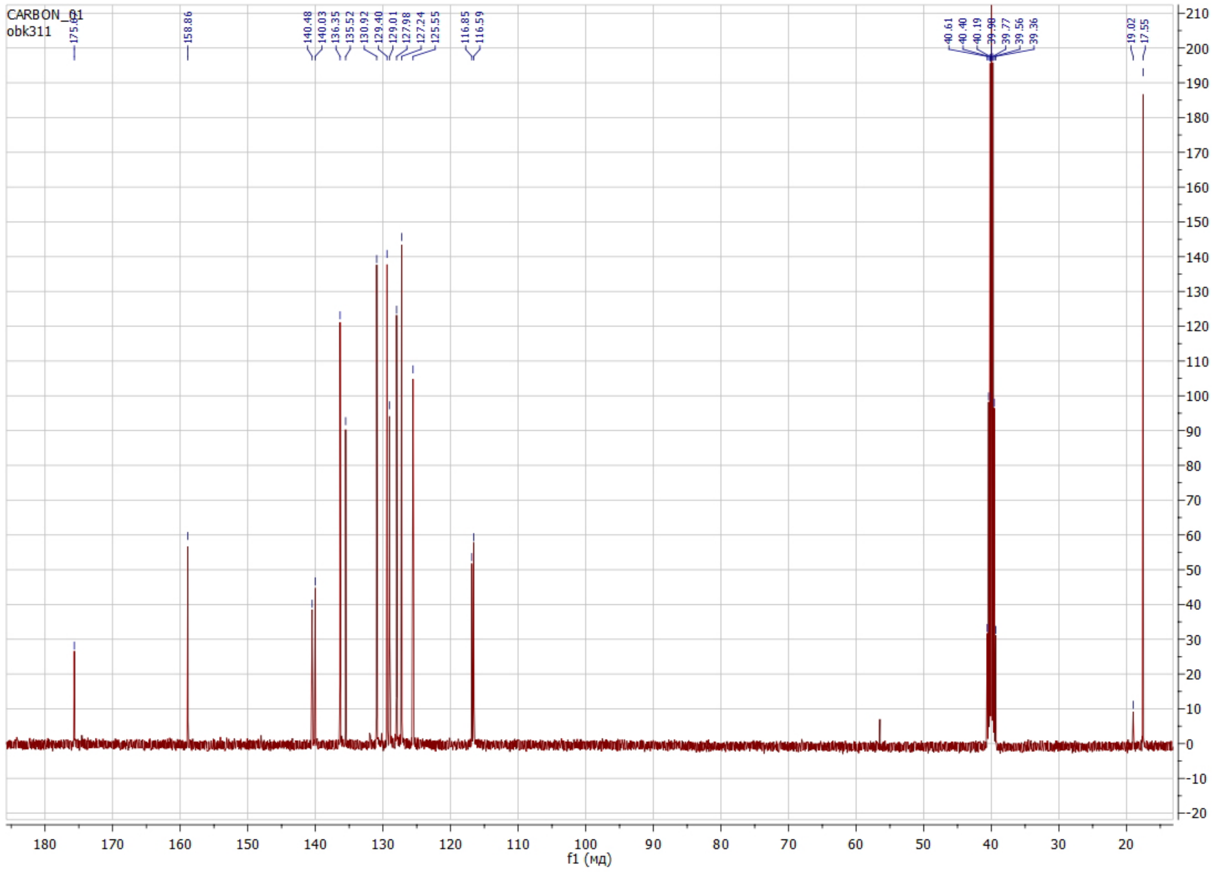

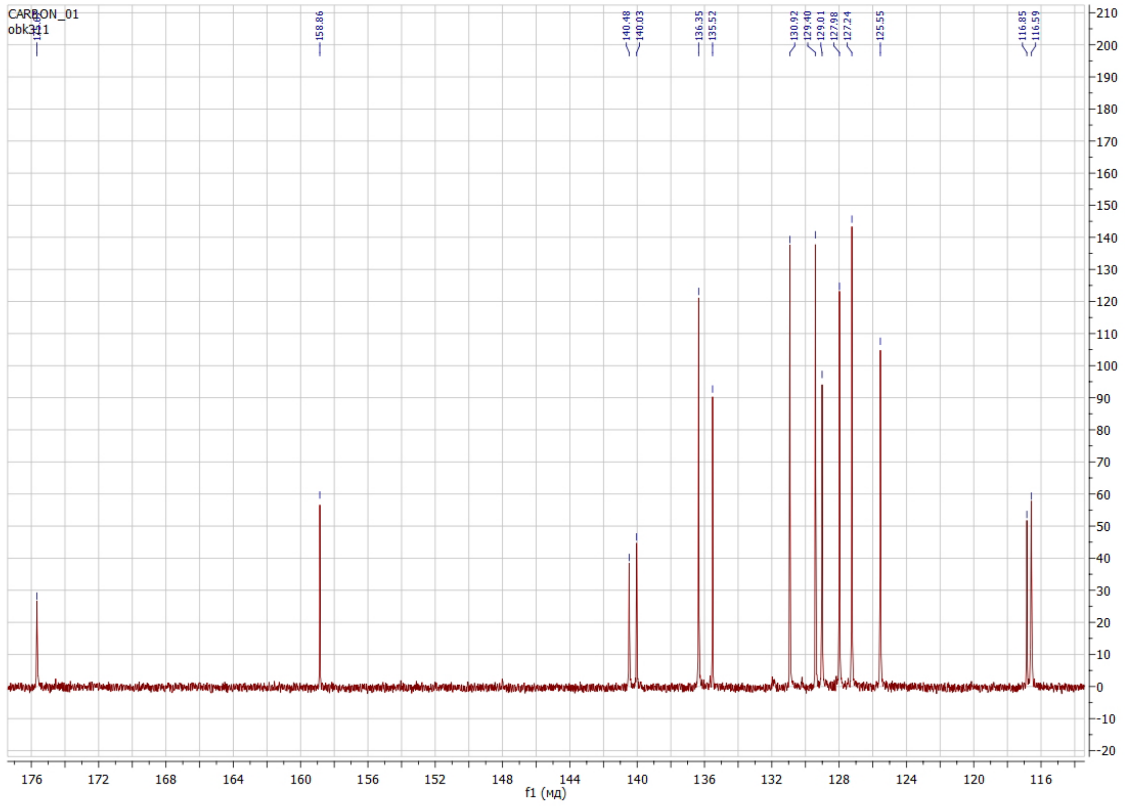

^{13}C NMR spectra of compounds **3b** (DMSO-*d*6)

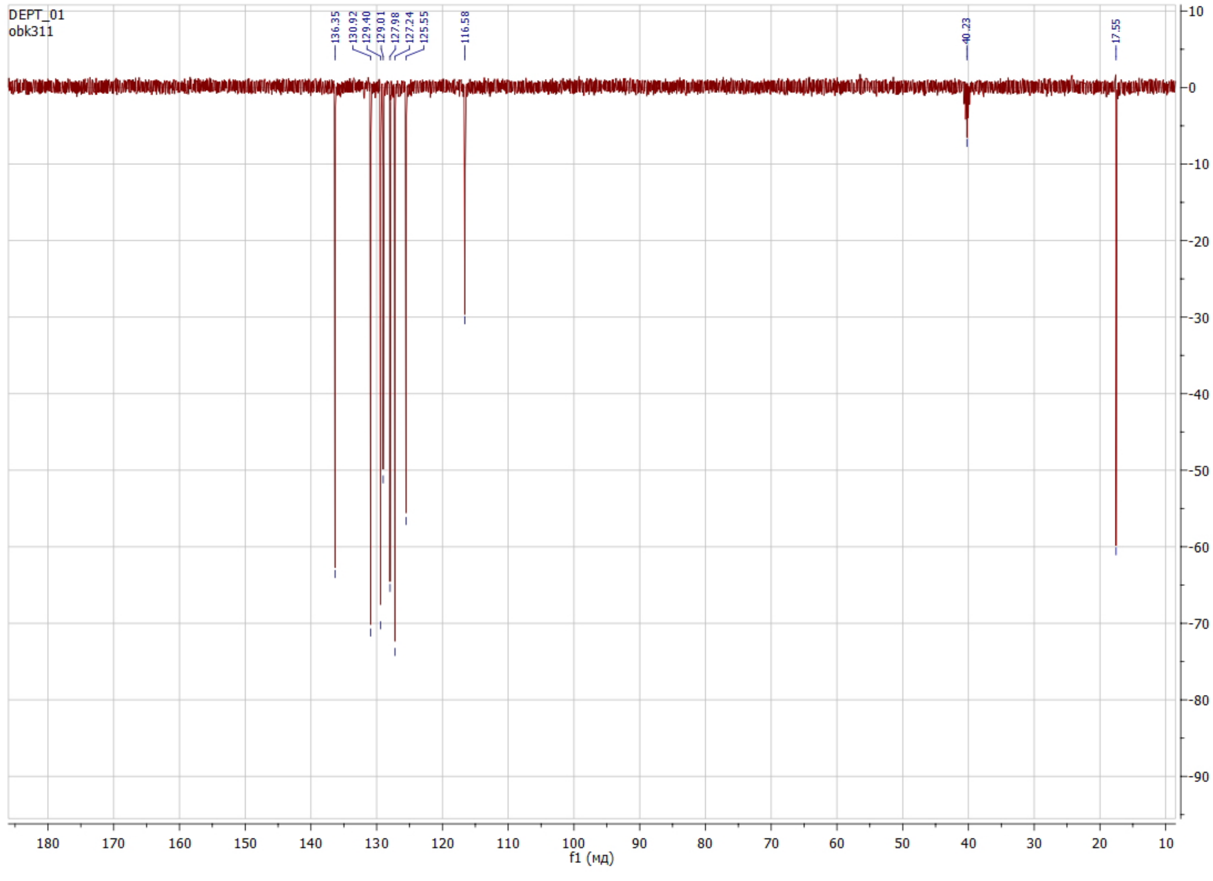

^{13}C DEPT NMR spectra of compounds **3b** (DMSO-*d*6)

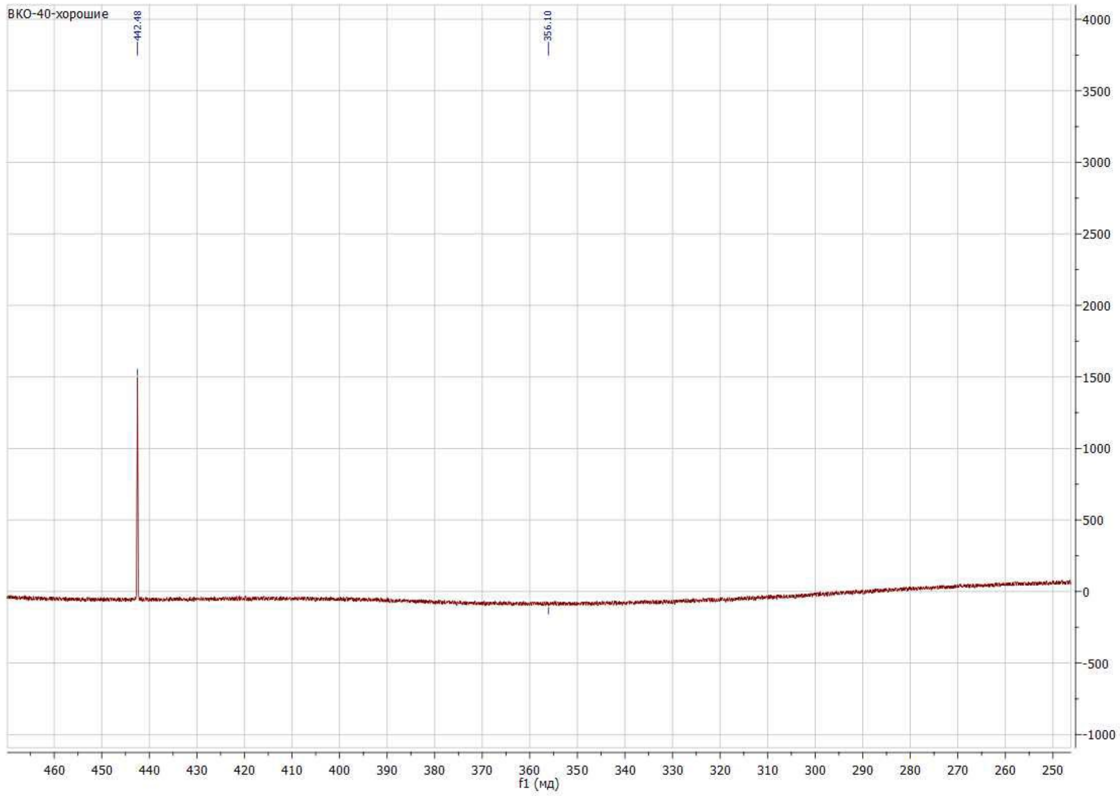

^{77}Se NMR spectra of compounds **3b** (DMSO-*d*6)

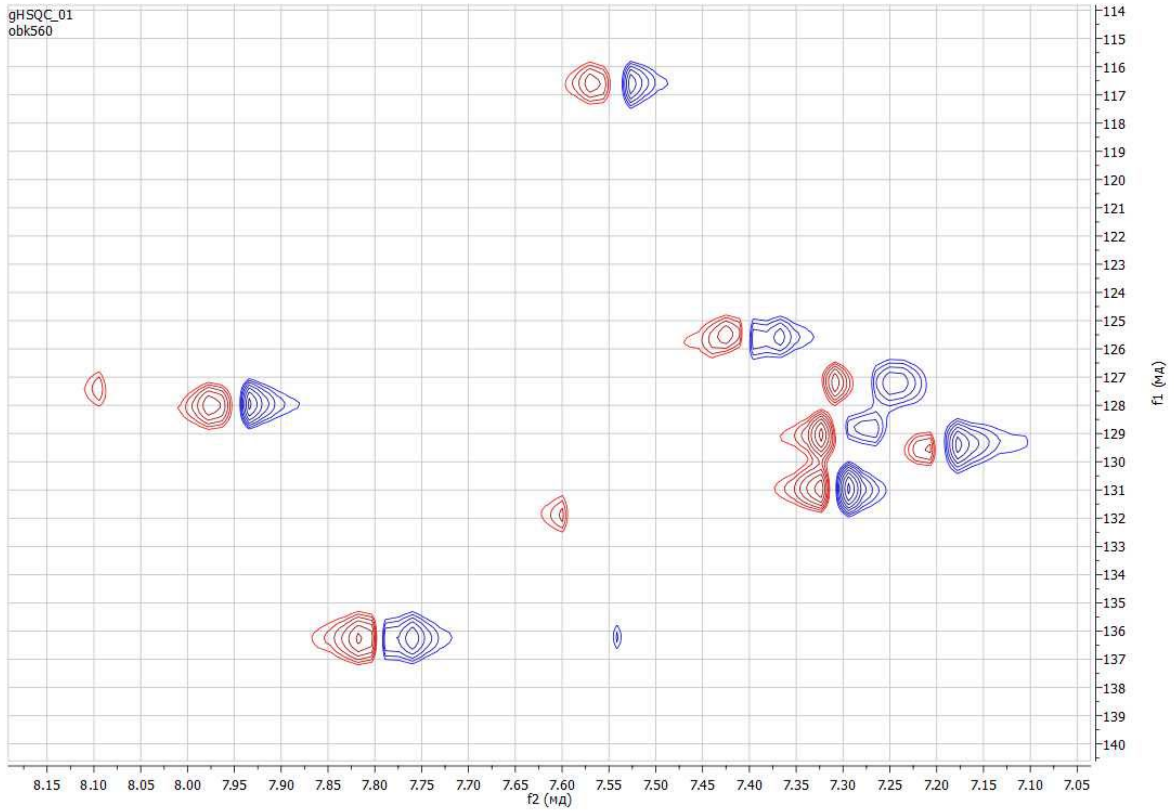

HSQC ^{1}H-^{13}C NMR spectra of compounds **3b** (DMSO-*d*6)

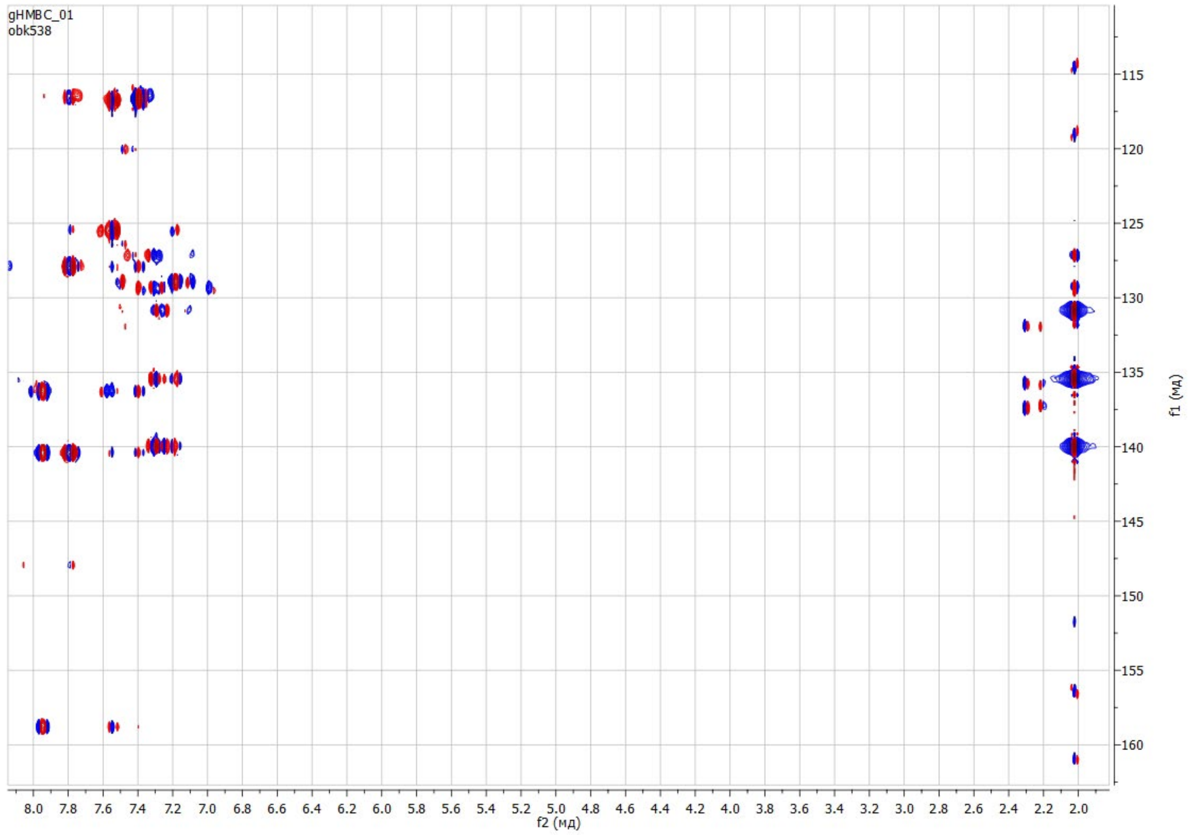

HMBC ^{1}H-^{13}C NMR spectra of compounds **3b** (DMSO-*d*6)

Display Report

Analysis Info

Analysis Name	C:\AOC2019\Osmanov\Nov_18\Nov_18_2019\bko-40_&clb.d	Acquisition Date	18.11.2019 14:18:14
Method	tune_wide.m	Operator	BDAL@DE
Sample Name	/CHIZ BKO-40	Instrument / Ser#	micrOTOF 10248
Comment	CH3CN 100 %, dil. 200, calibrant added		

Acquisition Parameter

Source Type	ESI	Ion Polarity	Positive	Set Nebulizer	0.4 Bar
Focus	Not active			Set Dry Heater	180 °C
Scan Begin	50 m/z	Set Capillary	4500 V	Set Dry Gas	4.0 l/min
Scan End	3000 m/z	Set End Plate Offset	-500 V	Set Divert Valve	Waste

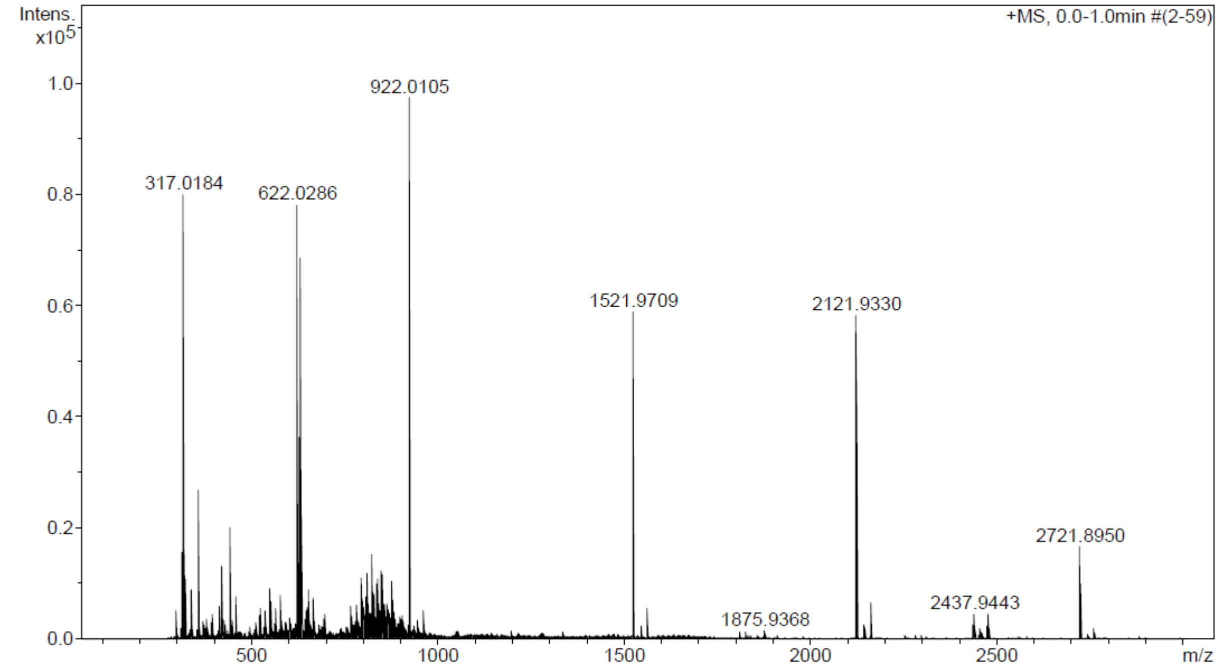

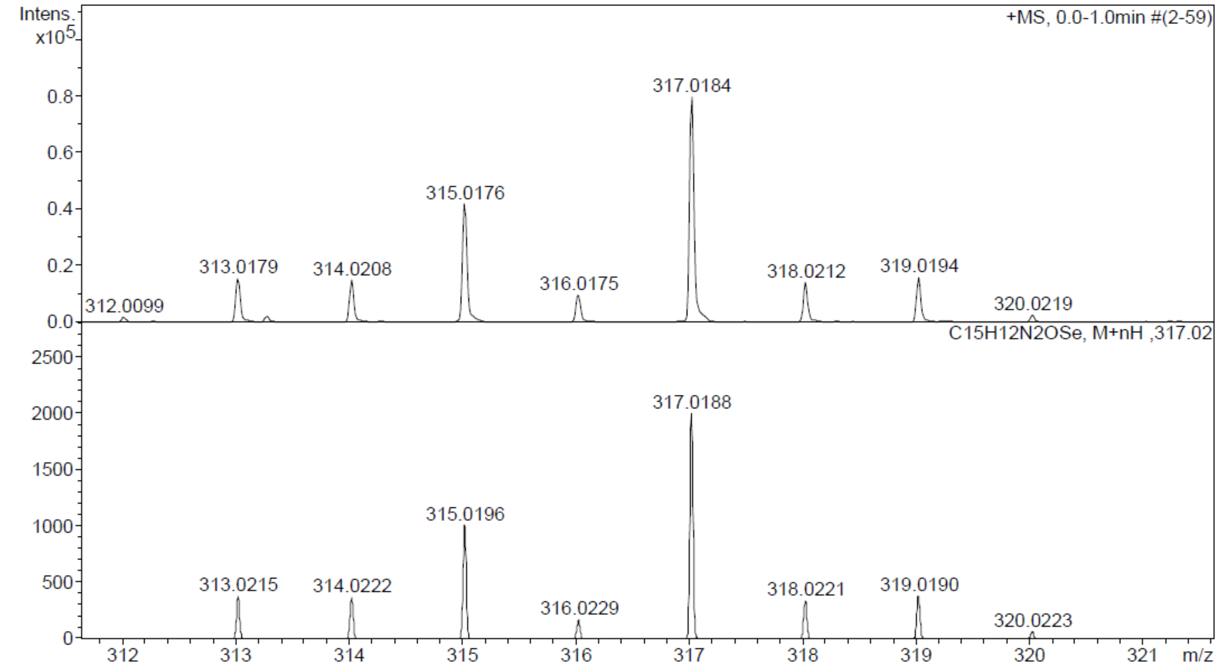

High resolution mass spectra (HR MS) of compounds **3b**

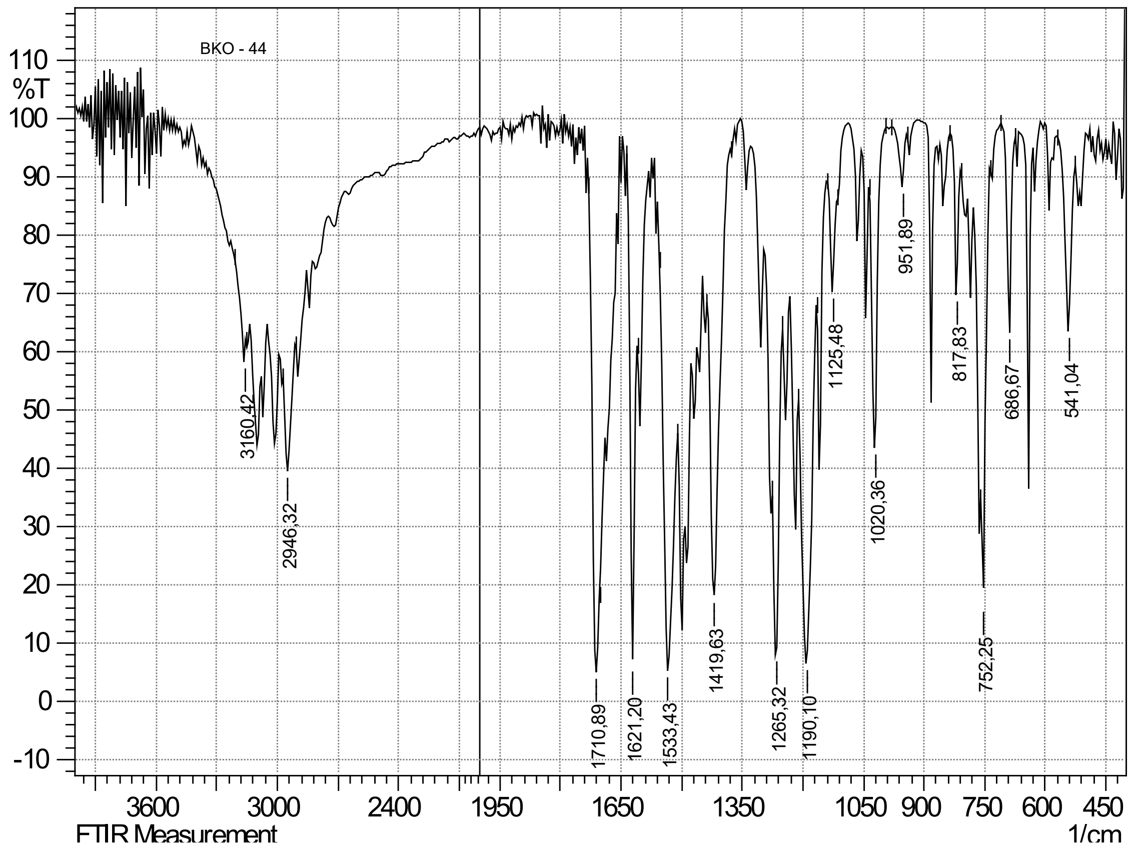

FTIR spectra of compounds **3c**

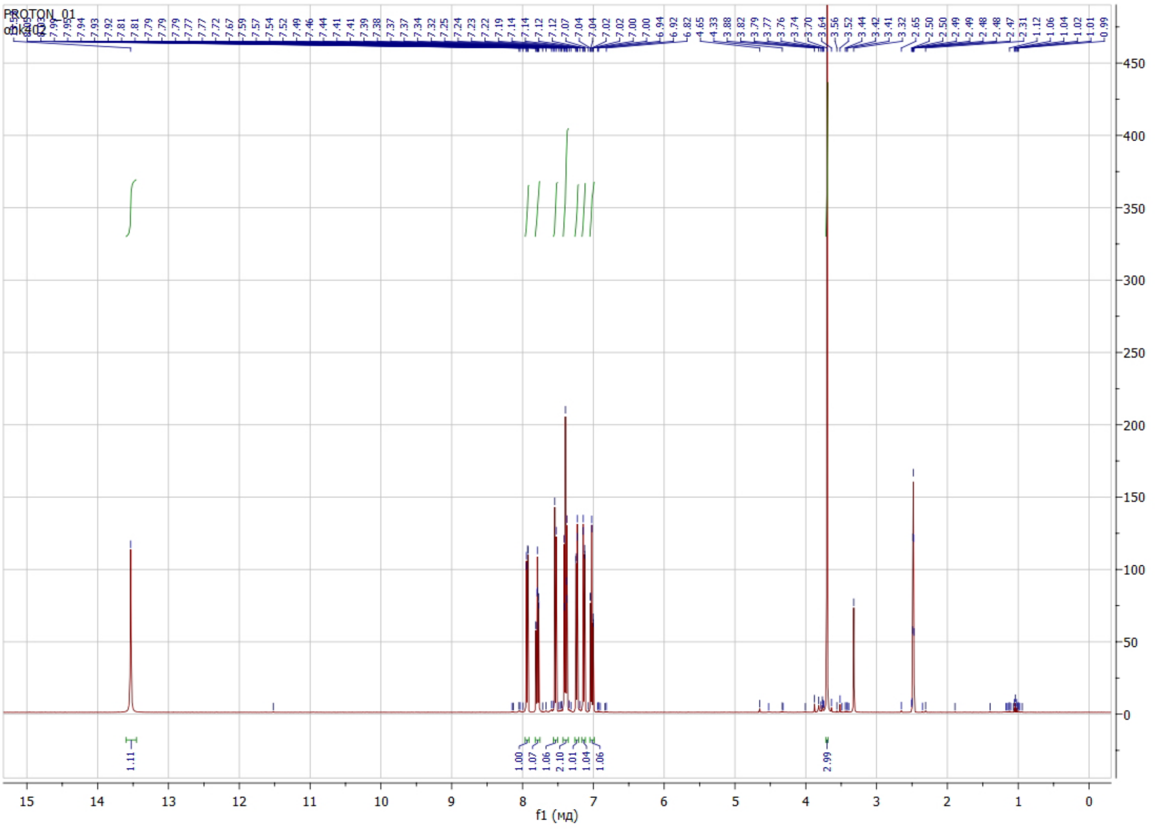

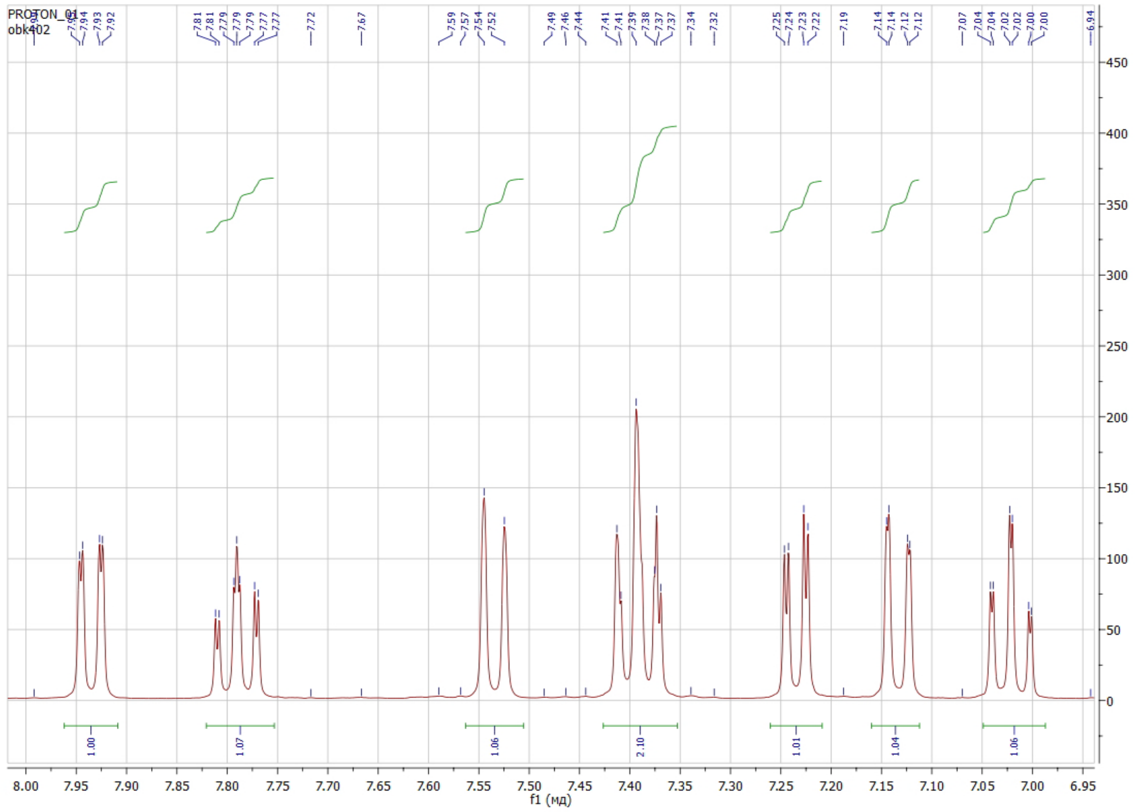

^{1}H NMR spectra of compounds **3c** (DMSO-*d*6)

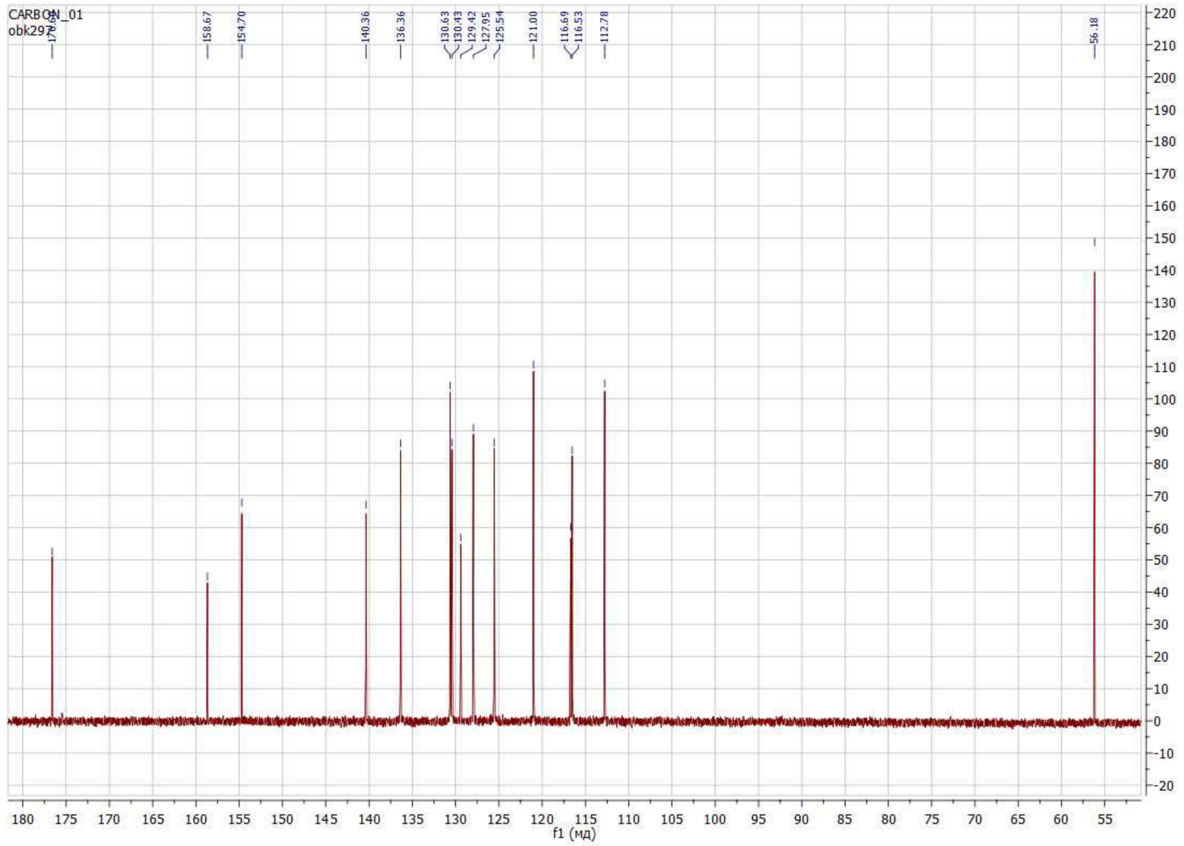

^{13}C NMR spectra of compounds **3c** (DMSO-*d*6)

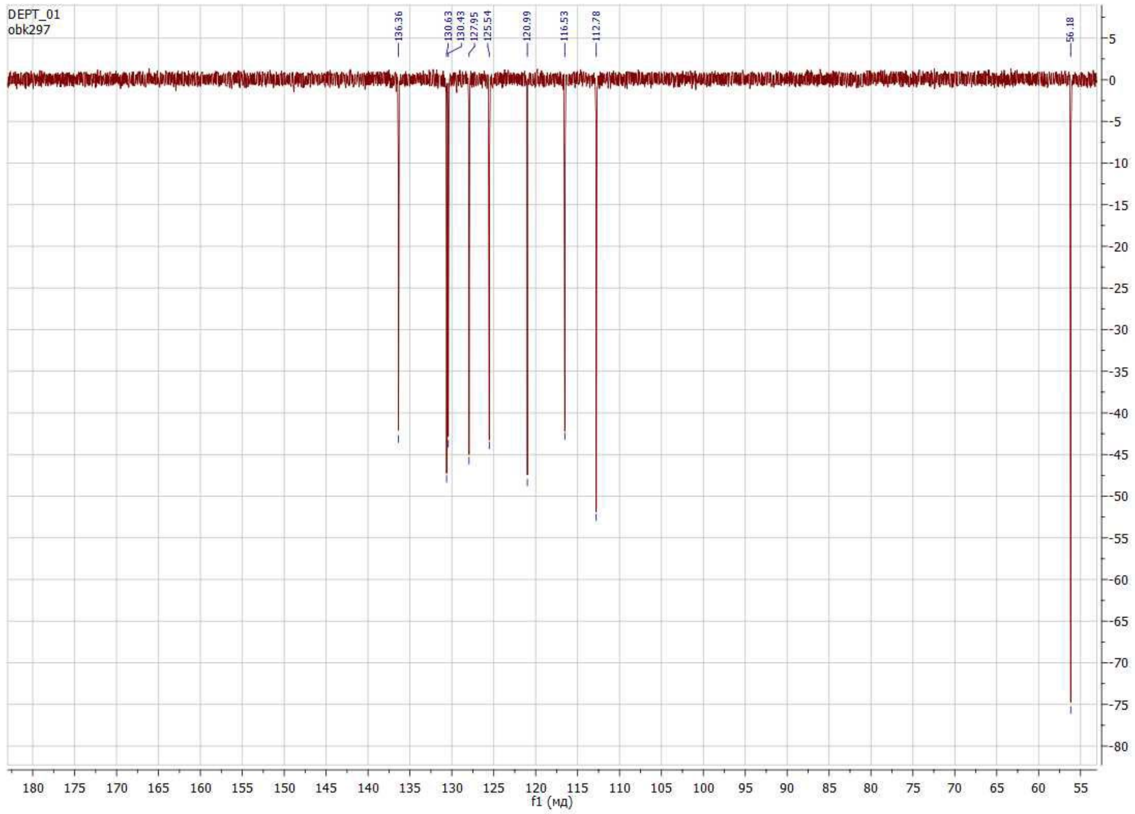

^{13}C DEPT NMR spectra of compounds **3c** (DMSO-*d*6)

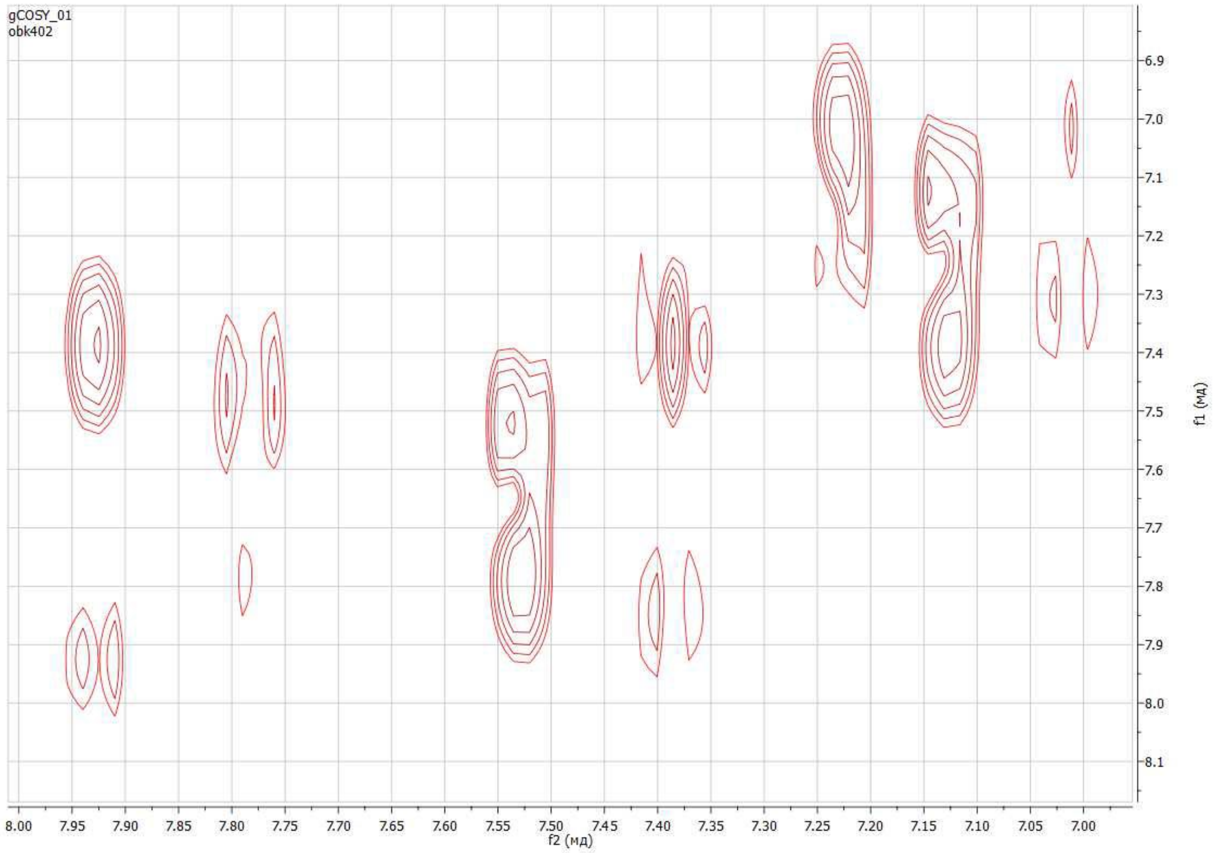

COSY NMR spectra of compounds **3c** (DMSO-*d*6)

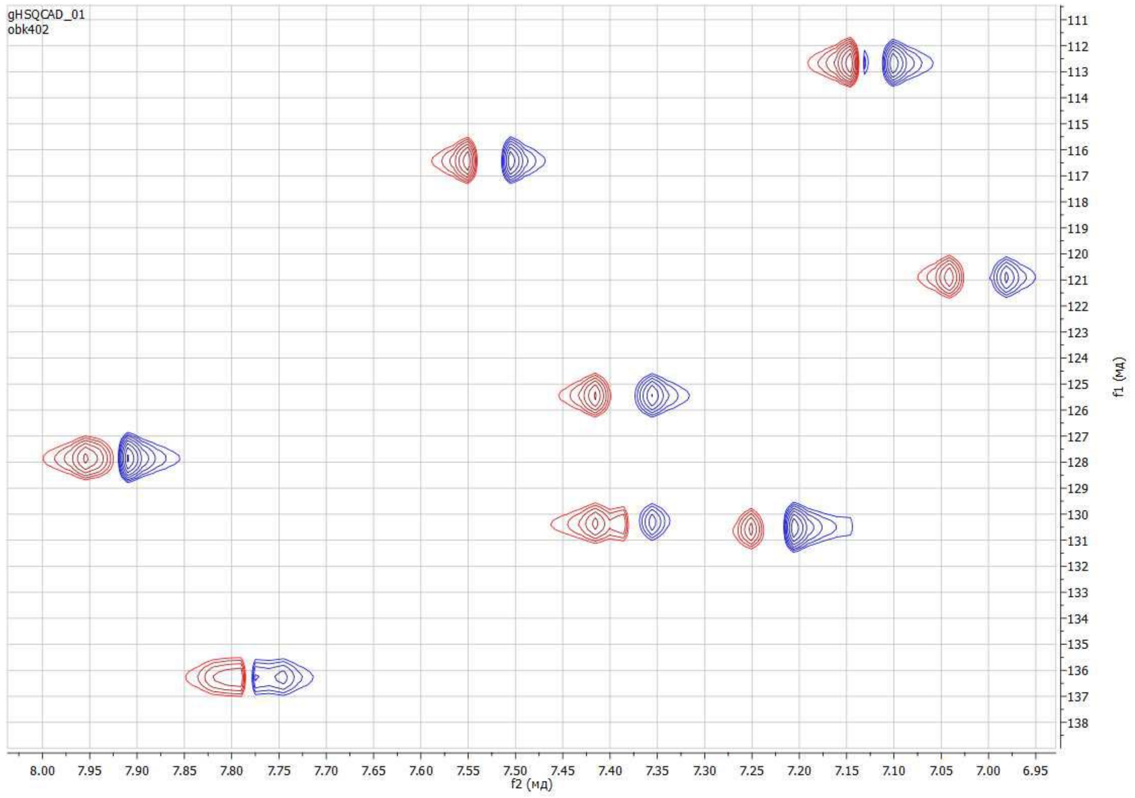

HSQC ^{1}H-^{13}C NMR spectra of compounds **3c** (DMSO-*d*6)

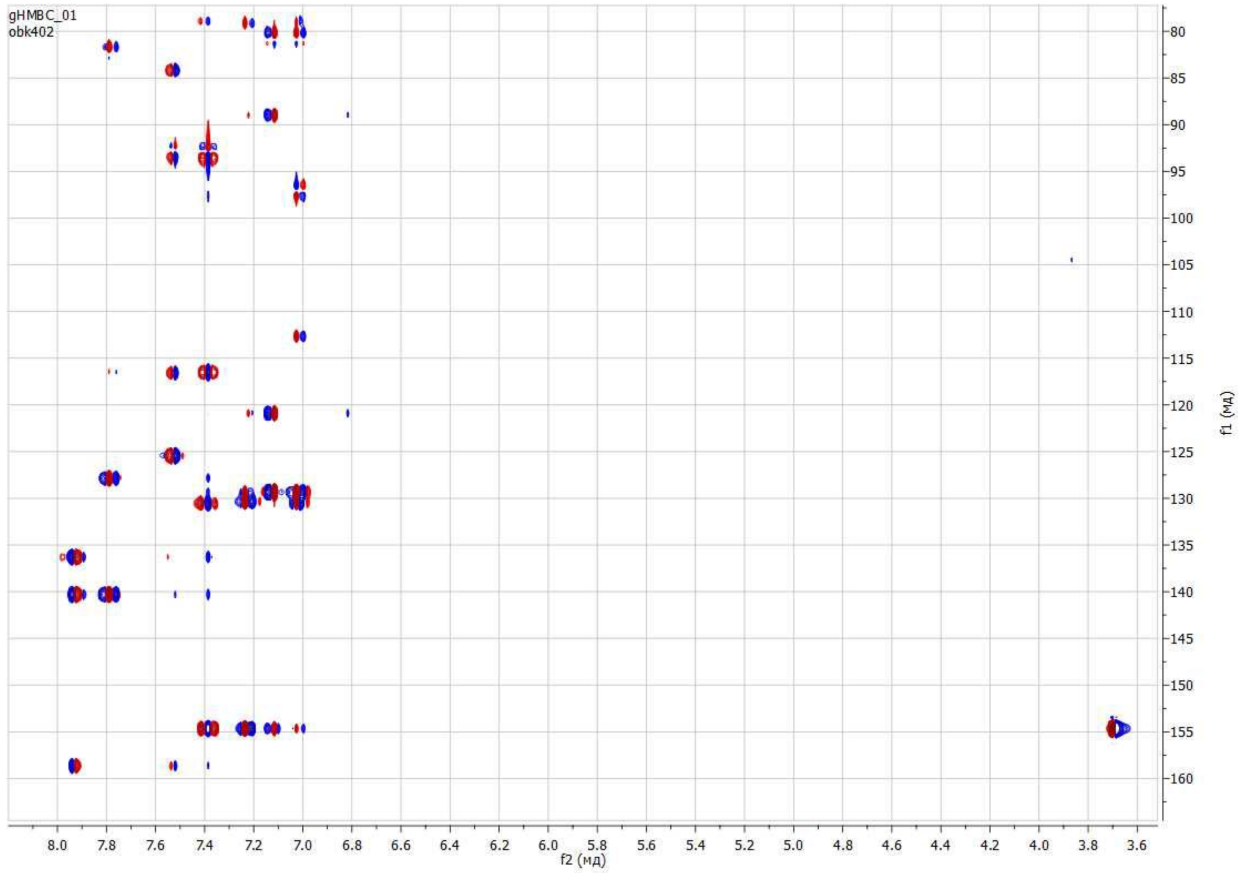

HMBC ^{1}H-^{13}C NMR spectra of compounds **3c** (DMSO-*d*6)

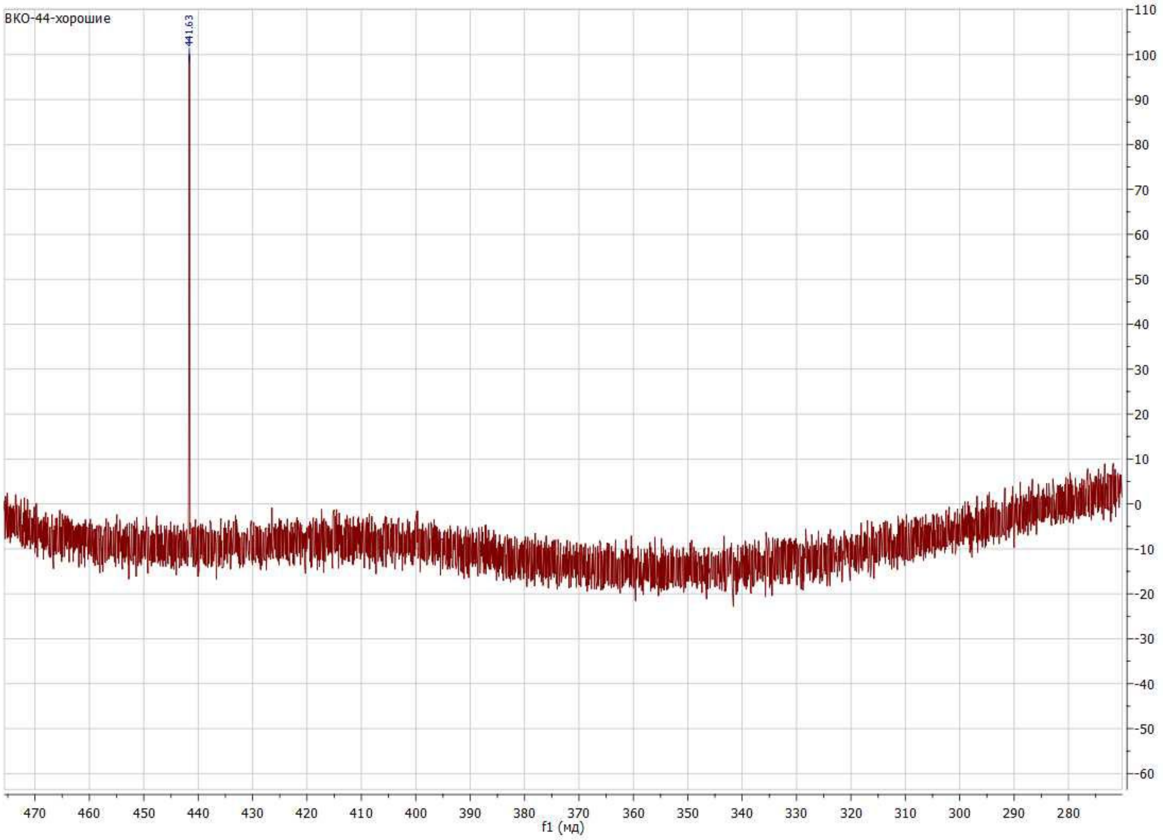

^{77}Se NMR spectra of compounds **3c** (DMSO-*d*6)

Display Report

Analysis Info

Analysis Name	C:\AOC2019\Osmanov\Nov_18\Nov_18_2019\bko-44_&clblow.d	Acquisition Date	18.11.2019 14:43:07
Method	tune_low.m	Operator	BDAL@DE
Sample Name	/CHIZ BKO-40	Instrument / Ser#	micrOTOF 10248
Comment	CH3CN 100 %, dil. 200, calibrant added		

Acquisition Parameter

Source Type	ESI	Ion Polarity	Positive	Set Nebulizer	0.4 Bar
Focus	Not active			Set Dry Heater	180 °C
Scan Begin	50 m/z	Set Capillary	4500 V	Set Dry Gas	4.0 l/min
Scan End	3000 m/z	Set End Plate Offset	-500 V	Set Divert Valve	Waste

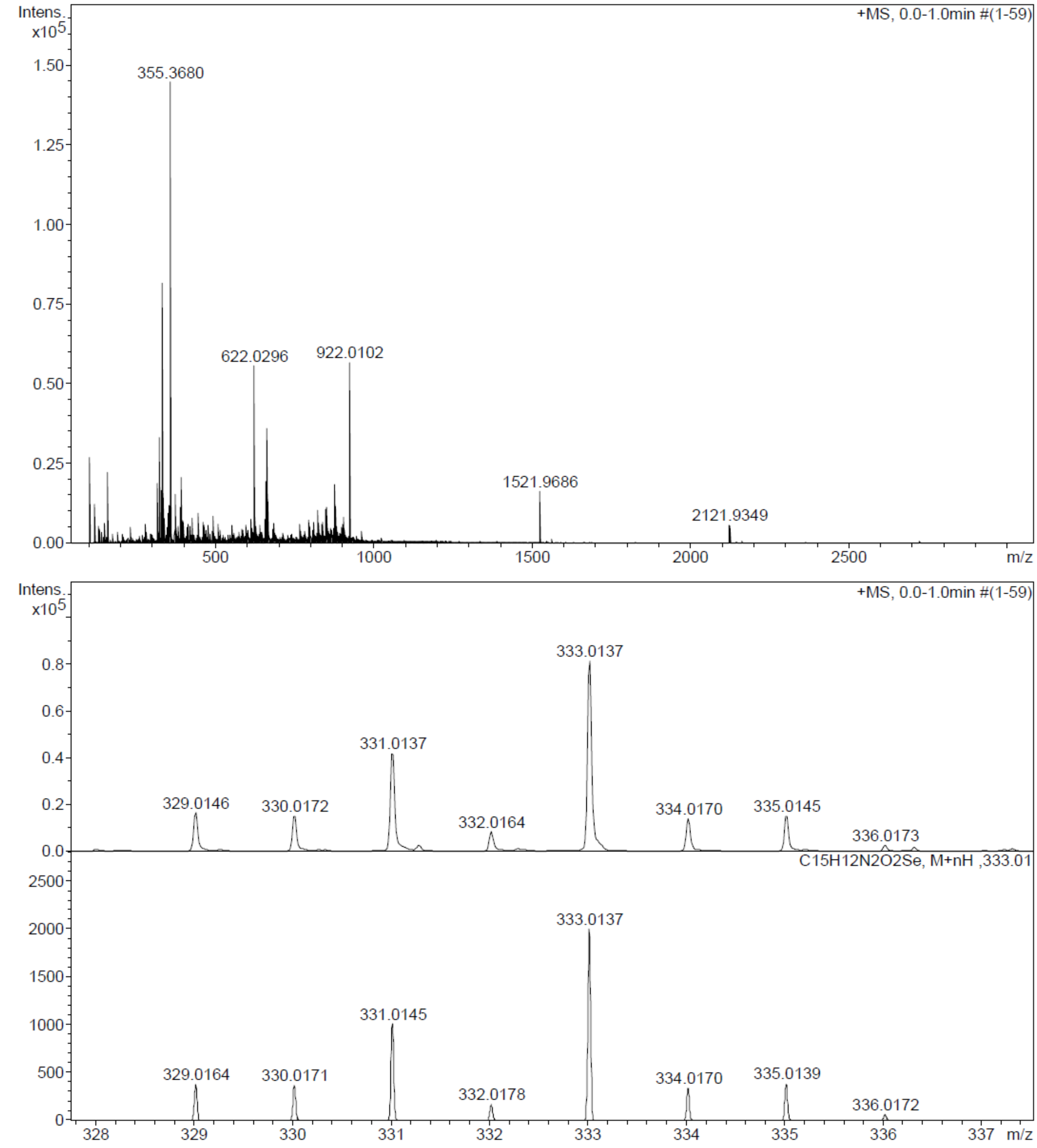

High resolution mass spectra (HR MS) of compounds **3c**

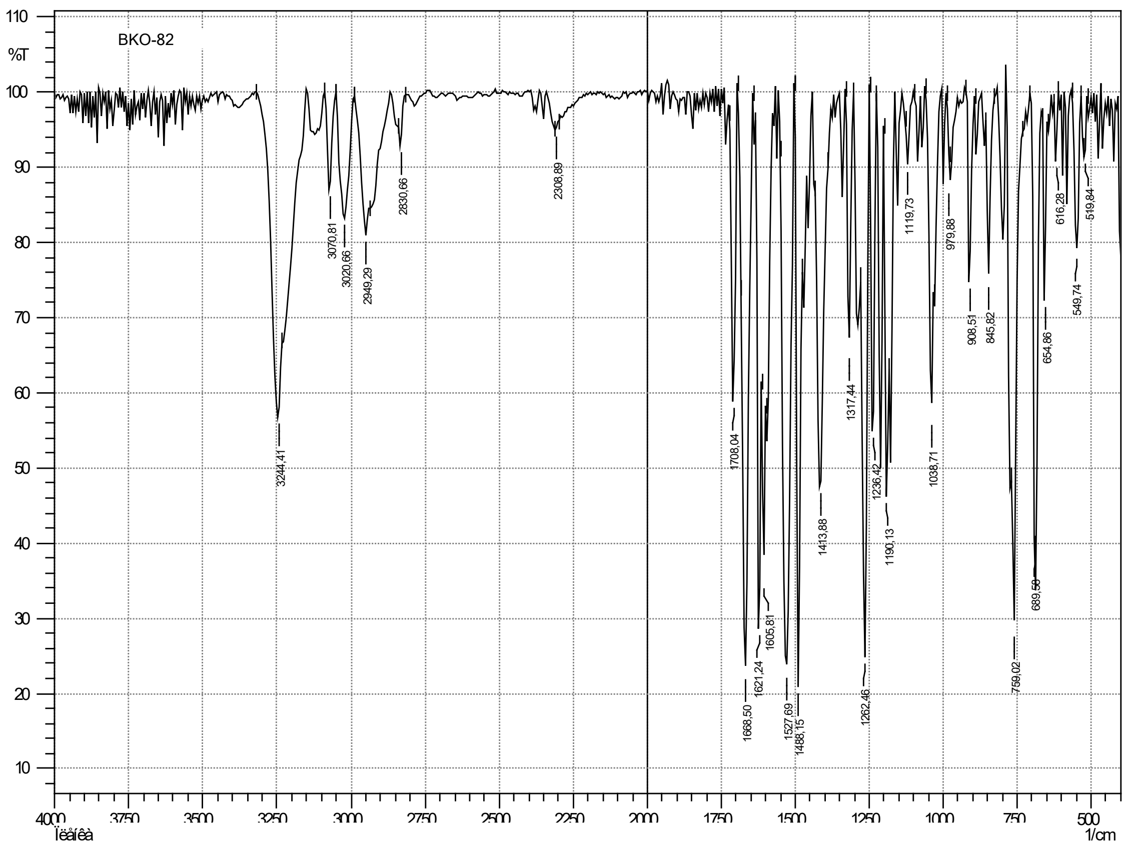

FTIR spectra of compounds **3d**

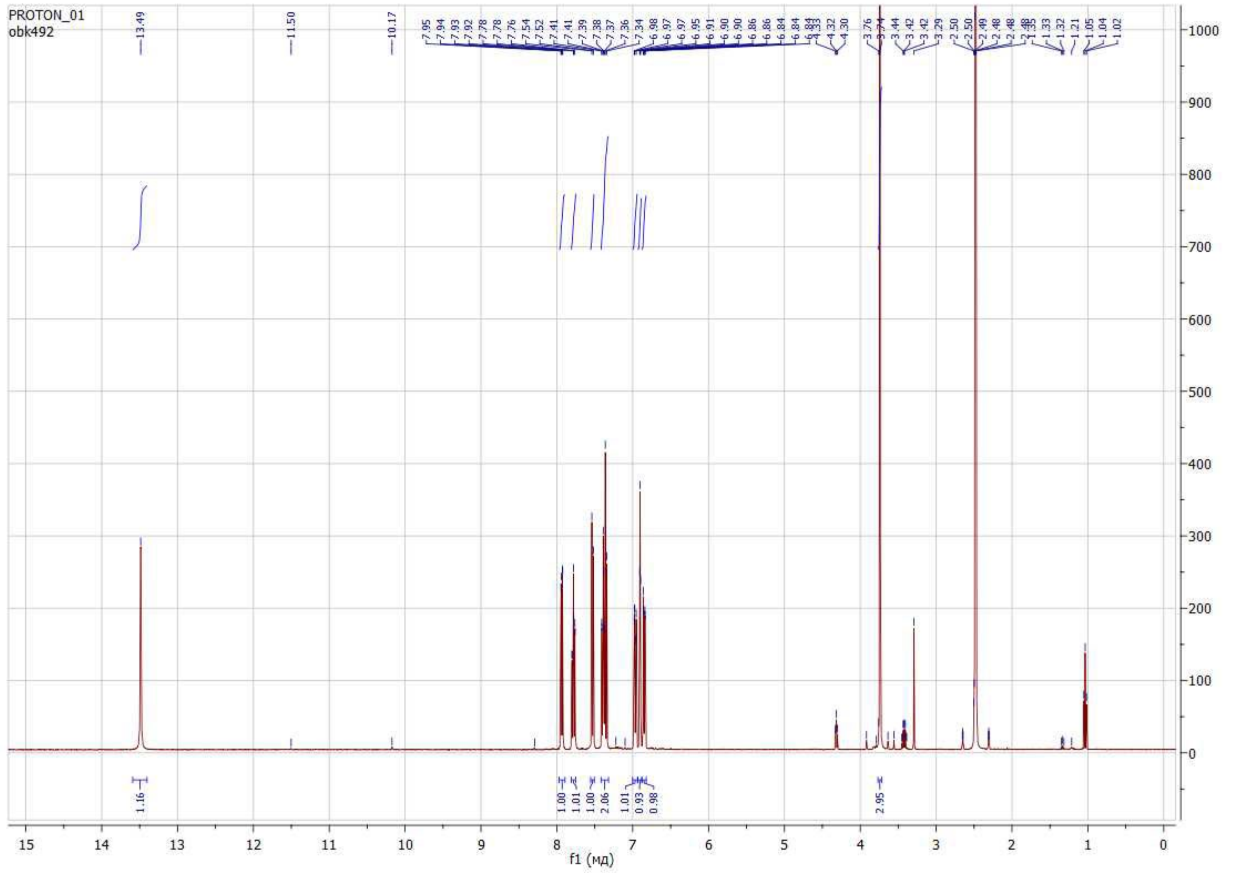

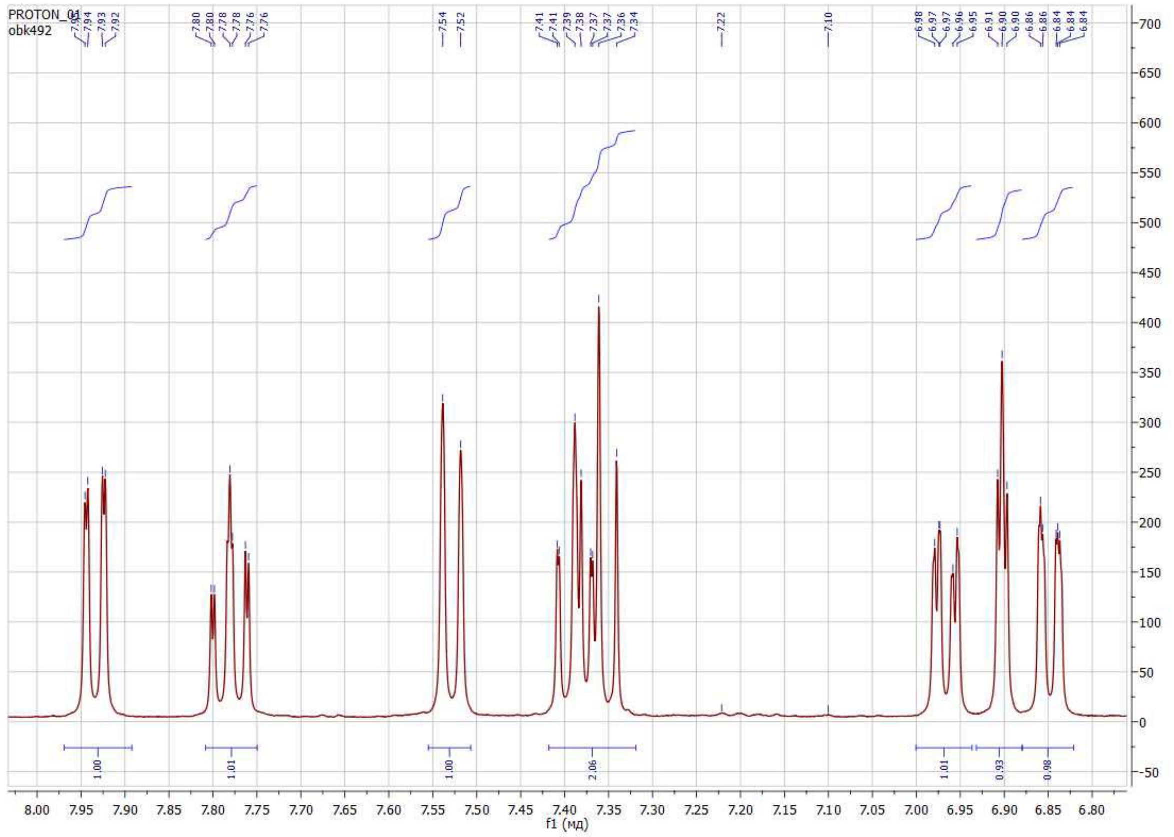

^{1}H NMR spectra of compounds **3d** (DMSO-*d*6)

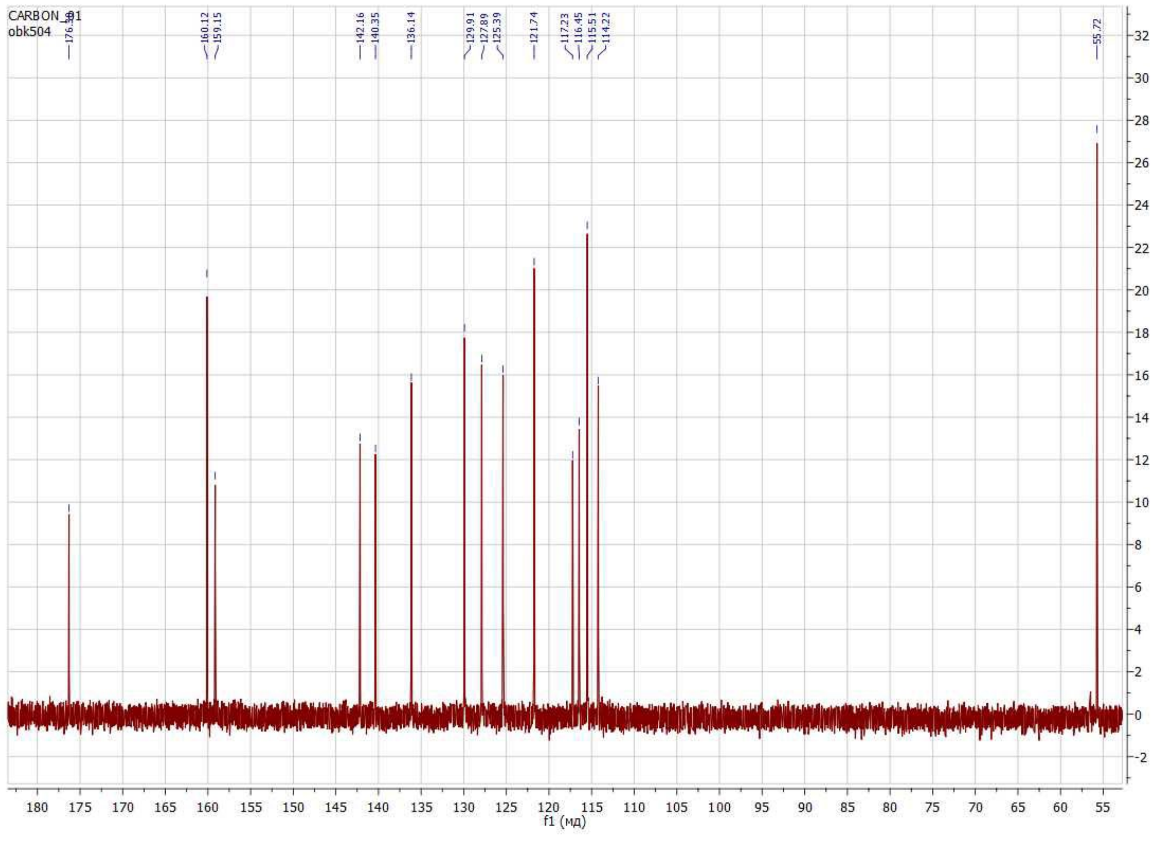

^{13}C NMR spectra of compounds **3d** (DMSO-*d*6)

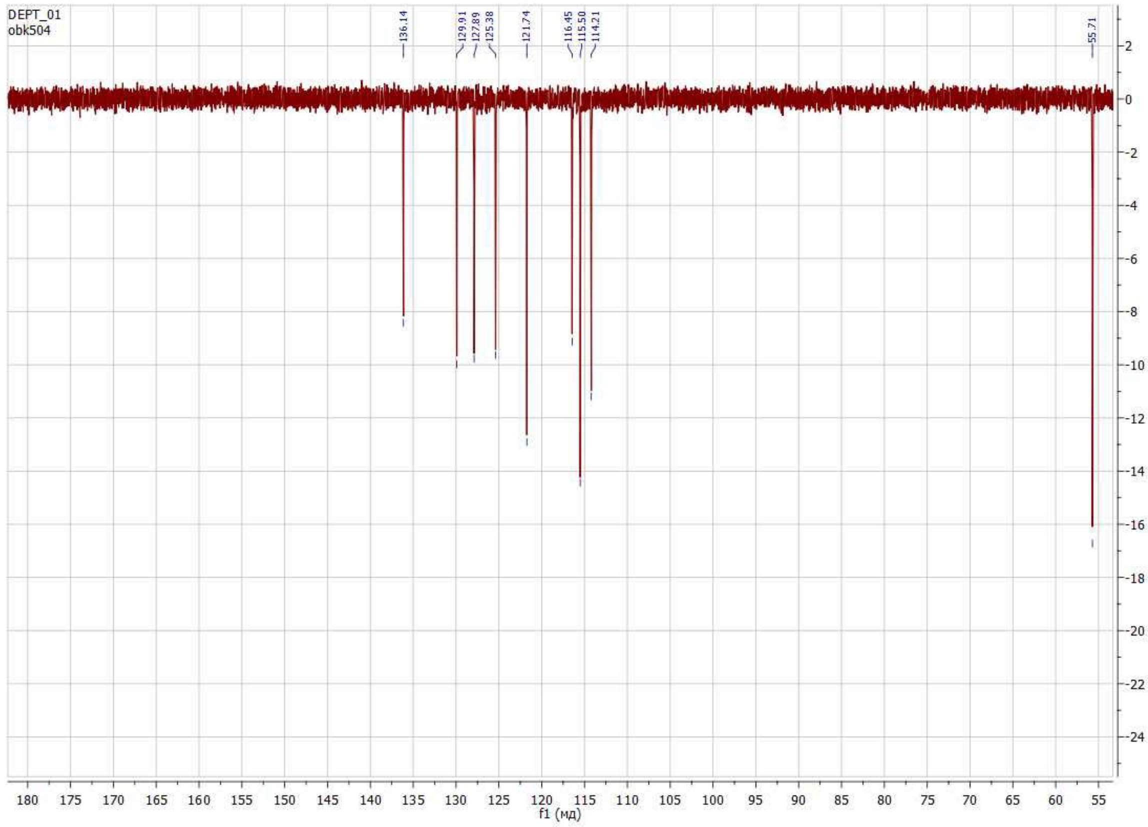

^{13}C DEPT NMR spectra of compounds **3d** (DMSO-*d*6)

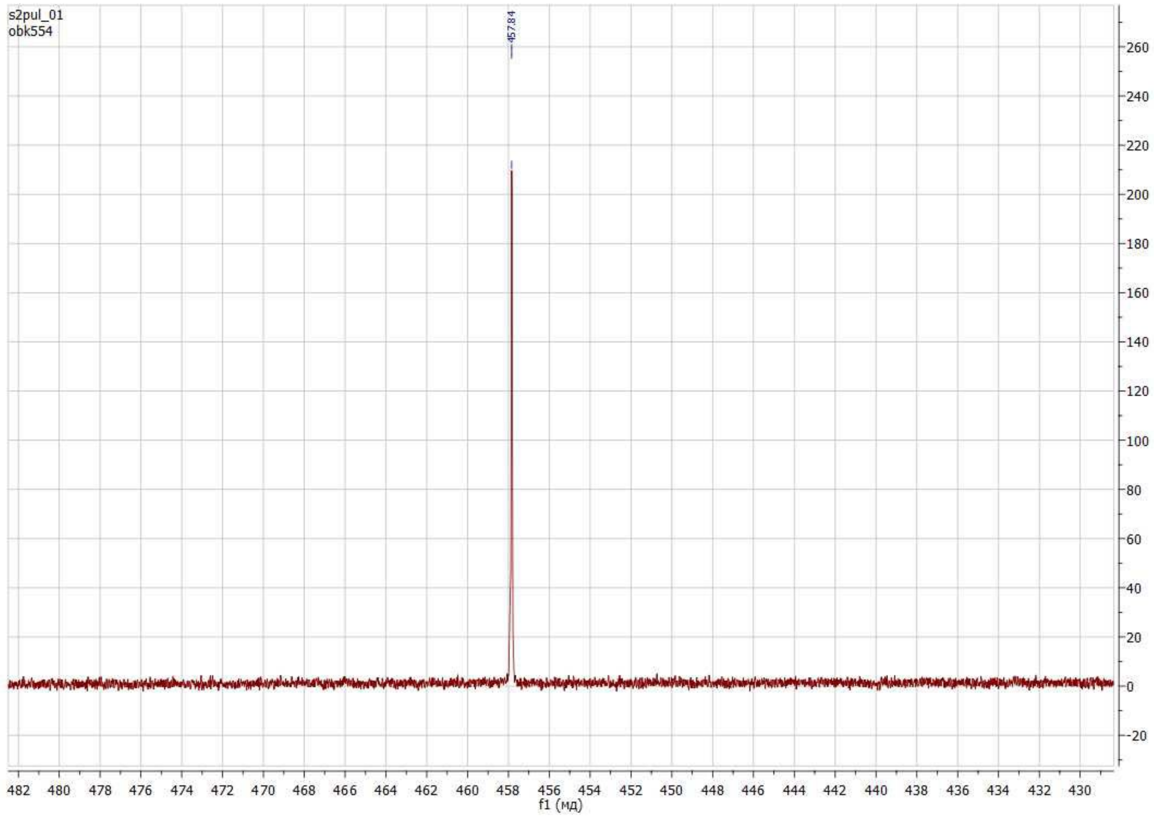

^{77}Se NMR spectra of compounds **3d** (DMSO-*d*6)

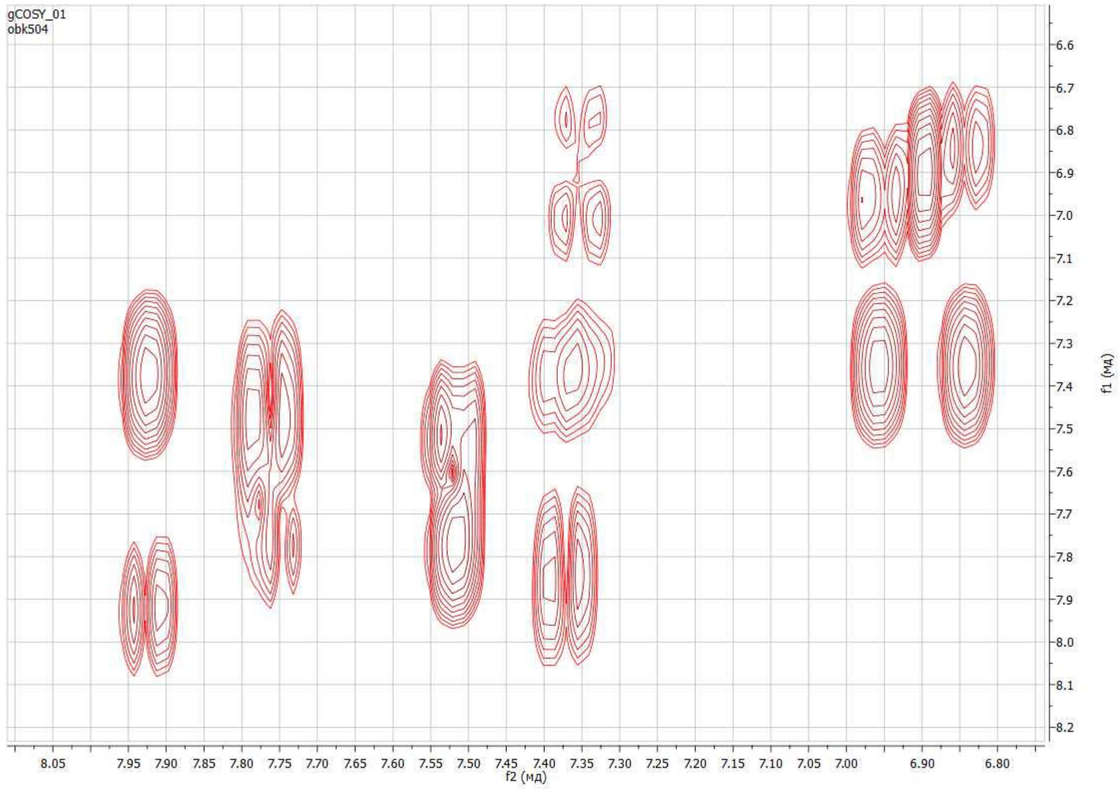

COSY NMR spectra of compounds **3d** (DMSO-*d*6)

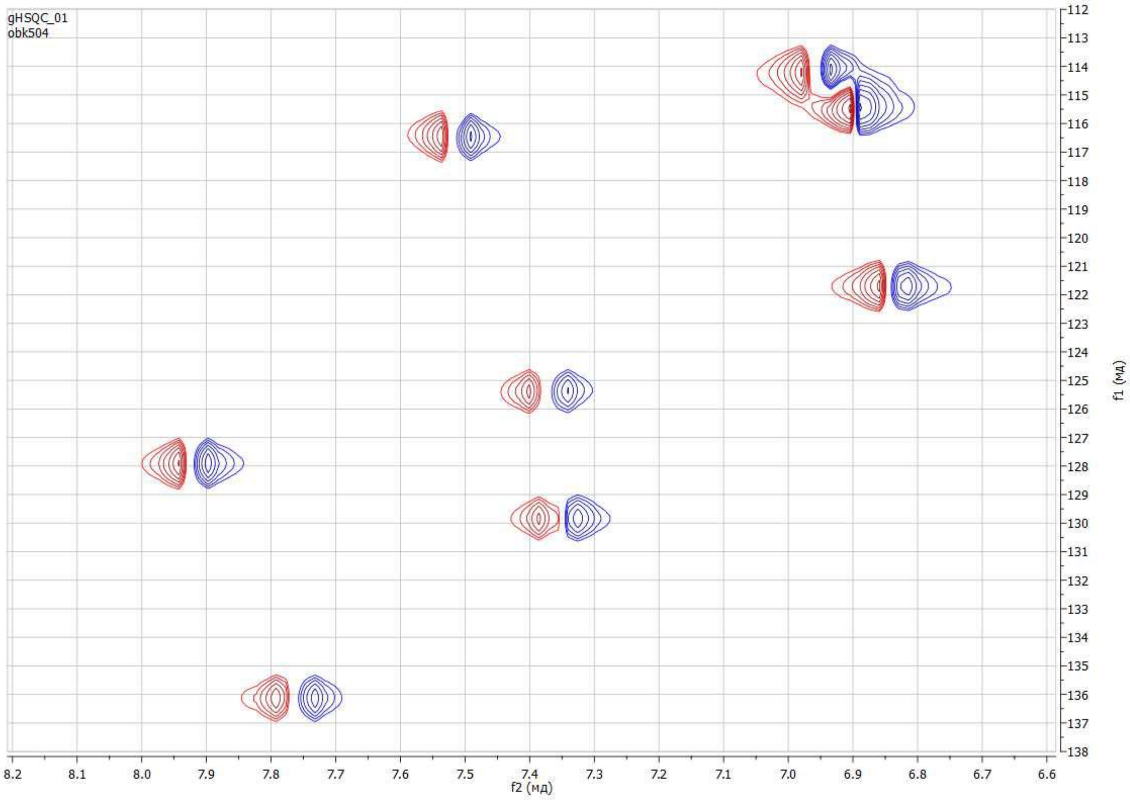

HSQC ^{1}H-^{13}C NMR spectra of compounds **3d** (DMSO-*d*6)

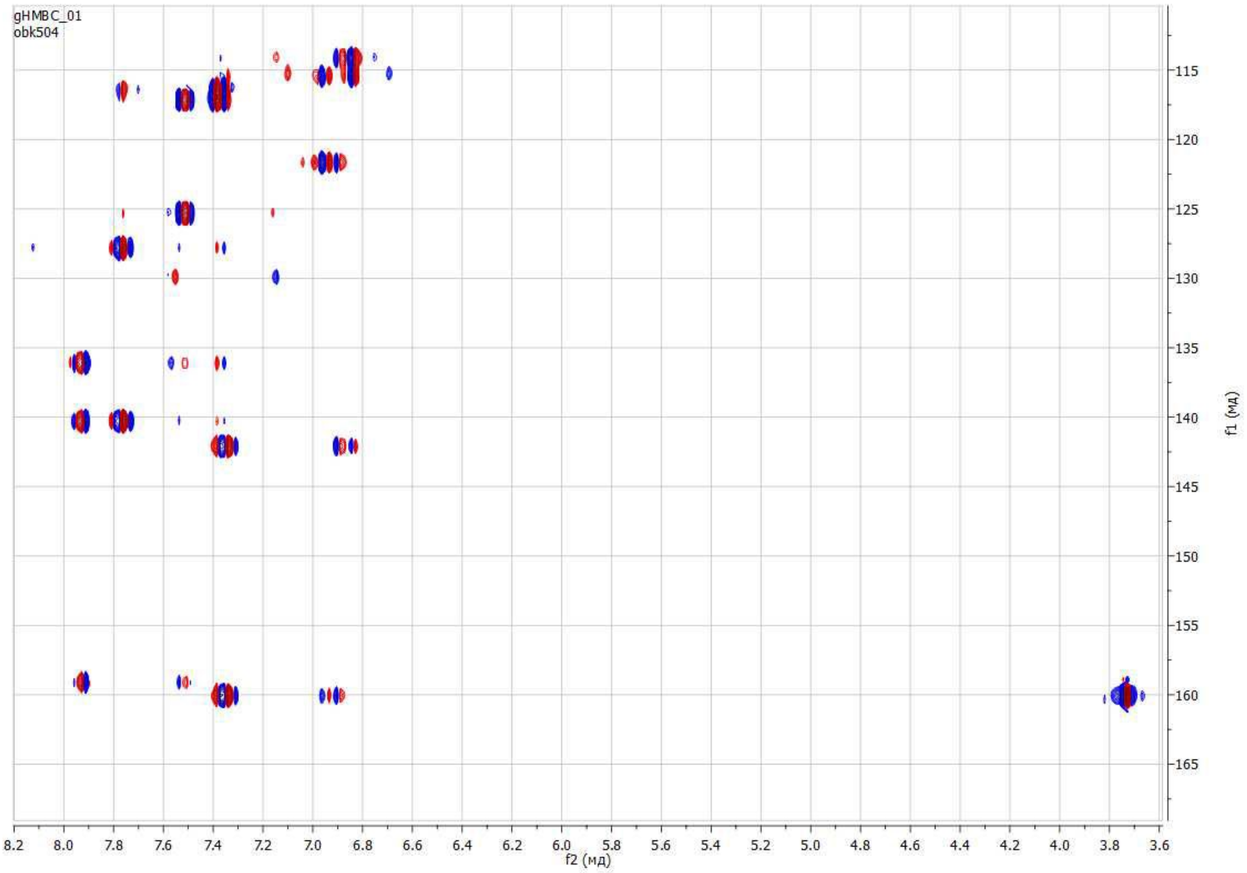

HMBC ^{1}H-^{13}C NMR spectra of compounds **3d** (DMSO-*d*6)

Display Report

Analysis Info

Analysis Name	D:\Data\Chizhov\Osmanov\Mar_15_2021\bko-82_&clblow.d	Acquisition Date	15.03.2021 15:10:07
Method	tune_low.m	Operator	BDAL@DE
Sample Name	/CHIZ BKO-82	Instrument / Ser#	micrOTOF 10248
Comment	CH3CN 100 %, dil. 200, calibrant added		

Acquisition Parameter

Source Type	ESI	Ion Polarity	Positive	Set Nebulizer	0.4 Bar
Focus	Not active			Set Dry Heater	180 °C
Scan Begin	50 m/z	Set Capillary	4500 V	Set Dry Gas	4.0 l/min
Scan End	3000 m/z	Set End Plate Offset	-500 V	Set Divert Valve	Waste

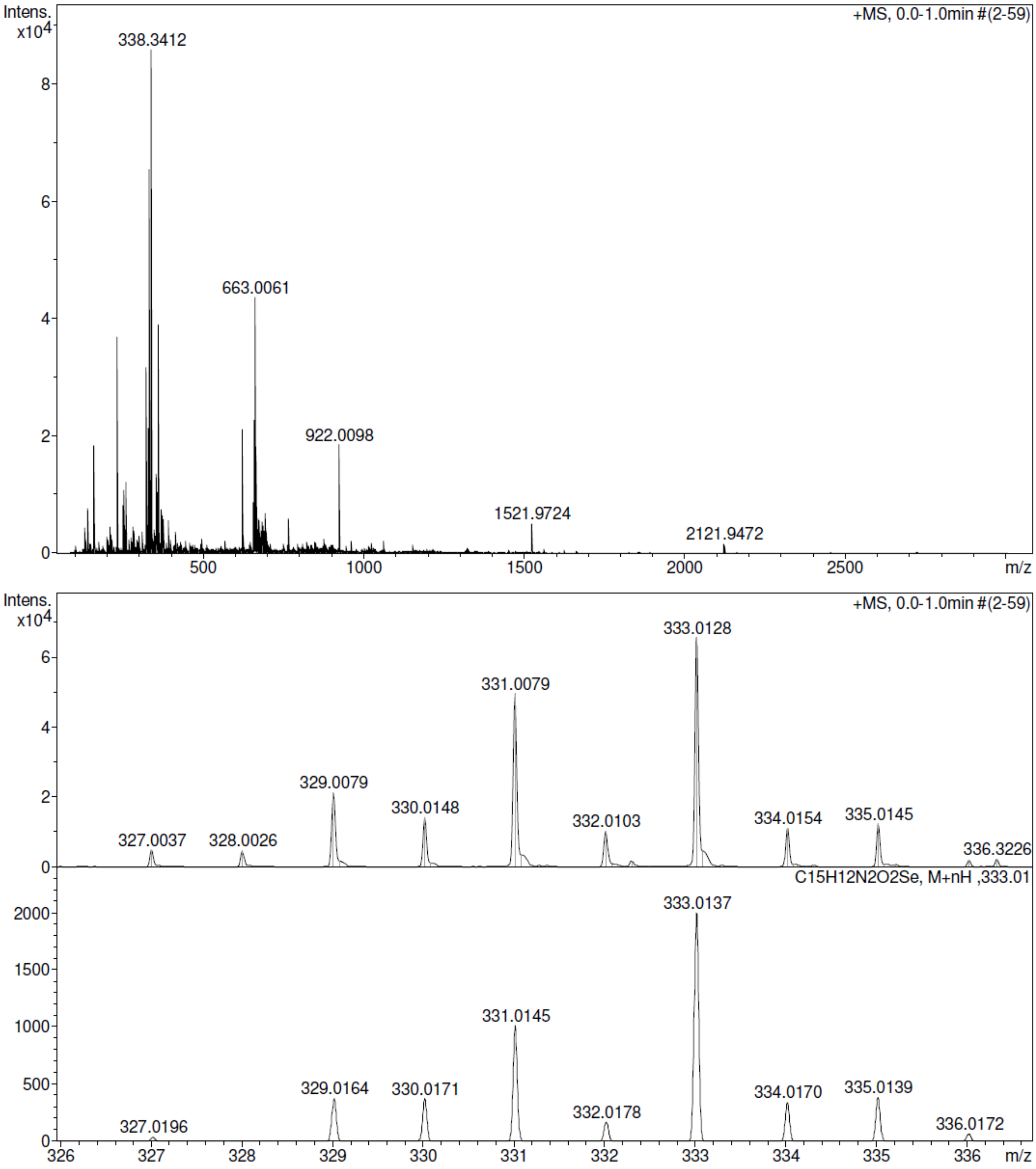

High resolution mass spectra (HR MS) of compounds **3d**

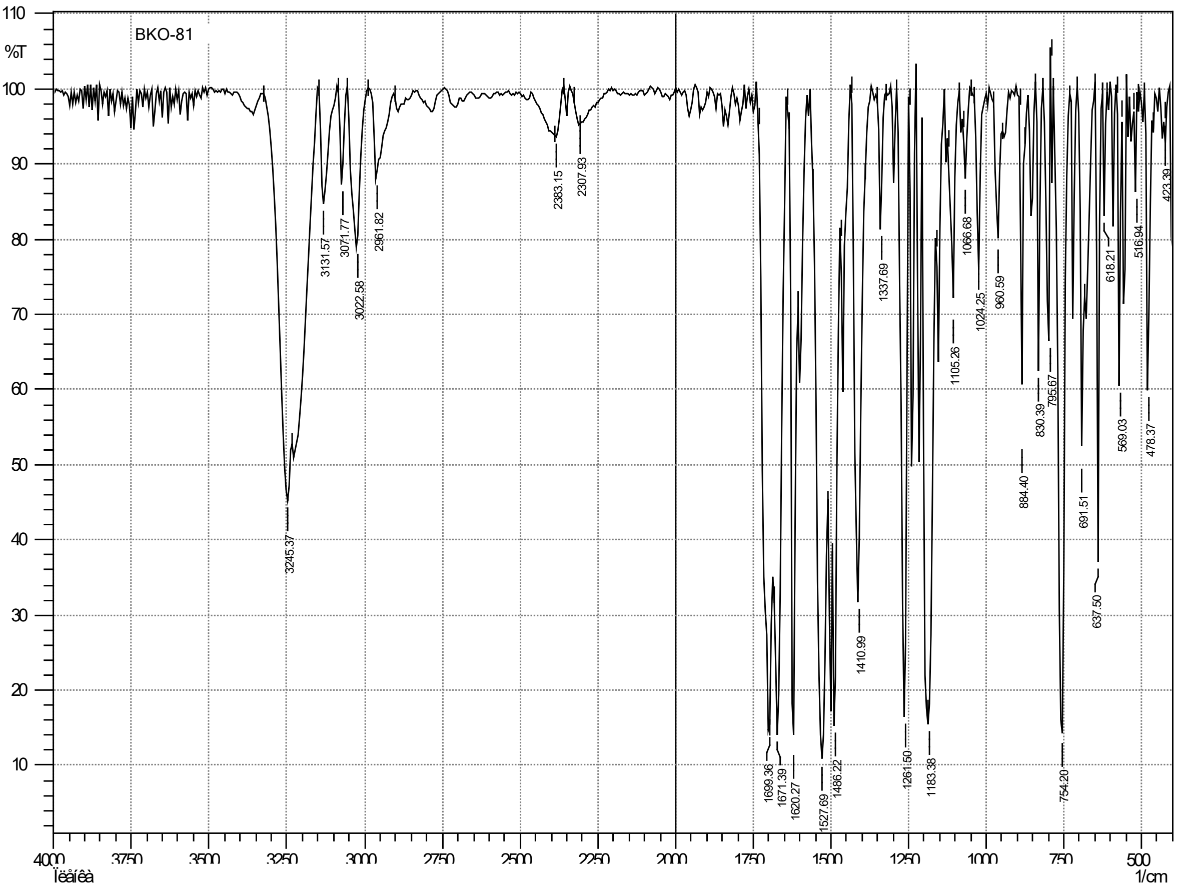

FTIR spectra of compounds **3e**

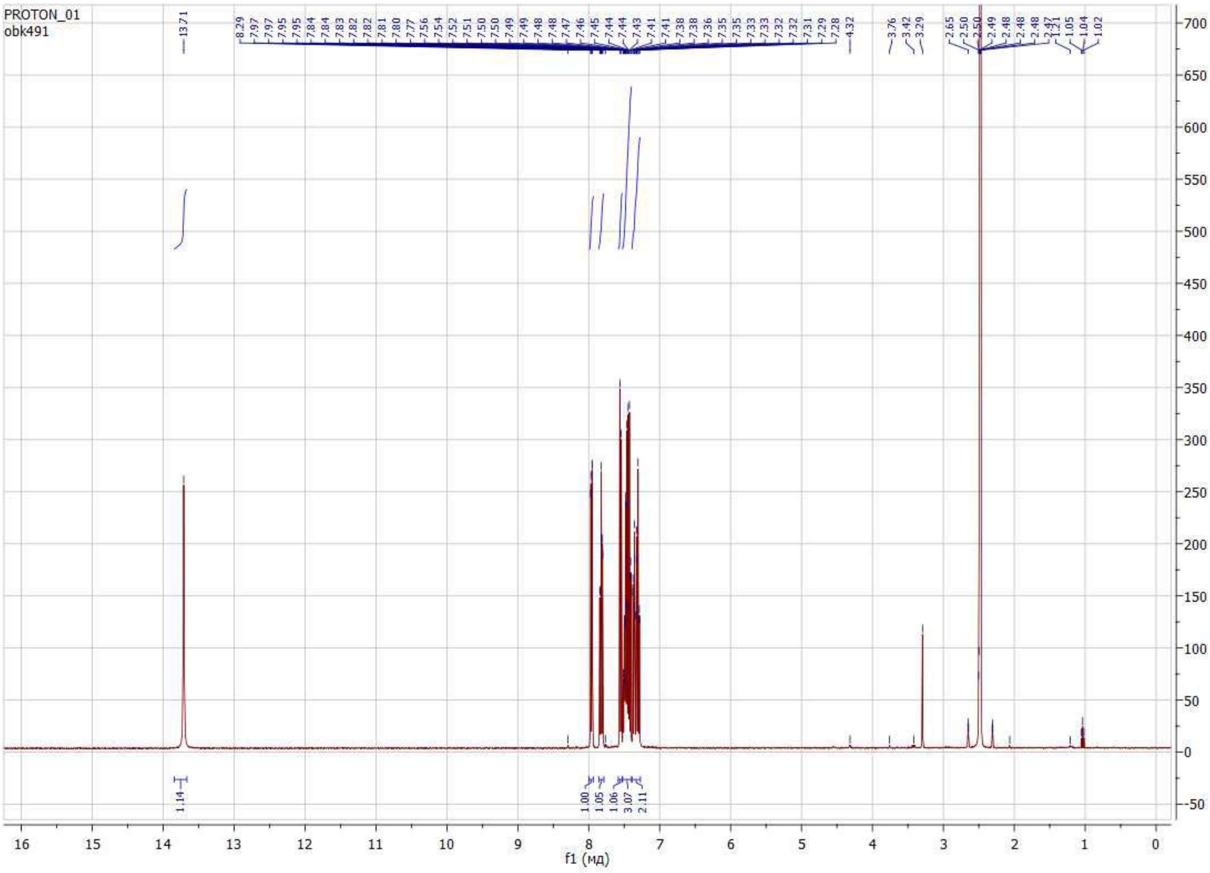

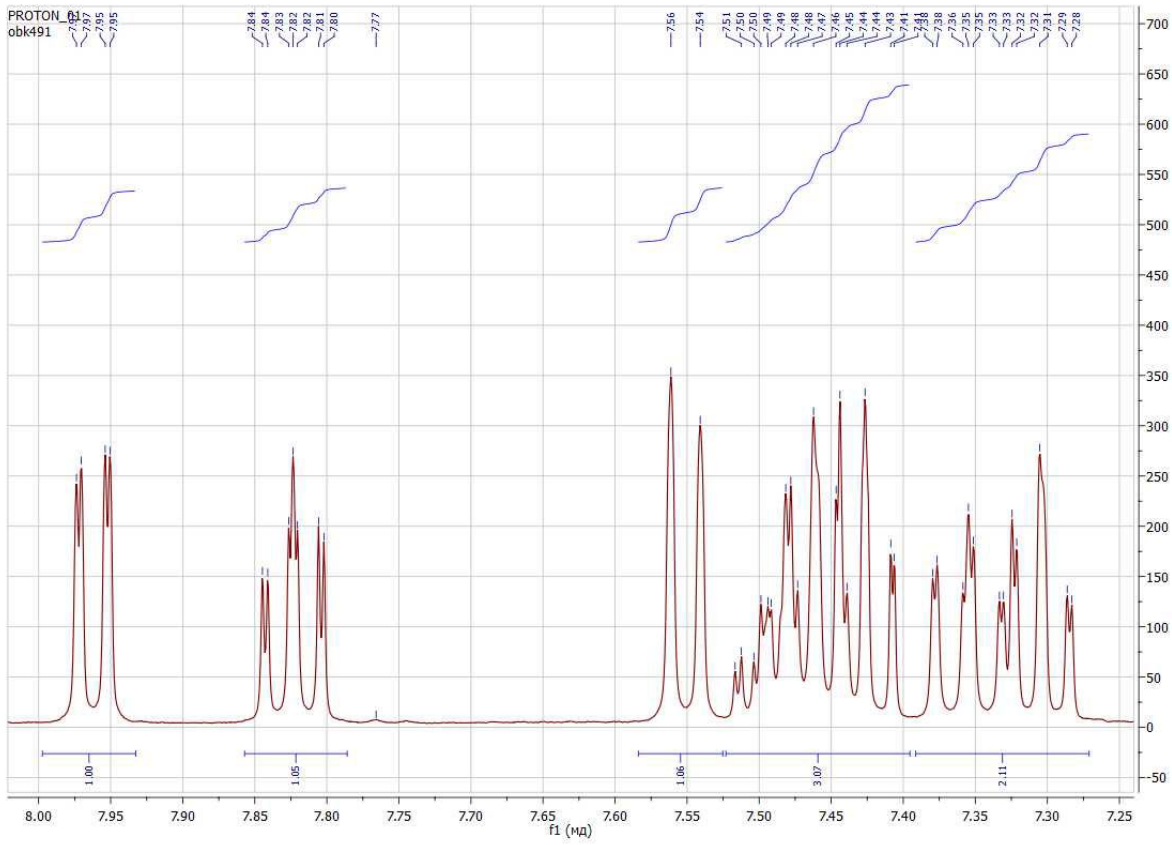

^{1}H NMR spectra of compounds **3e** (DMSO-*d*6)

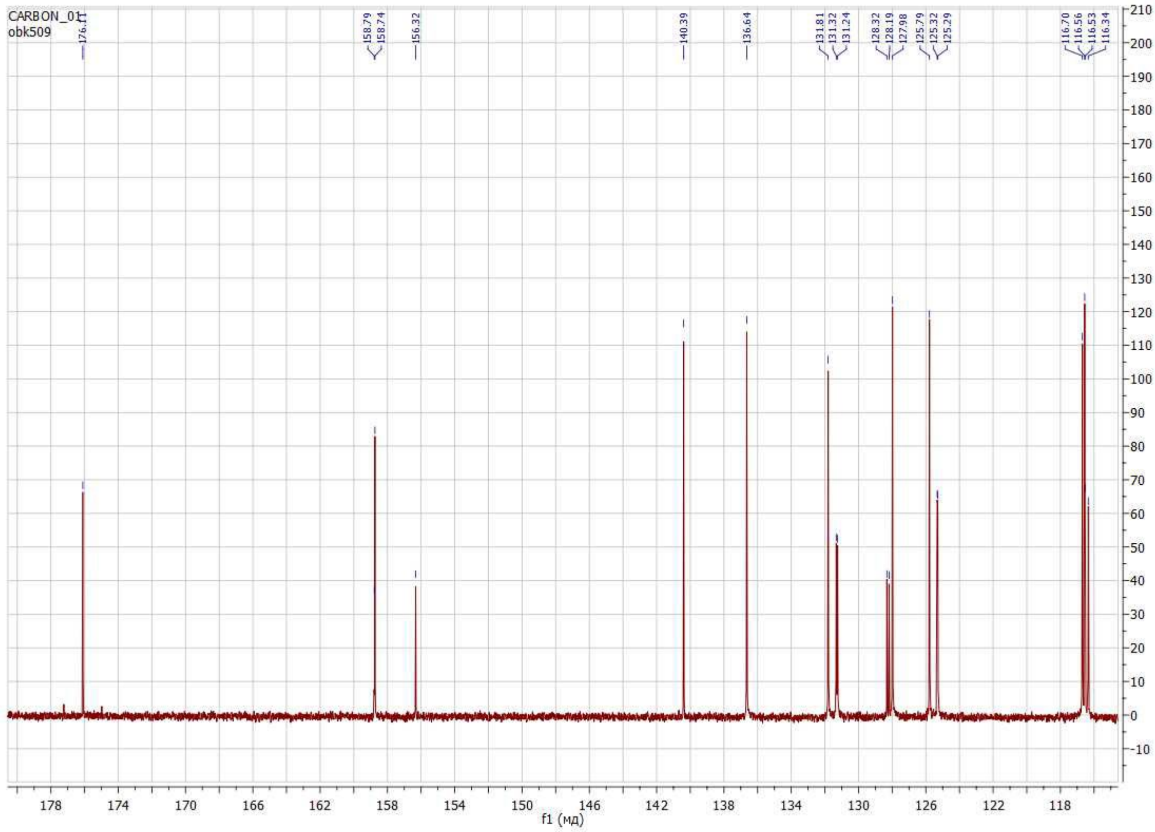

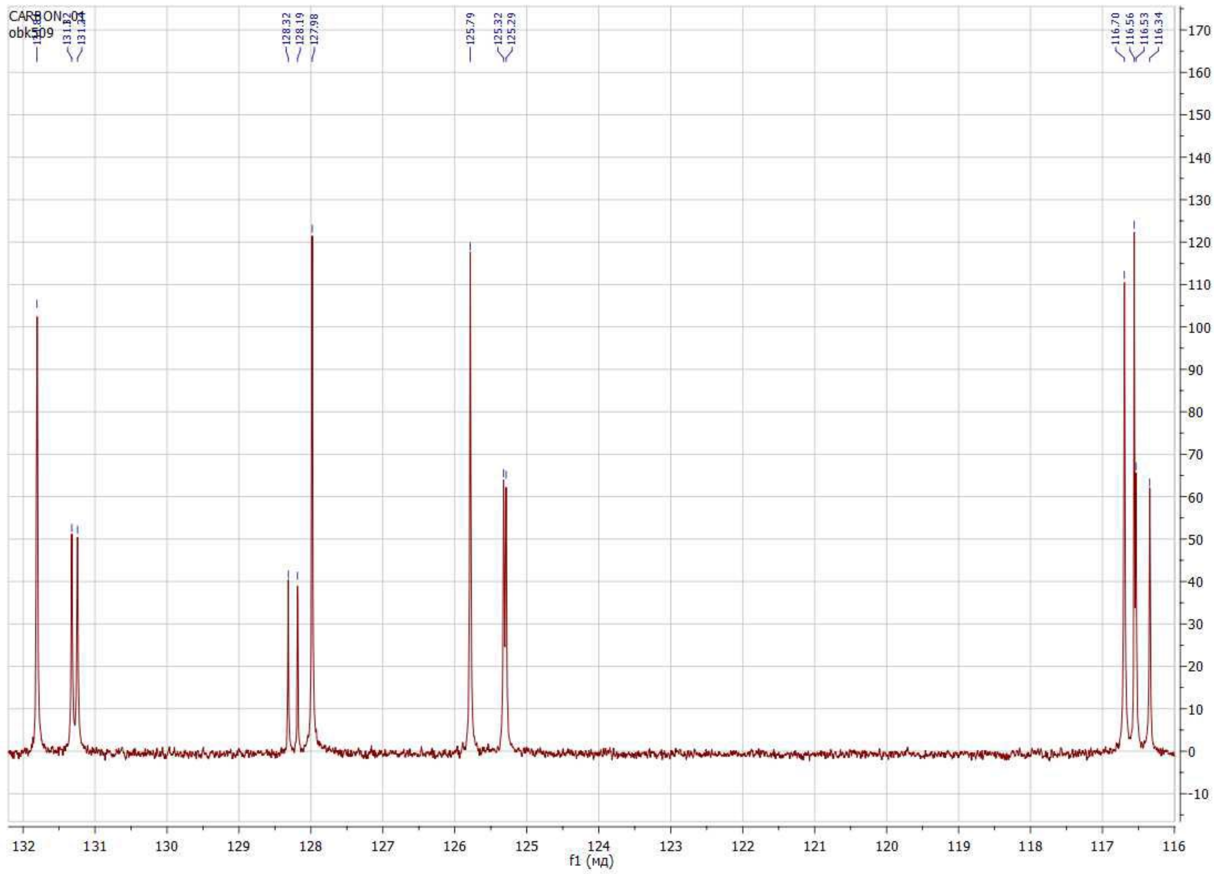

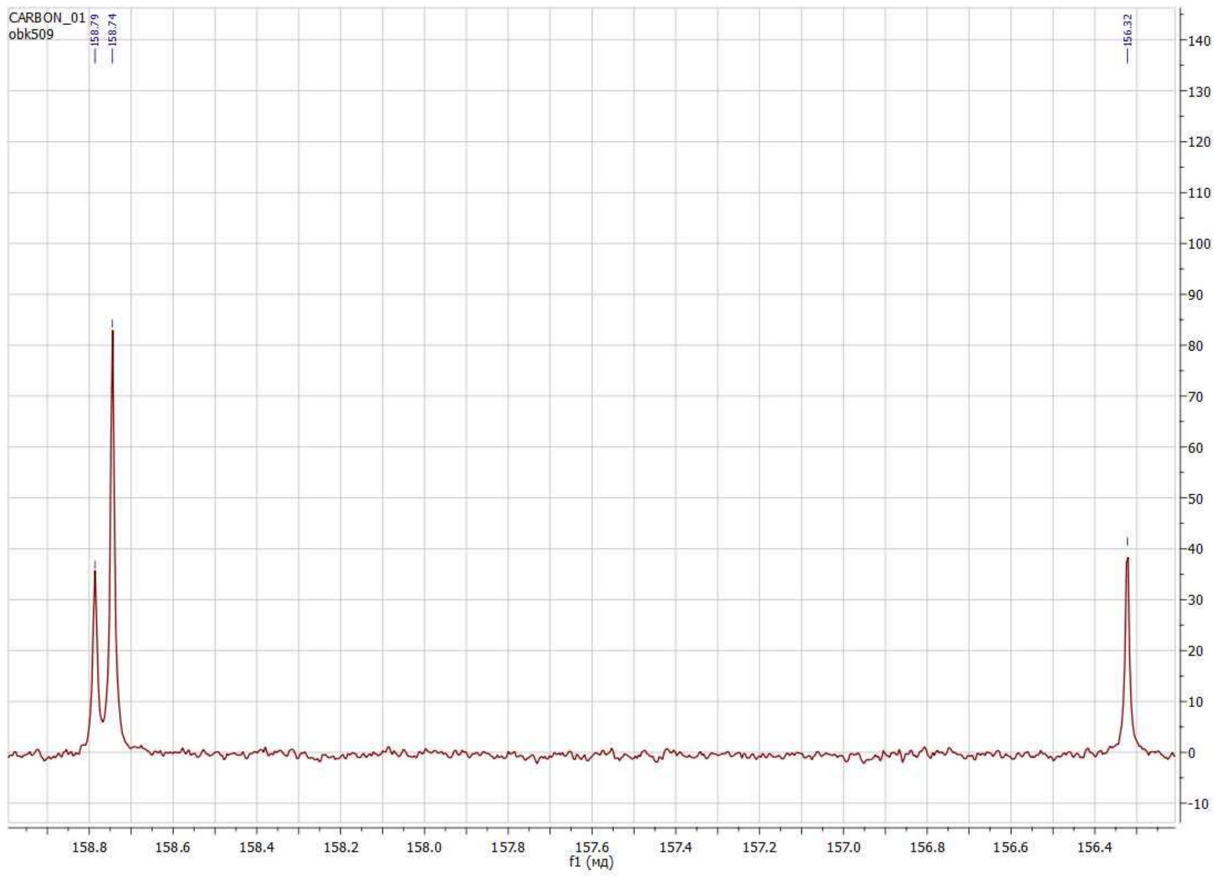

^{13}C NMR spectra of compounds **3e** (DMSO-*d6*)

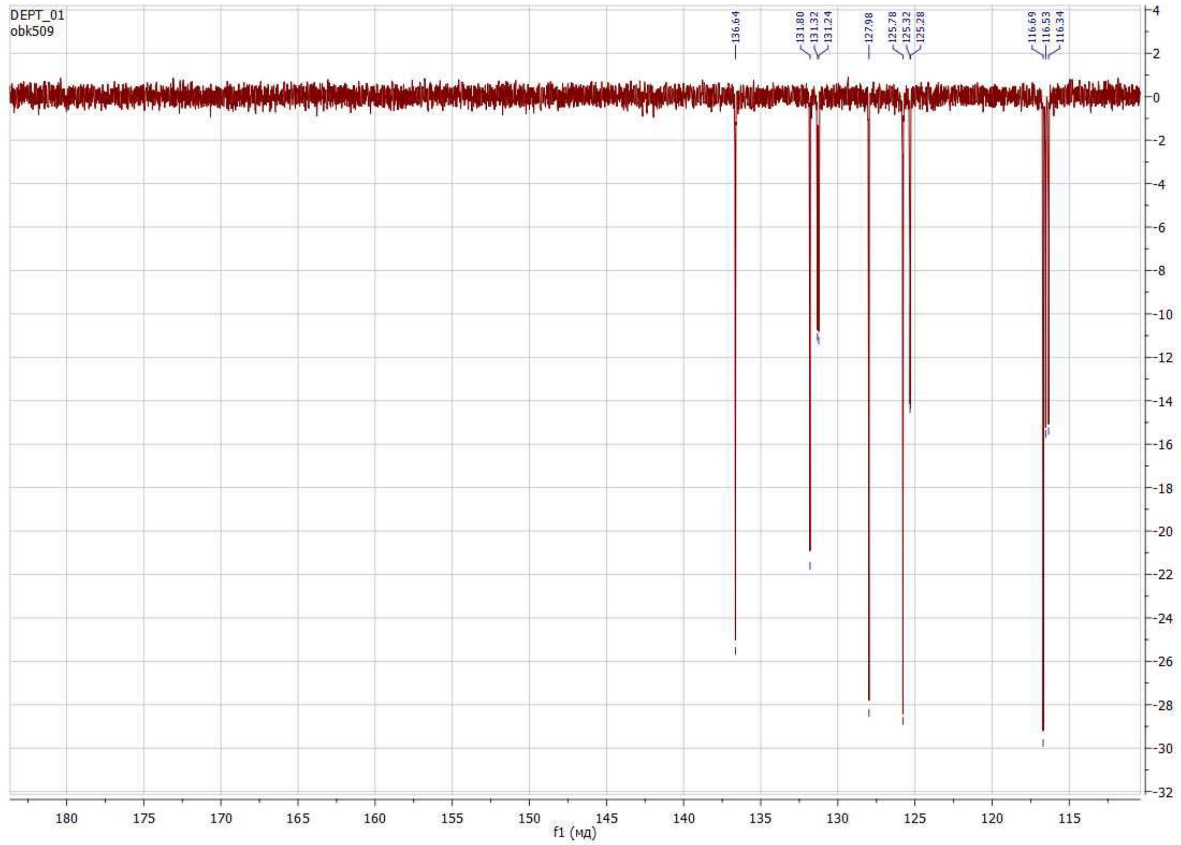

^{13}C DEPT NMR spectra of compounds **3e** (DMSO-*d*6)

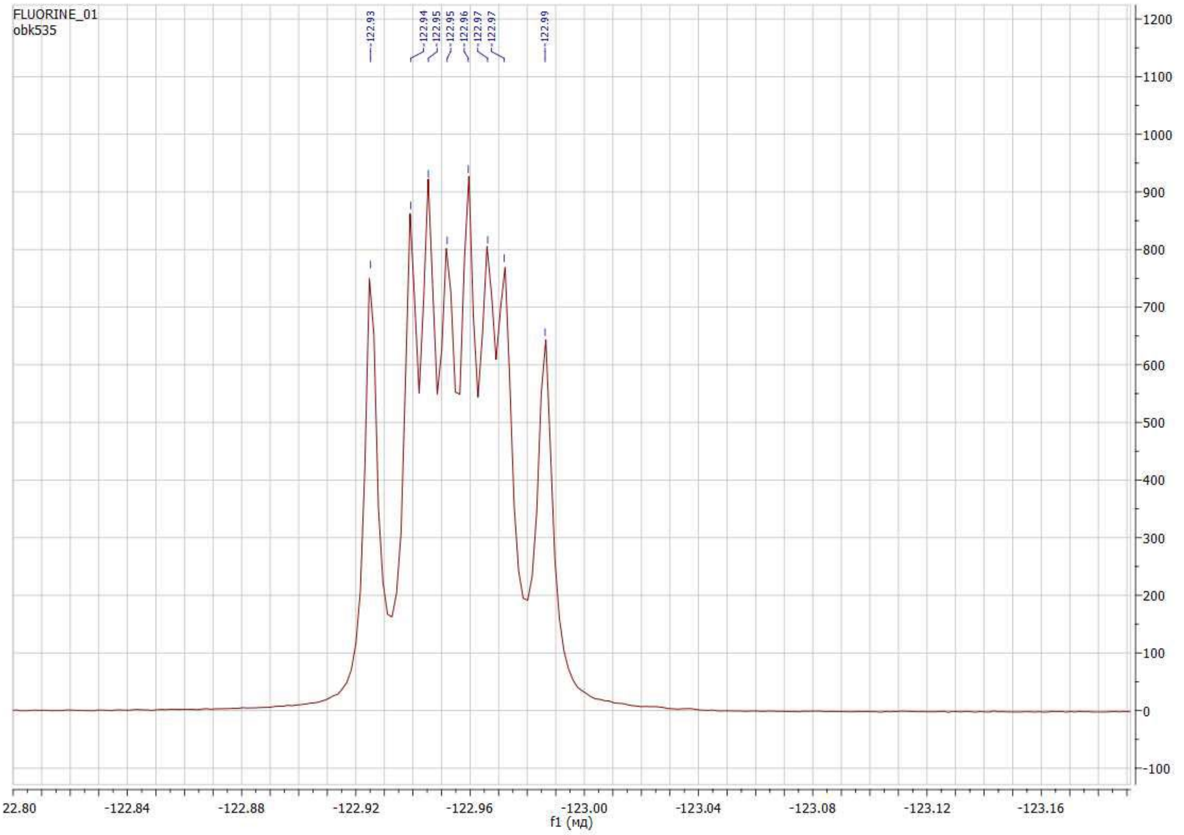

^{19}F NMR spectra of compounds **3e** (DMSO-*d*6)

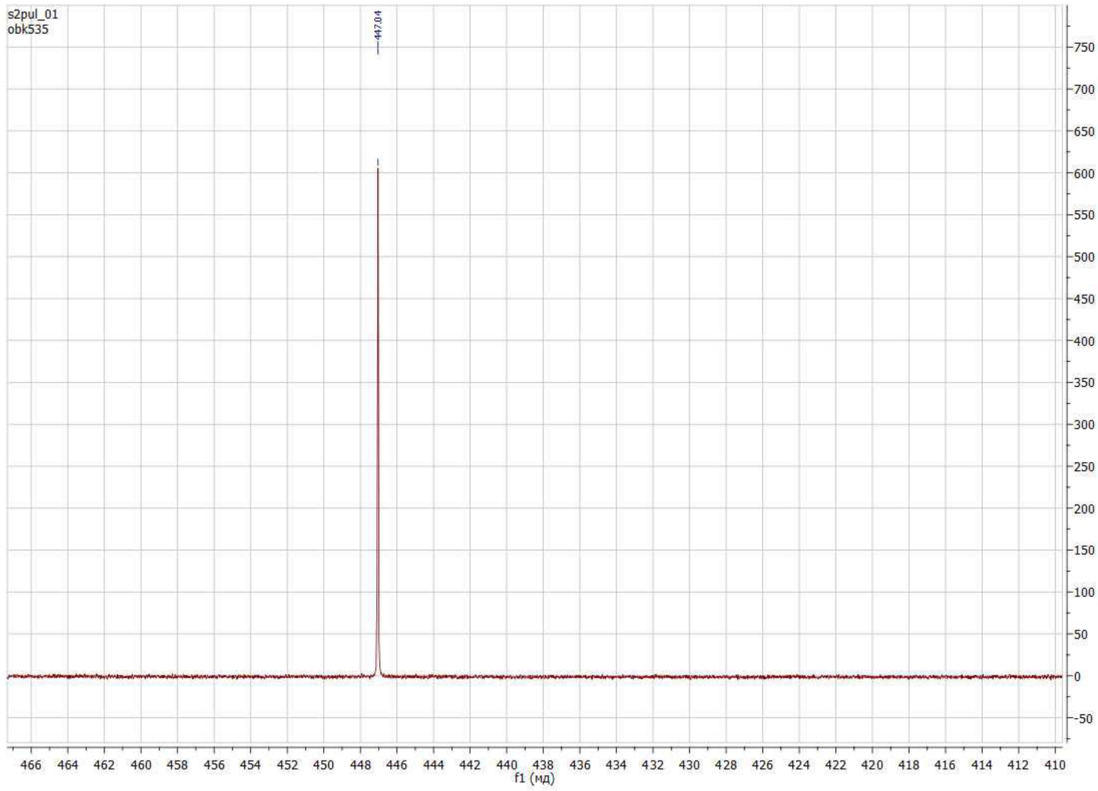

^{77}Se NMR spectra of compounds **3e** (DMSO-*d*6)

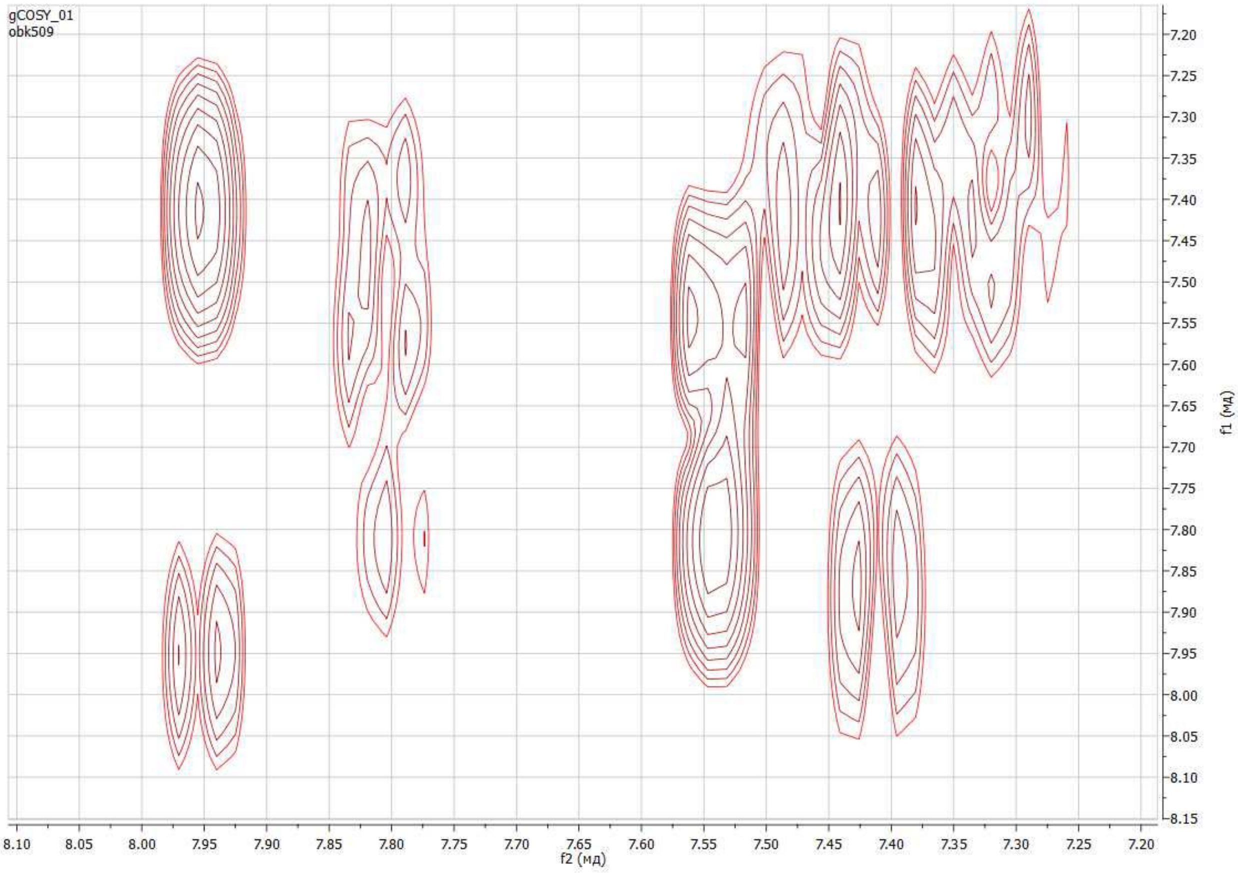

COSY NMR spectra of compounds **3e** (DMSO-*d*6)

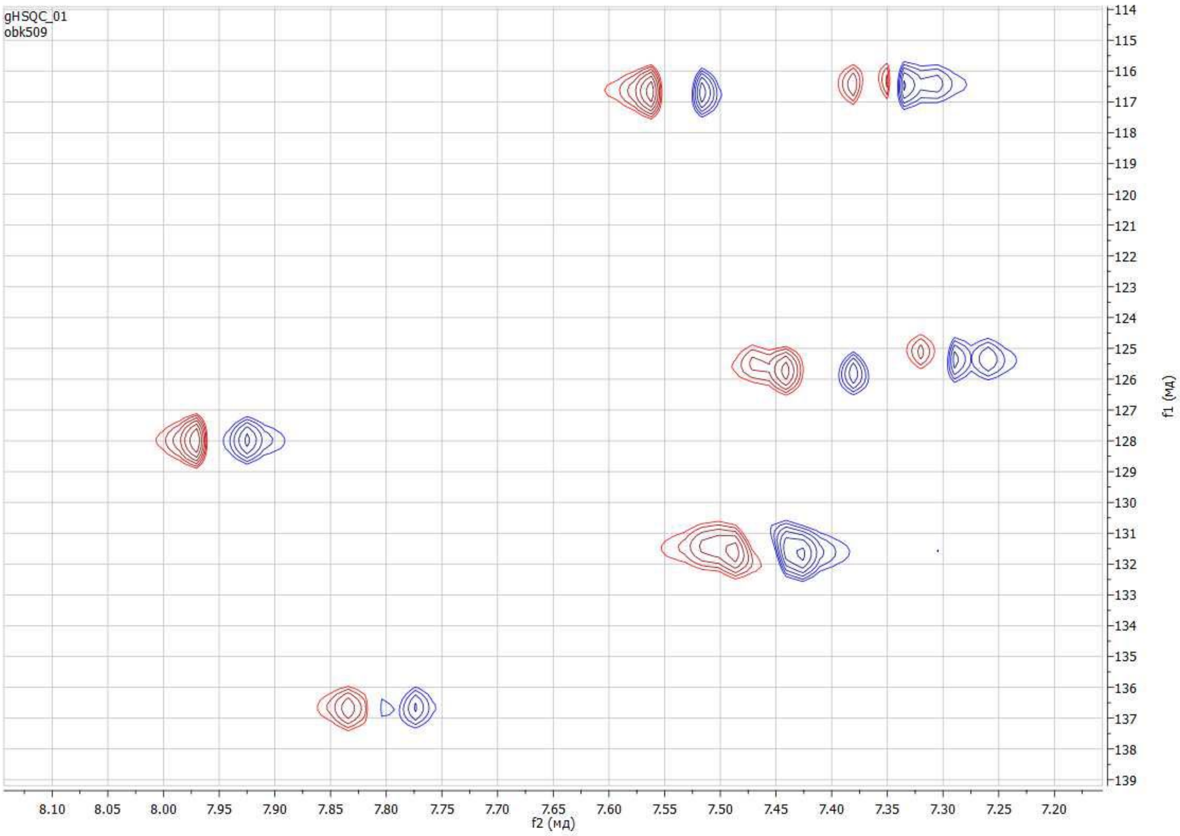

HSQC ^{1}H-^{13}C NMR spectra of compounds **3e** (DMSO-*d*6)

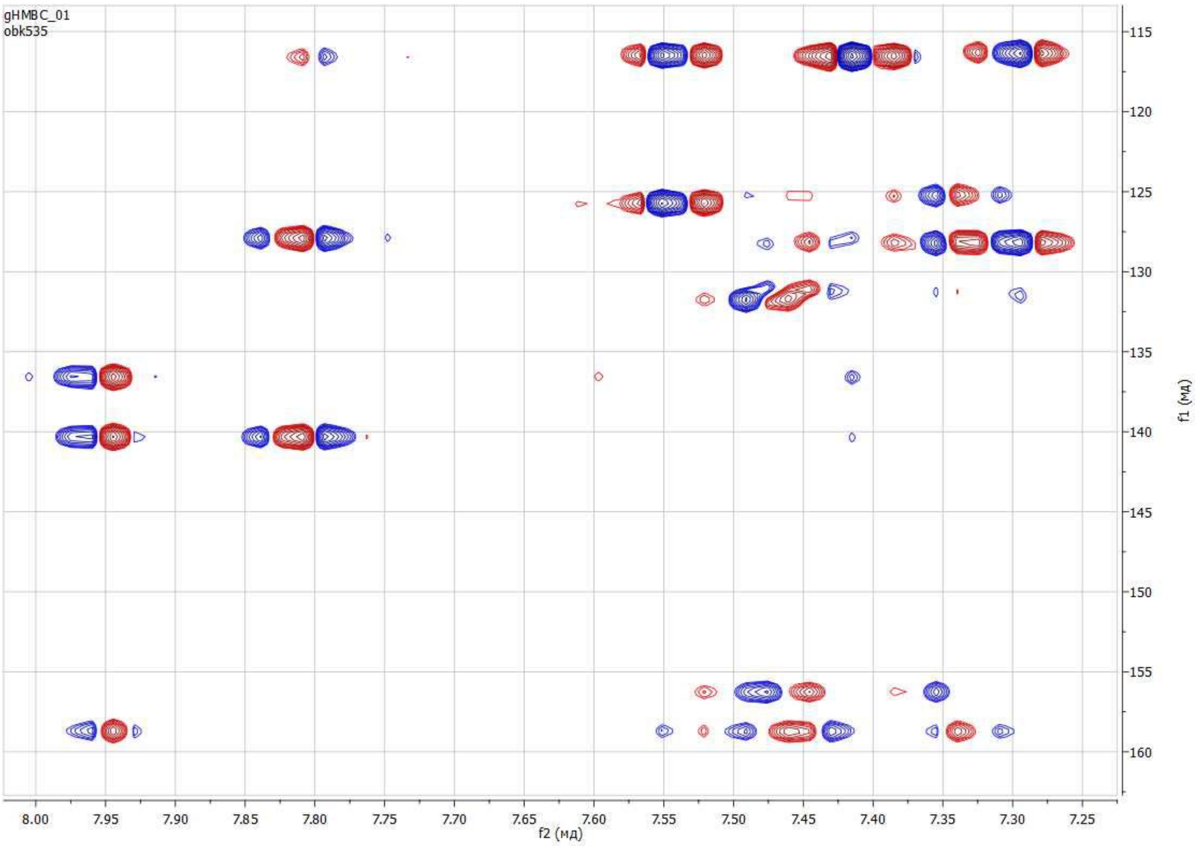

HMBC ^{1}H-^{13}C NMR spectra of compounds **3e** (DMSO-*d*6)

Display Report

Analysis Info

Analysis Name	D:\Data\Chizhov\Osmanov\Mar_15_2021\bko-81_&clblow.d	Acquisition Date	15.03.2021 15:06:22
Method	tune_low.m	Operator	BDAL@DE
Sample Name	/CHIZ BKO-81	Instrument / Ser#	micrOTOF 10248
Comment	CH3CN 100 %, dil. 200, calibrant added		

Acquisition Parameter

Source Type	ESI	Ion Polarity	Positive	Set Nebulizer	0.4 Bar
Focus	Not active			Set Dry Heater	180 °C
Scan Begin	50 m/z	Set Capillary	4500 V	Set Dry Gas	4.0 l/min
Scan End	3000 m/z	Set End Plate Offset	-500 V	Set Divert Valve	Waste

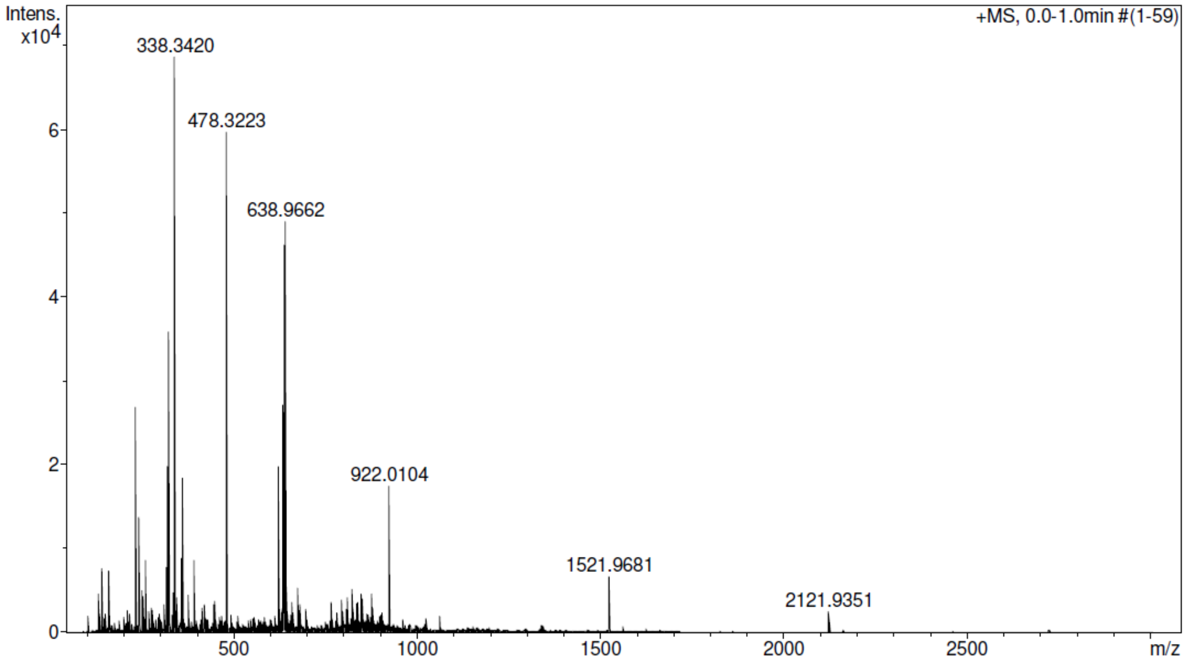

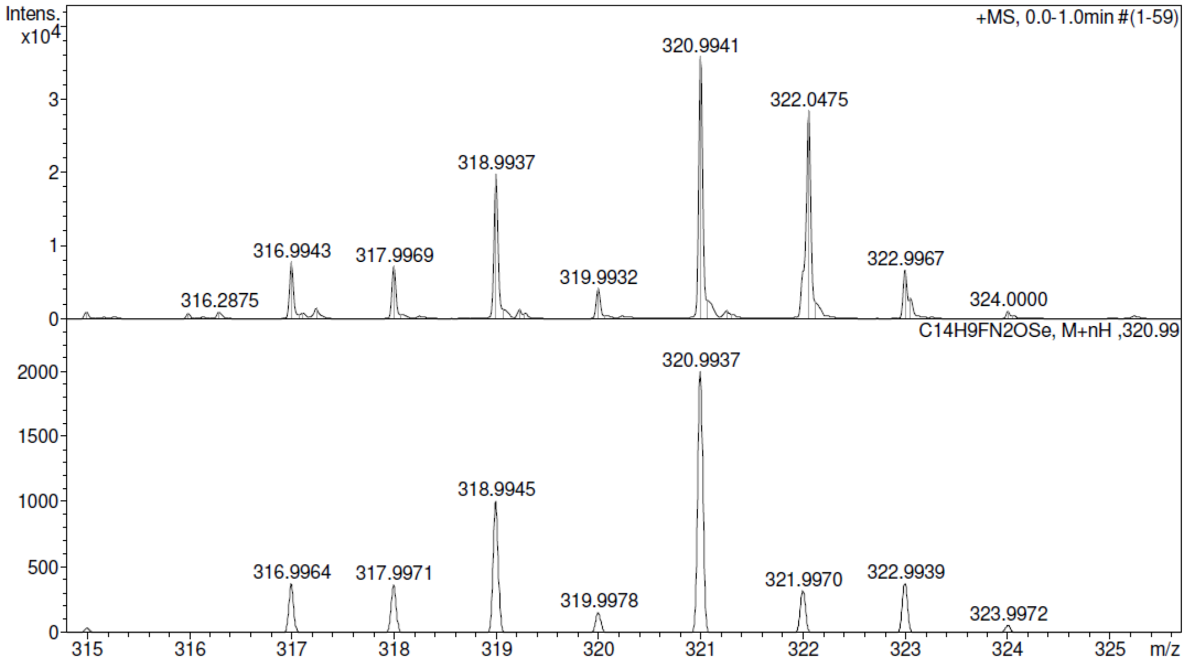

High resolution mass spectra (HR MS) of compounds **3e**

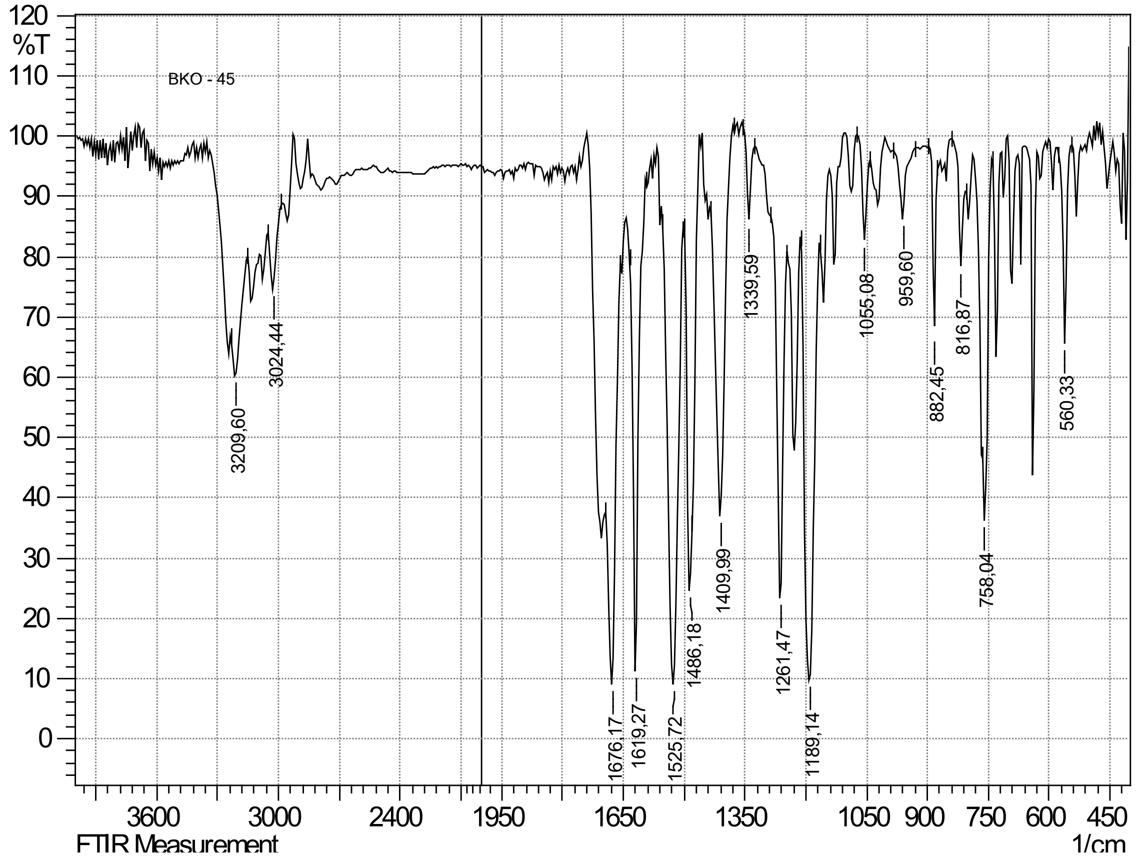

FTIR spectra of compounds **3f**

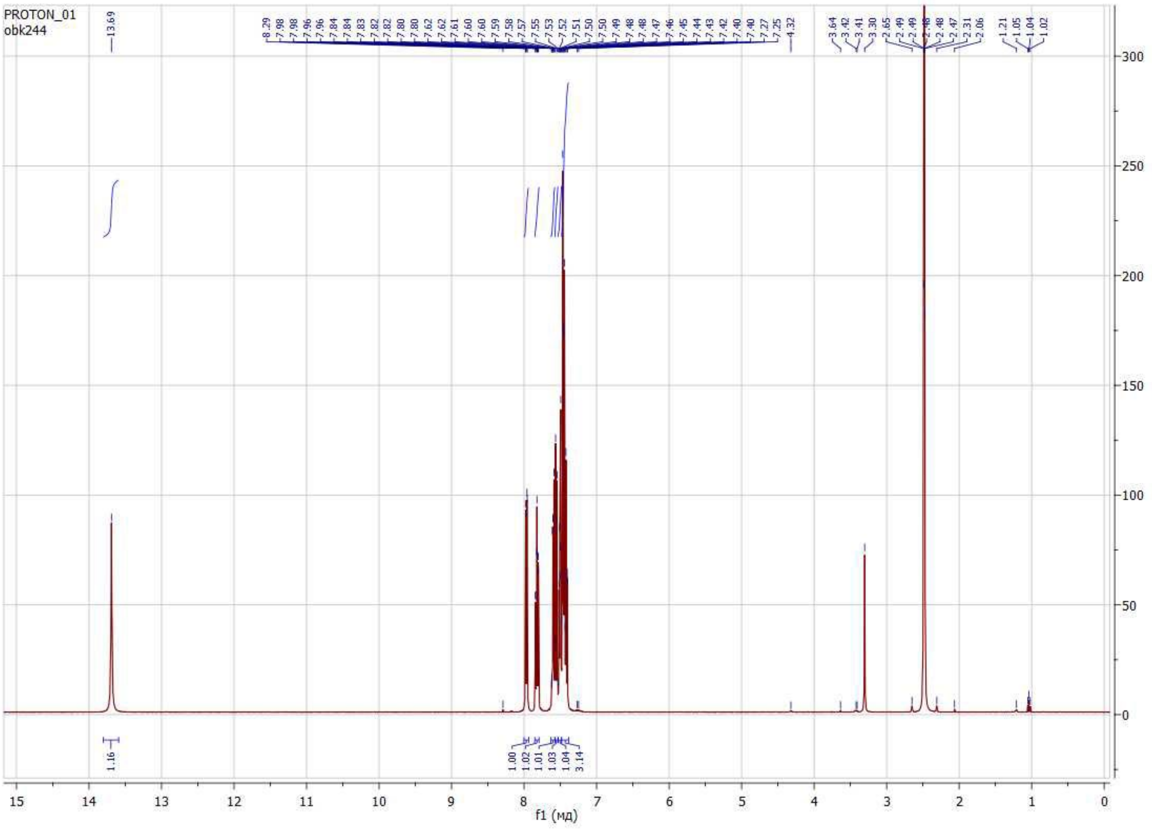

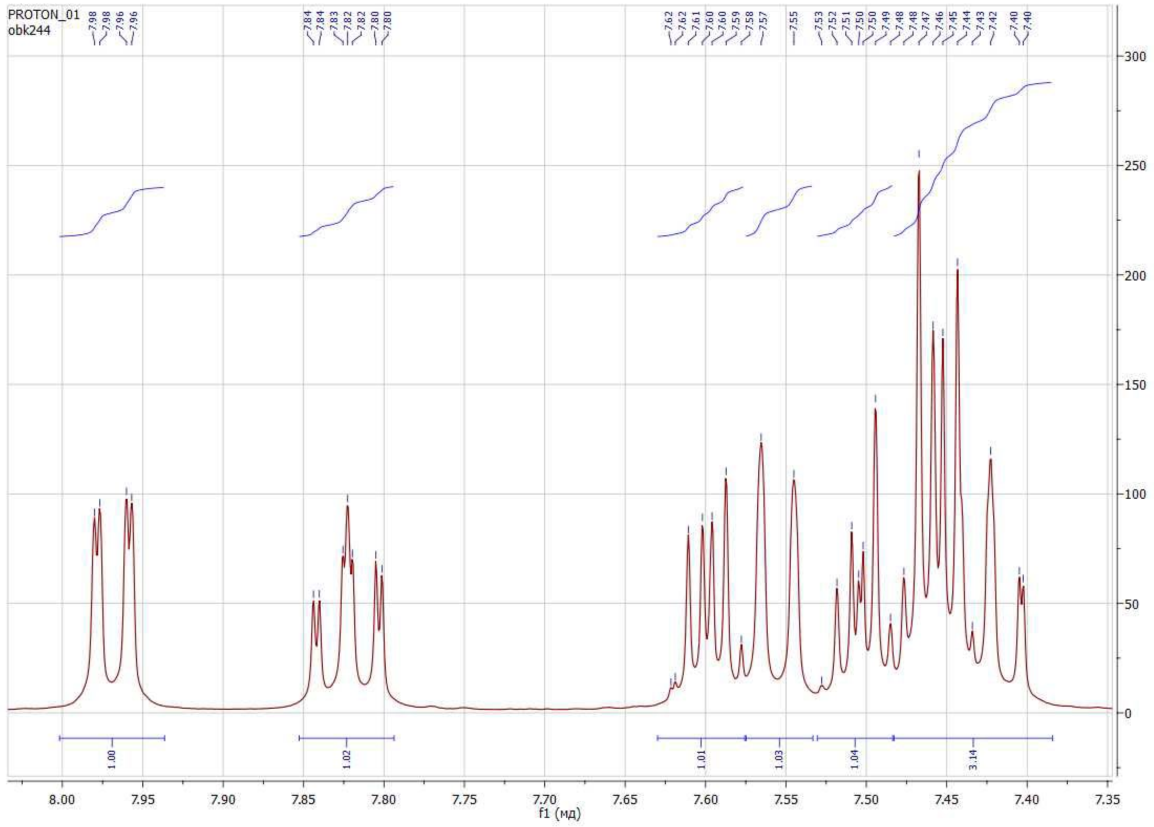

^{1}H NMR spectra of compounds **3f** (DMSO-*d*6)

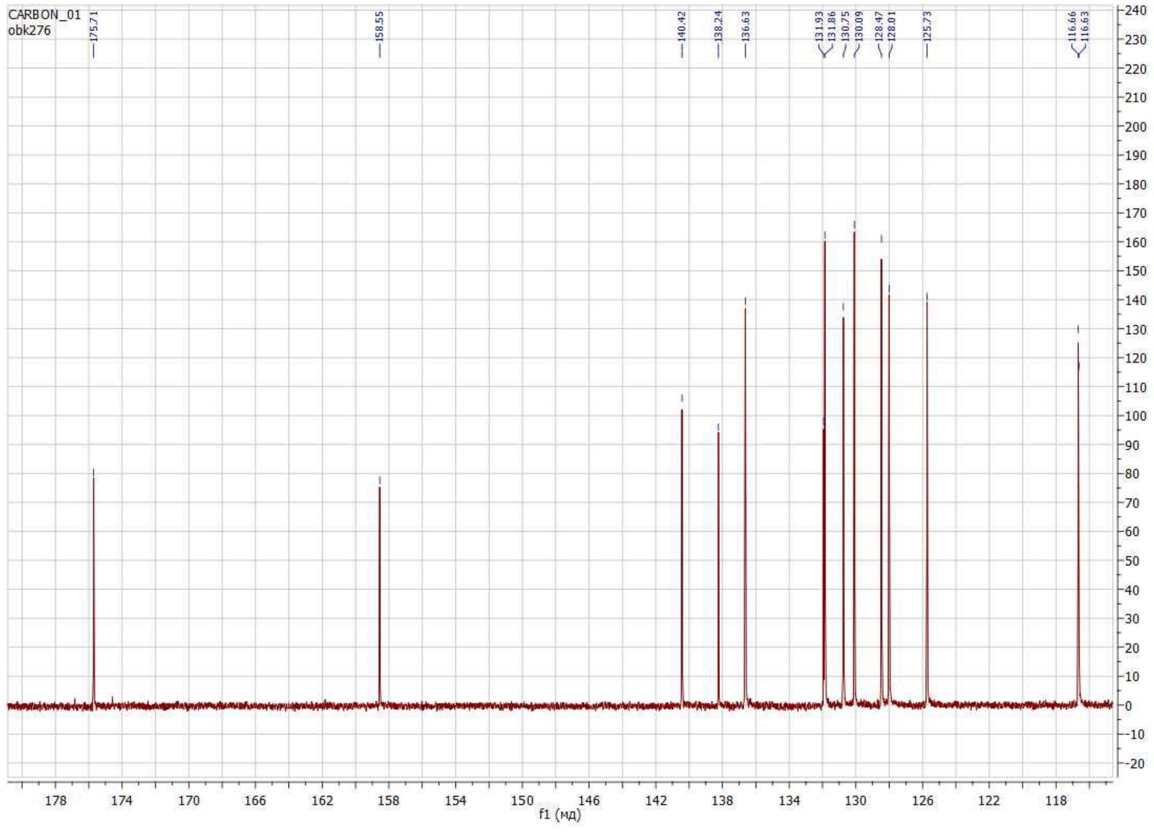

^{13}C NMR spectra of compounds **3f** (DMSO-*d*6)

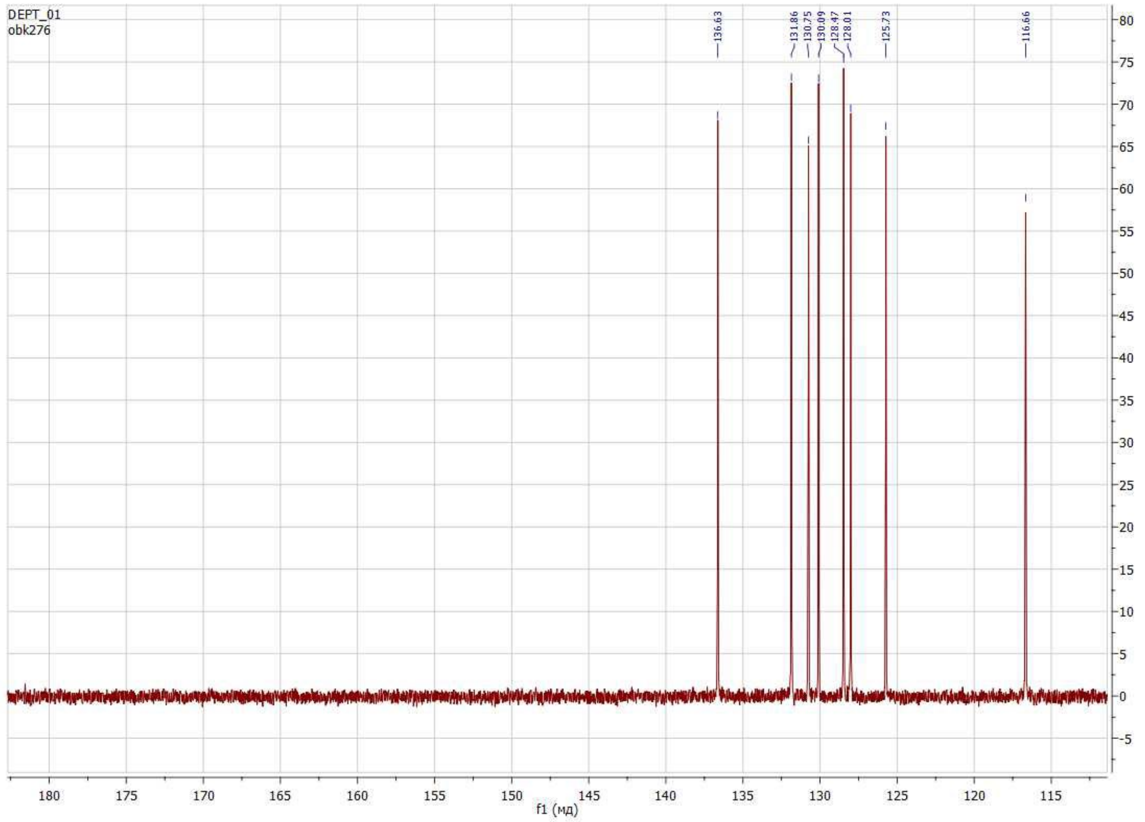

^{13}C DEPT NMR spectra of compounds **3f** (DMSO-*d*6)

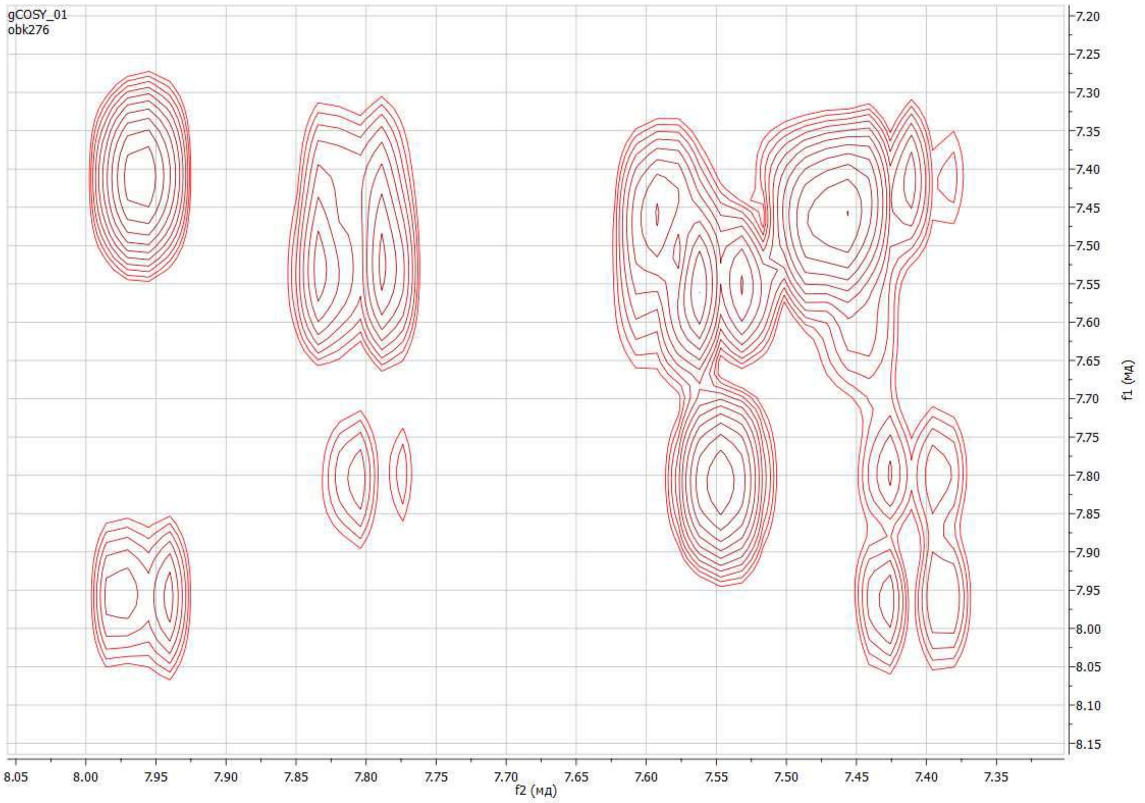

COSY NMR spectra of compounds **3f** (DMSO-*d*6)

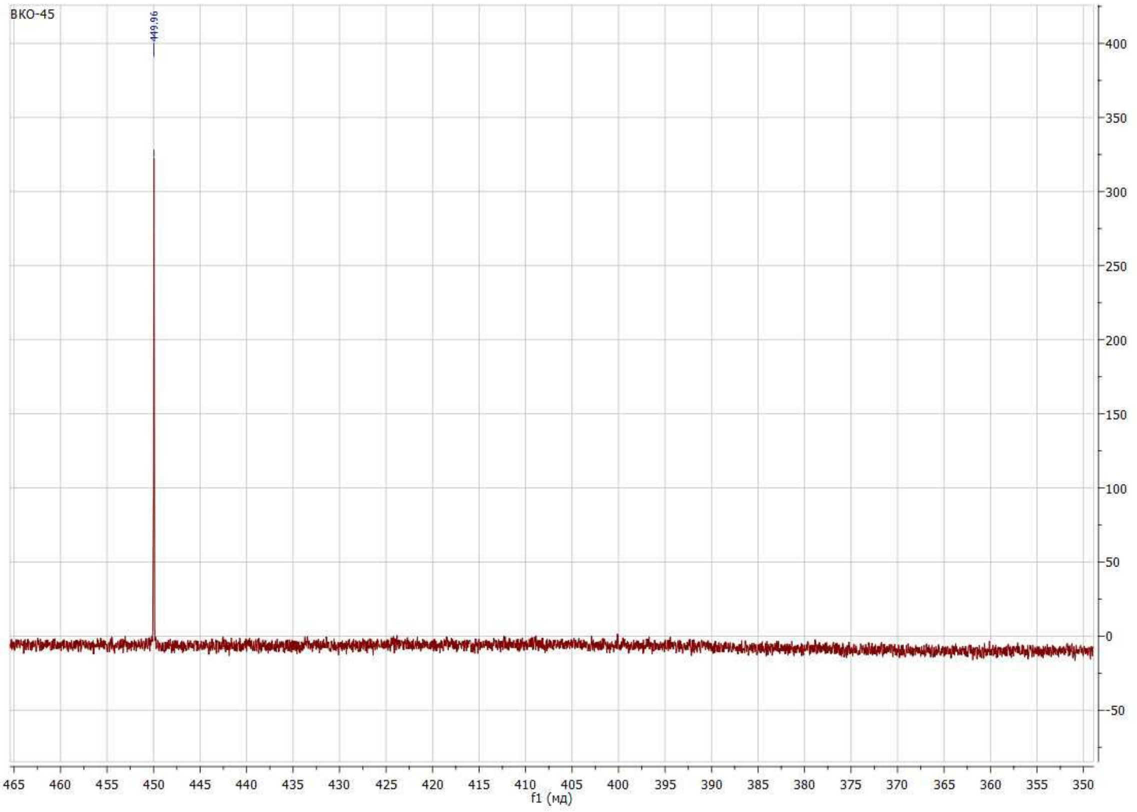

^{77}Se NMR spectra of compounds **3f** (DMSO-*d*6)

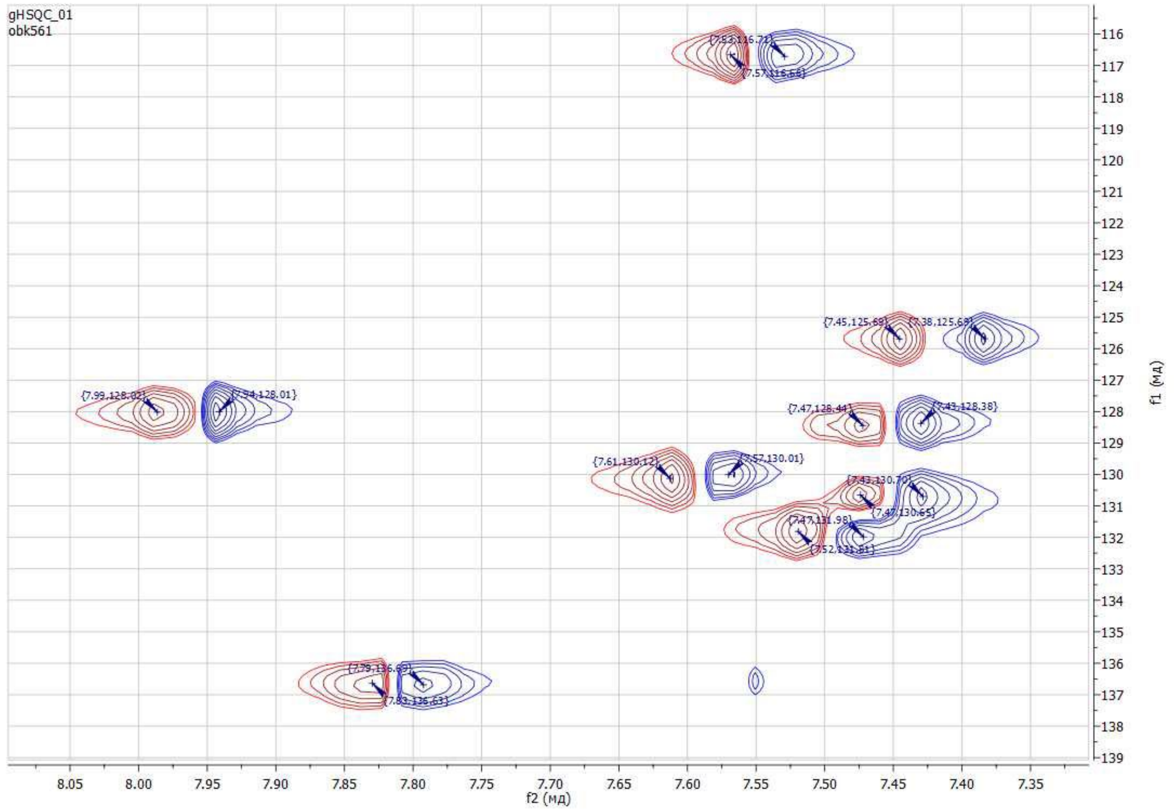

HSQC ^{1}H-^{13}C NMR spectra of compounds **3f** (DMSO-*d*6)

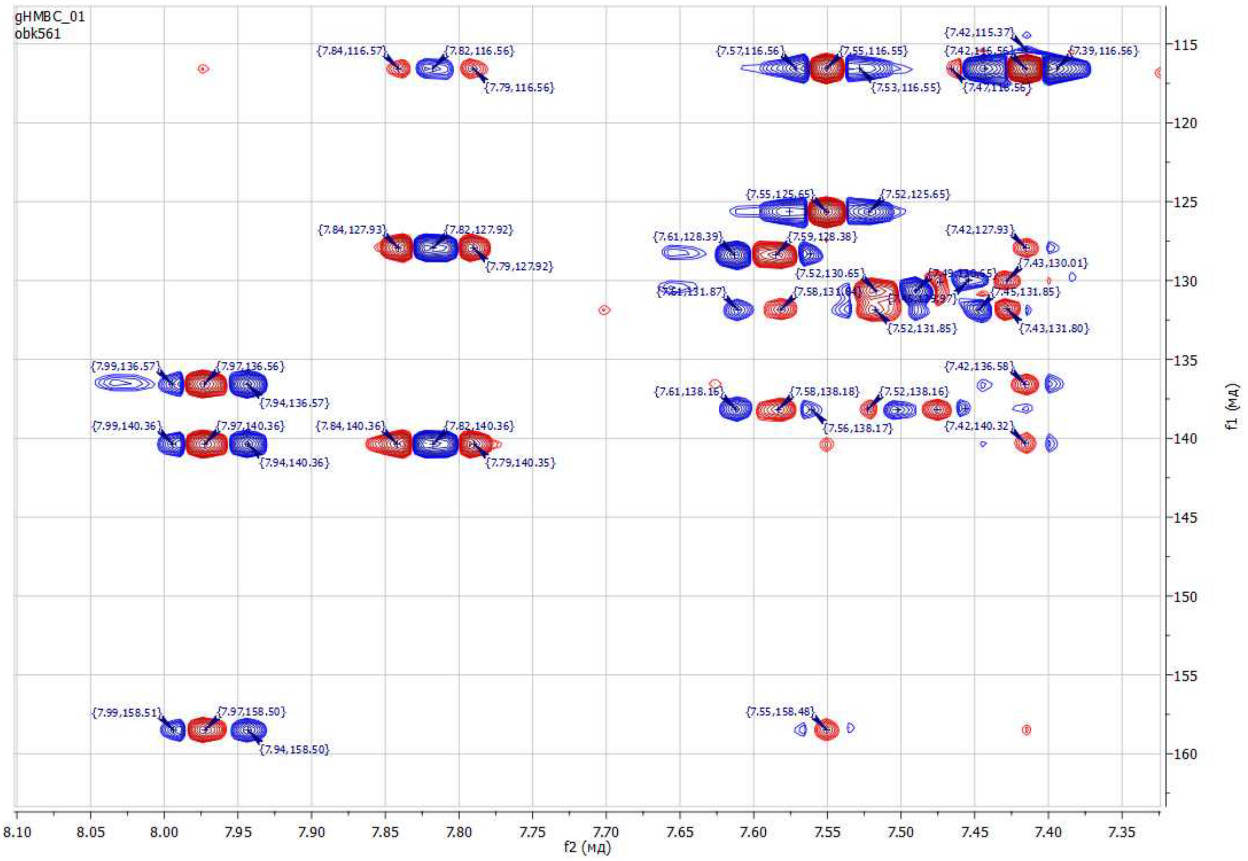

HMBC ^{1}H-^{13}C NMR spectra of compounds **3f** (DMSO-*d*6)

Display Report

Analysis Info

Analysis Name	C:\AOC2019\Osmanov\Nov_18\Nov_18_2019\bko-45_&clblow.d	Acquisition Date	18.11.2019 14:47:53
Method	tune_low.m	Operator	BDAL@DE
Sample Name	/CHIZ BKO-45	Instrument / Ser#	micrOTOF 10248
Comment	CH3CN 100 %, dil. 20, calibrant added		

Acquisition Parameter

Source Type	ESI	Ion Polarity	Positive	Set Nebulizer	0.4 Bar
Focus	Not active			Set Dry Heater	180 °C
Scan Begin	50 m/z	Set Capillary	4500 V	Set Dry Gas	4.0 l/min
Scan End	3000 m/z	Set End Plate Offset	-500 V	Set Divert Valve	Waste

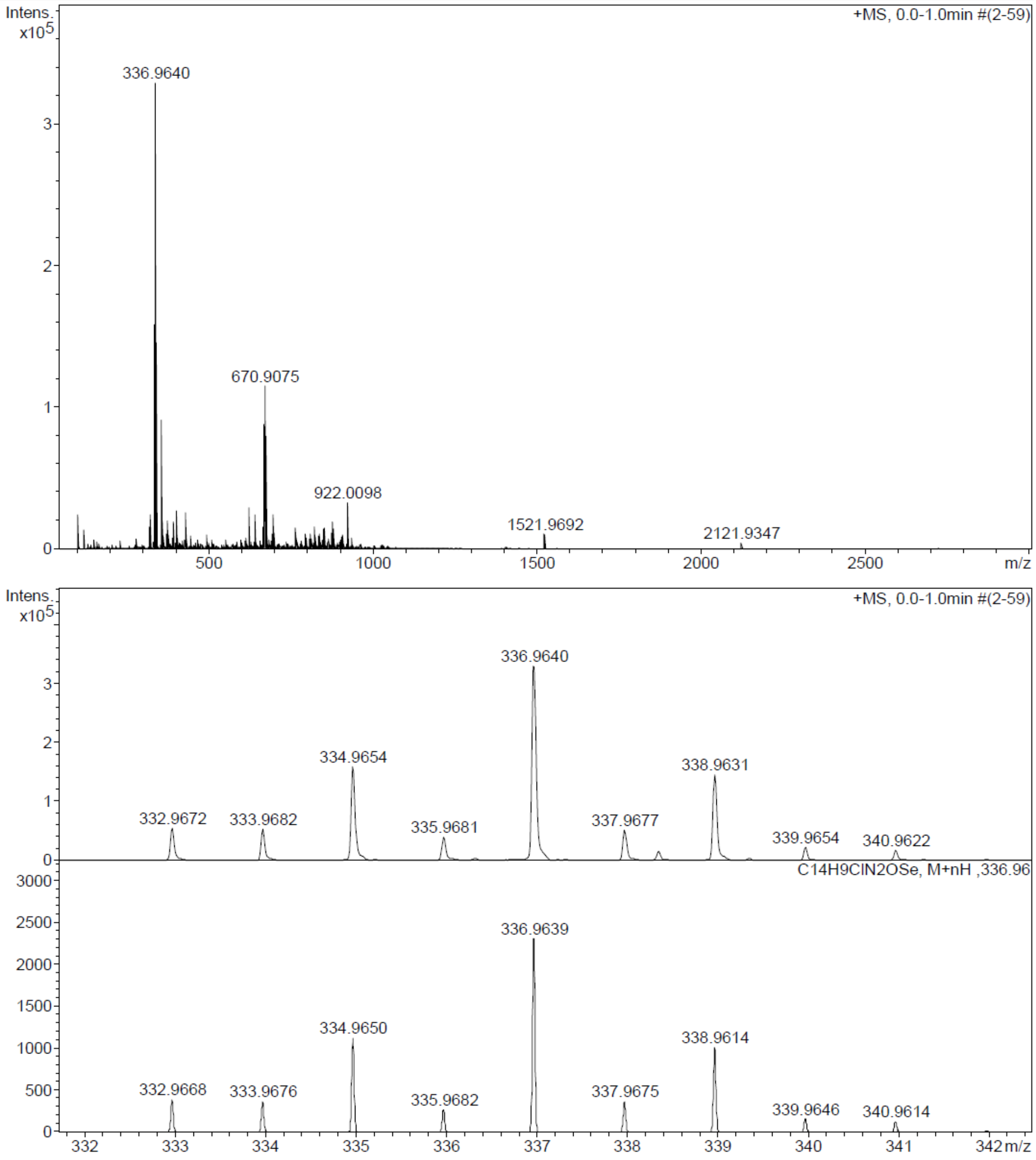

High resolution mass spectra (HR MS) of compounds **3f**

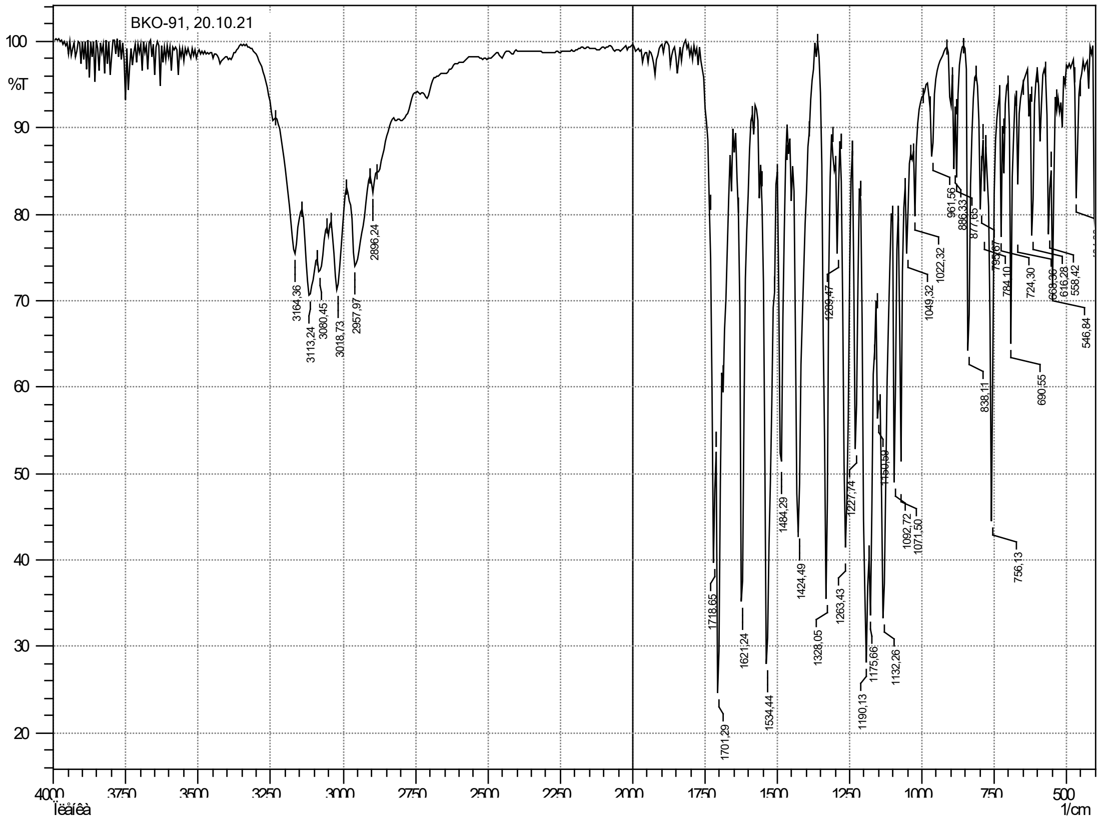

FTIR spectra of compounds **3g**

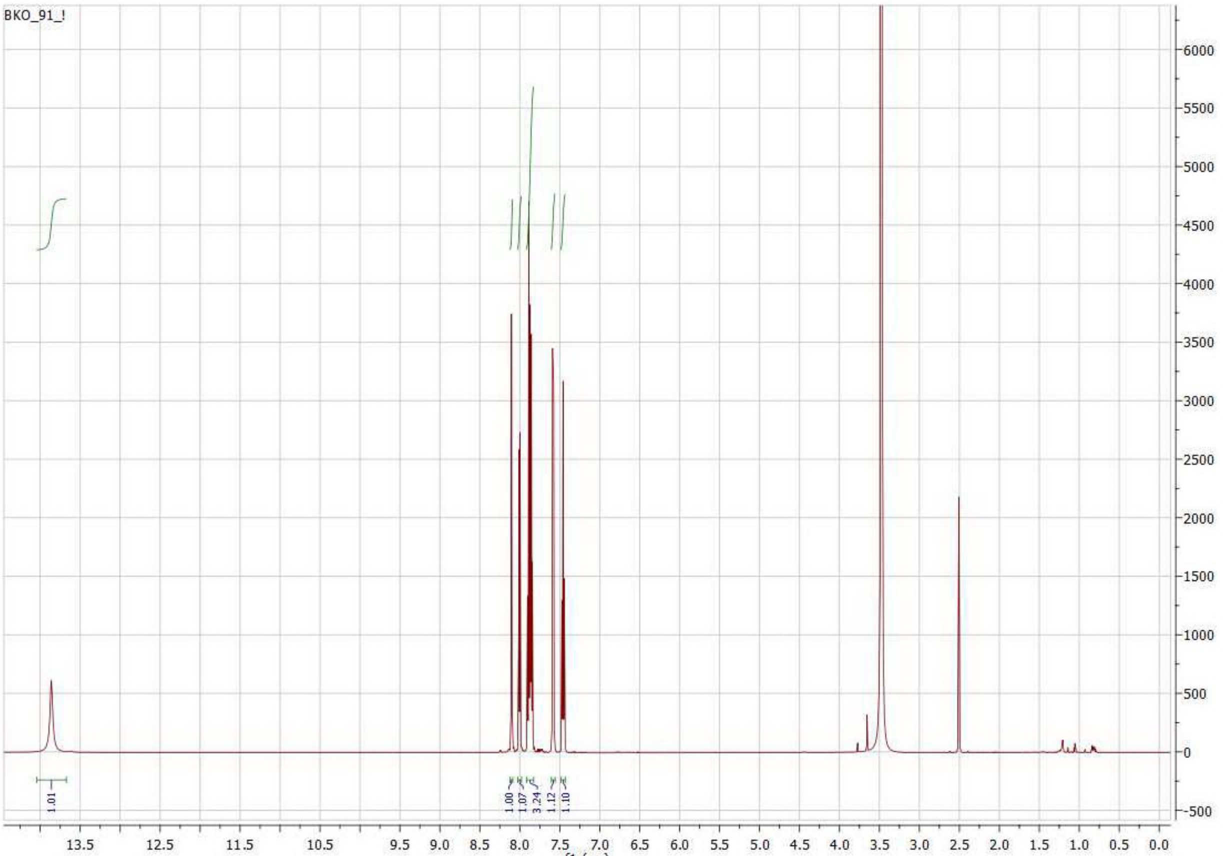

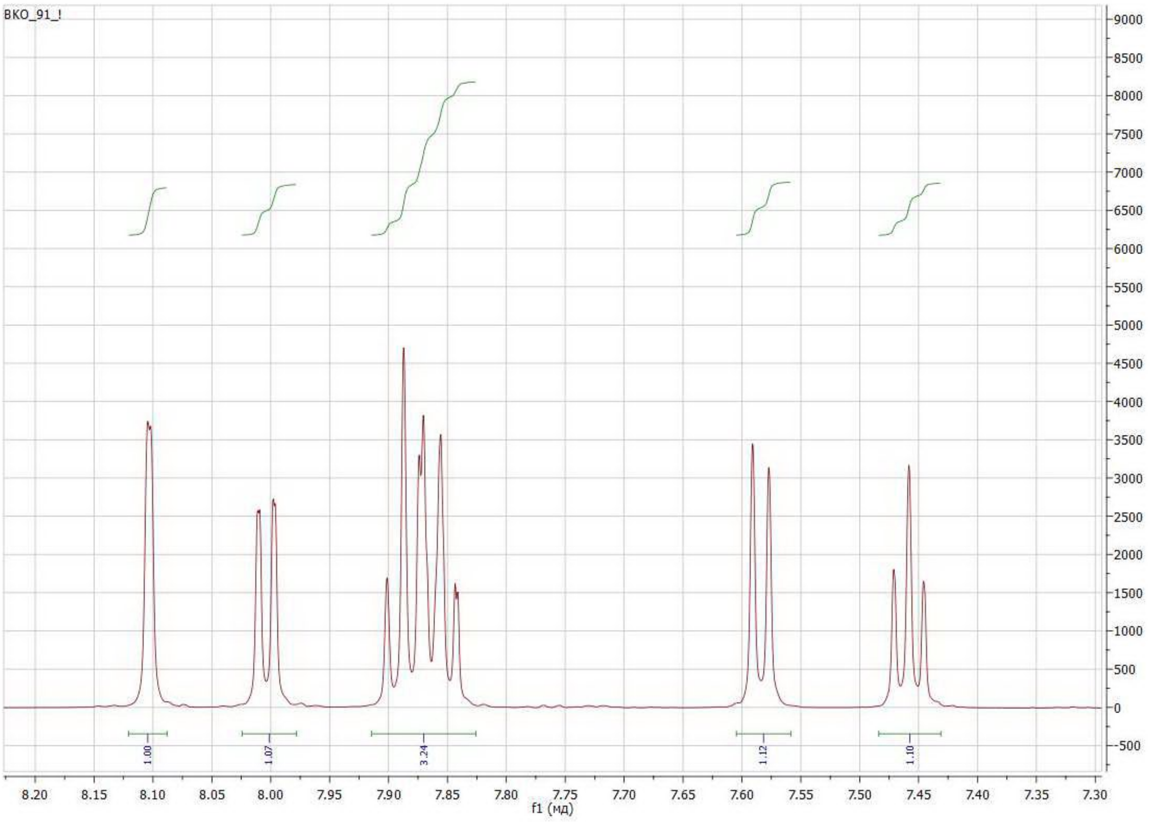

^{1}H NMR spectra of compounds **3g** (DMSO-*d*6)

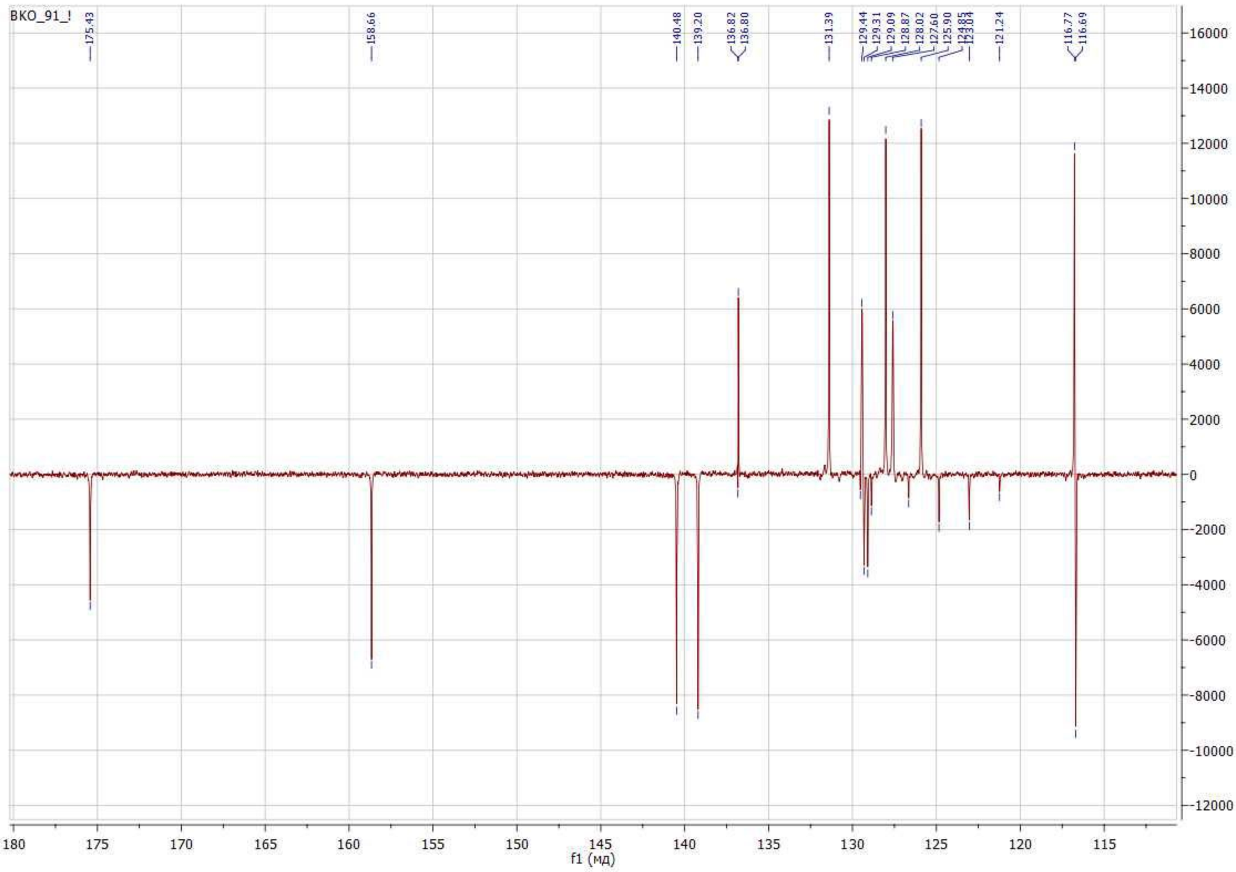

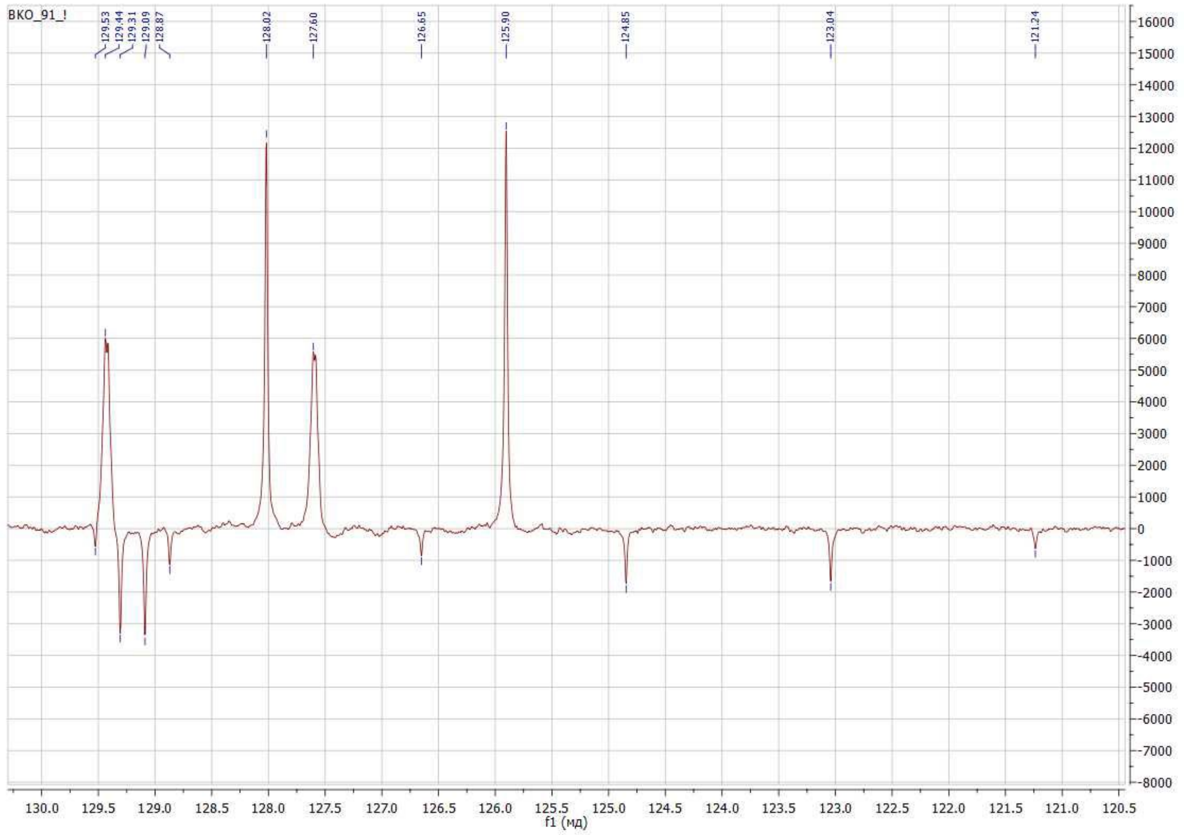

^{13}C NMR spectra of compounds **3g** (DMSO-*d*6)

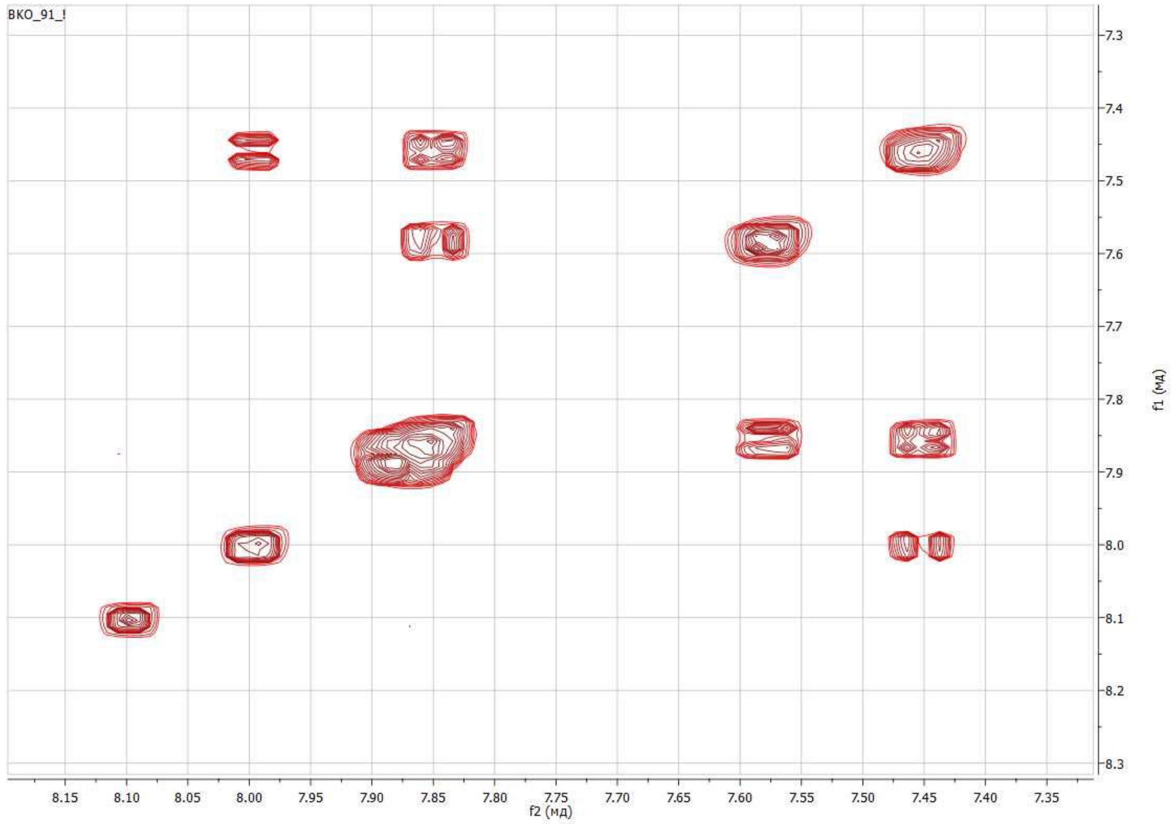

COSY NMR spectra of compounds **3g** (DMSO-*d*6)

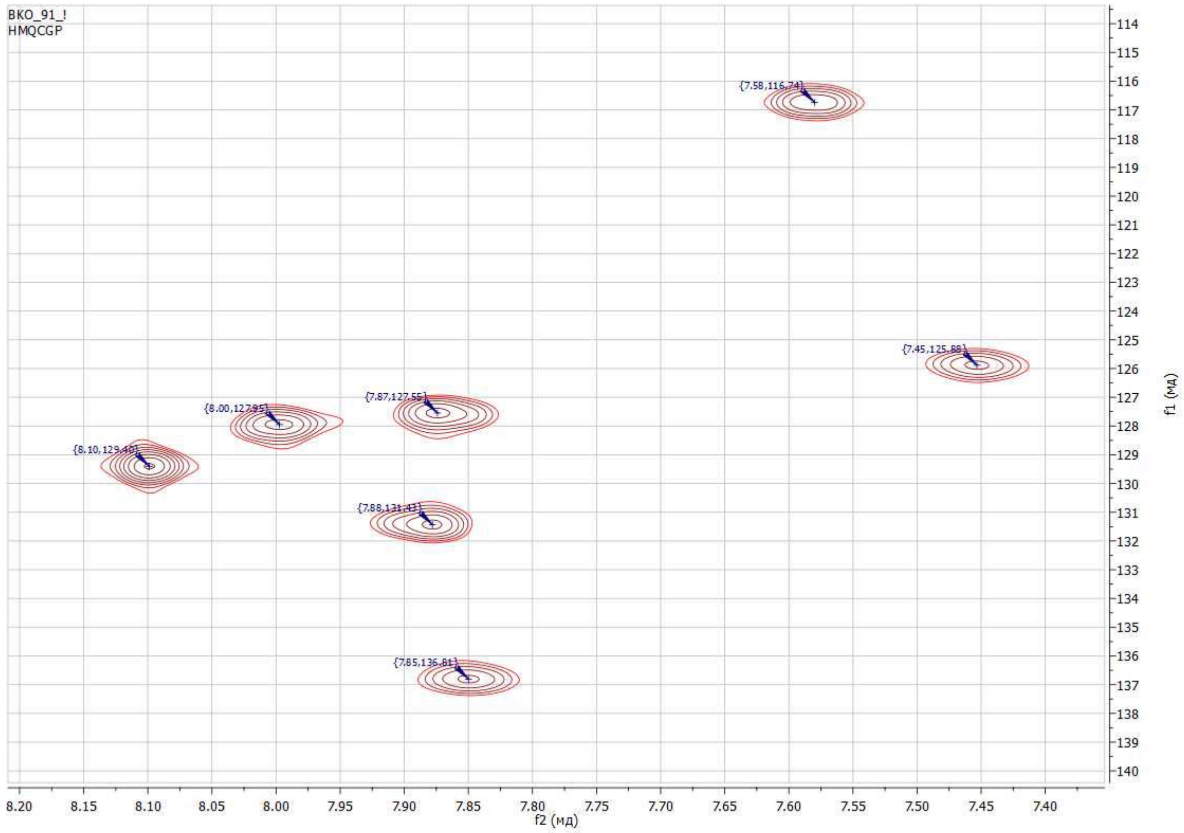

HSQC ^{1}H-^{13}C NMR spectra of compounds **3g** (DMSO-*d*6)

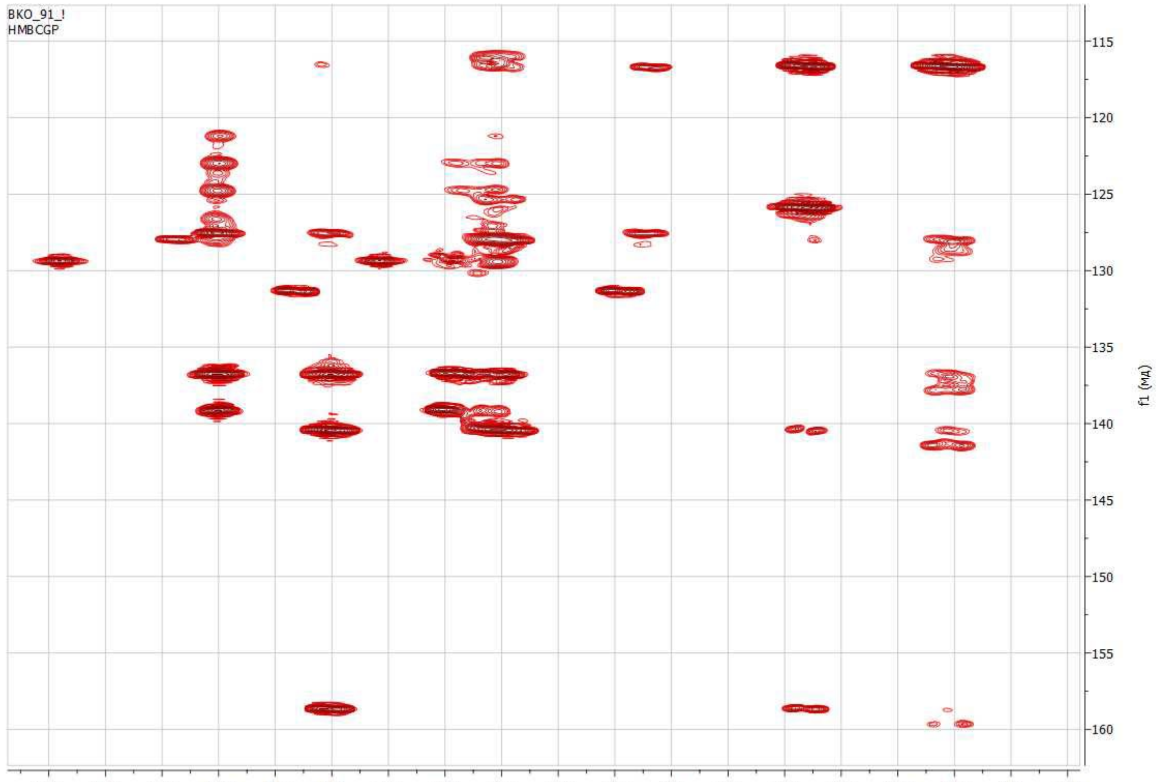

HMBC ^{1}H-^{13}C NMR spectra of compounds **3g** (DMSO-*d*6)

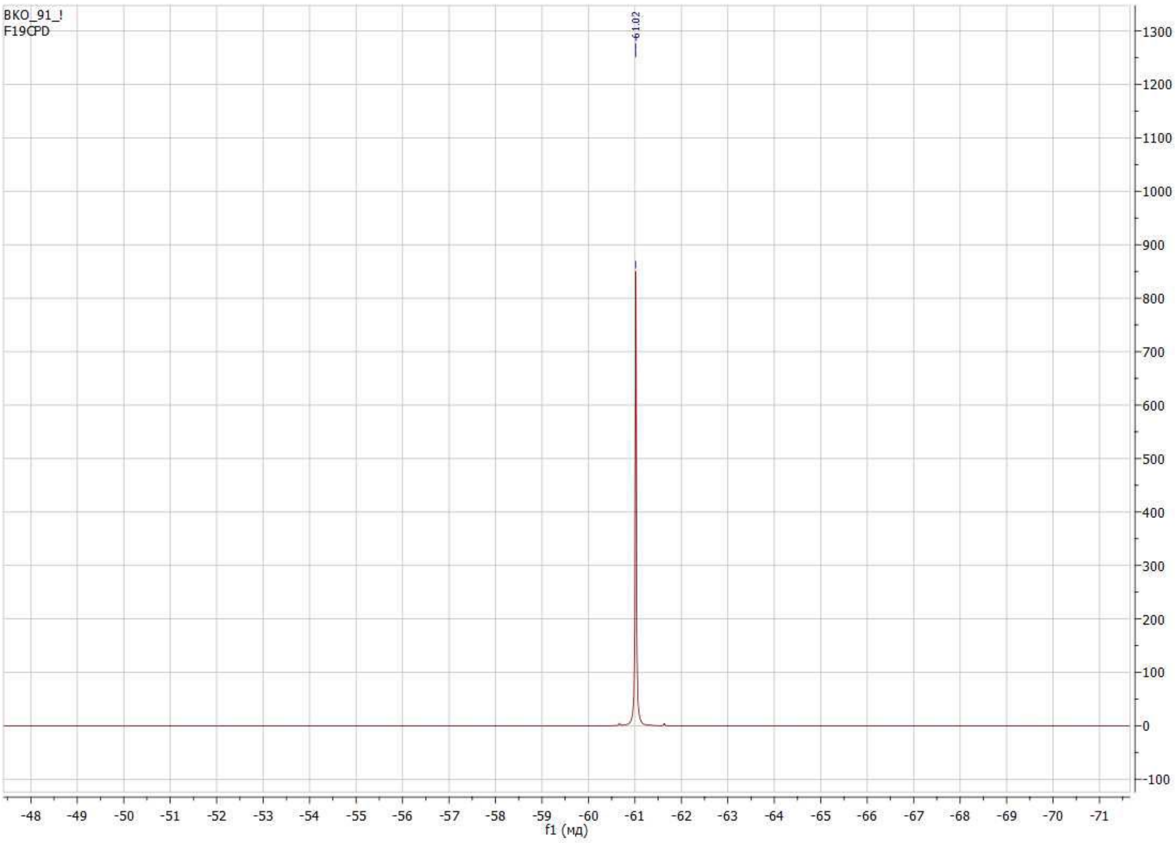

^{19}F NMR spectra of compounds 3**g** (DMSO-*d*6)

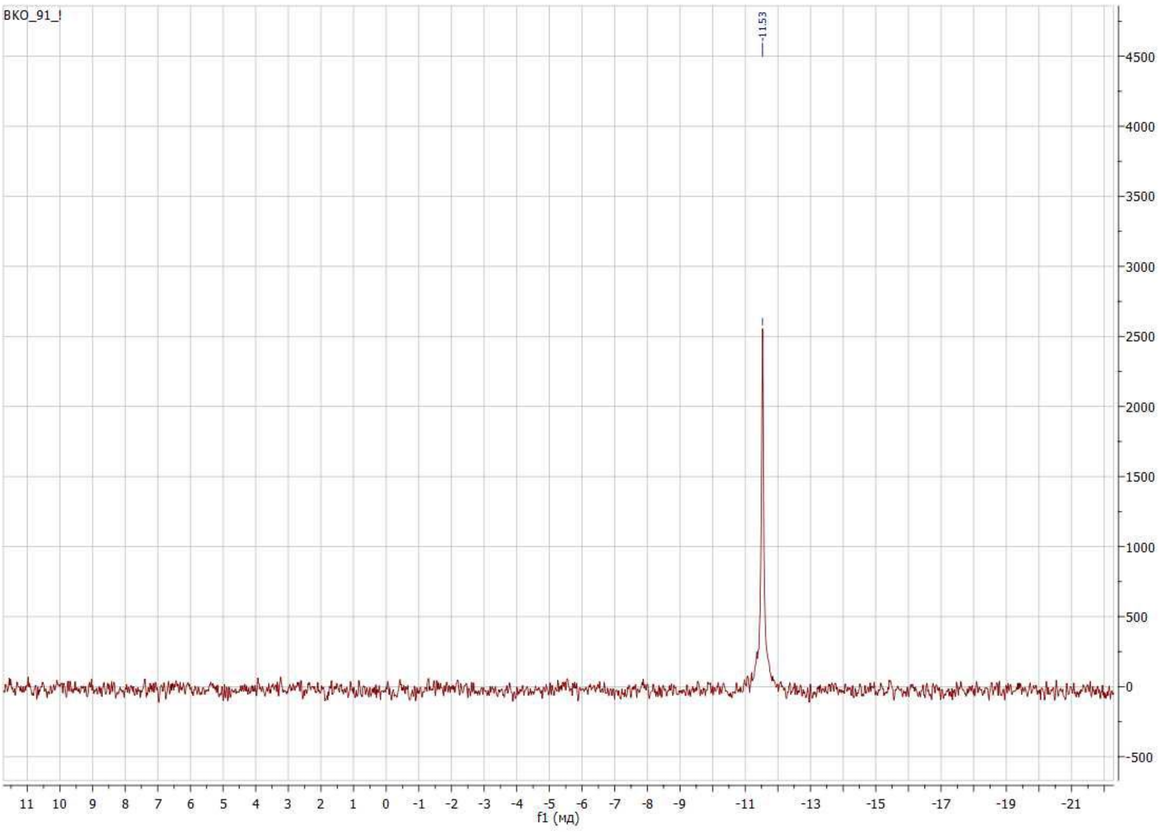

^{77}Se NMR spectra of compounds **3g** (DMSO-*d*6)

Display Report

Analysis Info

Analysis Name	D:\Data\Chizhov\Osmanov\Aug_26_2021\bko-91-1_&clb.d	Acquisition Date	26.08.2021 13:58:02
Method	tune_wide.m	Operator	BDAL@DE
Sample Name	/CHIZ BKO-91	Instrument / Ser#	micrOTOF 10248
Comment	CH3OH 100 %, dil. 200, calibrant added		

Acquisition Parameter

Source Type	ESI	Ion Polarity	Positive	Set Nebulizer	0.4 Bar
Focus	Not active			Set Dry Heater	180 °C
Scan Begin	50 m/z	Set Capillary	4500 V	Set Dry Gas	4.0 l/min
Scan End	3000 m/z	Set End Plate Offset	-500 V	Set Divert Valve	Waste

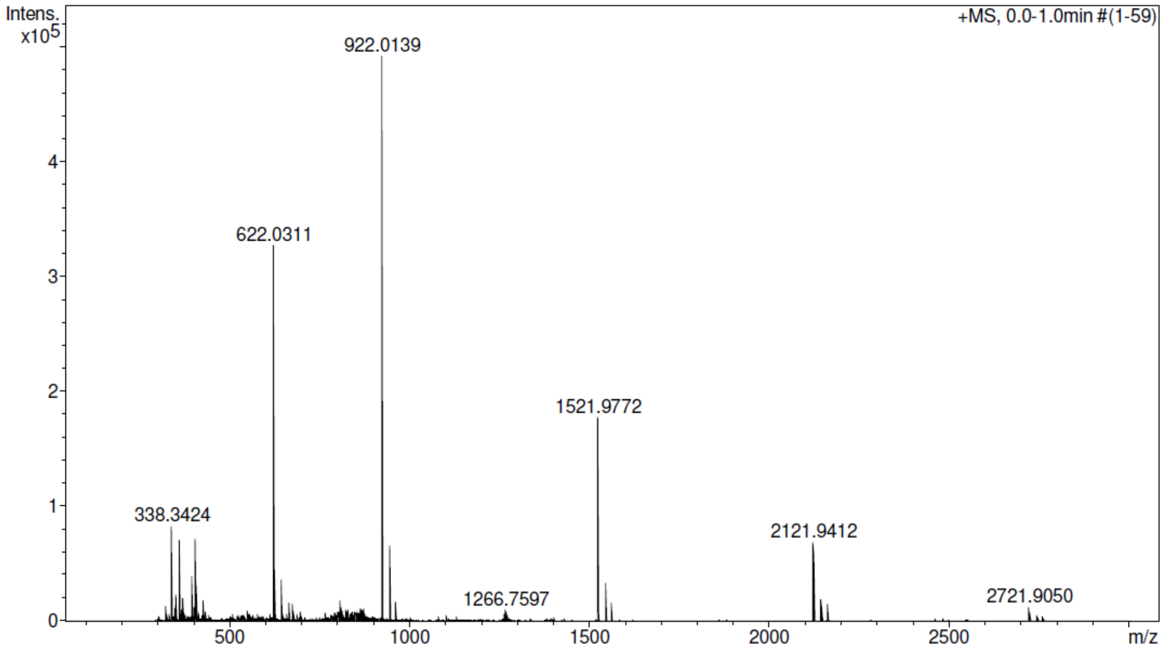

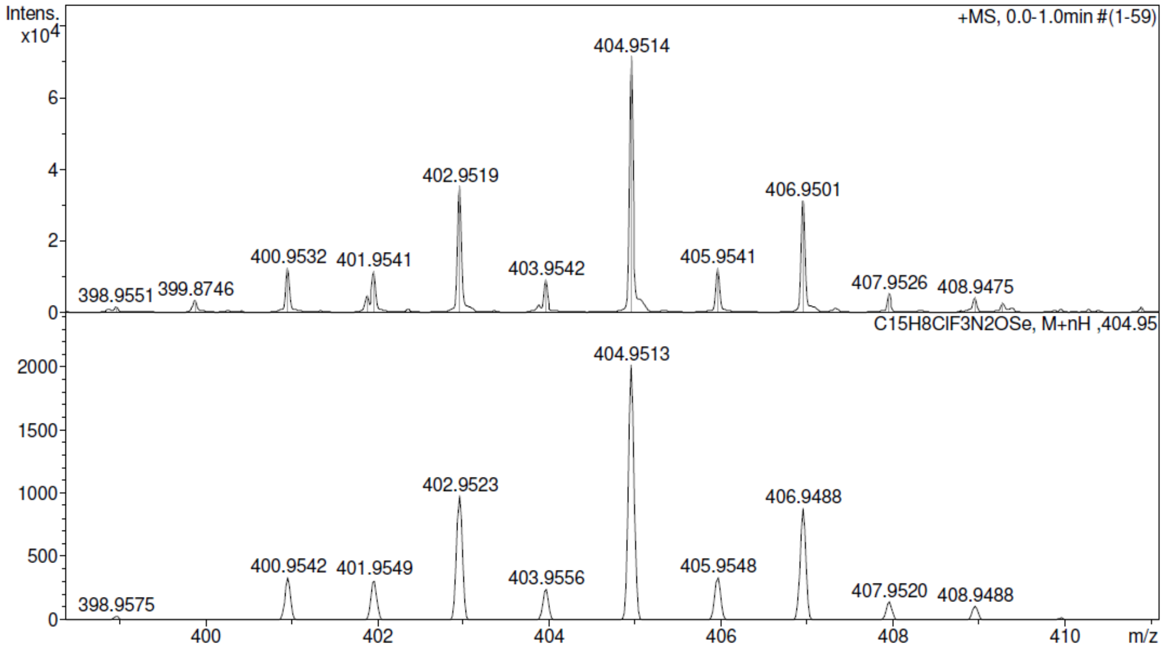

High resolution mass spectra (HR MS) of compounds **3g**

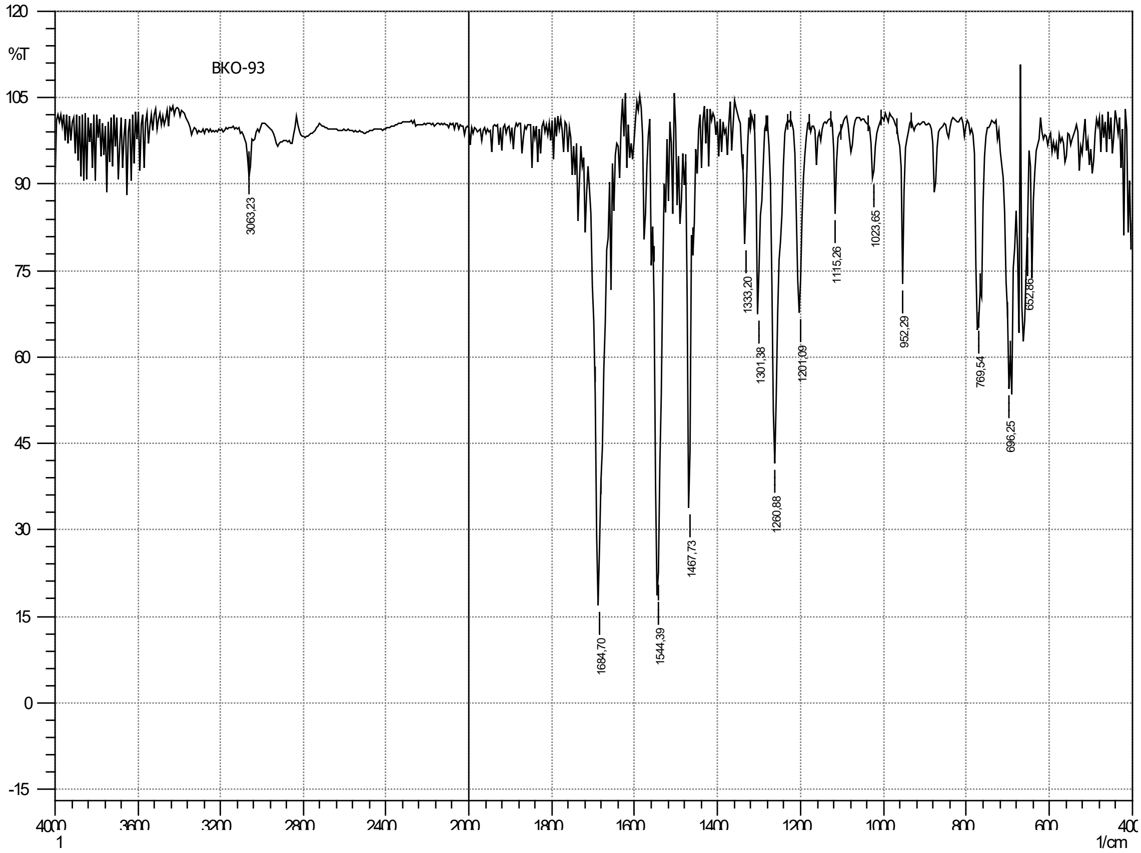

FTIR spectra of compounds **4a**

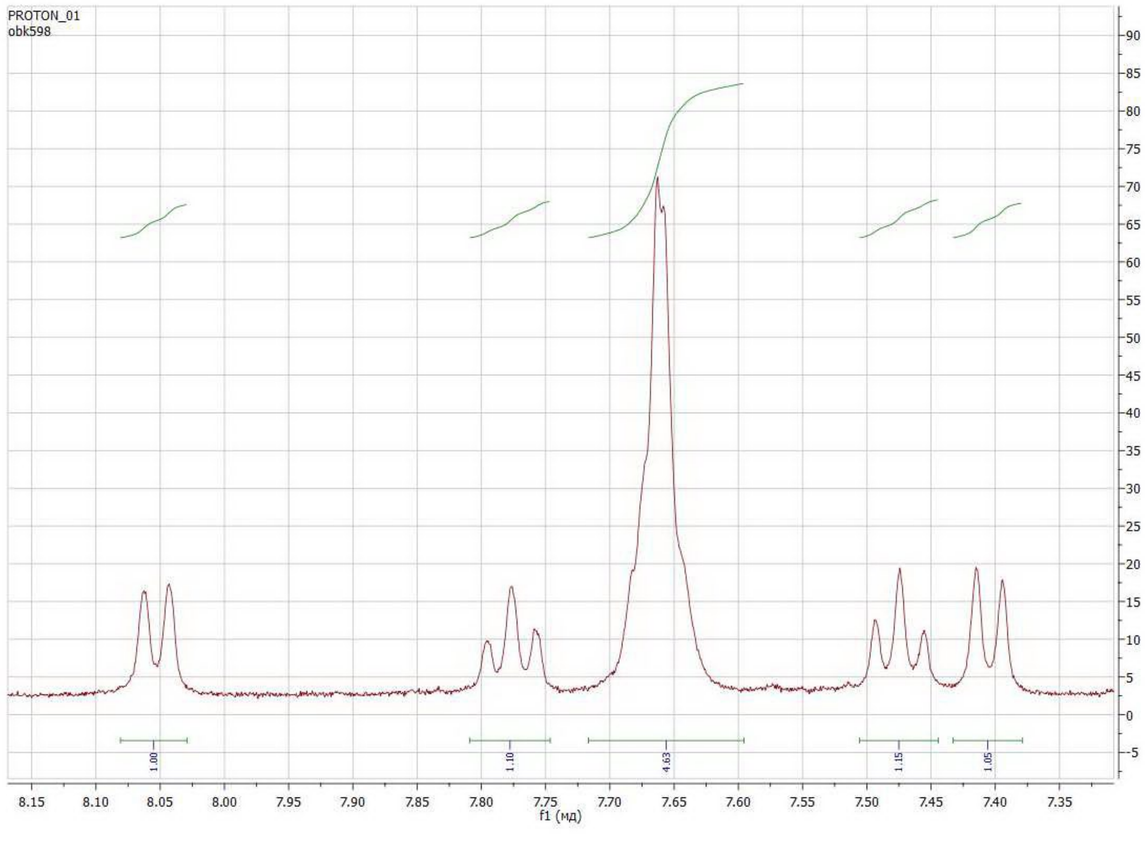

^{1}H NMR spectra of compounds **4a** (DMSO-*d*6)

Display Report

Analysis Info

Analysis Name	D:\Data\Chizhov\Osmanov\Aug_25_2021\bko-93_&clb.d
Method	tune_wide.m
Sample Name	/CHIZ BKO-93
Comment	CH3OH 100 %, dil. 20, calibrant added
Acquisition Date	25.08.2021 13:16:50
Operator	BDAL@DE
Instrument / Ser#	micrOTOF 10248

Acquisition Parameter

Source Type	ESI	Ion Polarity	Positive	Set Nebulizer	0.4 Bar
Focus	Not active			Set Dry Heater	180 °C
Scan Begin	50 m/z	Set Capillary	4500 V	Set Dry Gas	4.0 l/min
Scan End	3000 m/z	Set End Plate Offset	-500 V	Set Divert Valve	Waste

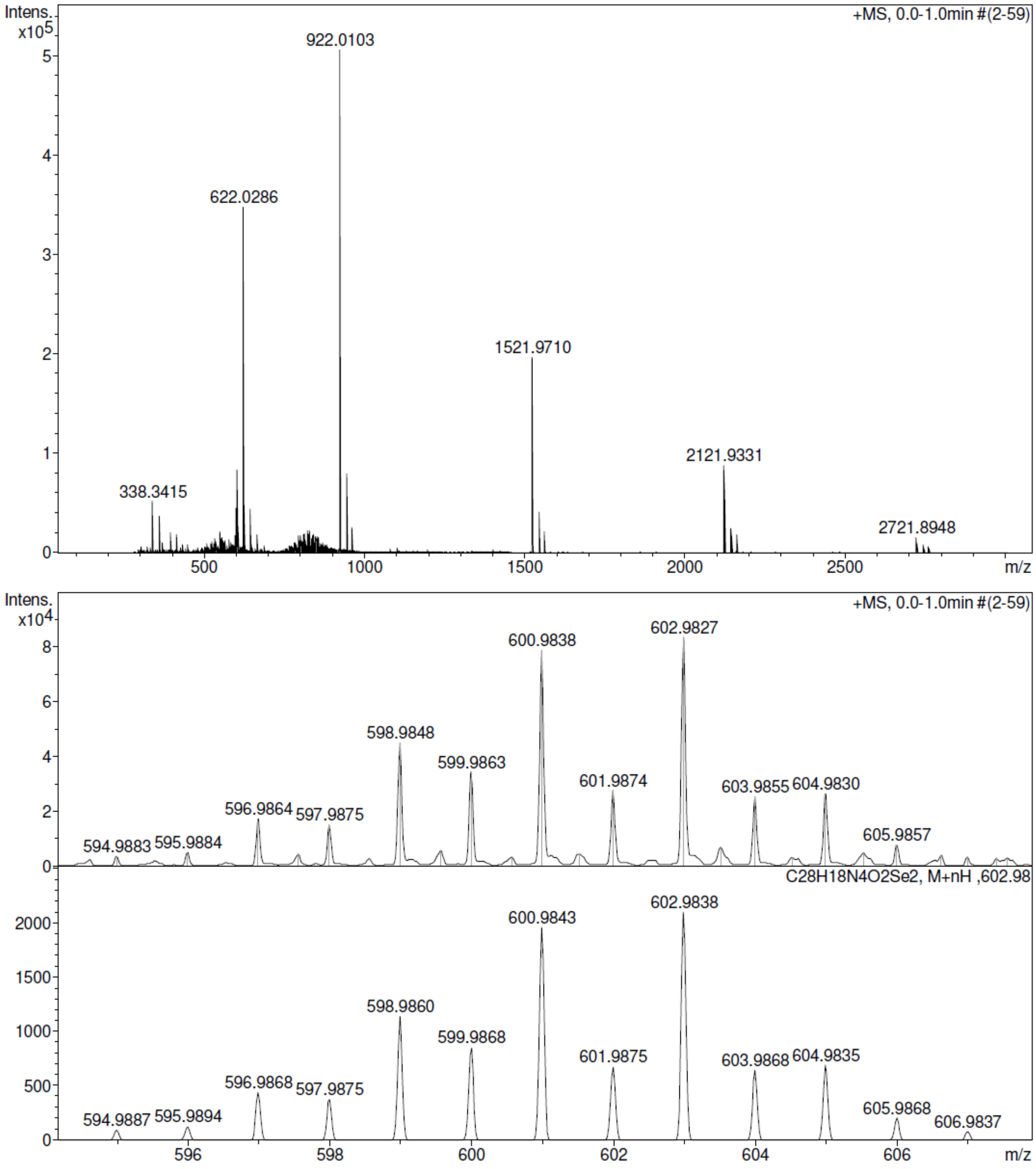

High resolution mass spectra (HR MS) of compounds **4a**

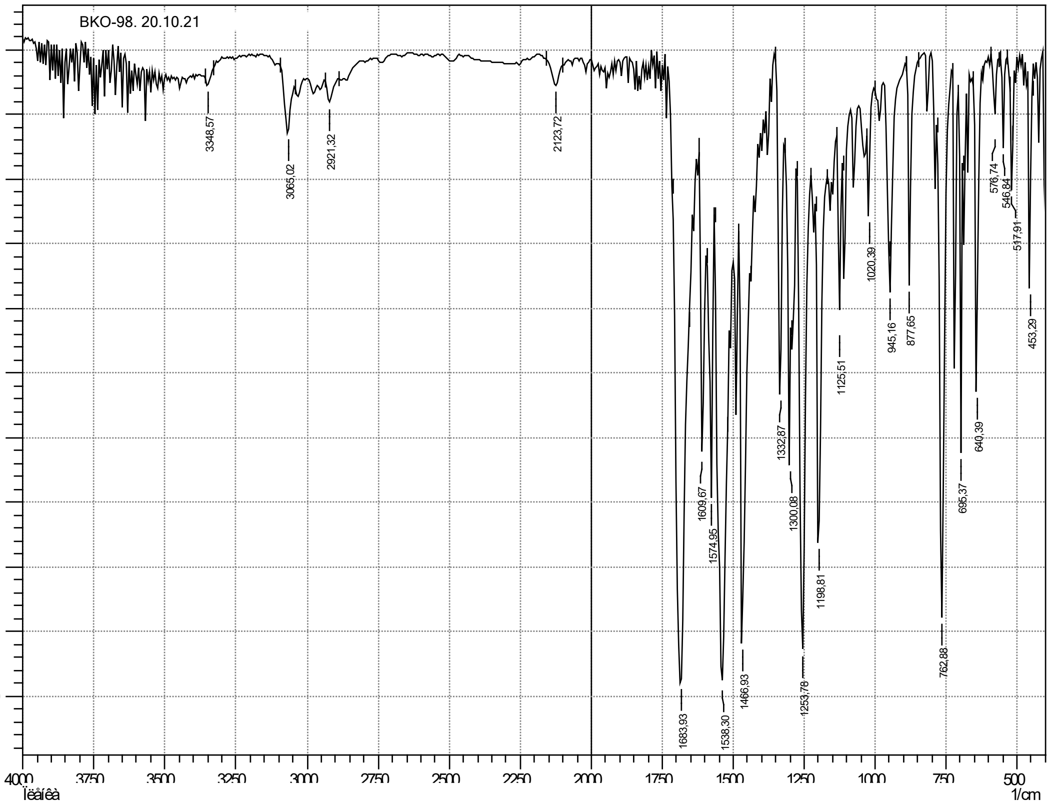

FTIR spectra of compounds **4b**

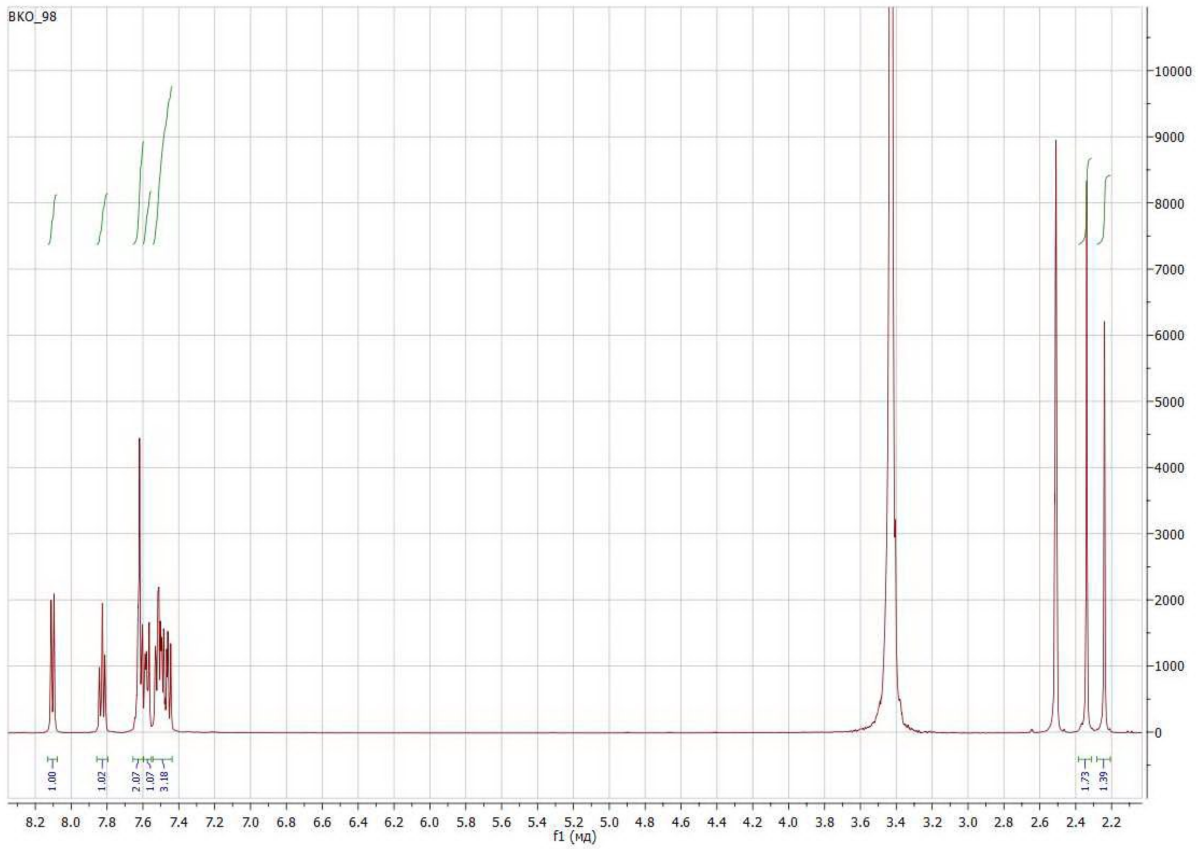

^{1}H NMR spectra of compounds **4b** (DMSO-*d*6)

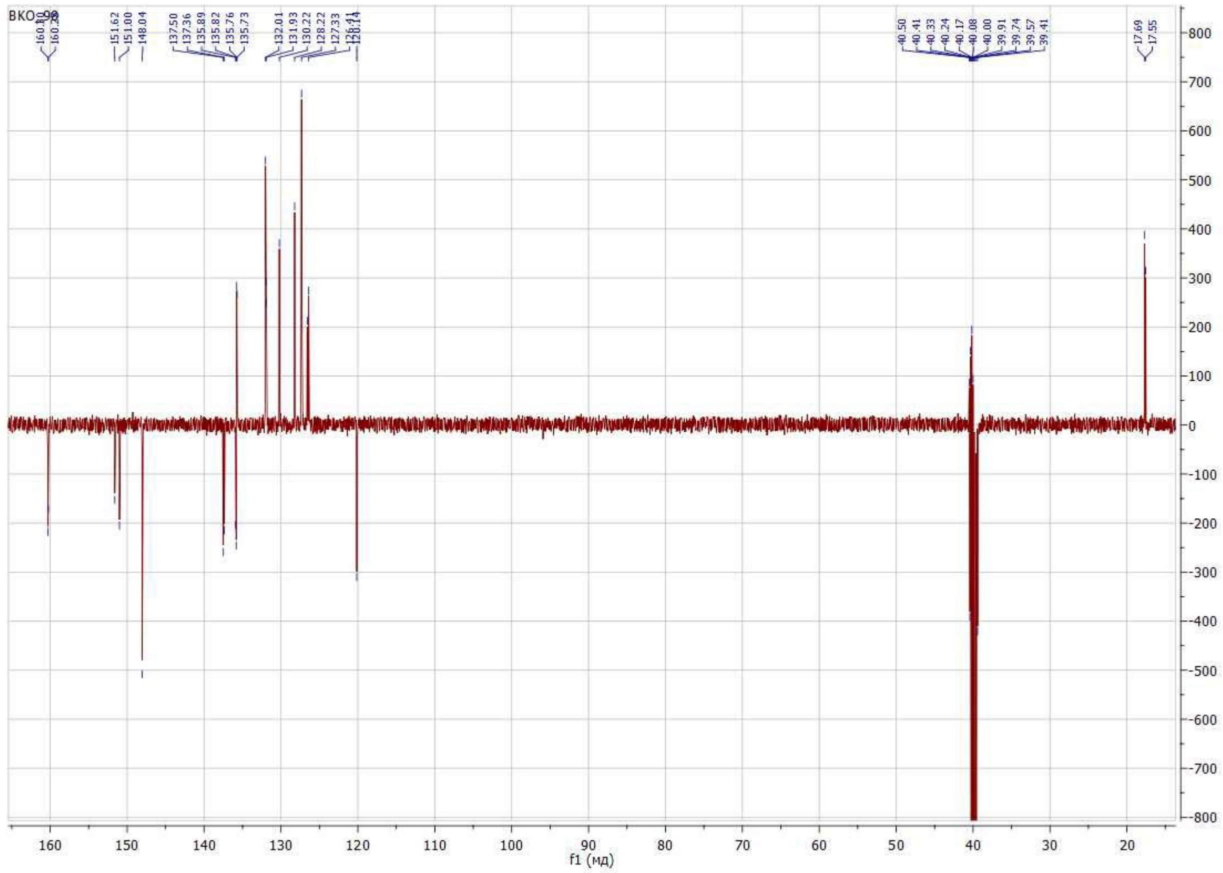

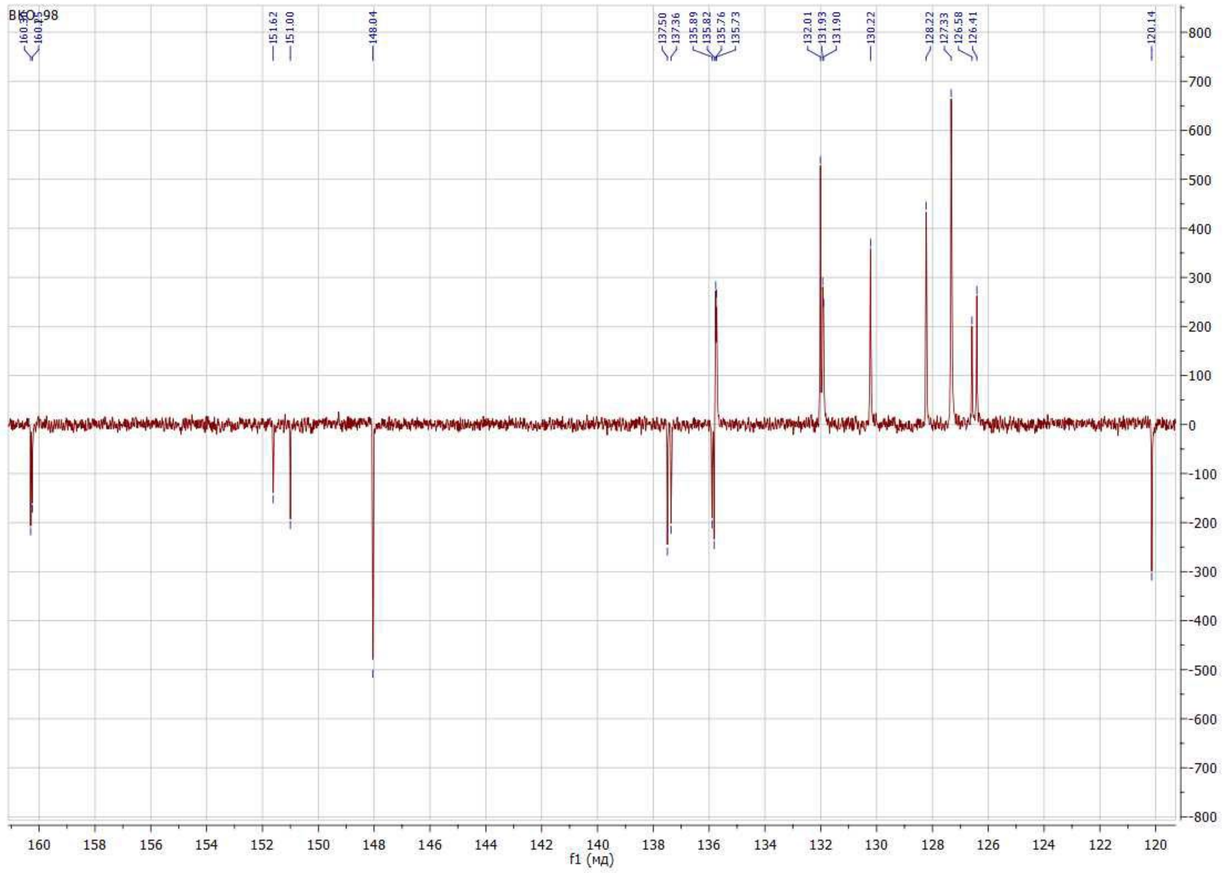

^{13}C NMR spectra of compounds **4b** (DMSO-*d*6)

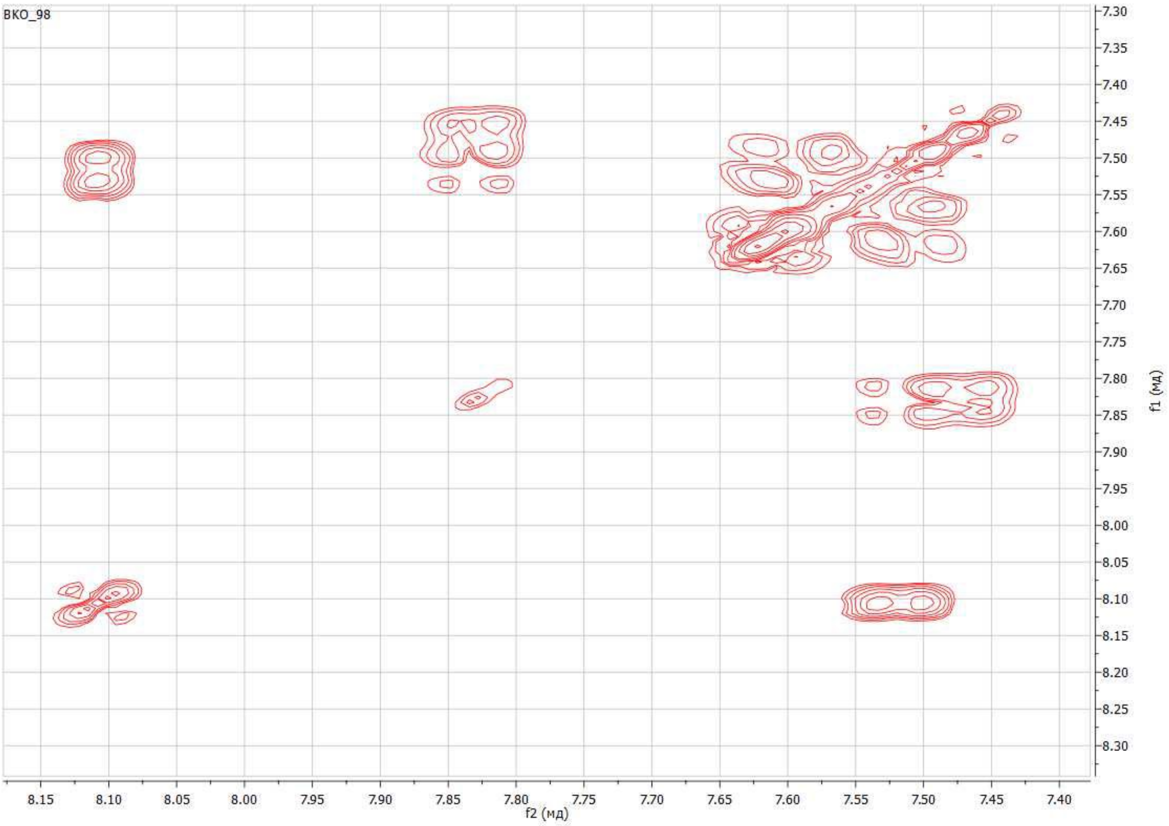

COSY NMR spectra of compounds **4b** (DMSO-*d*6)

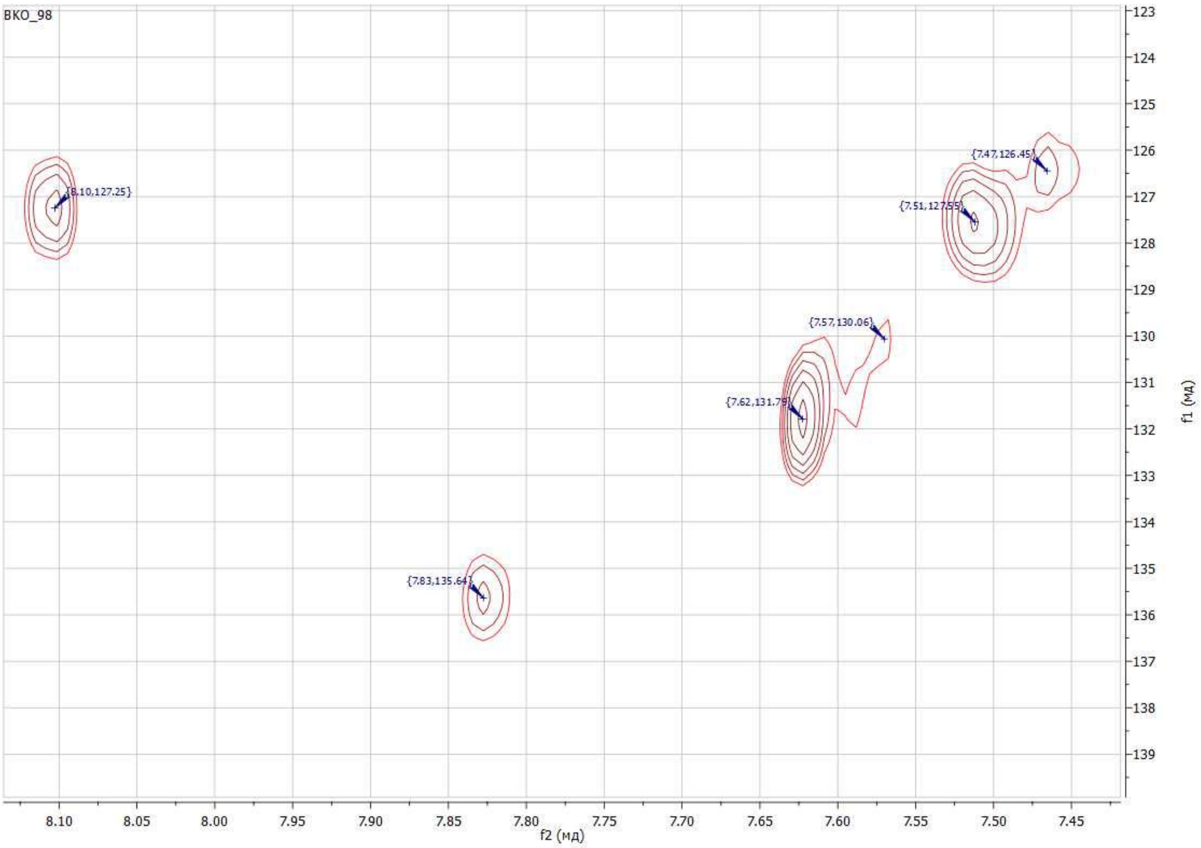

HSQC ^{1}H-^{13}C NMR spectra of compounds **4b** (DMSO-*d*6)

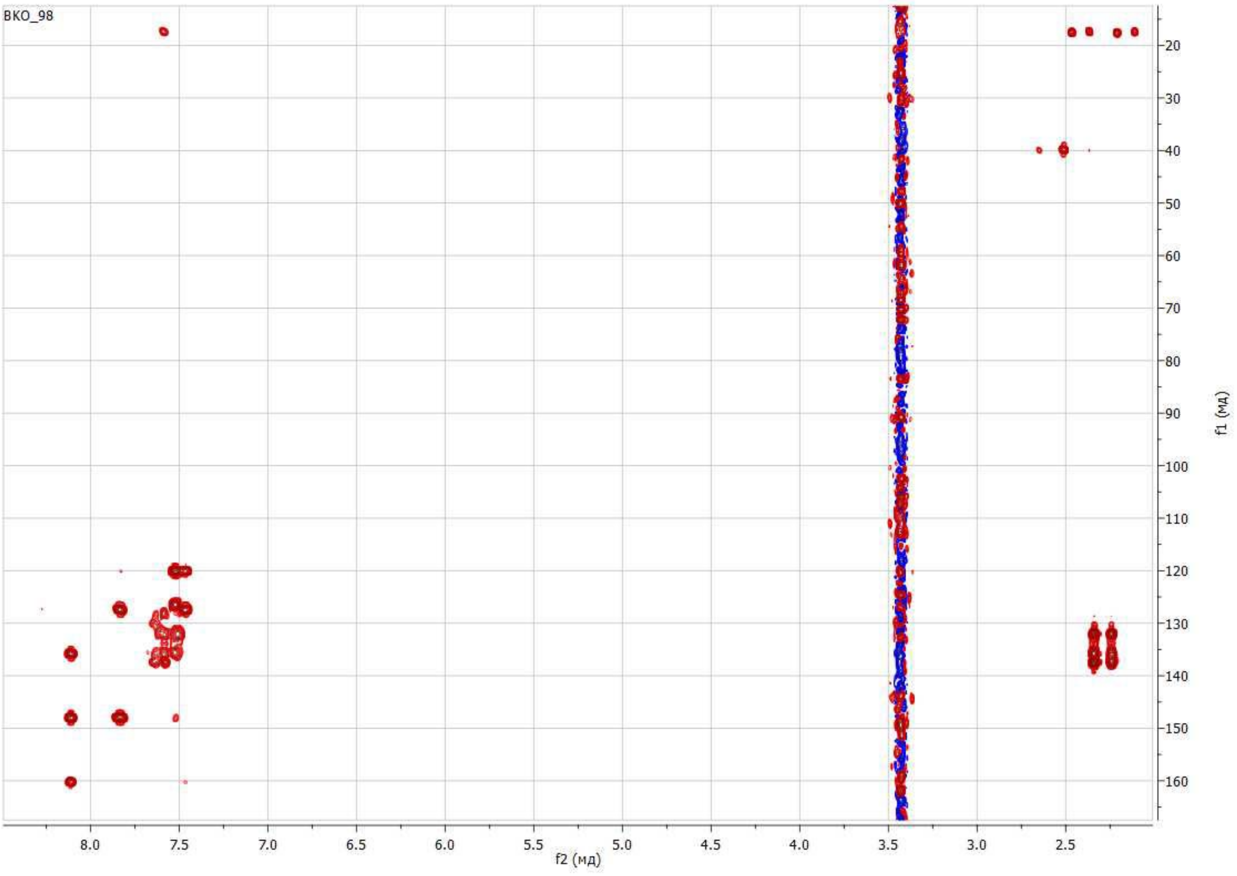

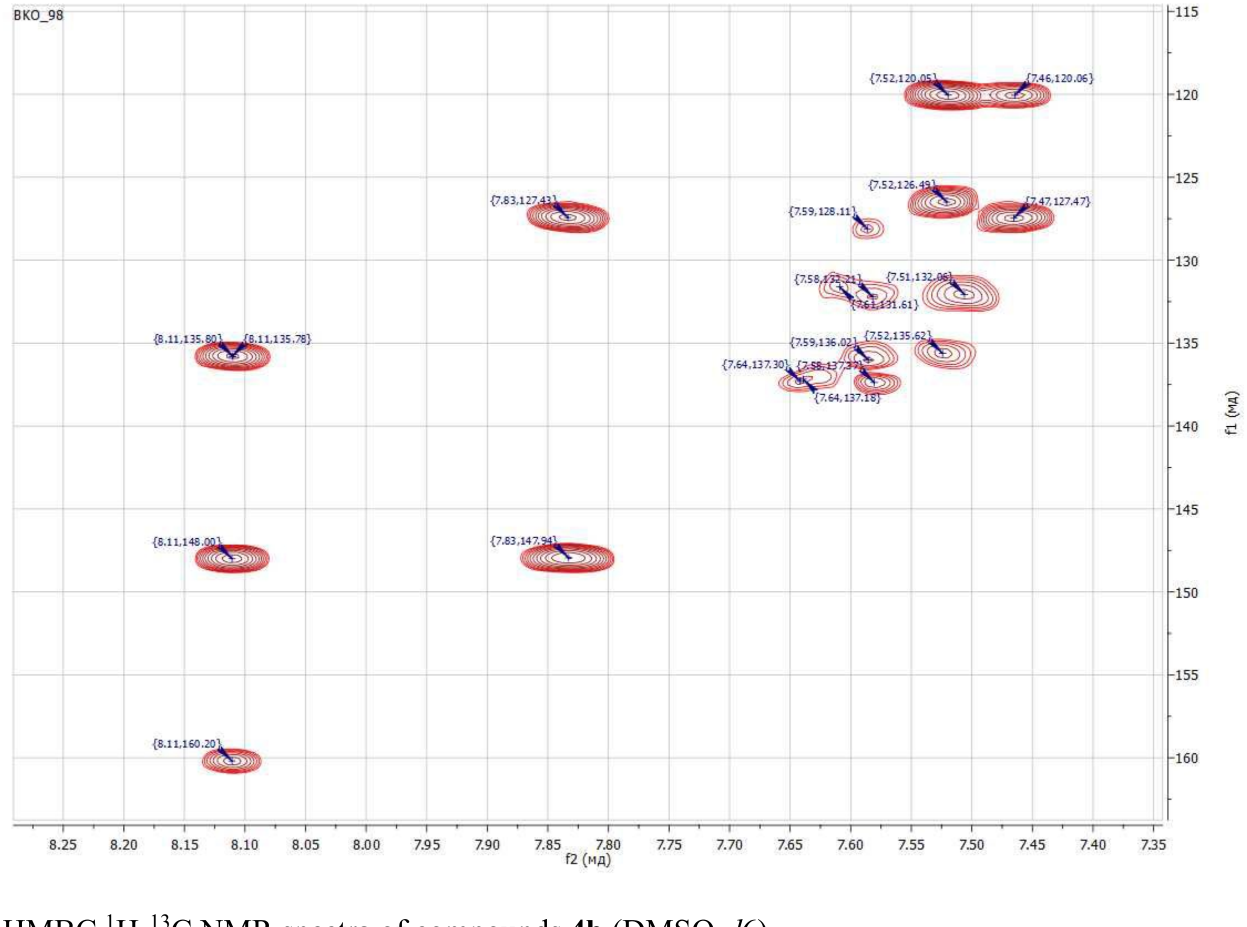

HMBC ^{1}H-^{13}C NMR spectra of compounds **4b** (DMSO-*d*6)

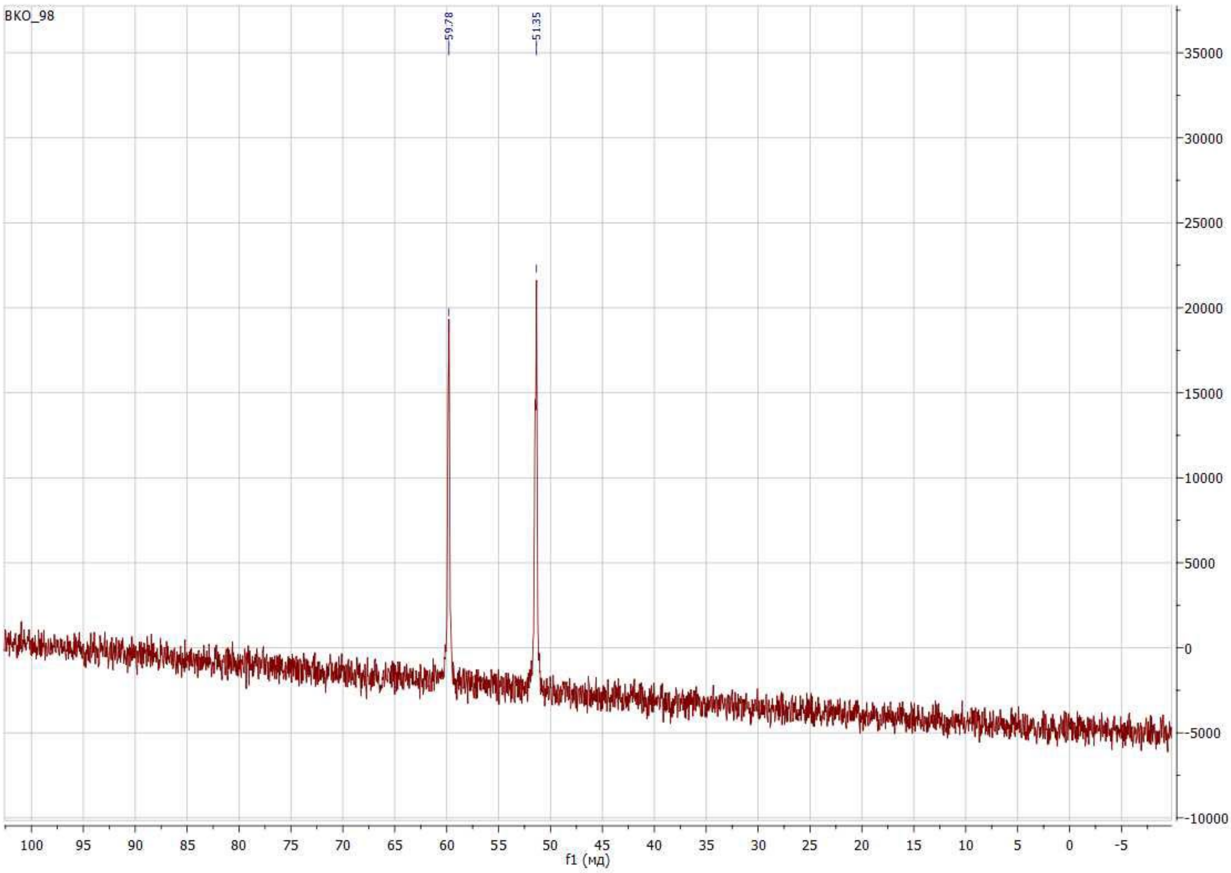

^{77}Se NMR spectra of compounds **4b** (DMSO-*d6*)

Display Report

Analysis Info

Analysis Name	D:\Data\Chizhov\Osmanov\Aug_25_2021\bko-98_&clb.d	Acquisition Date	25.08.2021 13:23:22
Method	tune_wide.m	Operator	BDAL@DE
Sample Name	/CHIZ BKO-98	Instrument / Ser#	micrOTOF 10248
Comment	CH3OH 100 %, dil. 200, calibrant added		

Acquisition Parameter

Source Type	ESI	Ion Polarity	Positive	Set Nebulizer	0.4 Bar
Focus	Not active			Set Dry Heater	180 °C
Scan Begin	50 m/z	Set Capillary	4500 V	Set Dry Gas	4.0 l/min
Scan End	3000 m/z	Set End Plate Offset	-500 V	Set Divert Valve	Waste

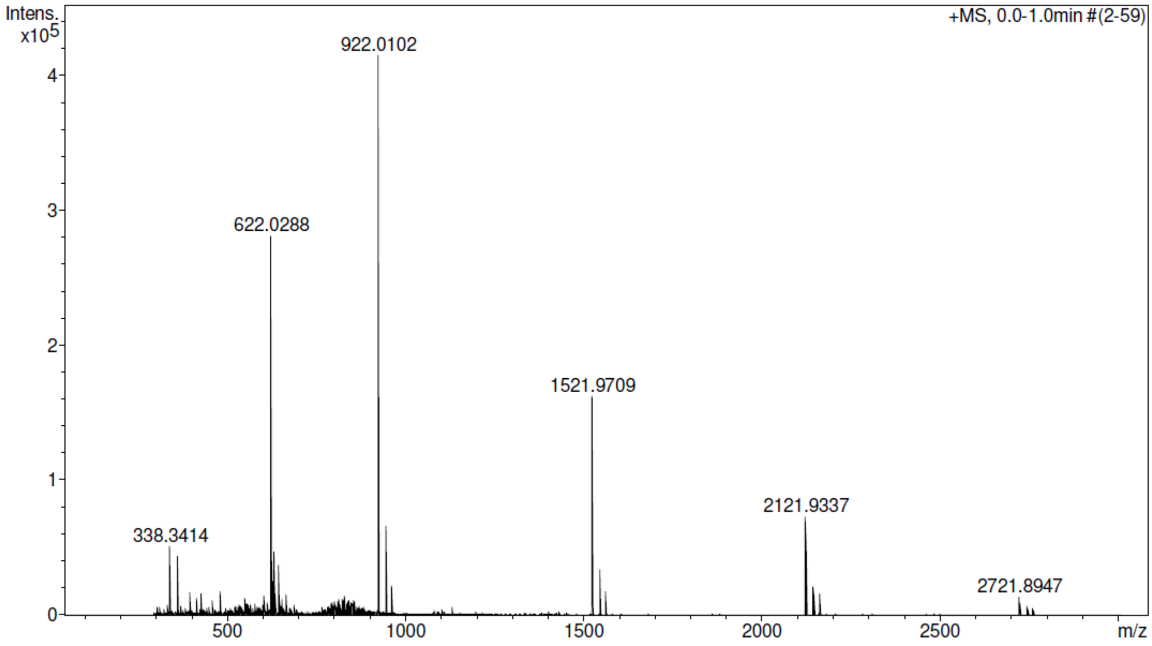

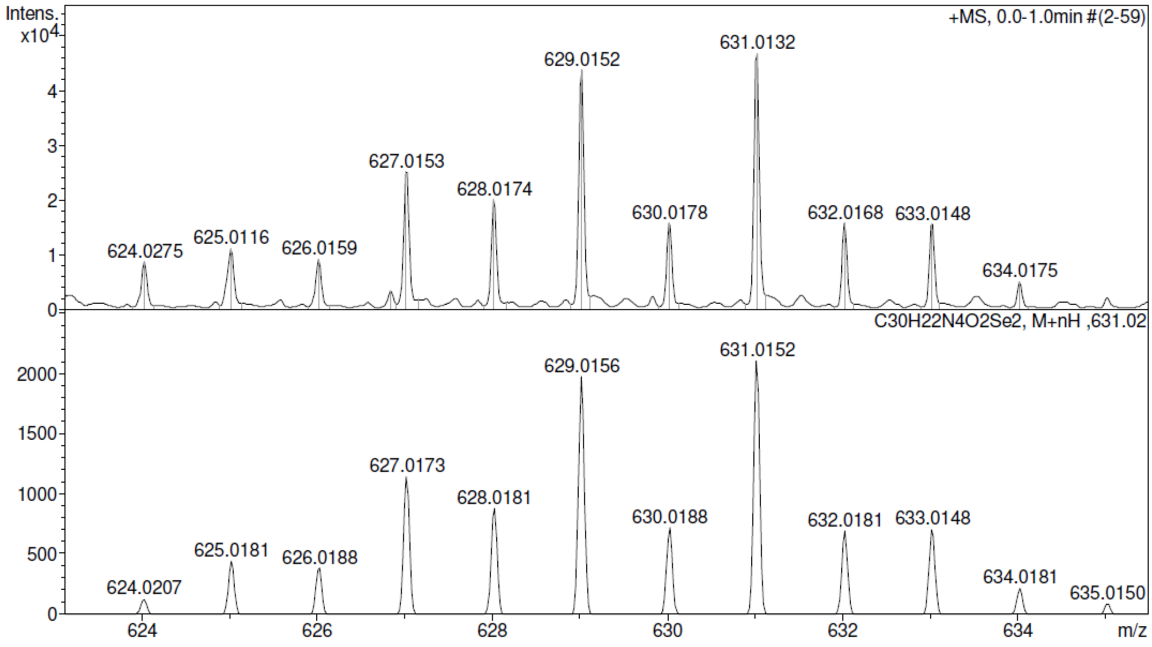

High resolution mass spectra (HR MS) of compounds **4b**

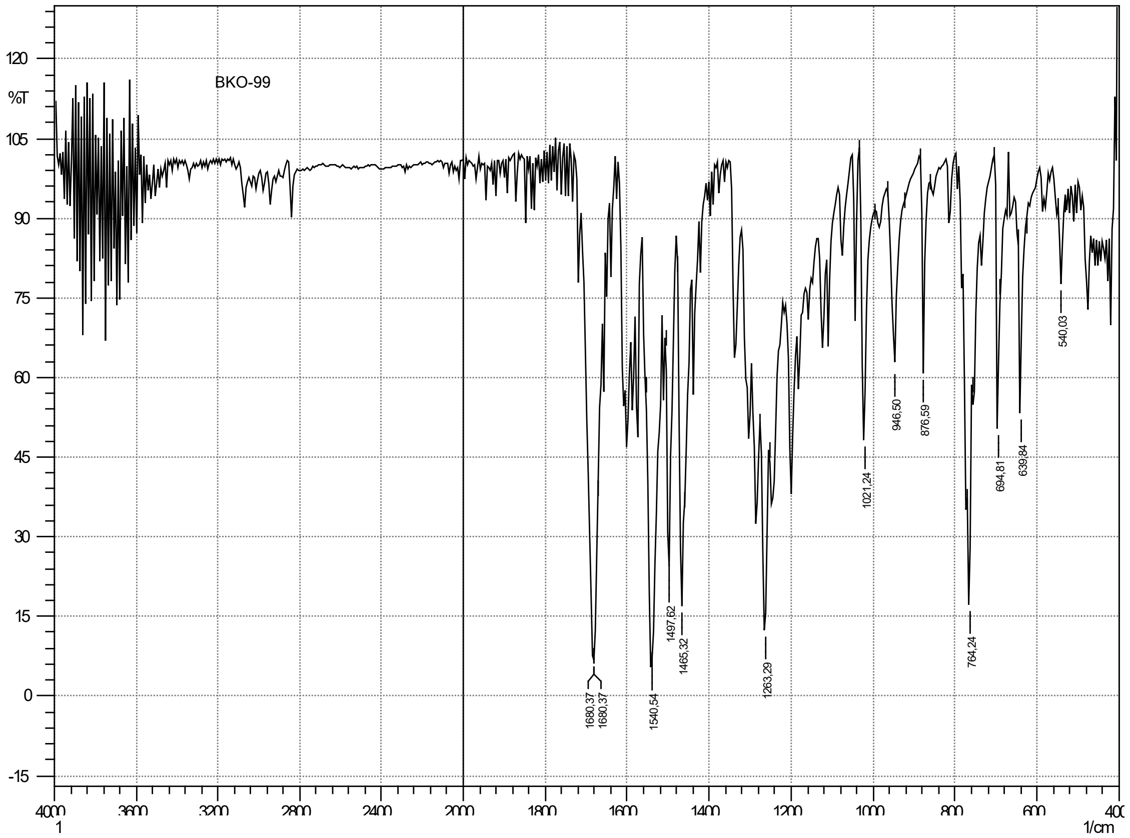

FTIR spectra of compounds **4c**

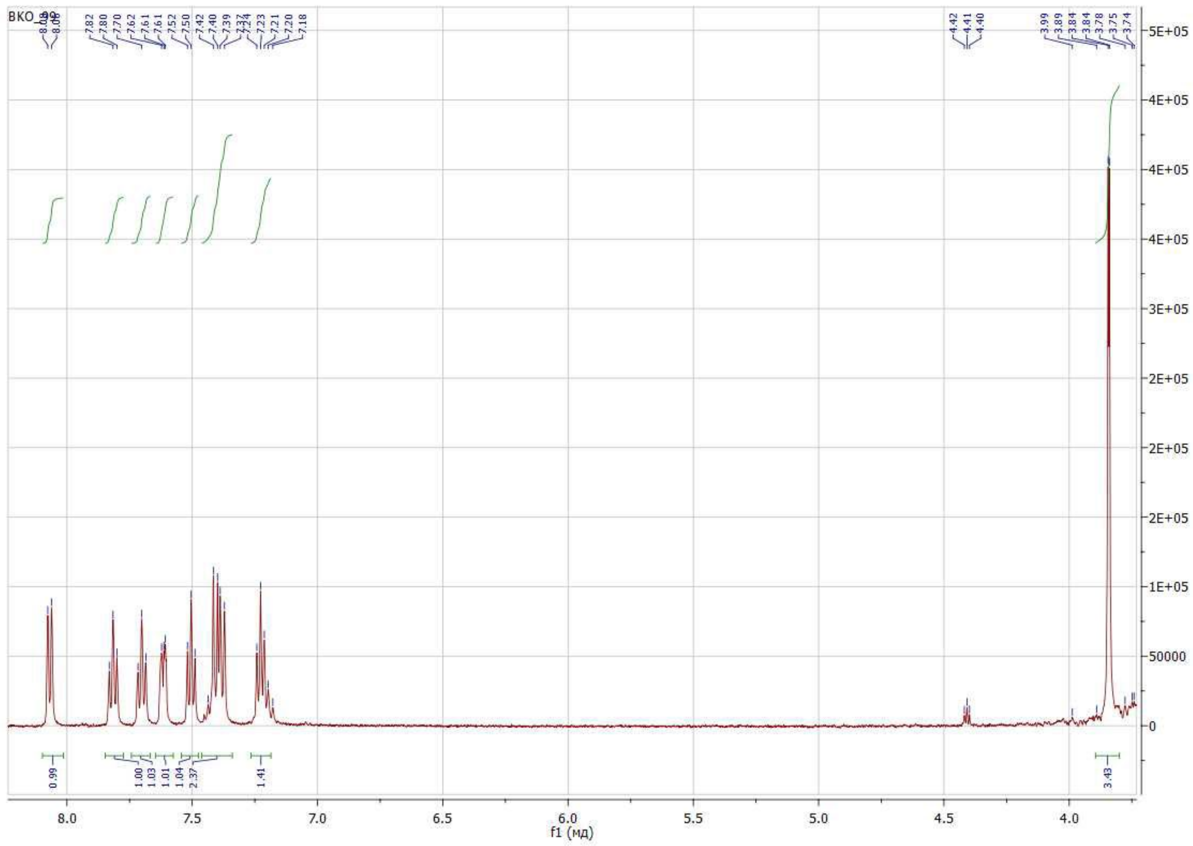

^{1}H NMR spectra of compounds **4c** (DMSO-*d*6)

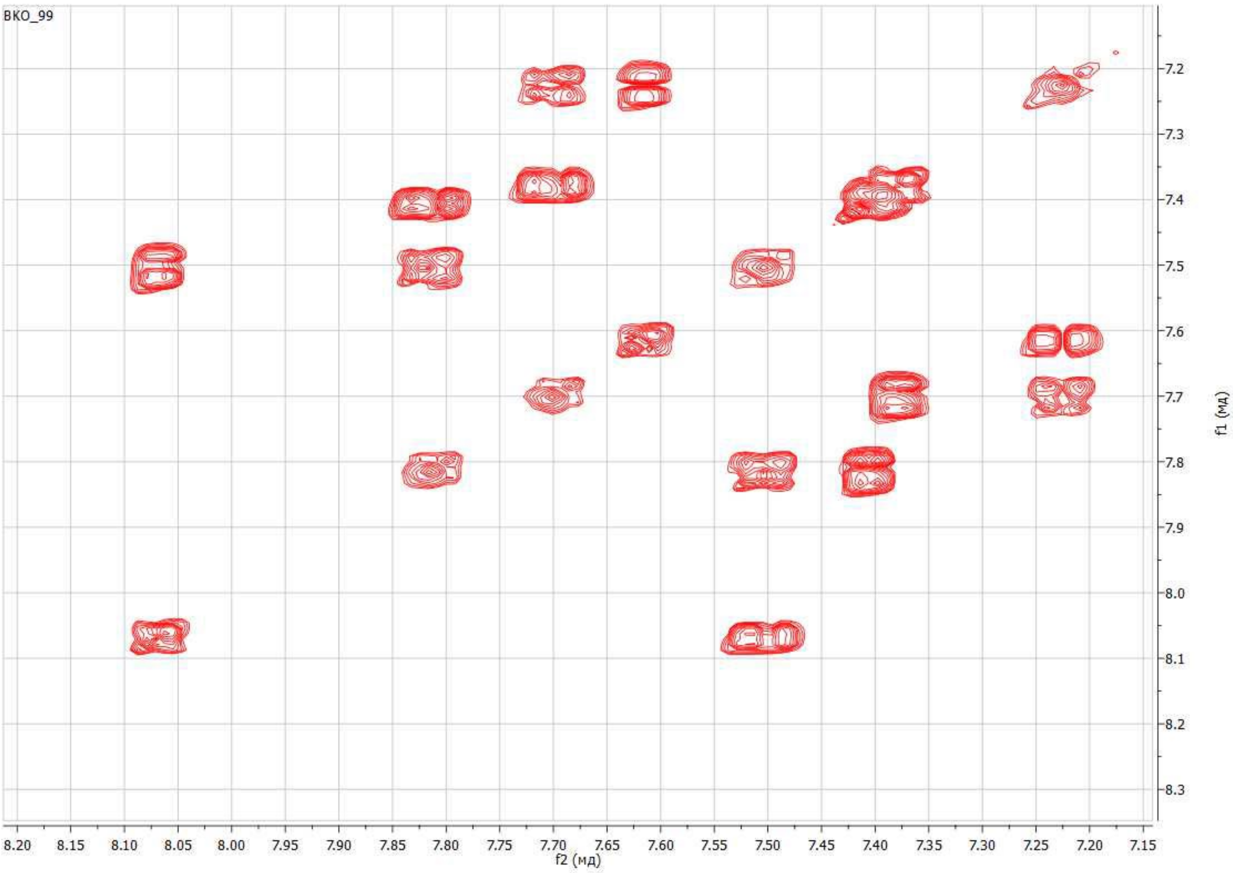

COSY NMR spectra of compounds **4c** (DMSO-*d*6)

Display Report

Analysis Info

Analysis Name	D:\Data\Chizhov\Osmanov\Aug_25_2021\bko-99_&clb.d	Acquisition Date	25.08.2021 13:41:49
Method	tune_wide.m	Operator	BDAL@DE
Sample Name	/CHIZ BKO-99	Instrument / Ser#	micrOTOF 10248
Comment	CH3OH 100 %, dil. 20, calibrant added		

Acquisition Parameter

Source Type	ESI	Ion Polarity	Positive	Set Nebulizer	0.4 Bar
Focus	Not active			Set Dry Heater	180 °C
Scan Begin	50 m/z	Set Capillary	4500 V	Set Dry Gas	4.0 l/min
Scan End	3000 m/z	Set End Plate Offset	-500 V	Set Divert Valve	Waste

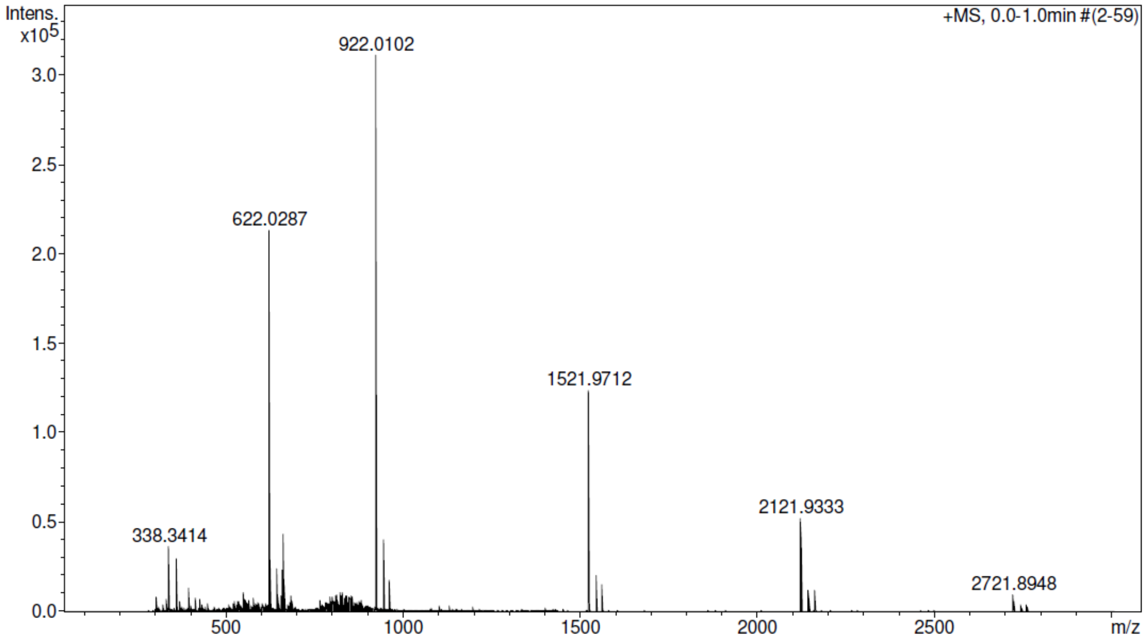

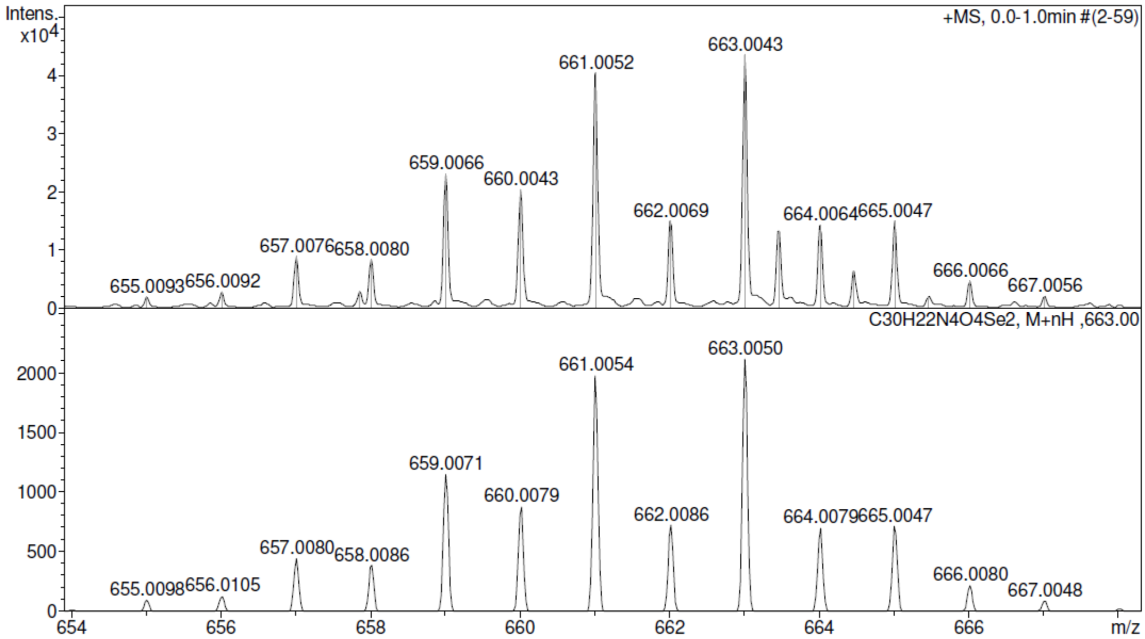

High resolution mass spectra (HR MS) of compounds **4c**

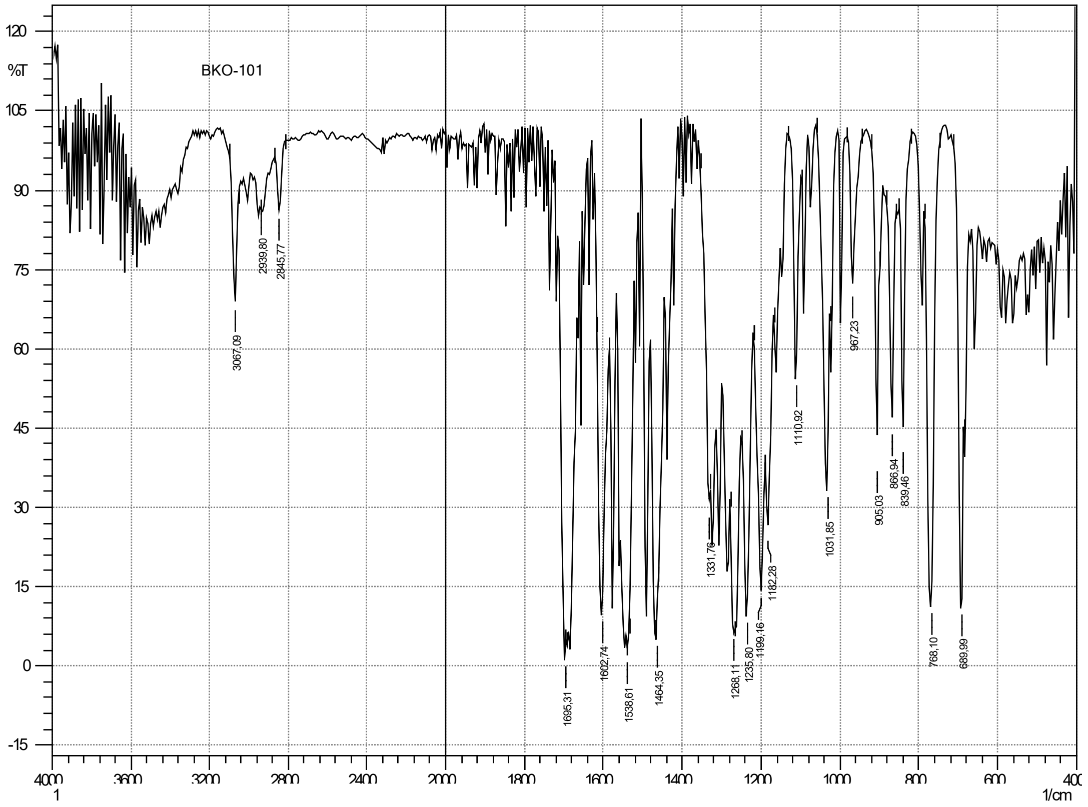

FTIR spectra of compounds **4d**

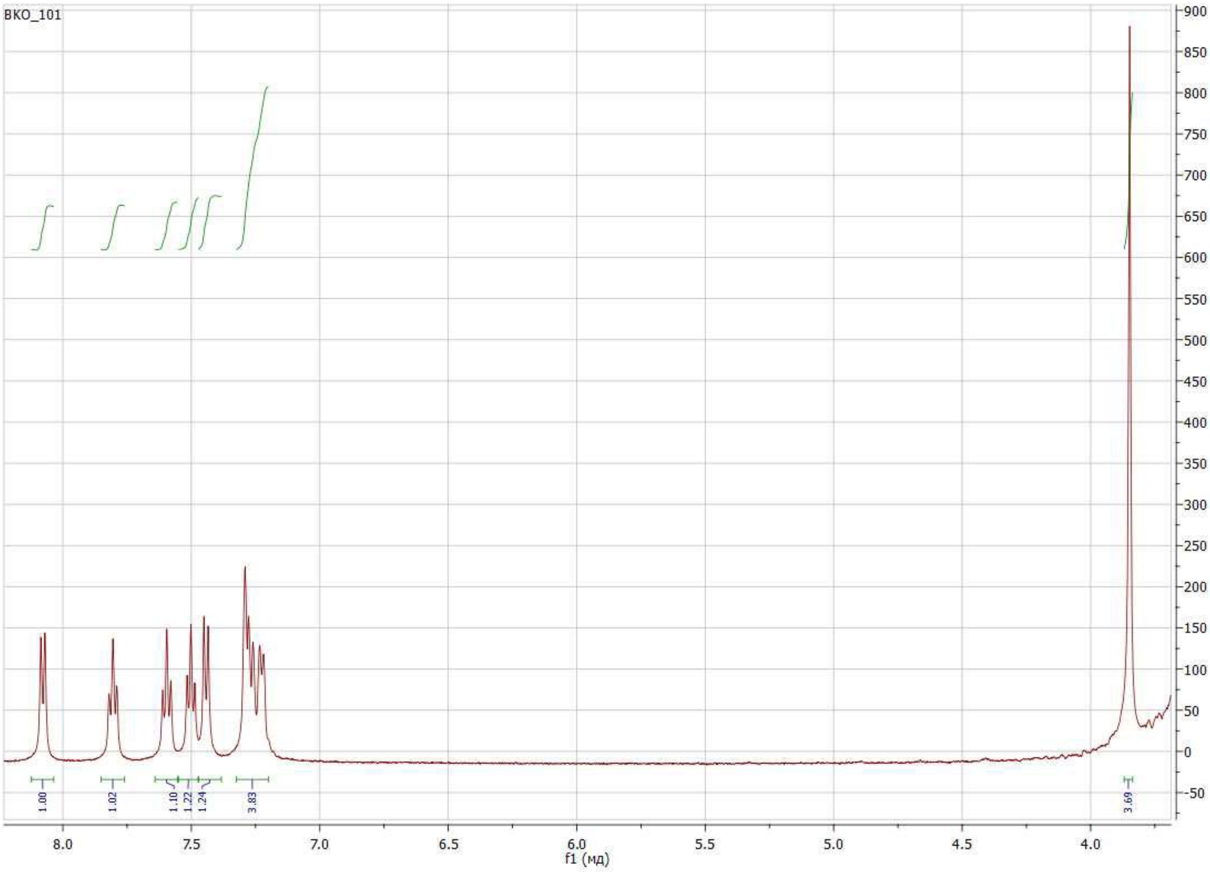

^{1}H NMR spectra of compounds **4d** (DMSO-*d*6)

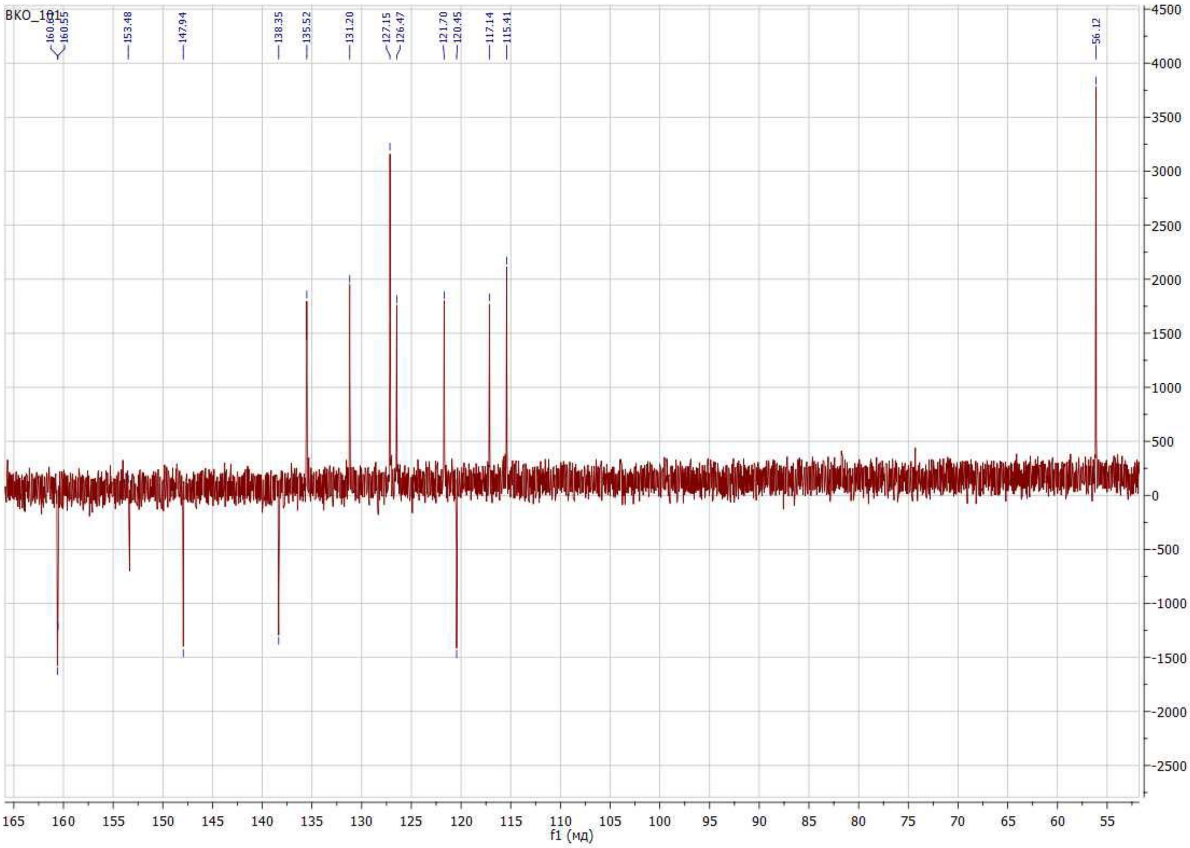

^{13}C NMR spectra of compounds **4d** (DMSO-*d*6)

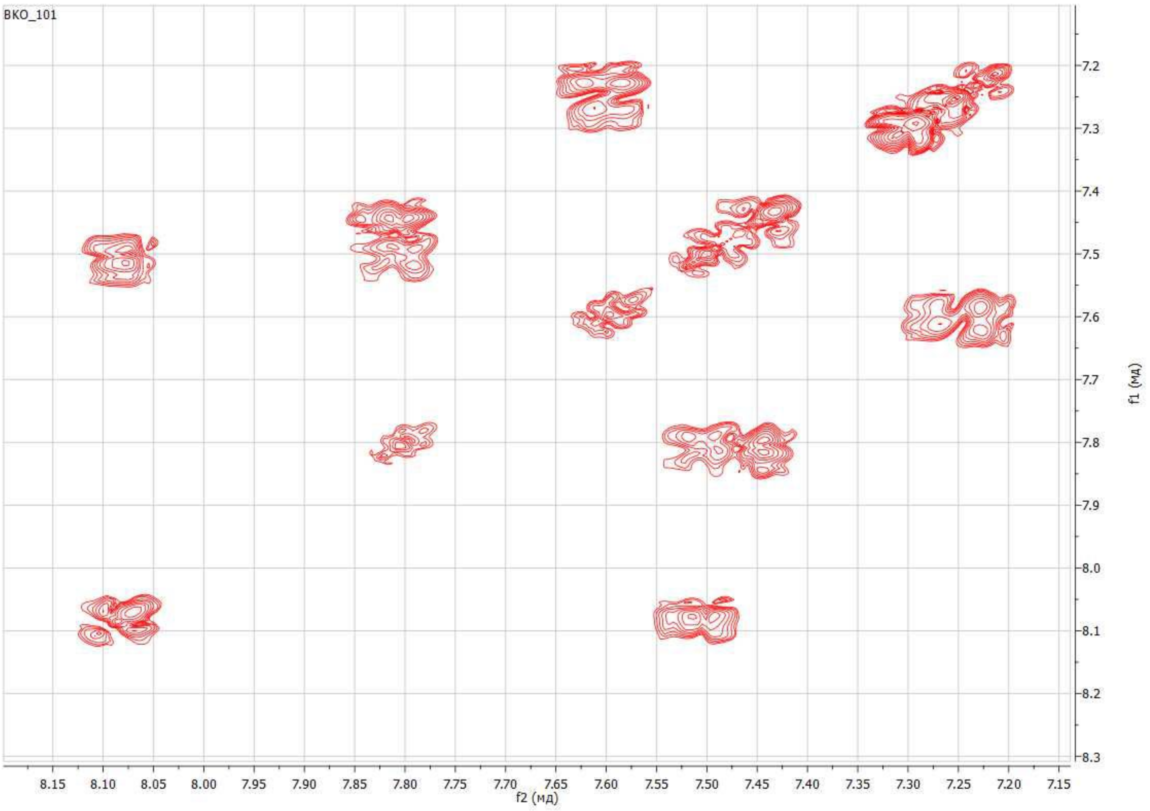

COSY NMR spectra of compounds **4d** (DMSO-*d*6)

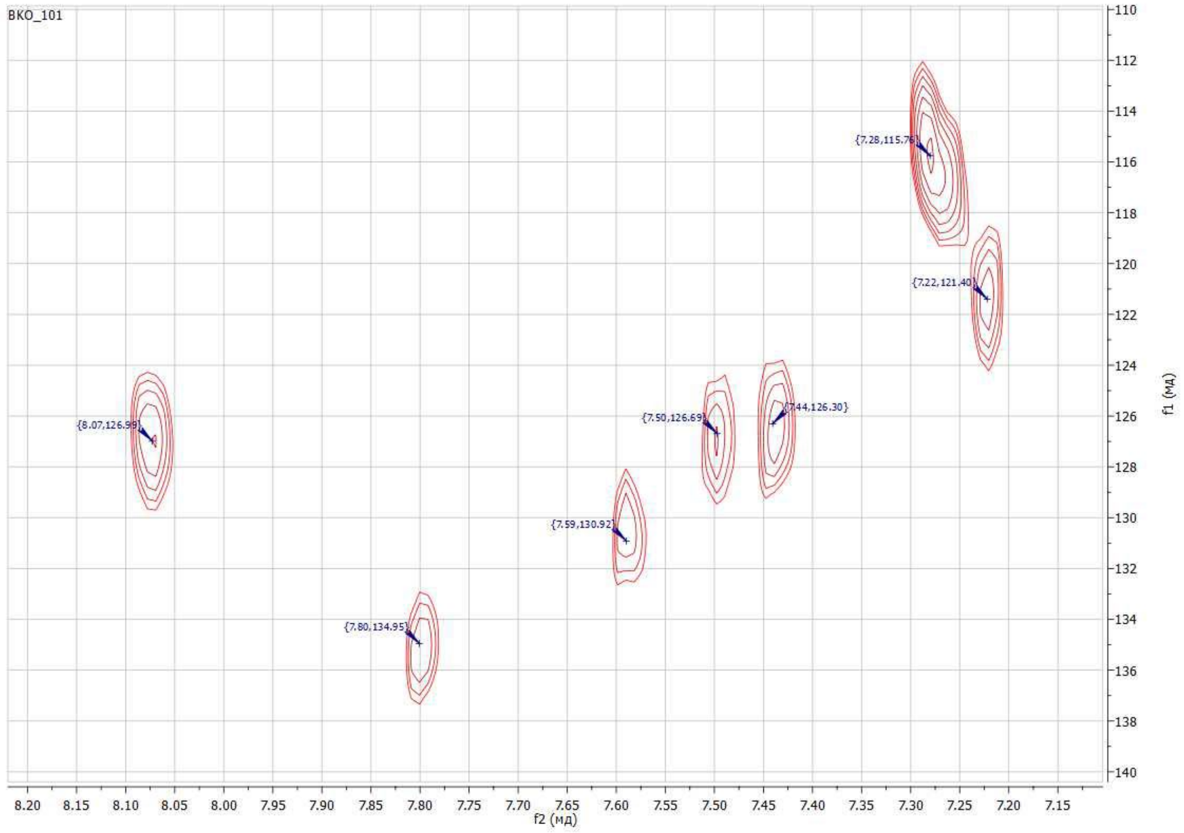

HSQC ^{1}H-^{13}C NMR spectra of compounds **4d** (DMSO-*d*6)

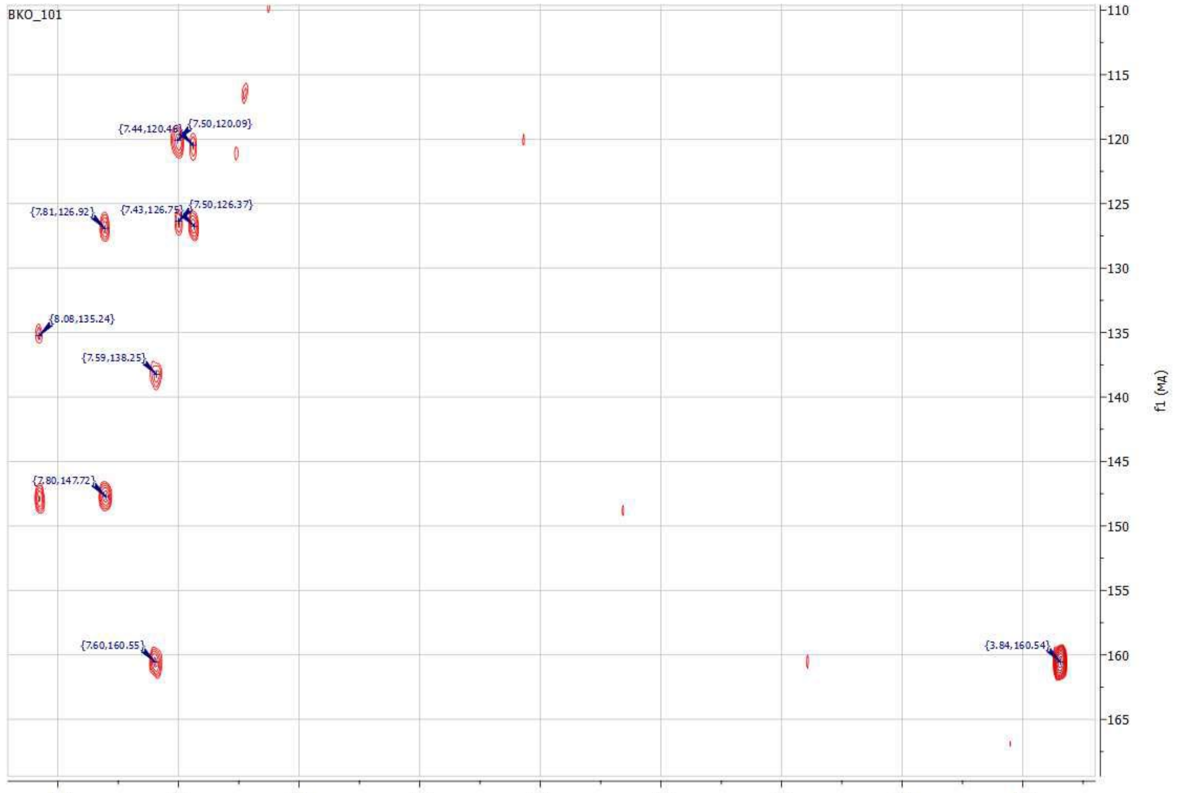

HMBC ^{1}H-^{13}C NMR spectra of compounds **4d** (DMSO-*d*6)

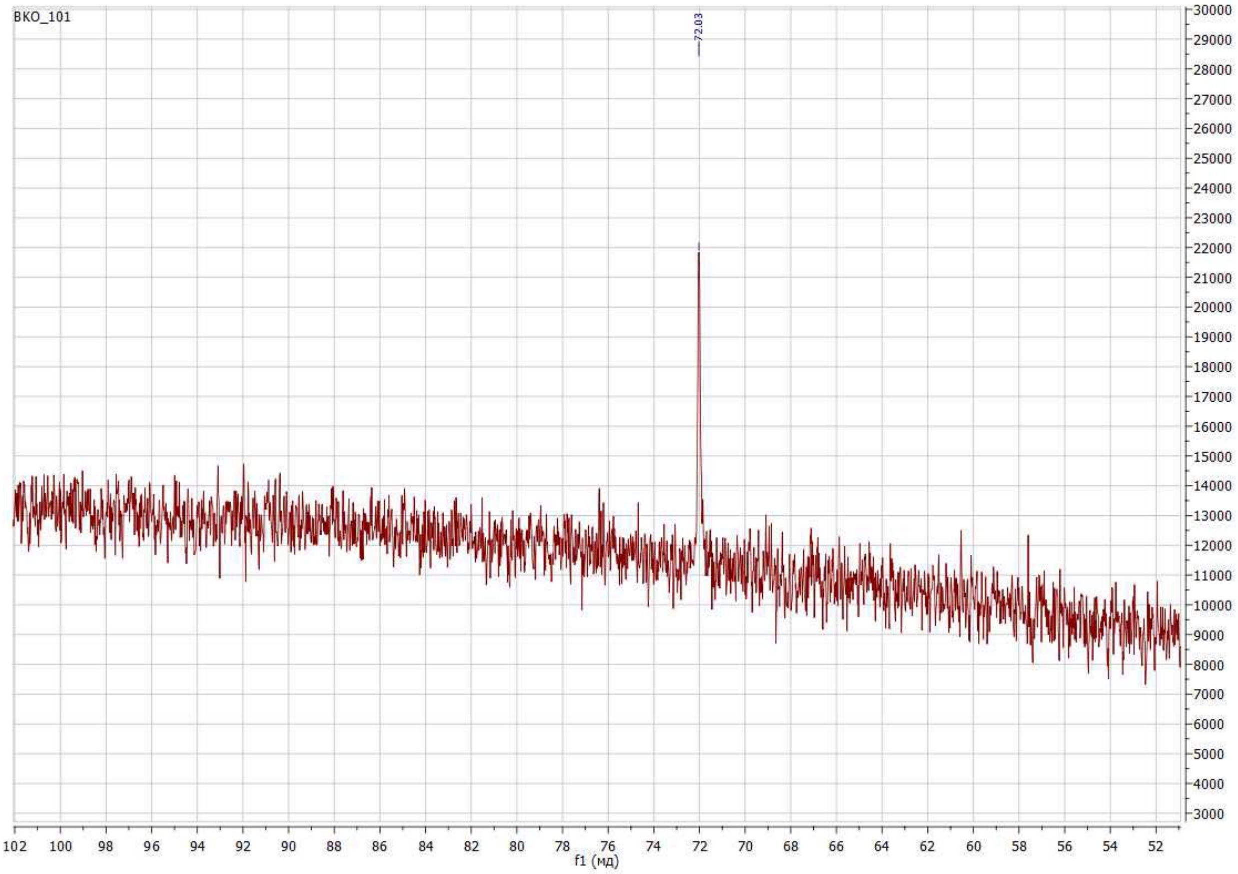

^{77}Se NMR spectra of compounds **4d** (DMSO-*d*6)

Display Report

Analysis Info

Analysis Name	D:\Data\Chizhov\Osmanov\Aug_25_2021\bko-101_&clb.d	Acquisition Date	25.08.2021 13:47:47
Method	tune_wide.m	Operator	BDAL@DE
Sample Name	/CHIZ BKO-101	Instrument / Ser#	micrOTOF 10248
Comment	CH3OH 100 %, dil. 20, calibrant added		

Acquisition Parameter

Source Type	ESI	Ion Polarity	Positive	Set Nebulizer	0.4 Bar
Focus	Not active			Set Dry Heater	180 °C
Scan Begin	50 m/z	Set Capillary	4500 V	Set Dry Gas	4.0 l/min
Scan End	3000 m/z	Set End Plate Offset	-500 V	Set Divert Valve	Waste

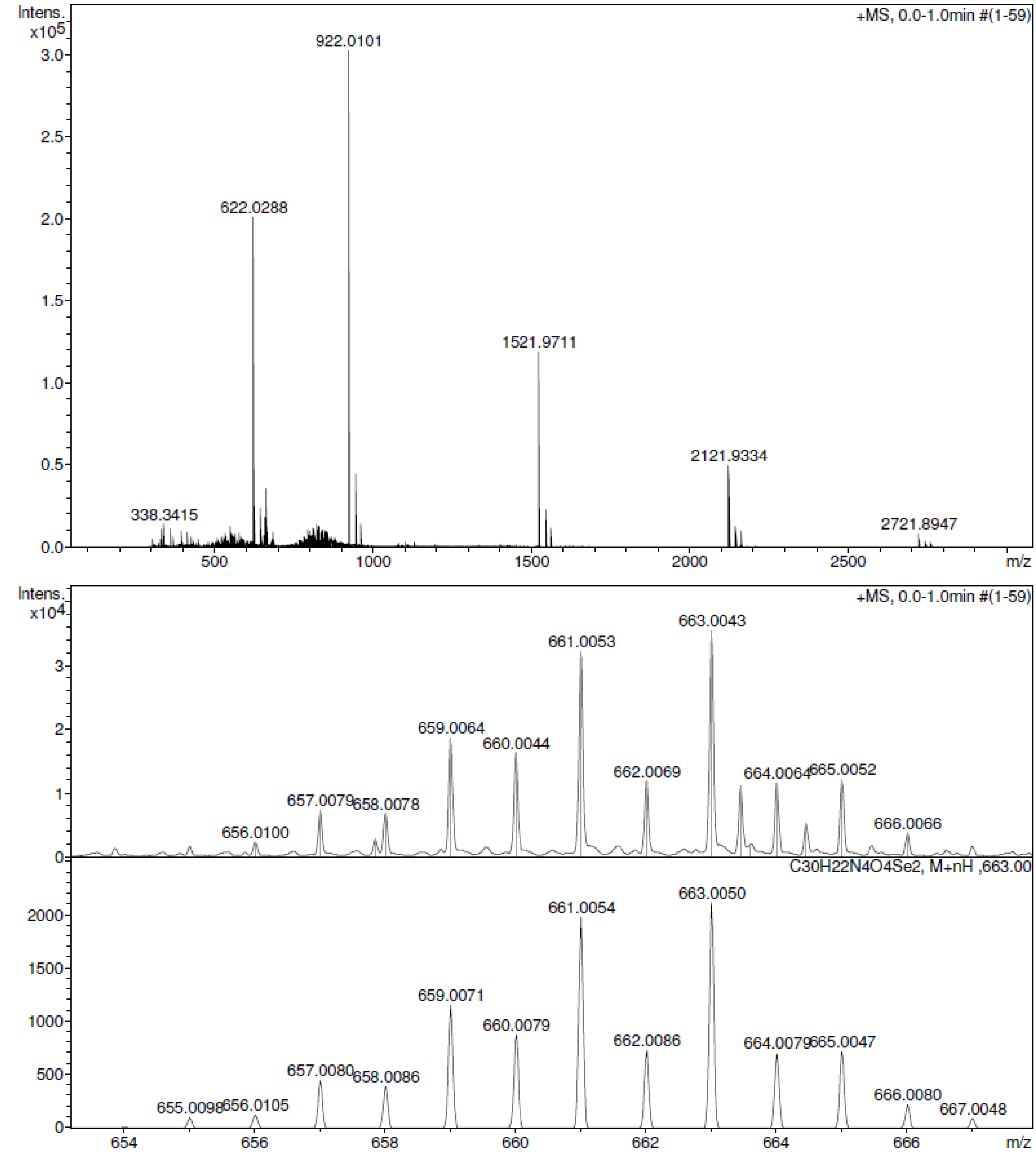

High resolution mass spectra (HR MS) of compounds **4d**

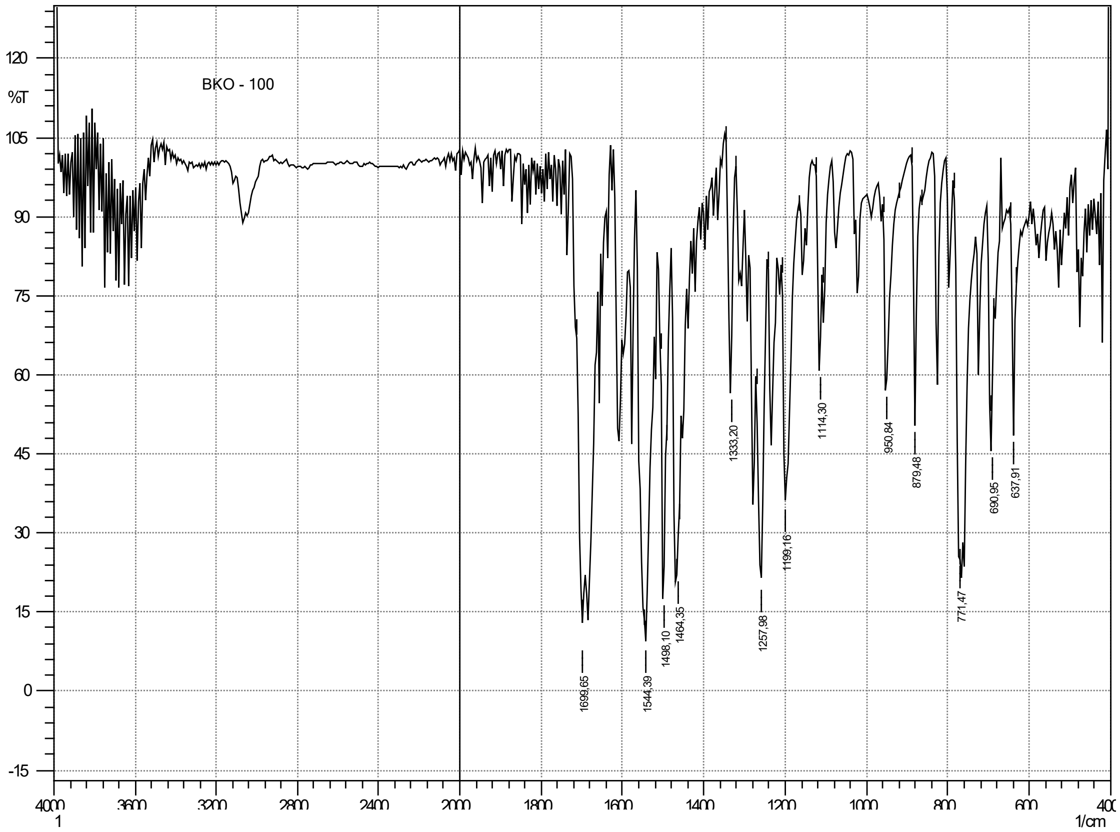

FTIR spectra of compounds **4e**

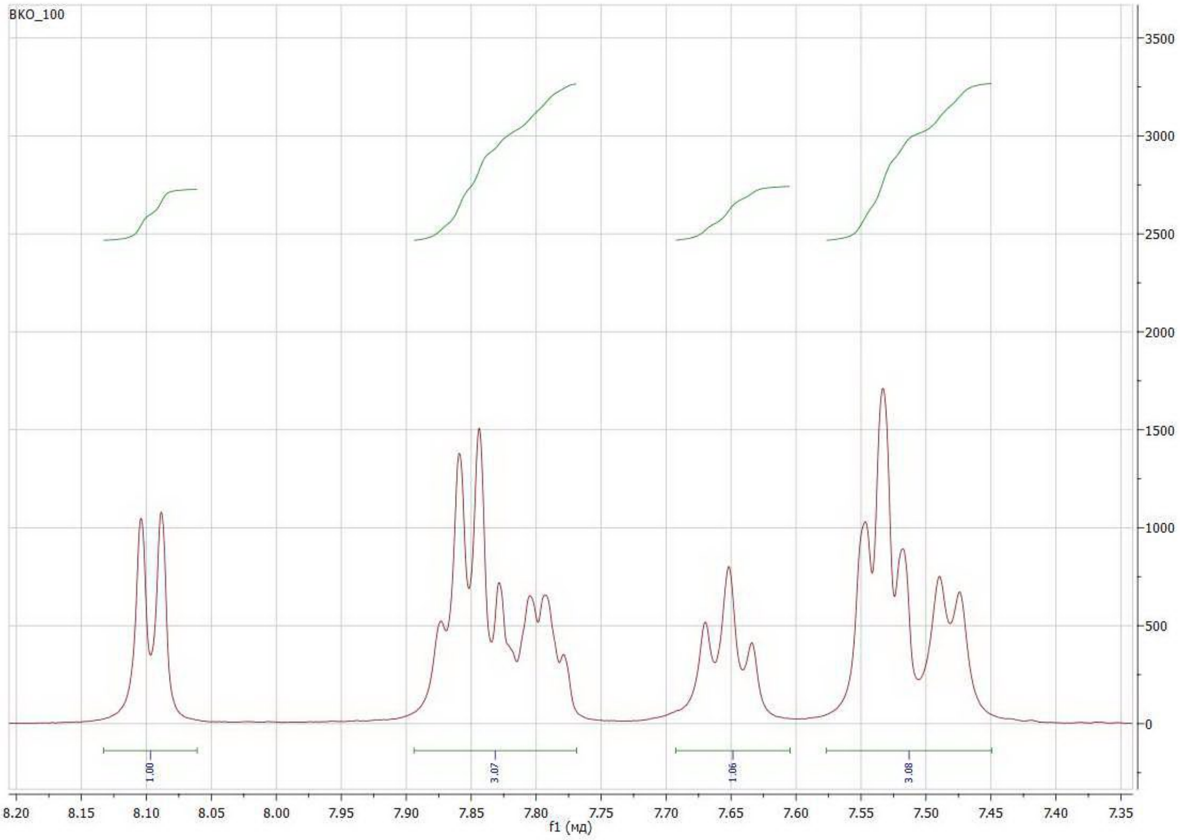

^{1}H NMR spectra of compounds **4e** (DMSO-*d*6)

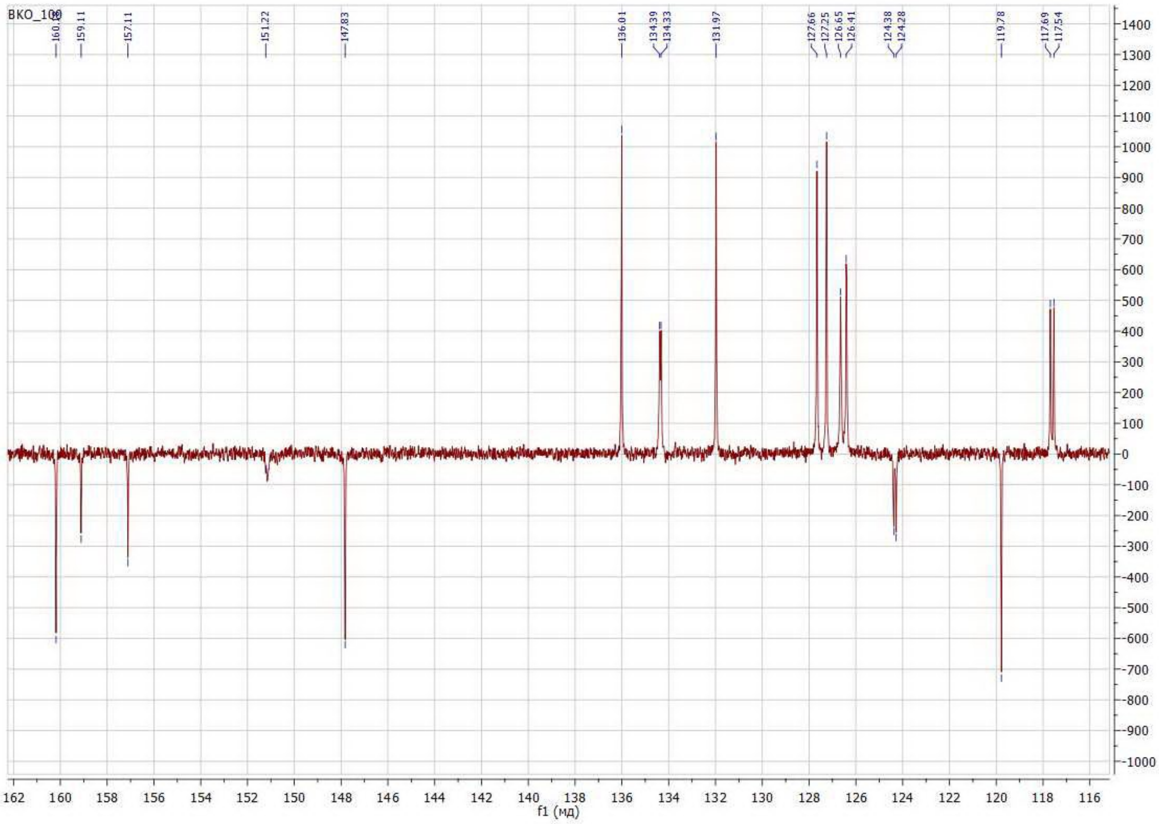

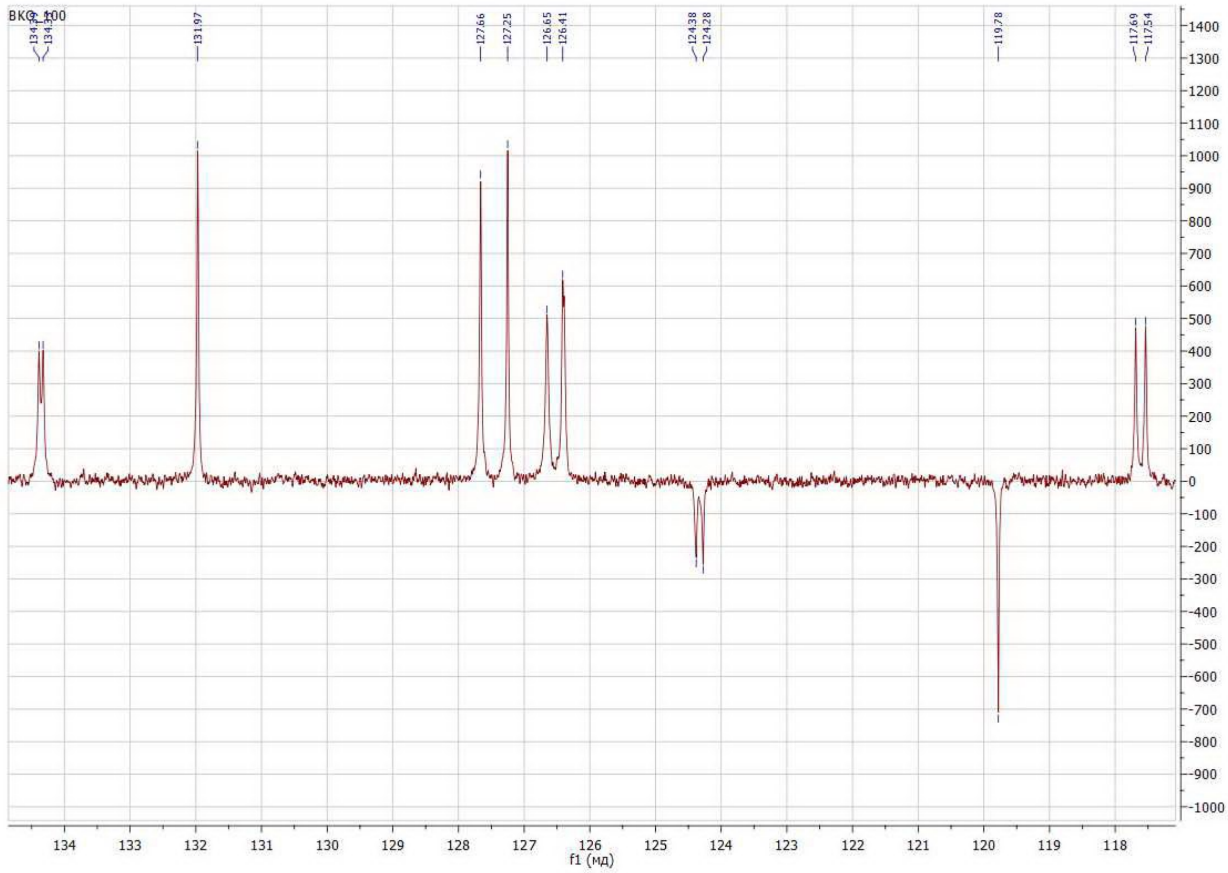

^{13}C NMR spectra of compounds **4e** (DMSO-*d*6)

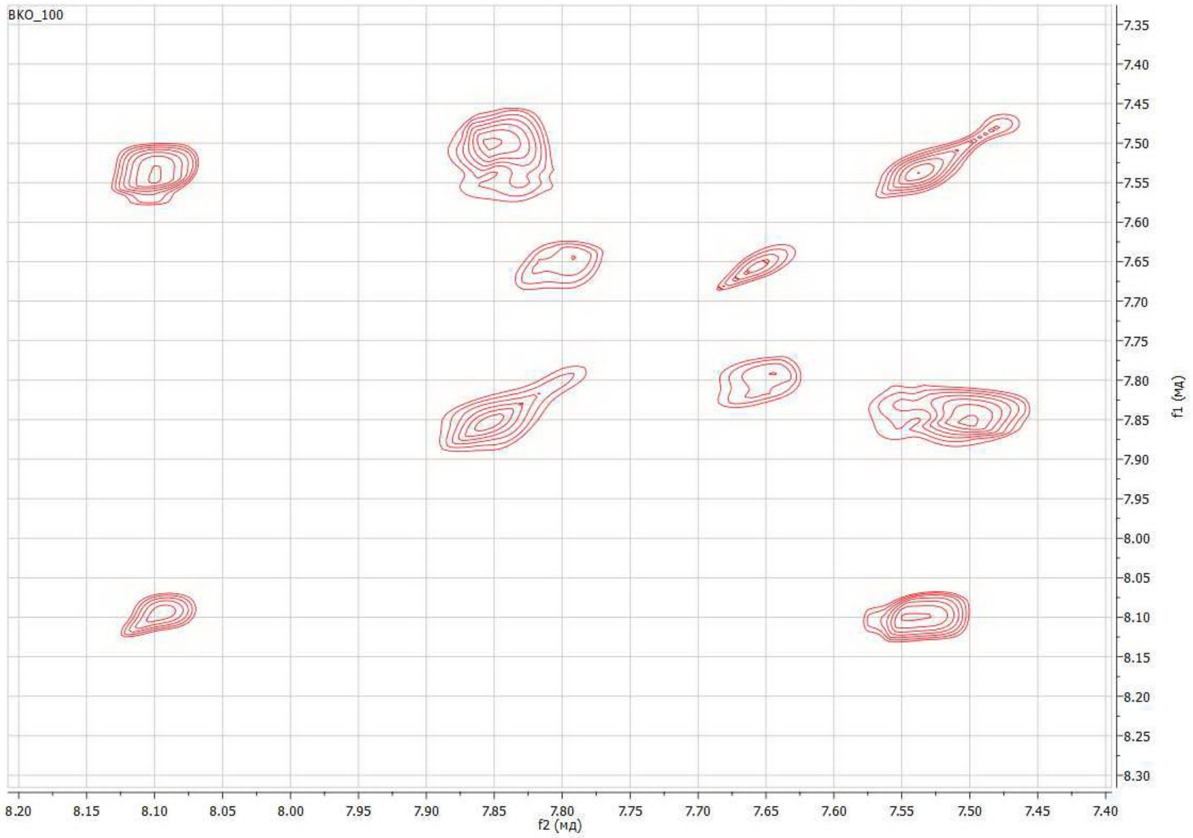

COSY NMR spectra of compounds **4e** (DMSO-*d*6)

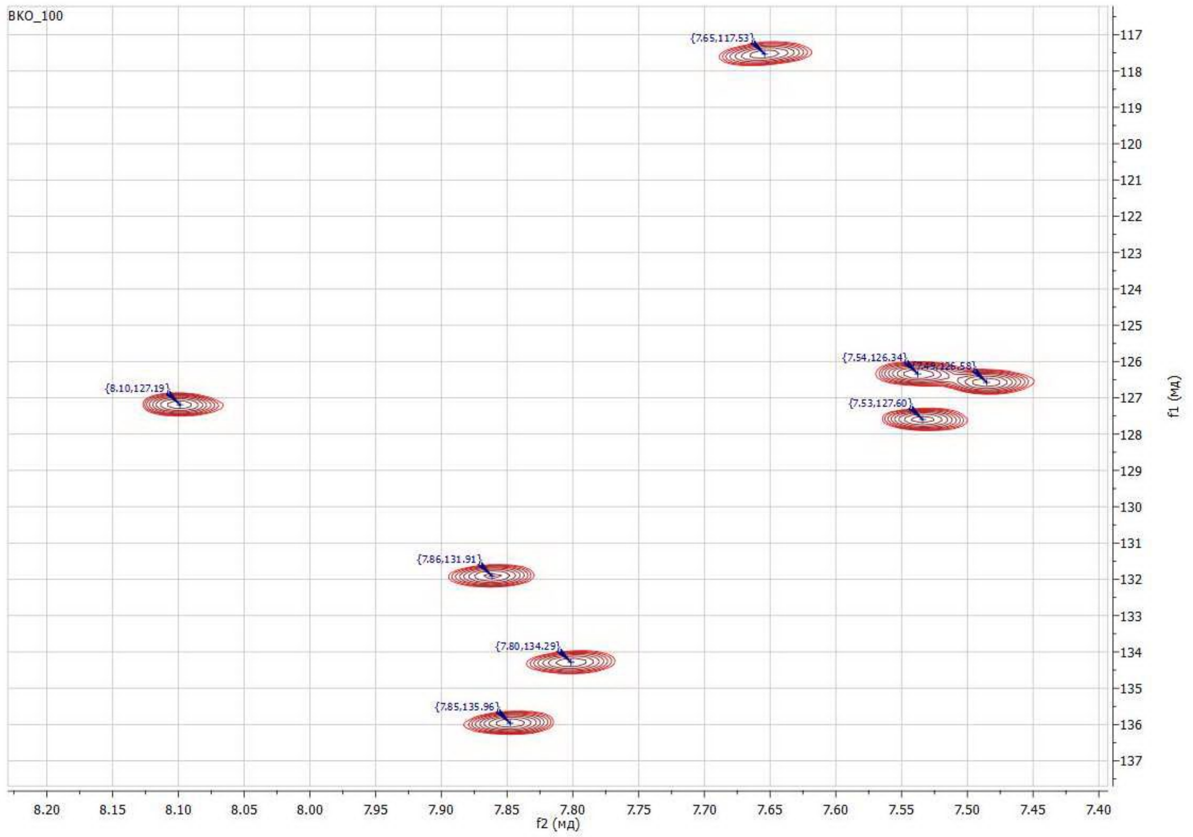

HSQC ^{1}H-^{13}C NMR spectra of compounds **4e** (DMSO-*d*6)

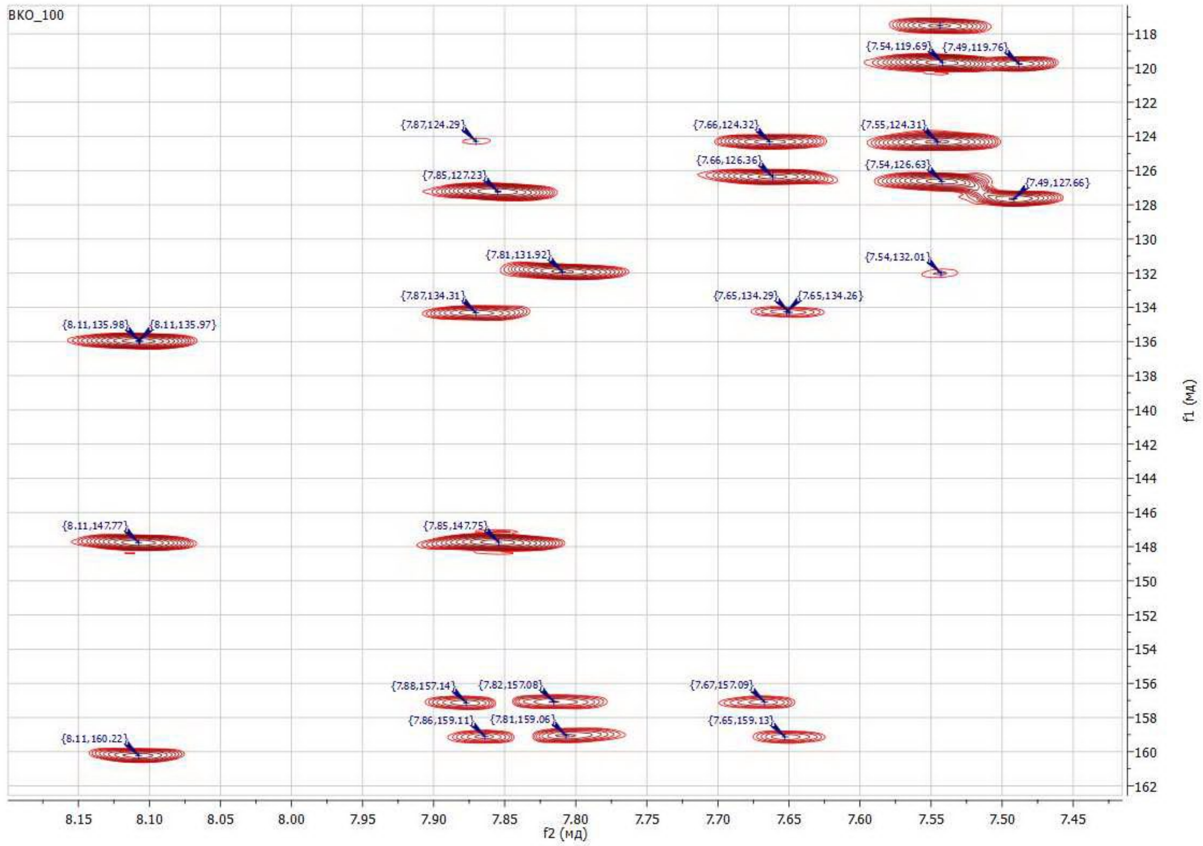

HMBC ^{1}H-^{13}C NMR spectra of compounds **4e** (DMSO-*d*6)

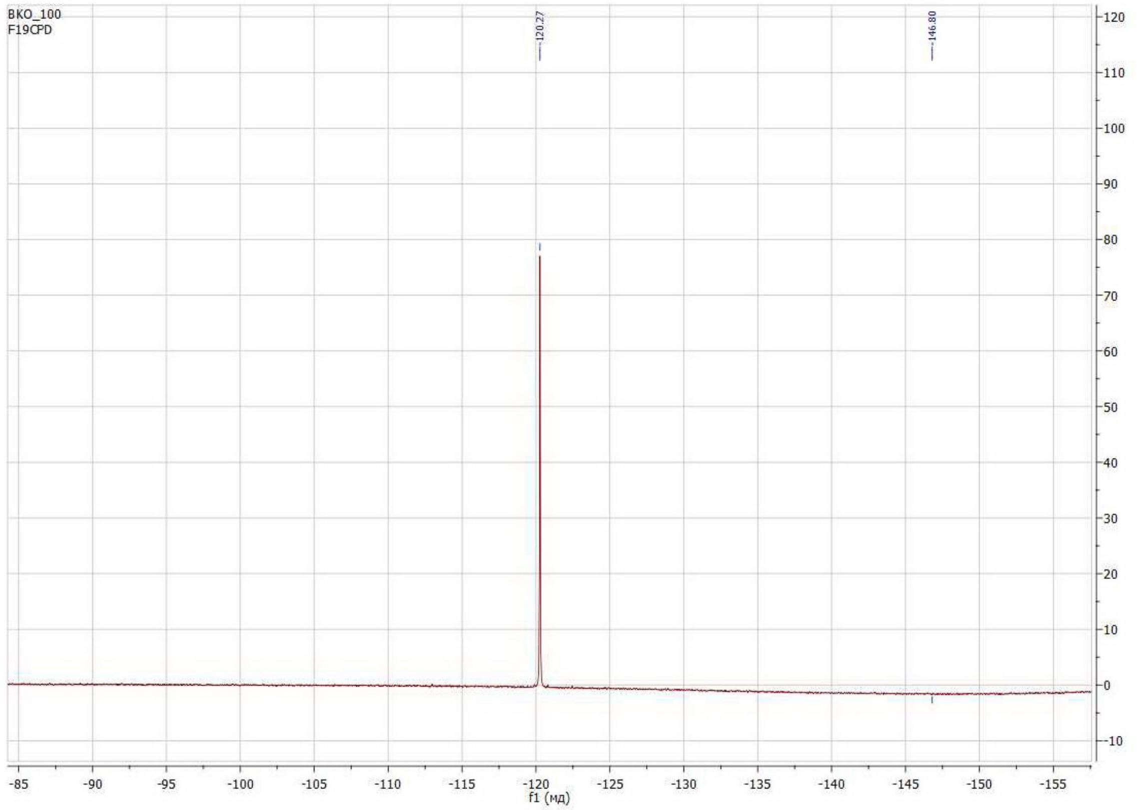

^{19}F NMR spectra of compounds **4e** (DMSO-*d*6)

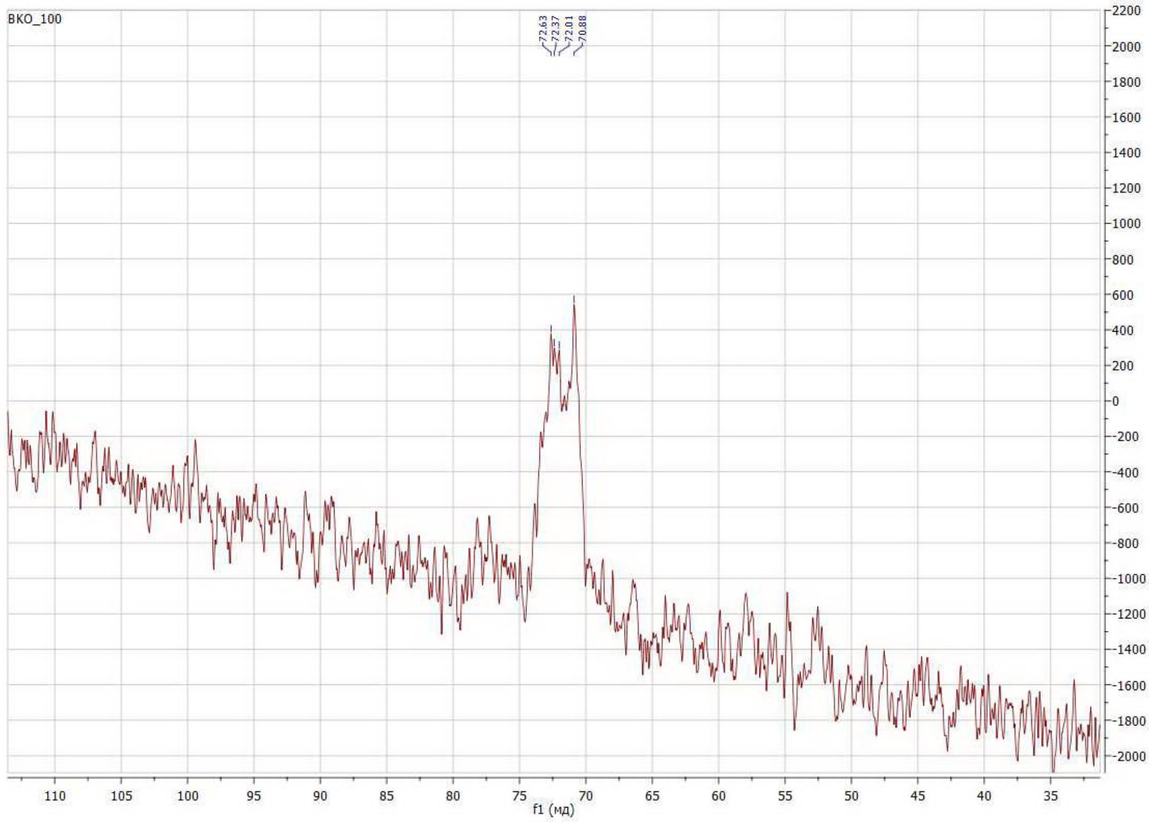

^{77}Se NMR spectra of compounds **4e** (DMSO-*d*6)

Display Report

Analysis Info

Analysis Name	D:\Data\Chizhov\Osmanov\Aug_25_2021\bko-100_&clb.d	Acquisition Date	25.08.2021 13:33:57
Method	tune_wide.m	Operator	BDAL@DE
Sample Name	/CHIZ BKO-100	Instrument / Ser#	micrOTOF 10248
Comment	CH3OH 100 %, dil. 200, calibrant added		

Acquisition Parameter

Source Type	ESI	Ion Polarity	Positive	Set Nebulizer	0.4 Bar
Focus	Not active			Set Dry Heater	180 °C
Scan Begin	50 m/z	Set Capillary	4500 V	Set Dry Gas	4.0 l/min
Scan End	3000 m/z	Set End Plate Offset	-500 V	Set Divert Valve	Waste

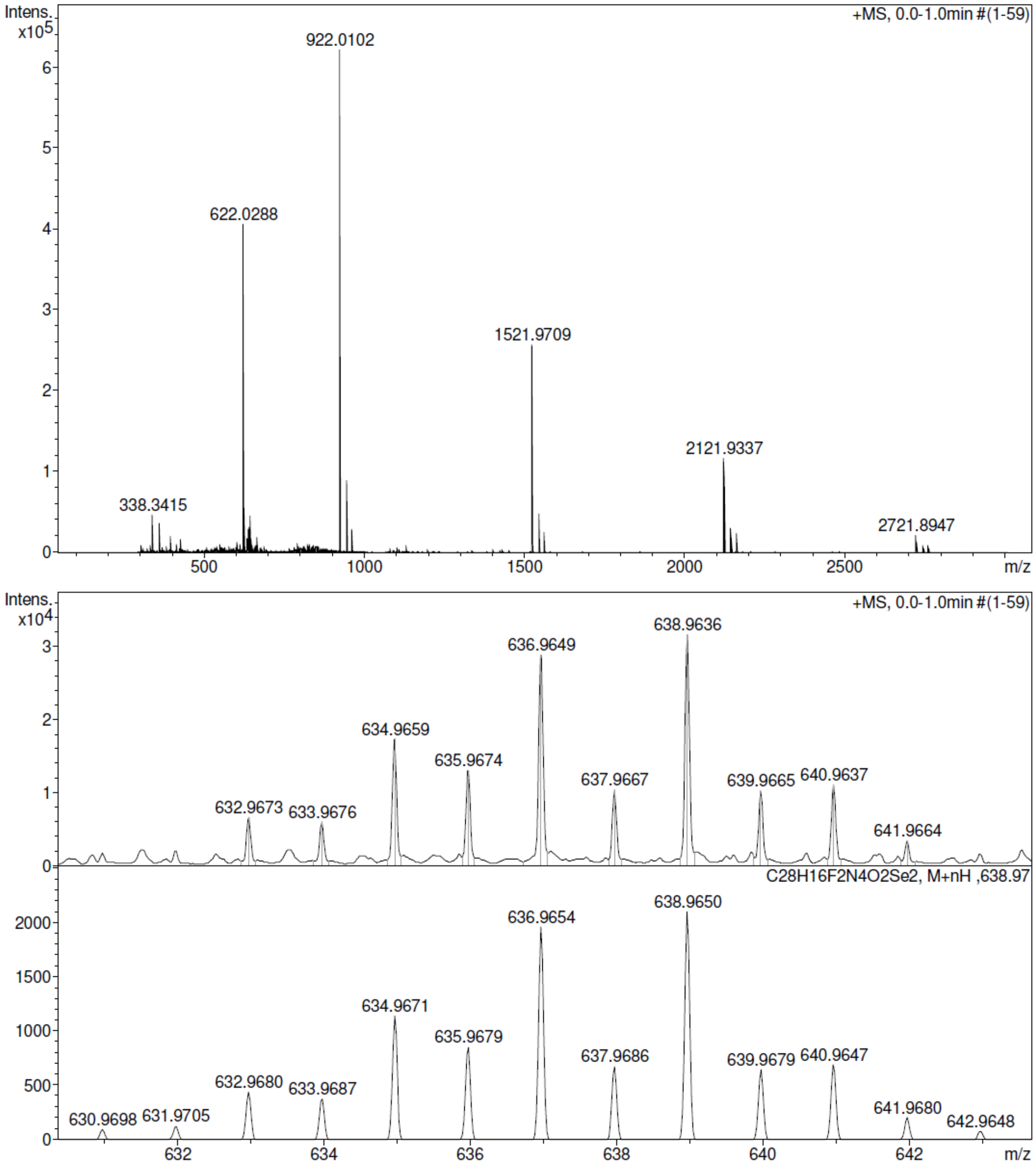

High resolution mass spectra (HR MS) of compounds **4e**

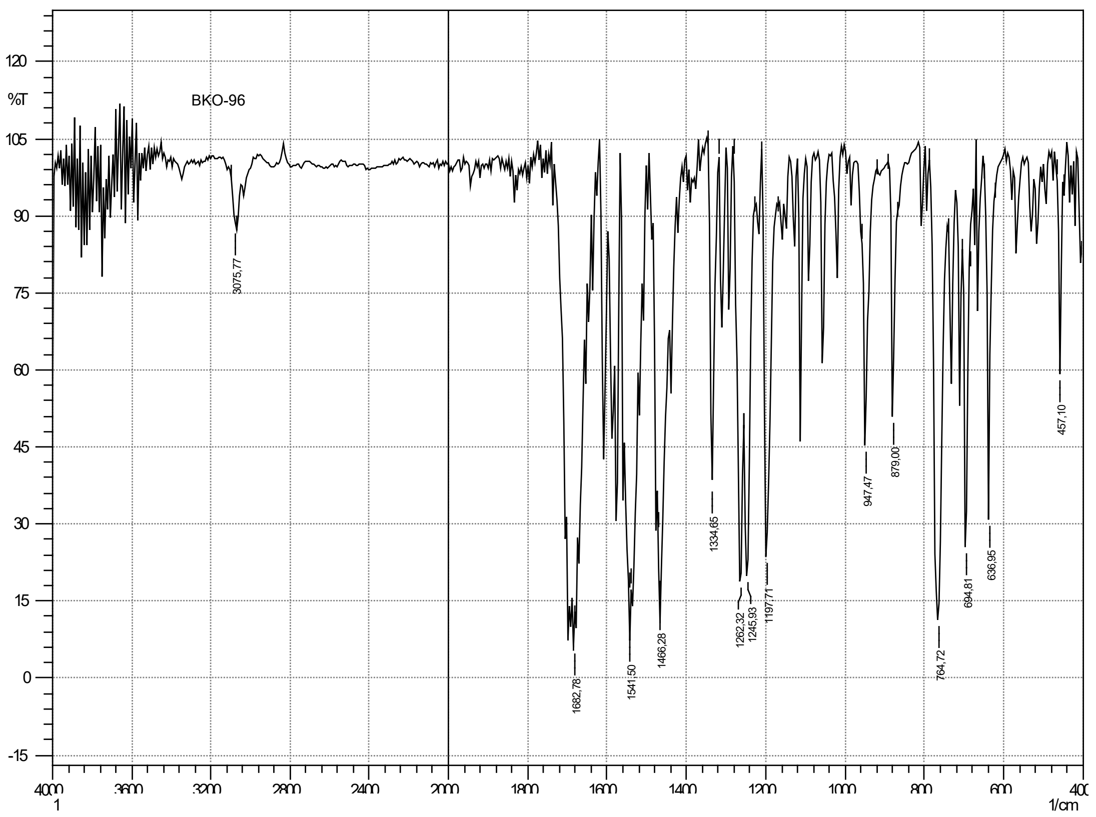

FTIR spectra of compounds **4f**

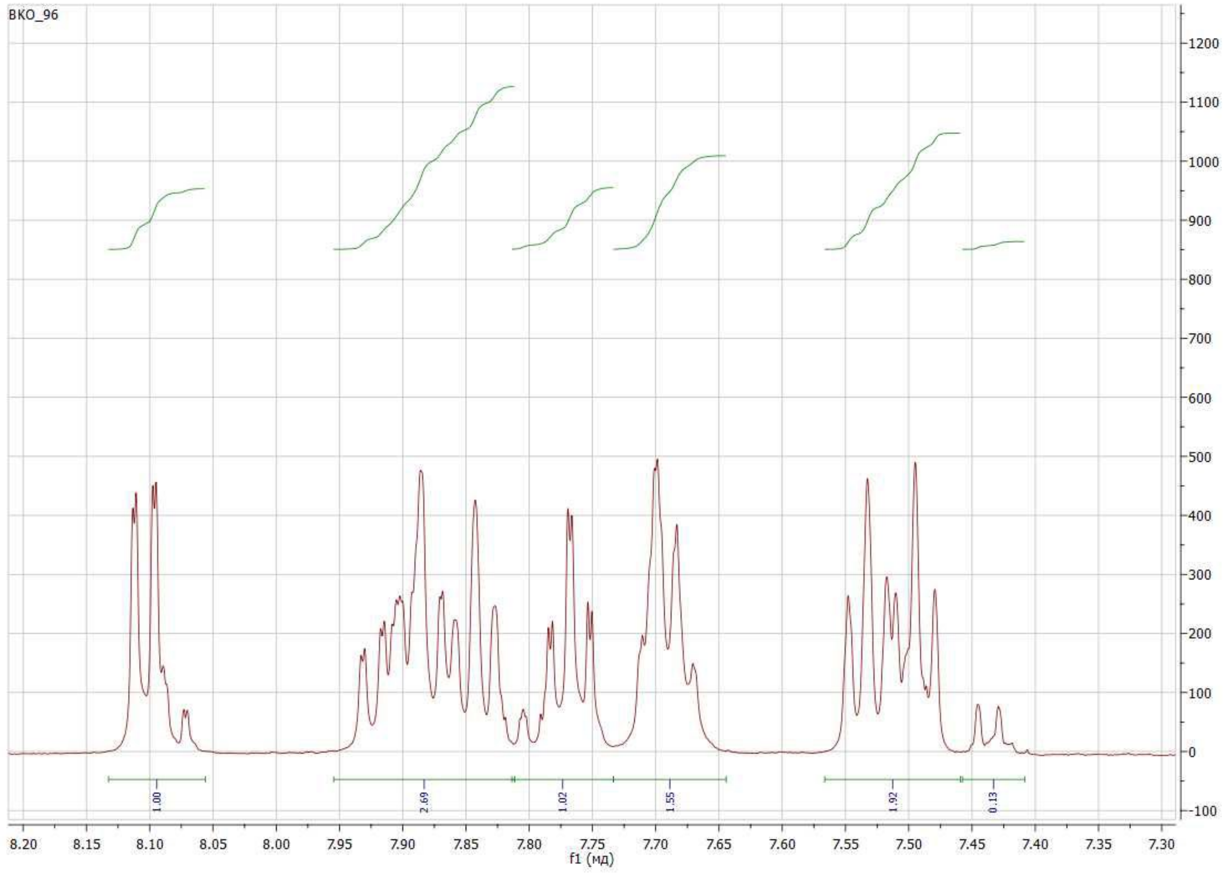

^{1}H NMR spectra of compounds **4f** (DMSO-*d*6)

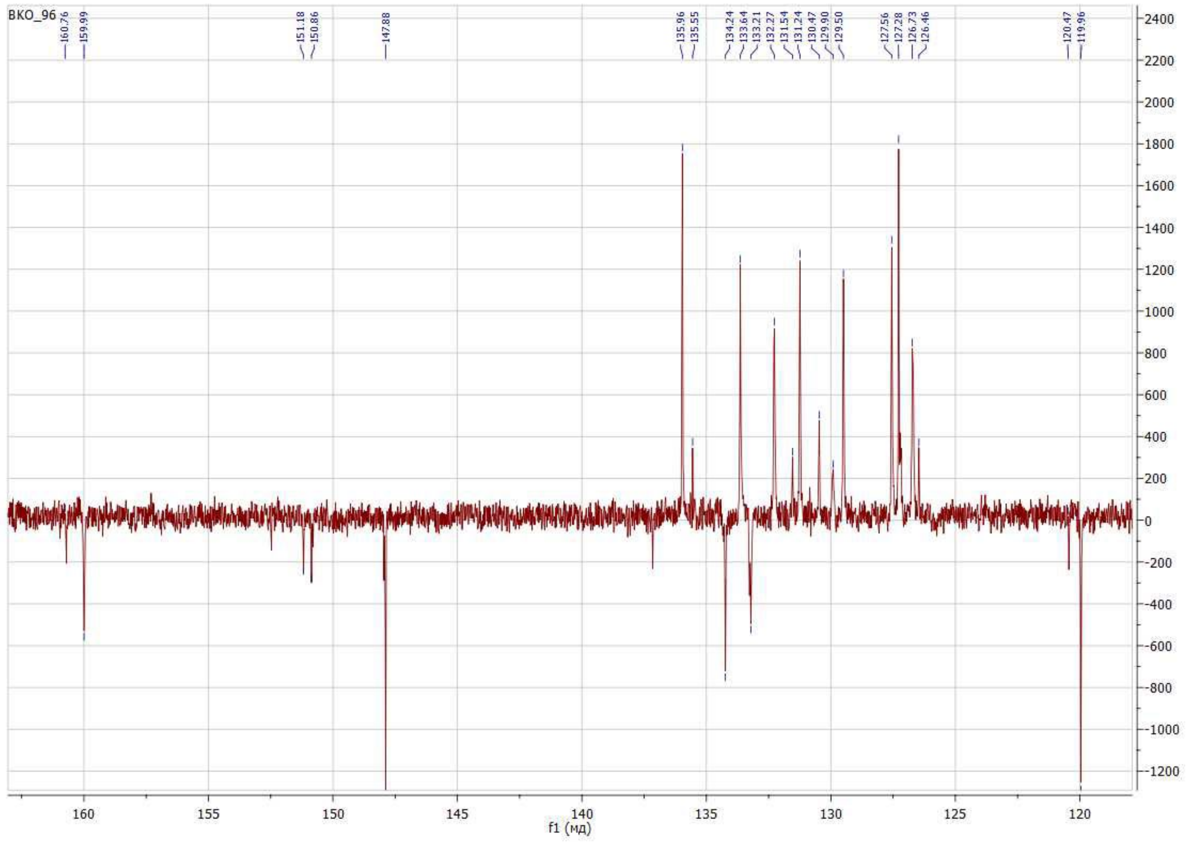

^{13}C NMR spectra of compounds **4f** (DMSO-*d6*)

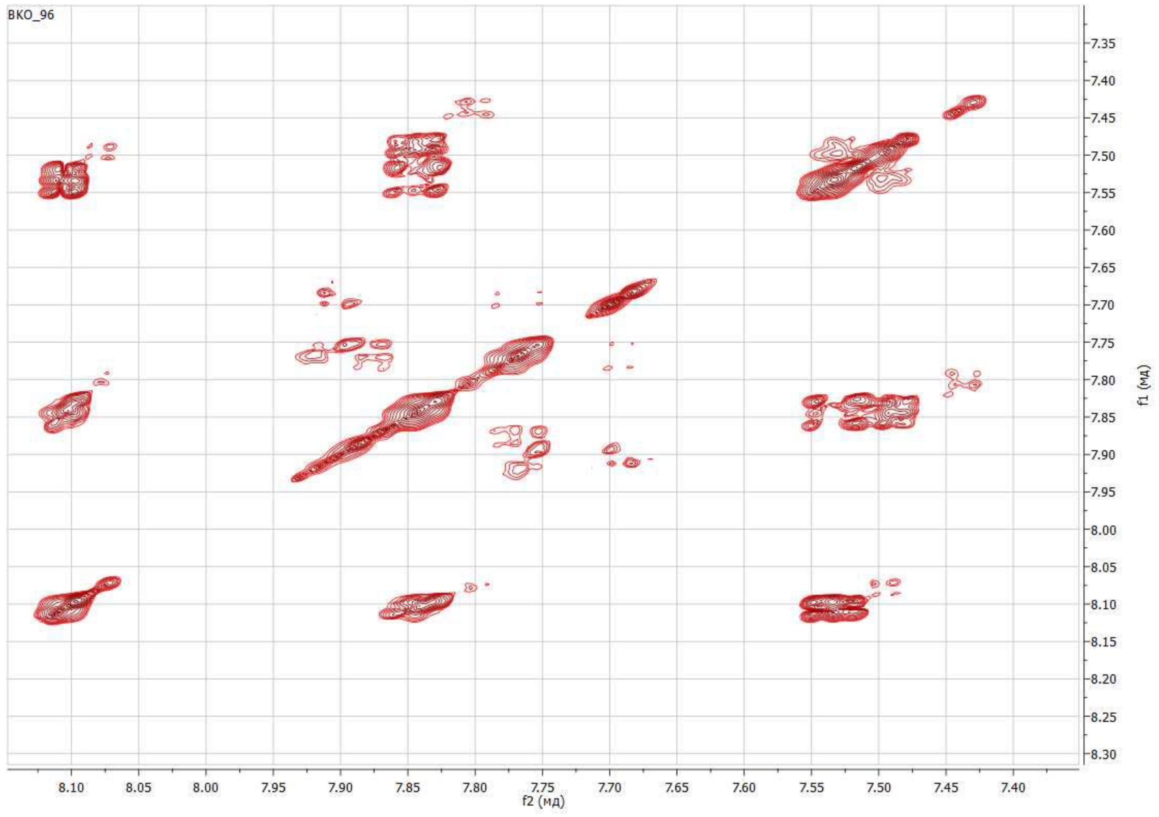

COSY NMR spectra of compounds **4f** (DMSO-*d6*)

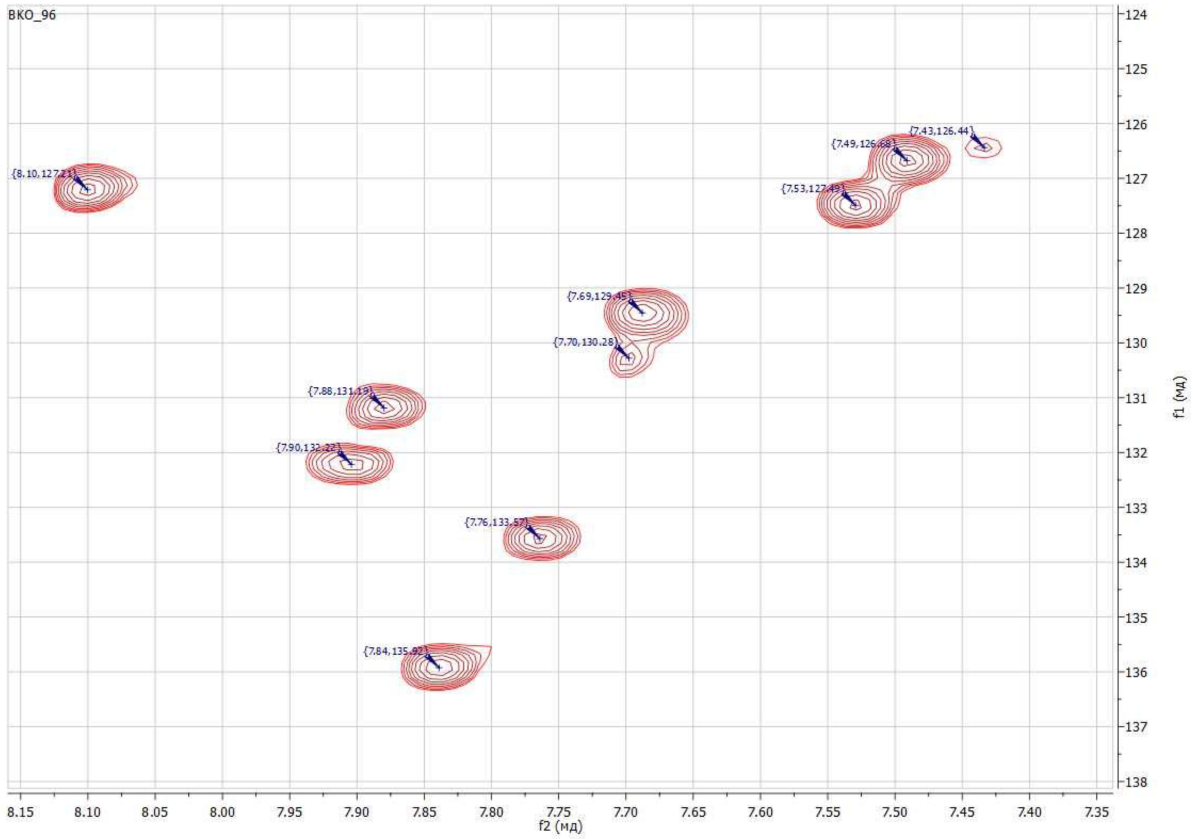

HSQC ^{1}H-^{13}C NMR spectra of compounds **4f** (DMSO-*d*6)

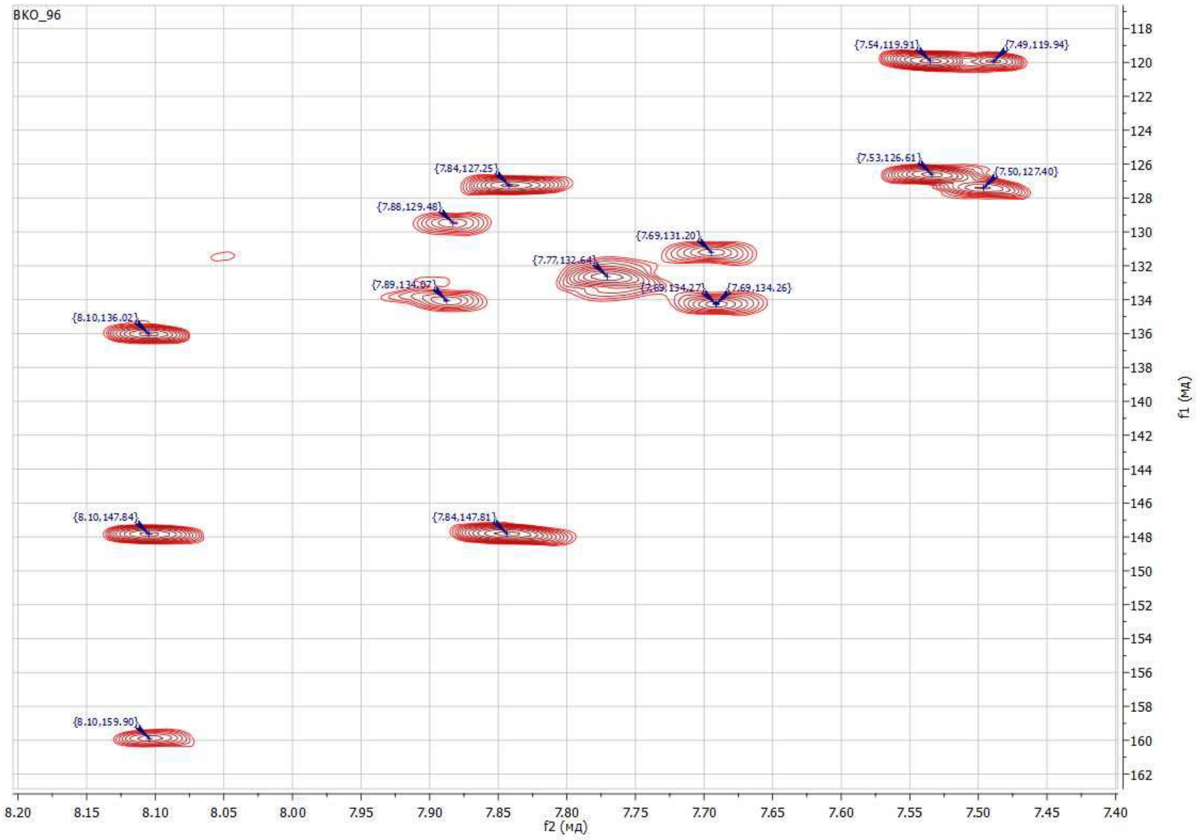

HMBC ^{1}H-^{13}C NMR spectra of compounds **4f** (DMSO-*d*6)

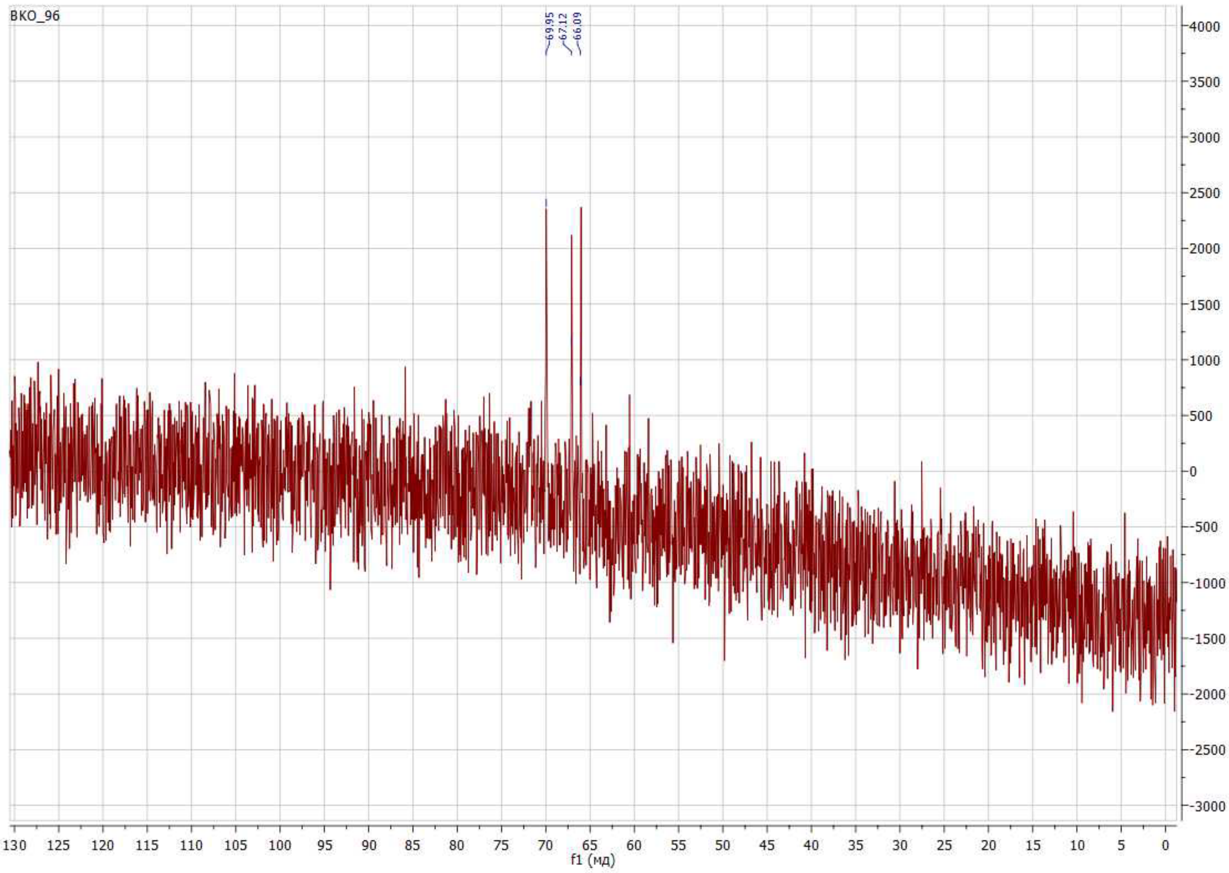

^{77}Se NMR spectra of compounds **4f** (DMSO-*d*6)

Display Report

Analysis Info

Analysis Name	D:\Data\Chizhov\Osmanov\Aug_25_2021\bko-96_&clb.d	Acquisition Date	25.08.2021 13:29:20
Method	tune_wide.m	Operator	BDAL@DE
Sample Name	/CHIZ BKO-96	Instrument / Ser#	micrOTOF 10248
Comment	CH3OH 100 %, dil. 20, calibrant added		

Acquisition Parameter

Source Type	ESI	Ion Polarity	Positive	Set Nebulizer	0.4 Bar
Focus	Not active			Set Dry Heater	180 °C
Scan Begin	50 m/z	Set Capillary	4500 V	Set Dry Gas	4.0 l/min
Scan End	3000 m/z	Set End Plate Offset	-500 V	Set Divert Valve	Waste

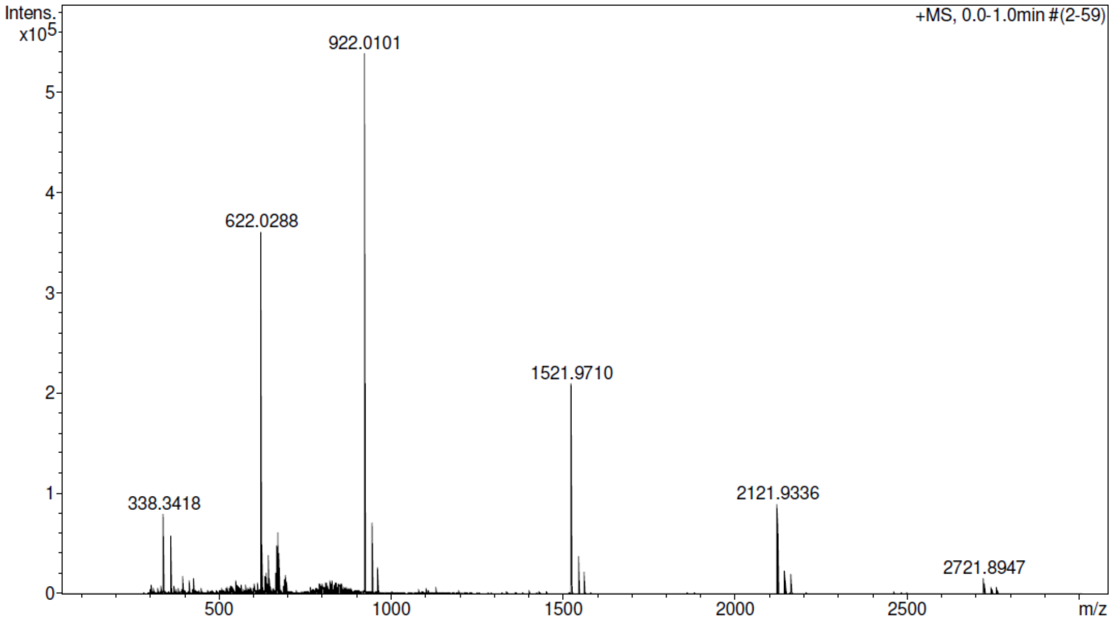

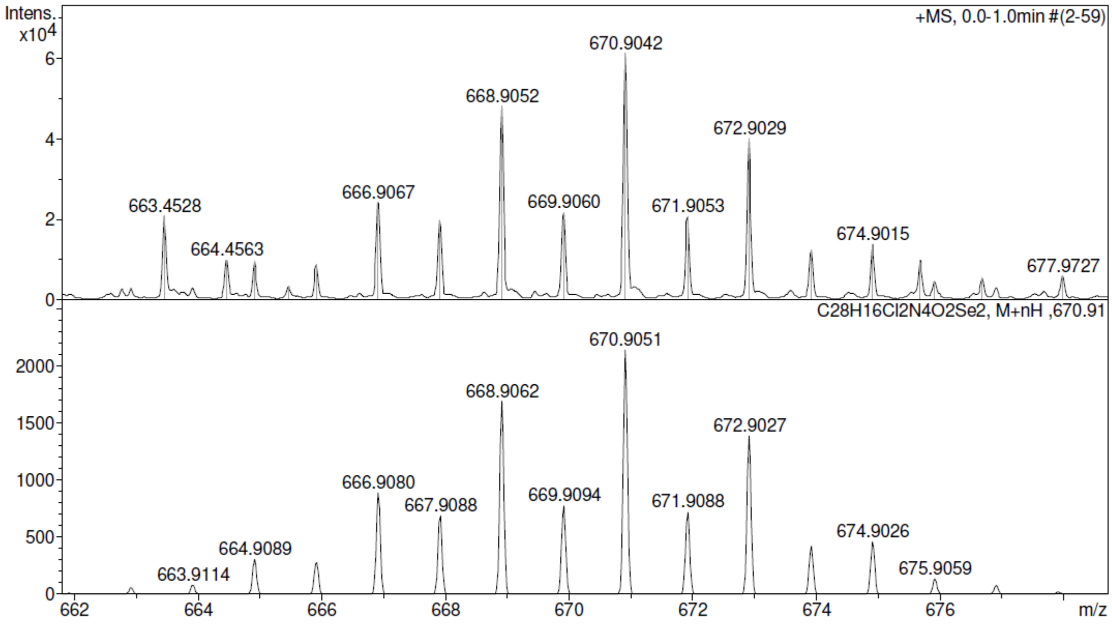

High resolution mass spectra (HR MS) of compounds **4f**

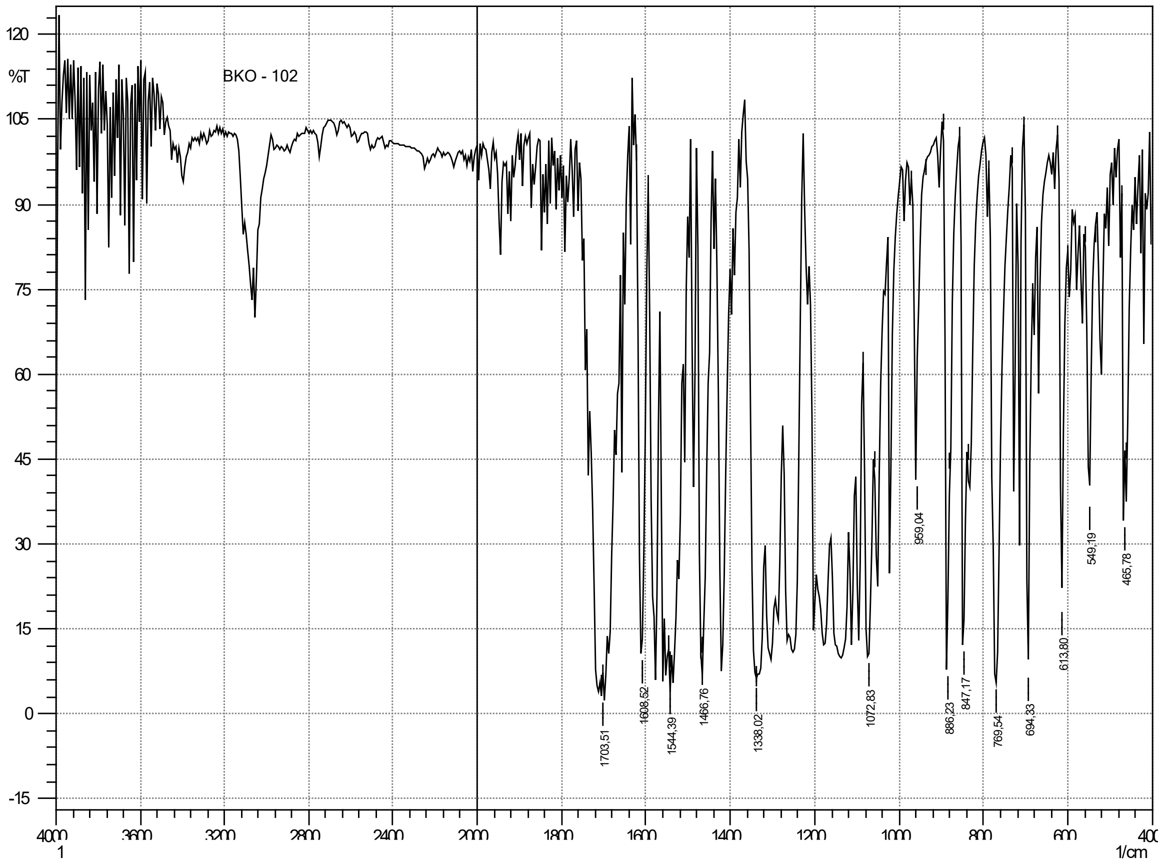

FTIR spectra of compounds **4g**

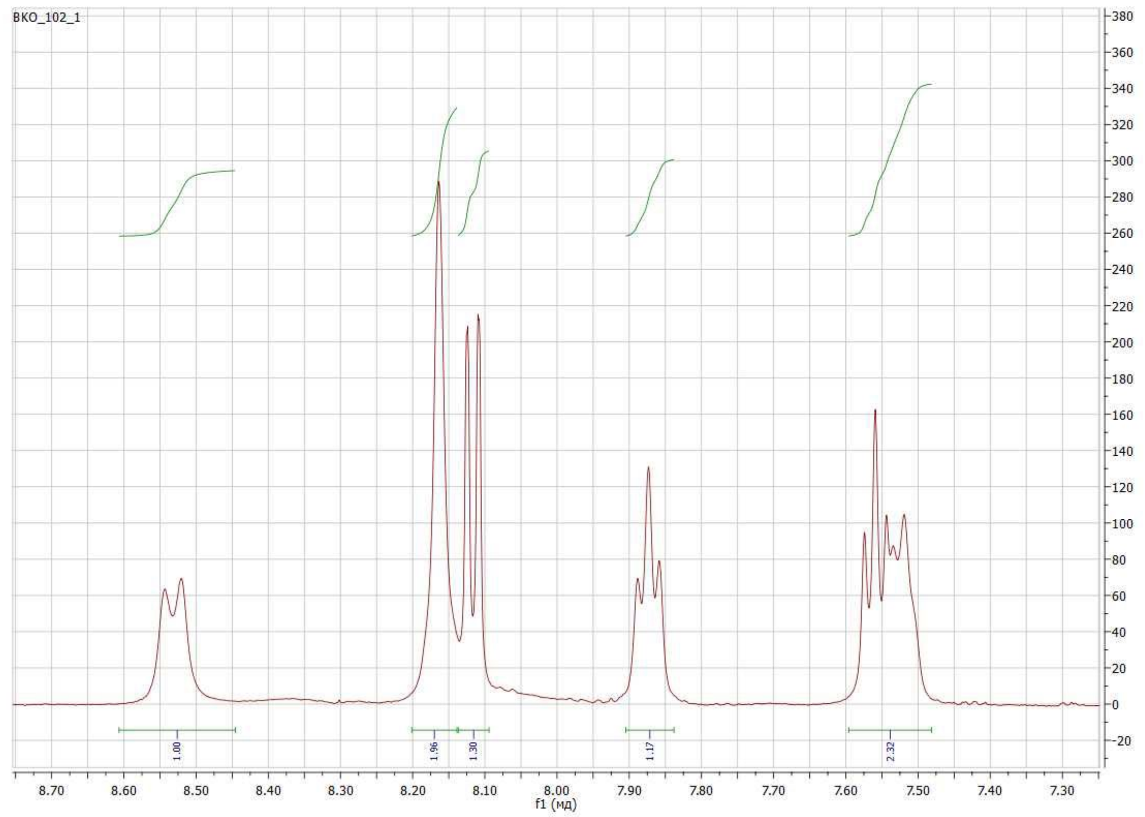

^{1}H NMR spectra of compounds **4g** (DMSO-*d*6)

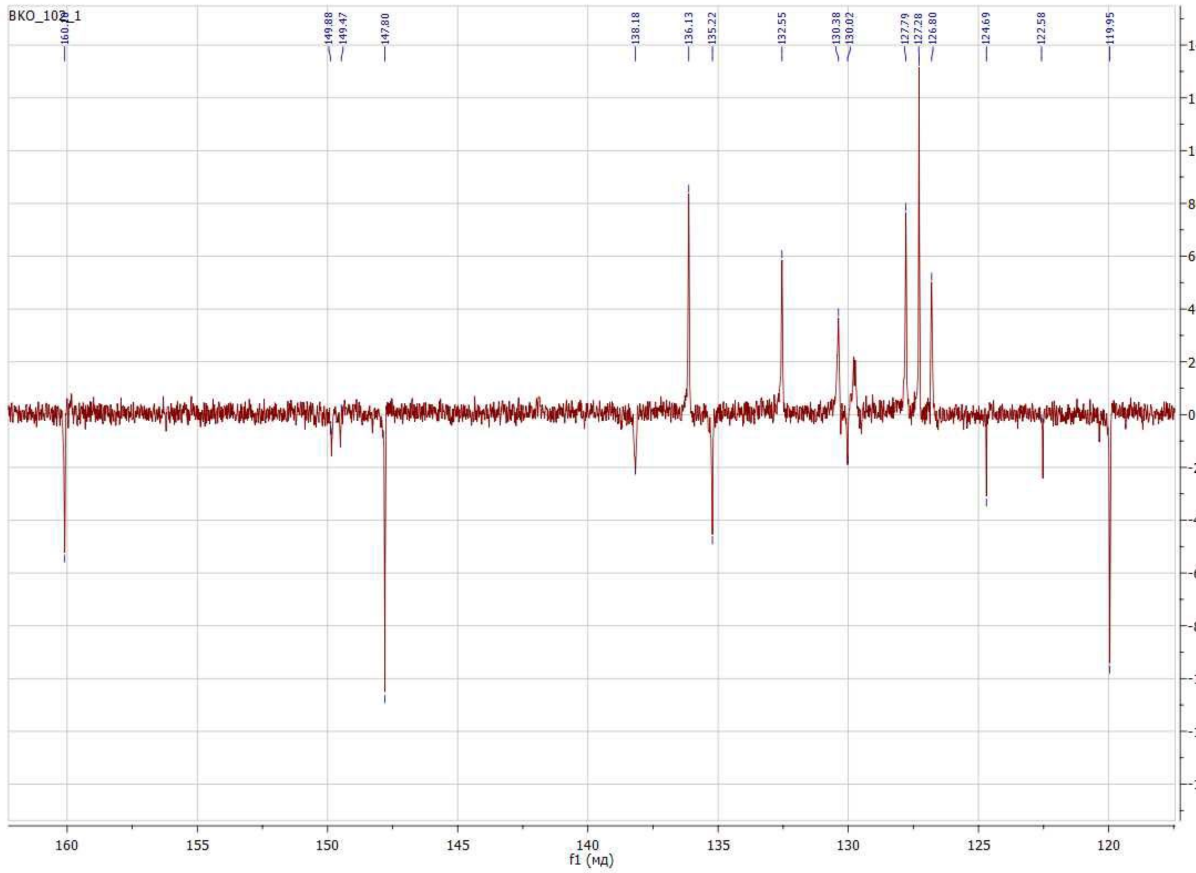

^{13}C NMR spectra of compounds **4g** (DMSO-*d*6)

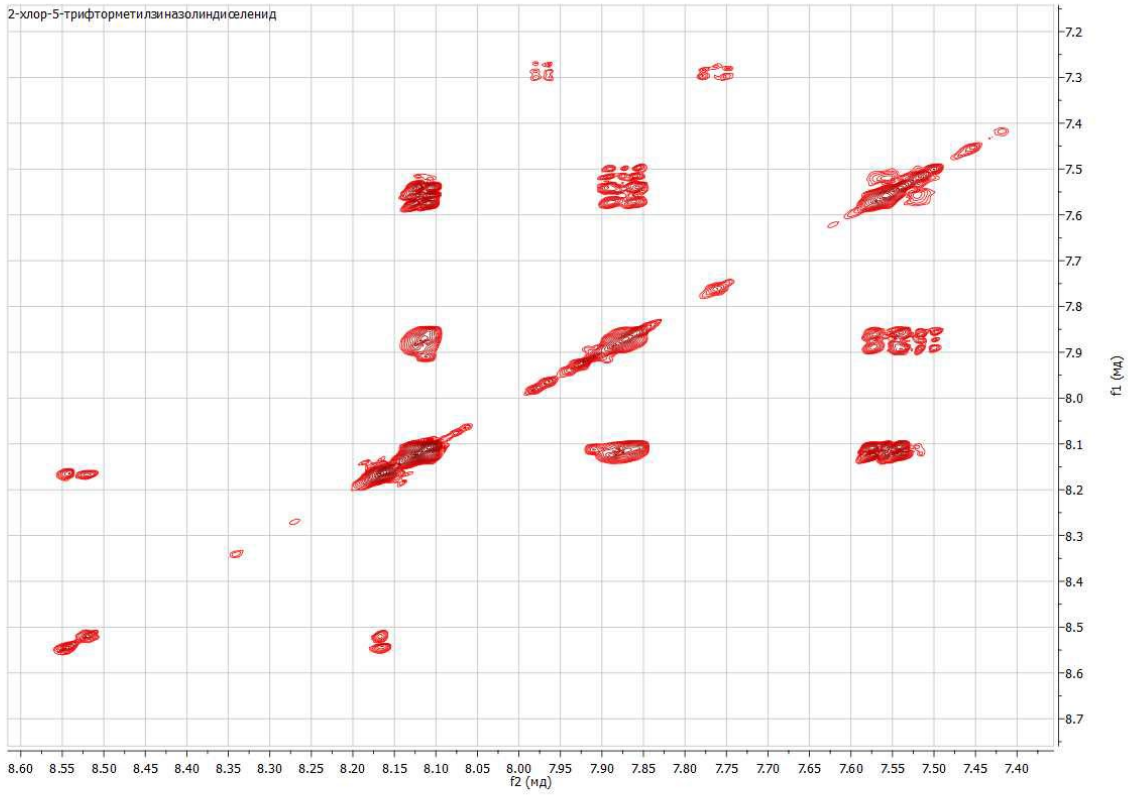

COSY NMR spectra of compounds **4g** (DMSO-*d*6)

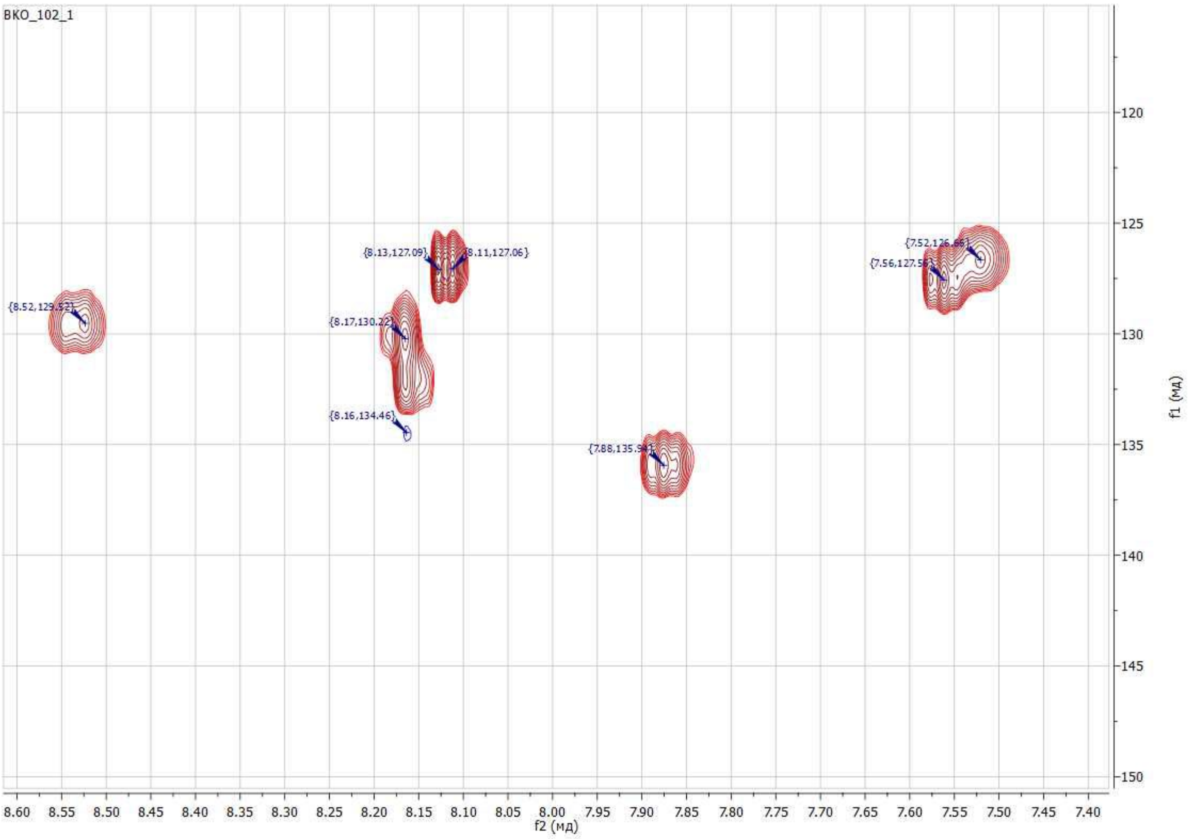

HSQC ^{1}H-^{13}C NMR spectra of compounds **4g** (DMSO-*d*6)

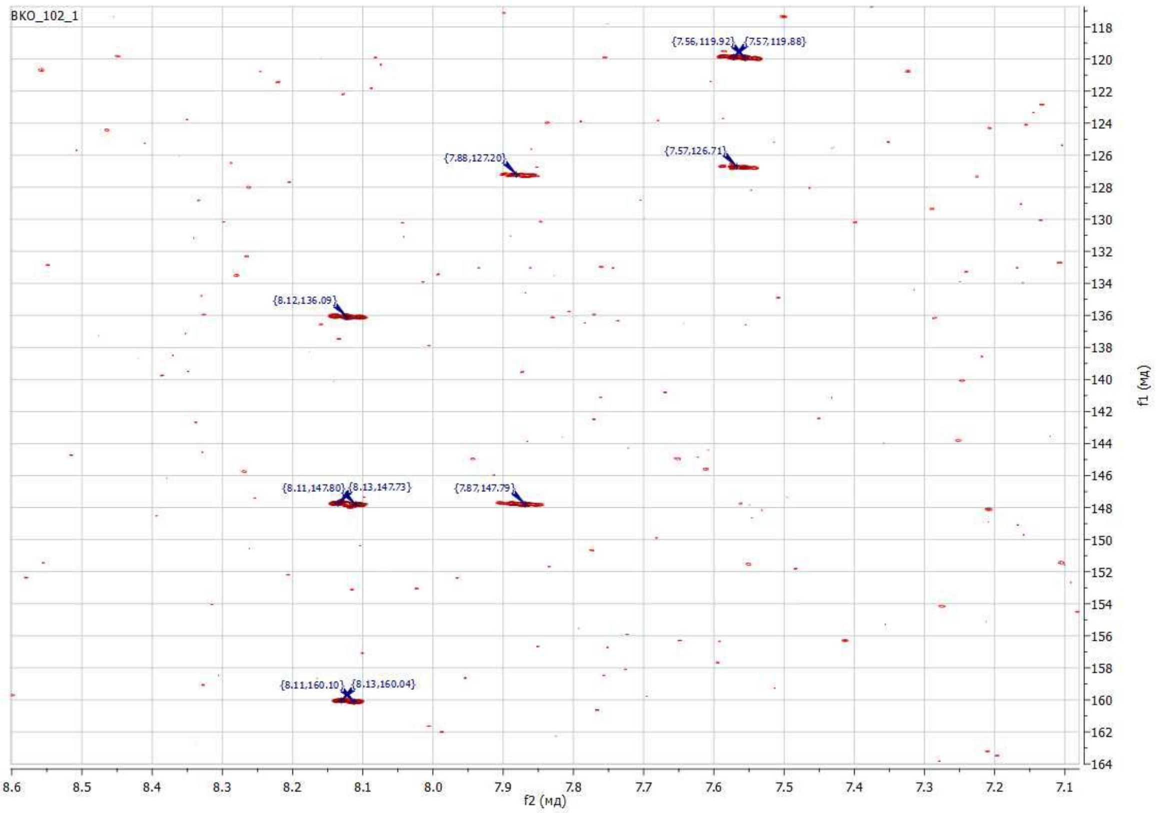

HMBC ^{1}H-^{13}C NMR spectra of compounds **4g** (DMSO-*d*6)

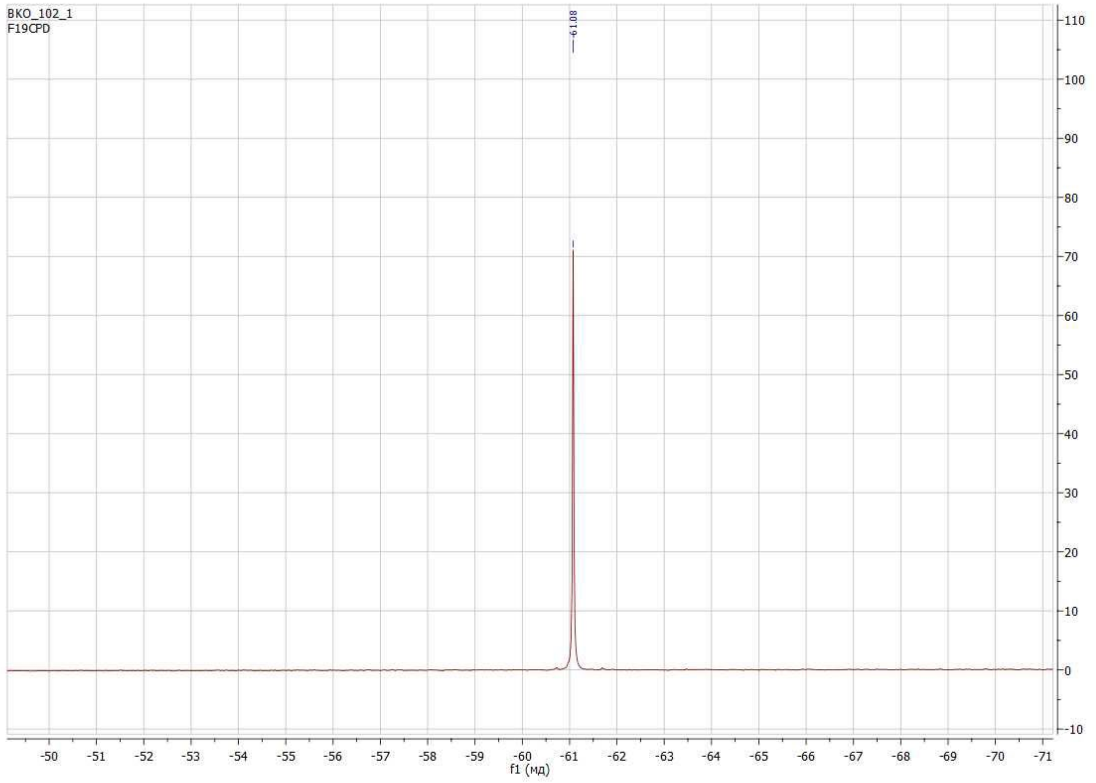

^{19}F NMR spectra of compounds **4g** (DMSO-*d*6)

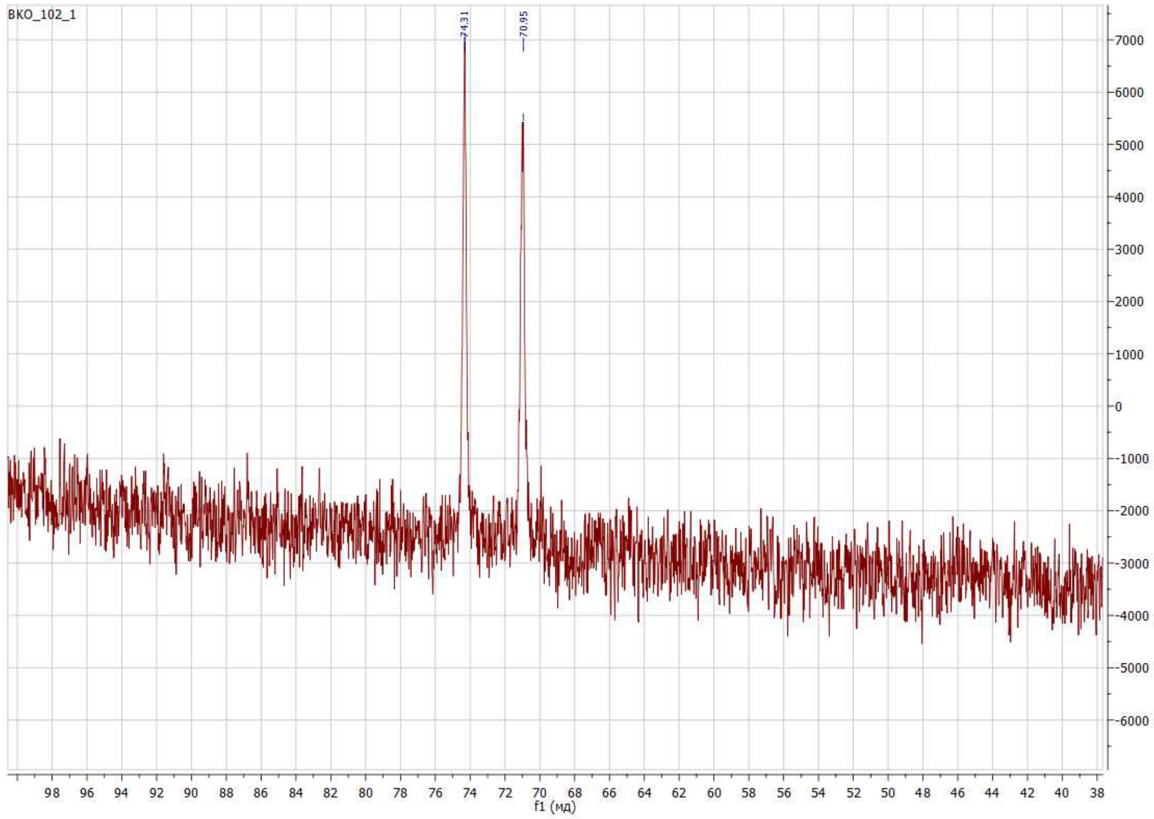

^{77}Se NMR spectra of compounds **4g** (DMSO-*d*6)

Display Report

Analysis Info

Analysis Name	D:\Data\Chizhov\Osmanov\Aug_26_2021\bko-102-1_&clb.d	Acquisition Date	26.08.2021 14:09:36
Method	tune_wide.m	Operator	BDAL@DE
Sample Name	/CHIZ BKO-102-1	Instrument / Ser#	micrOTOF 10248
Comment	CH3OH 100 %, dil. 20, calibrant added		

Acquisition Parameter

Source Type	ESI	Ion Polarity	Positive	Set Nebulizer	0.4 Bar
Focus	Not active			Set Dry Heater	180 °C
Scan Begin	50 m/z	Set Capillary	4500 V	Set Dry Gas	4.0 l/min
Scan End	3000 m/z	Set End Plate Offset	-500 V	Set Divert Valve	Waste

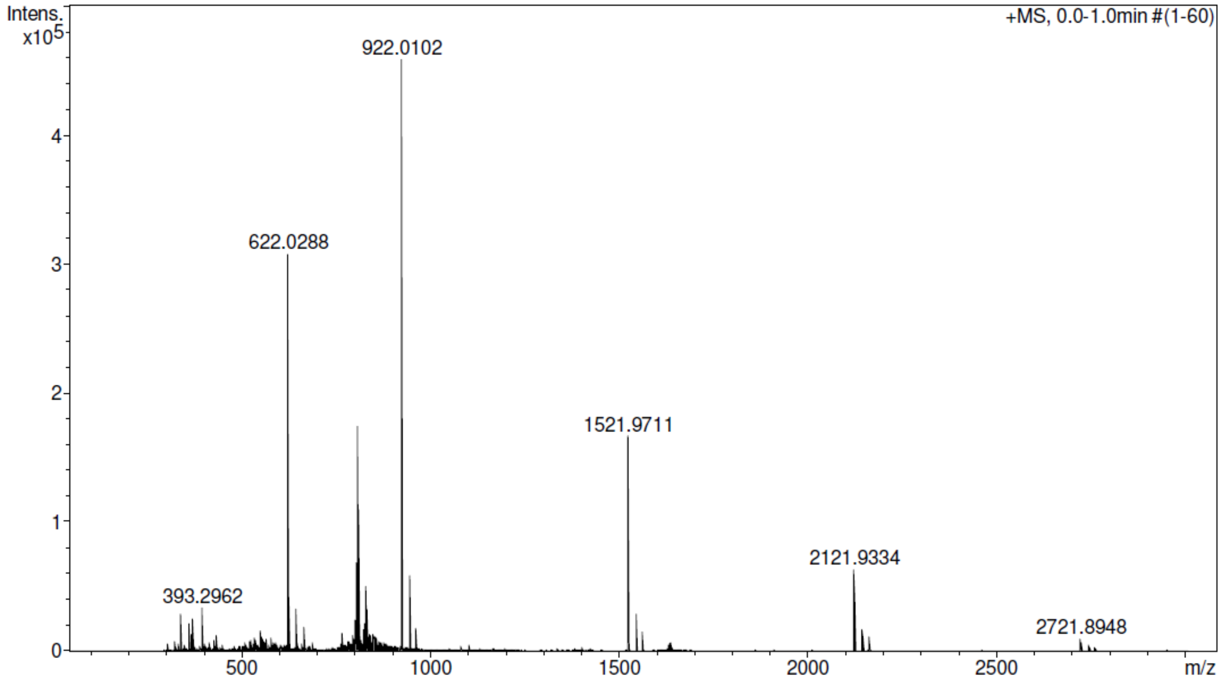

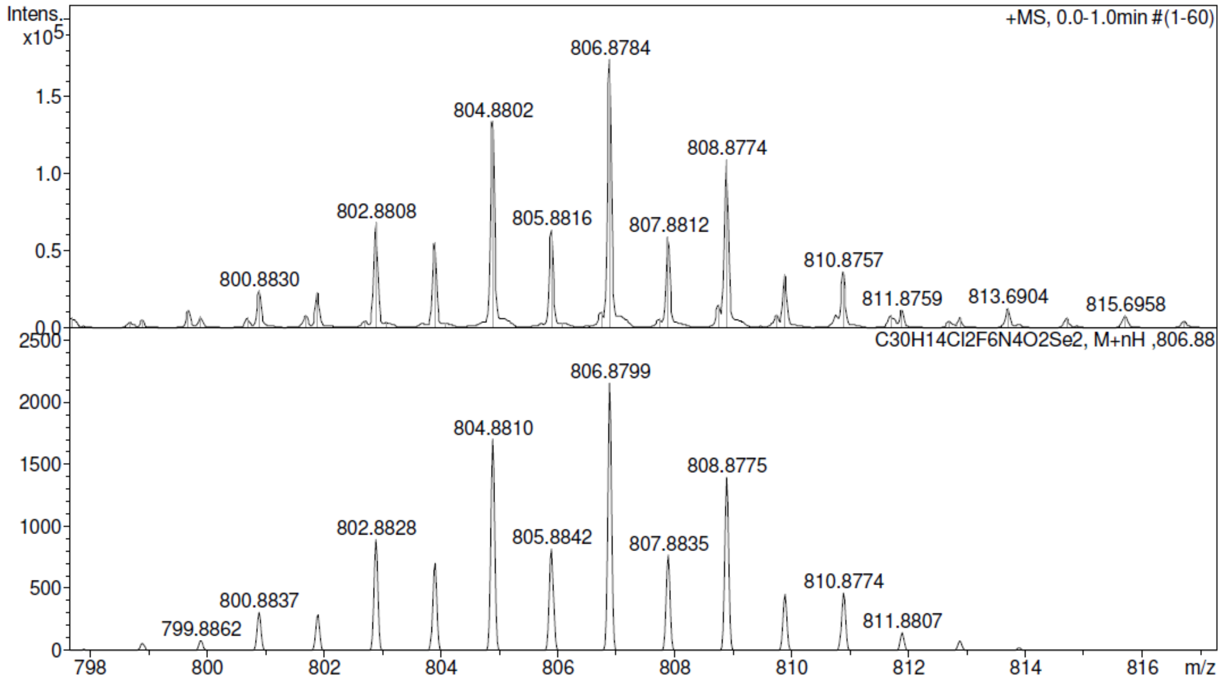

High resolution mass spectra (HR MS) of compounds **4g**
